# Supplementary material for: Evolution of Endogenous Retroviruses in the Subfamily of Caprinae
Source: Viruses. 2024 Mar 4;16(3):398. doi: 10.3390/v16030398 (PMC10975924; doi:10.3390/v16030398)
Supplement: Supplementary file 1 [file viruses-16-00398-s001.zip › 2-Supplementary File (2)-Dataset.pdf]

Supplemental_Dataset_S1

>CAP_ERV_1

ACACTGATTGAAACCGCCCACCCTGGCCAGGCACCATAGTAACCATTTGCATGAGTTGTT
TTATGACAGGAGATCCTGGTAAGGAATACAGAACTAATAAGCCATCACCAACTGGAAGAG
TCCAGGAAAGGTCGAAAGGAGATACTGCGTGTCTGTCCACTTCCCAGAATCCCTCTTGCT
AGCATCCATCTTGGCTGAGCGATGTTTGCGCCACCAGGAAAGACTCTGAATTAGAATGAT
TGGCCAAAGACCACCCGAAACTAATCCCATCACCATAAAACCTAACATTGTGAGCCATGT
GGCAGAGCAGTTCTCCTGGGTTCCCTTACCCAACTGCTCTCCACCCGGGTGCCCTTTCCC
AATAAAATCTCTCGCTTTGTCAGCACGTGTCTCCTCAGACAATTCTTTTCCTAGTGTTAG
ACAAGTGCCTGCTCTTGGGGCCCTGTAGCGGGGCCTCCCTCTTCCTACAACAACTGGCGA
CCACGAAGGGACACCCCCATTTTTACTGGGGCTGACATCCAGTCTGCTCGGGGTACTCCA
GGACCAGCTCACCTGCCGATGGATCTGACCCAGCGGCCGCAACTGGGACTCGTTTGTCCC
TGGTCTCCTCCTGACGCAGACAACTGGCCAGGGTGCCCTGACCAGGTAAGGAACAAGAGA
TTTTGTTGACCTCCTTTATCTCACCCCTCCTTTCAACCTATCCTATCCTATTTTTCTAGT
CCCCCAGTCCTGGATACAGAAATCTTGTCGAAGGGCCTCAGCCTGAGCTGAGGATTGGAG
ACTGATCACCTCCTCTTGGCAGAGAACTCGAACTCTGATTCTGATTCCAGTCCTCCCTTC
TCTGGAGGCCCAGGGAAAAGTCCCTTAACGCCTGGGTATCTGTAGGTGGCAGAAGACGTC
TGTAAGGCCACCCTTTTTTTCTTTTTCCTCTTTCTCTCCCCGACTCTTAGATCTTTTGTC
TCTTCCCACTCCTTCTCTCTCAACCTGGCCTCTCCTCCTCTCTTTAAAAACCCAAGTTAT
TTCCATTCACTCTGTCGATACTTGGACCTGAAGTTCTGGGTCTTTCCAAGAGATTTTCTG
AGAGGCTACGCTCTCTGCTCCCCATCAATTGTGCTTTCTATTCTTGGGGAGACAATCGGG
CGTTAGAAACCCTCCATCGGGTGCCCTGTTTCTATAAATTCAGTCATTTAATTGAGCATT
TGGCTGAGTGCTTCCACGTGGAAGTGATTGATGGGATTCAGTATAGTCACACCTGGTCTT
GGGTGAAAATTAACATCCTAGAAACATCCTTAGGGGTTAATTCACCTTTCAGGACGAGTC
CTTCAGCCTCGTTGATTGTGCTTACTACTGAACATTTCCTTTGGTGCATATACAATCAGG
CAATTTGCGACCAGGAGAGAGCTTACAATCTCTAGAGTCAGATATAGGGGCACCCACCCC
AATACTGGTCCCCTGGGAATGAGATATAAACTATACCATCACTAAAATGGGAAATAAGCC
TAGCTTCCCTTTAGACACCCTGCTGGGTTGTCTTTTGGCCCACTAGGAGGGATACTAACT
GGAAGGGCTAAAAAAGAAAAAACTTATCAAATACTGTACTCAATACTGGCCTACTTACTC
CCTGGGAGGAGGAGAAAATGGCCCCAGTTGGGATCACTTGGATACAACACCATTTTACAG
CTCAGTCTATACTGCAAACGCGAGGGTAAATACAAGGAGGGTCCCCATGTCCAGGCCTTT
GTGGCCCTCTACCAGGATTCCGGGAAAAAGGGAAAATATAAGCTTGAGGACTTTGAGAAA
TGCTCTTCCAAAATCCTTTTAGCAAGGTCTGAAATAGAAGATCCCCTTGACCTTCCTTGA
CCTCCTCTGCTTCAGCAGAAAGGTCACGGGGAAAGGAGGGTAAGACACACCCGGAAATTA
CGAGCTCGGACCTAGAAGTCAGGAGGCTGGGTCCAGAAATCATGGGATCTGAAGGGAAGT
CACTCACCATCACCTCGGTTCCTCCTCCATATGCCCCCTCGCCCCATGGGTTTATGATCA
GGAAGTAAGCTTTGTCCTCCACAGAACGAAAGGTCTTCCTCAGACAAAATCTGCCCCCTA
AGAGAGGTACCAGACAGGGAGGGGGGAACAATCCACATTCATGCTCCATTCTCTATGCAA
GACATTACCCAATGCAAGGAACAACTGGGCTCATATTCCGAGAACCCCCCCAACTTTAAG
GACGAATTTGAATGTCTTAGCCTTAACTTCTCCCTCACCTGGATGGATATAATGGTCATC
CTCACCCAGTGTTGTAATGATGAGGAAAGAGCTCGAATCCTAGATCAGGCTAGAAAAGTG
GCTGATGAGAGACAGCGGGCAGATGTCAGTCTGGCCCCTGCCGAAGAGGCAATCCCCTCT
ACAGAGCCAGACTGGGACCCTAATACAAGGGCAGGAGAGGAATCCGTAAGACACCTCATT
ACTTGCCTACTACAGGGAATGACCCAGGGTGTCCGAAAGGGTGTTAATTATAATAAGATA
AAAGAGGTTACACCGGAAGAGAATGAGAACCCAGCTCTTTTTTTGGGCAGACTCACAGAA

GCCTTTAAAAACTTTACCAAGACAGATCTAAAAATCATGGAAGGAAGAGTCCTGTTGGGC
CATTCCTTCATAACCCAGGCAGCCCCAGACATAAGGAGAAAATTACAAAAACTAGGAAAG
GGACCAGAGACTCCAATTTCCGATTTGGTGGAGGAGGCAAATAGGGTGTTTTTGAACAGG
GACCAGGAGGAGGAAGCAAGAAGGGAGCAAAAGGAAGCCTGAAAAGATAGGAGAATGGAG
AGGCAGACCCAGGCCTTGGCCCAACAACAGGCTAAAATCCTGGCTTTGATTCATCCTGCC
ACTATGCGAGAGTCTGGGGTAGAGCAAGGAAGGAAAAAGGATCTTAACAATCCACCGTGT
CTGAGACACACCCAGTGTGCCTACTGCAAAGAGGATGGGCACTGGAAAAGGGAGTGCCCA
TATTACCCTAAGGGAAGGCGTATGGAGACCCCTAAACCTGTTCCCTCTGTATTAGTCCTG
GGCGACCAGGACTGACAGTACTCAGTGGCTCAAGAAATCCCCGTGAATGGTGAGGTCCAG
ATCACTCCTCTCGACCCTTGGGTGACTCTACAAATAAAAGGTTAAGGATATGAACTTTCT
TTTAGACACGGAGGCTGCCTTCTCTGTTCTTCCTTTCTGATTAGGACTTTTAGACTCCCA
GATTAAAATGGTTATAGGGGTAGATGGAAAACCCCAGAGCCGAAATTTTACTAAGCCCCT
CTGCTGTAAGGTGGGCAATTGGCAAGGAACCCACCCCTCCTTGTACATCCCTGGTTGCCC
CACTCCCCTACTGGGGAGAGACCTTCTTTGCTGCCTCCAGACCAGAGTGACTTGGGGAAA
GCTAGAAGTCAATAATTTCCTTTGCCTAGTGTCTGAATGTTTACAGTTAGATGTAGAAAA
GGAGGAGTCTGTCCCTTTCTTAGATGAGGTCAATCCCCAGGAGTGGGATATCTCCAGCCC
TGGTCTAGCCATAAATGTTCCACTGGTAAAGATTCTTCTGAGGCCAAATGCTCCCTACCC
CTGGAAAAGACAATATCCCTTAAAACCAGAAGTTCTTGAAGGTCTTGGACCATCAGTTAA
CAAATACCAAGATACTGGAATTCTAATCACCTGTGAATCTCCCTGCAATACTCCAATCTT
GCCTGTTAAAAAACCAGATGGATCTTATCGATTCATCCAGGATCTAAGTGCTGTTAATGA
AGCTGTGGTTCCCATCCACCCCATAGTCCCAAATCCGTATACACGCCTATCTCAAGTCCC
AGGAAATGCTAAGTACTTCTCAGTCTTGGATCTTAAAGATGCCTTCTTTTGTATTATTCT
CCATCCTGAATCCCCAAAATTCTTTGCTTTTGAGTGGCGGGACTTGGAAACAAGGGAGGC
CATATAACTCTGCTGGACTCGACTACCACAAGGGTTTAGGGACAGCCCTCATATTTCTGG
AACTTCCTTGGGGAGAGAACTAAGGGAATTAACTTCAACCAACAGTAATCTAATACAATA
TGTGGATGACTTGCTCATAGCTAGTCCAAATTTTACCAGCTCTCAAATGGACACTATTAA
AACTCTAAATTTCCTGTATGAAAAGGGTTATCGAGTATCCTCCAGGAAAGCTCAGATTAG
CTTAACCCAAGTAAAATACCTTGGATTTATAATTATGGAAGGAAAAAGAATGCTCGACCC
CCAGAGGAAATCCCTCATCTTAAATACCCTATACCCCCAAACAAAAAAACAGCTTAGGGG
ATTTTTAGGAATGACTGGGTTTTGCAGGATCTGGATTCCTAATTATGGCAATCTGGCCCA
ACCCCTATATGAAAAGTTAAGAGGGAAAGAGGAAGAGCCACTCGACCAGGATGAGACCTG
CAAGGTGGCCTTTAATGCCCTAAAAGAGTCTGTCACTACAGCCCCAGCTTTAGGCCTCCC
AAACCTGGAAAAGCCTTTGAGGCTTTATGTTTCTGAAAGGATAGGAACTGTTCTTGGGAT
GTTAGGACAAATGATGGGGCCTGTATTACAACCCATGGCTTATCTCTCAAAACAACTAGA
TGAGGTGGCCAGAGGGTGGCCCACTTGCCTCTGGGCAGTAGCGGCCACCACTCTTATGGT
TAAGGAGGCATCTAAGCTGACCCTGGGTCAGCCCACCACAGTGTATATGCCTCACCAGGT
GCAAGCAGTCTTGGAAACTAAAGAGGATAGGTGGATGACAGGGAGAAGGATCACACAATA
CCAAGCCCTCCTCCTTGACACTCCAGAAATAAAGTTGAGGGTCTGTCAGACTTTAAATCC
AGCCACCTTACTGCCAGATCCCCCTACTTCCCCTGTGGATCATCAATGCATTCAAATCAT
AGACGAGTTATACTTTTCTCACCCAGACCTATCAGAGACACCTTTATGTGACCCAGAGGA
AAAATAGTACACAGATAGCAGTAGTTTTGTAGAAAAGGAAGAGAGGAAAGCAGGATATGC
TGCAGTGAGCCTAGAAGAAACTAGGAAAAGTGGGATTCTTCTCCCAGAACCTCAGCCCAG
AAAGCTGAACTTTTTGCCCTGCCAAGAGCTTTGGAATTAGGGGAAGGAAAGAGGATTAAT
ATGGACTGTAAGTATGCTTTCCTCATCCTGCATGCCCATGCAGCAATTTGGAAAGAAAGA
GGGATGCTCAGTGCTCGAAGCTCTCCTATTAAACACAAGGAGCTCATTCTCAGGCTCCTG

GAGGCAGTCAGACTTCCTGCTAAATTAGCTGTCATCCACTGTAAGGGTCACCAAAAGGGG
CAAGAAGAGGAGGCCCAGGGAAATAGAAAAGCTGACCAGGAGGCAAAACGAGCTGCTAGC
TGCTAGATAGCTGTCACTGCTATCTGCCCCCTTTTCCCCAAGGAAACCCTGACTCCAGAT
TATACCCCAGAGAAACATTCCCGATAAGCTGAGCGGGGCTGGGAGATCGGGTCACATGGG
TGGTTTCAGACTGATCAGGCCCAAGTAATACTCCCTGACTCTCAGGTTTGGAAAATTATT
AACTCCTTACATAAAAGTGCCCATTTTGGAAGAGATAATCTGGAAATCTTGCTCAAGCCT
ATTCTCTACCATCCCCAGTTGGCTAAGGTTGTTAGGTCACACAGAACTGTGATACTTGTC
TAAGAAATAATCCAAAGACCAGACCCTGGGCACCTCCTCTAATTAAACCTGTCCAACTTC
GGGGATCATACCCAGAAGAGGACTGGCAGGACTTACAGCCATGCCAAAGACCCAAGGGTT
TTCTTATTTGCTAGTCTTTATTGACACGTTCACAGGATGGATTGAAGTGTTCCCTACTAA
GACAGAGAGAGCCACAGAAATCTGTAAAGCTCTGCTGAAGGATACAGTACCTATGTTTGG
GTTGCTTCGATCACTTCAGAGTGACAATGGGCCCTCATTTACAGCCACGATATTGCAGGA
CCTGGCCGCATGCTCAGGCATCAAATACCGCCTCCACTCACCCTGGAGGCCTCAAGCTTT
GGGAAAGGTAGAGAGAGCCAACCAGACTCTTAAAAGGGCTCTGGCCAAGCTATGTCAAGA
GACACATAATAAGTGGATCCATGTGTTACCCATAGCCCTCATGAGGGTACAGACAGCCCC
TAAATGGCCACTATTACTAAGCCCTTAGGAACTGATGTATGGATGTCCATTCTTAACTTC
TGATTTGTTTTTTGATGAGGACAGCAATACTCTCTTAAAACATATAATTGACCTGGGAAG
GTTTCAACAAGAACTCCAACGATATGGAGAACAGATCCTCCCTAGACCACAGGAAAACTT
GAAAAATCCCCAGGTAGAGCCGGGGGACCGAGTATTAGTAACGACCTGGCAAGAAAAAGG
GAGTCAAAGCCAGCTGTCCGAGAAATGGACAAGACCATATCAGGTTGTCCCAGTAACTTC
AACTGCTGTAAAAGTGAAGGGTCTATCTGCCTGGGTCCACAATTCTAGAATAAAACCCTA
TGGCCTACAGGAAGGGGAGACAGGGAATGAGACTCCAAAACCAGAGGACAACTATTCCTG
TGAACCTGTTGAAGATCTCAGACTCTTGTTCAGGTGGAACCCAATCATCTCTCCCTCAGA
TAAGTAAAAATGCATCATATGATGTTCTTCCCCTTTCTAATCATGTCTCCTTGTATGGCT
ATTAATCTCTCCCTAAACGAGGCATATACGCTTGCAAACCACACTGCTTCTCTACTCAAC
CTAACCAGCCCCTGCTGGATCTGCATTAAAGGCAGGAGTTTGGGGTATGTCAACCTGTCC
CAGGGTATCAGTGGCCTACCCTTCCAGCCGAGCTACAAGCTGTTACCCAGCACACCCCAT
CTGGTCACAGTATGACCTTTTGGGAAAAATGGGAAAAAAACTTCATGCCCTGGTATCAGC
AACAACTGTCTATGTTTACCCCCTCGATAATTACAGACCCACCTGAGCCCTTATTTTCTC
TTTTGACCAACATTCCCCAAGTGACCTTCCCTACCTGTCTAAAAAGTGACTCCCTGACAG
GGGTGTCAGTGAGAAATCTAGGCAACTCACAATATGCGGTGACCTTCACTGTTGAAACTG
ATGGGAAAAACTGGGACCAAAAATTTCACTAGGGTATTACCATTTAGGCCAAAGTCTGGG
AACTAGAGATTCTGGCAGAACTGACATACTATATCTATCCCTGAACATAAGGCTCTCTTC
AGATGCTATTAACCAGTCTCAAAAATCCATGTCCTGTTCTGGACGACCAAAGAACCACAC
CCATTCATCCTGGTTTCTAAATCGGGCAGGCAACTCTGACCCCTGTTATTCCCTTGTAGG
TGTACCCACCCCTGAAAACCTAACCCTATGGTGGGAGCCTAAACAAAATACTCTTTACCT
TGAGAACAAAACCCAGGATGCCTCACAACACTTAATCCCTTCCTAAATGCCCTCACTGCT
GCCGGTTTTGCAGCAAGTGGAGTGATAGTCTCCTCCCAGCTACTAAGAGACTGCCCCTGT
TCCATTATGCCACGTTCCAGTTGTAAGGCAGATGGAAATCCGGGTTTGCTGCCCAGGTTT
AAACCCAGTCATGGGGTCTATTTCATATGTGAGGATAAAGCTTATTTATCCCTACCACCT
TTTTGGTCAGAAGCTTGCTCCCTAGGCTATGTTACACCCCACATAGATCTGGCCTGGAGT
AATACCTCCTTGCCTTTACCAGTATATACCAACCAGAGGATACATCGTGCAGTAATACTT
ACCTCTCCTTTCCTGGCATTAGGACTGACTGCTGGTTTGACAGGAGCTGCCATGGGGGGA
ACTTCTTTACATAAATTTCAGCAGCTTTCTACCAACACTGCTGTATCCATAGAAAAAACA
GCGAGGACCCTCCAATGCCTACAATCCCAGCTAGACTCCCTAGCCGCAATGGTCCTACAA

AATCATCGGGCCCTAGATTTACTGACTGCAGGACGGGGGGCACTTATCTCTATCTAAAGG
AAGAATGCTGGTTTTATTACAACCAATCTGGGCAGTTCCAAGAAGATATCAAGGGGCGTT
TGGAACAGGCCACAAAGATCCAGGATTTGAGTTCAACAAGTGTATGGAGTTCTCTTTCTT
TCCCTTCTTGGTTCCTACCATTCATGGGTCCAGTGATAACTATCTTATTAGGCCTCCTAT
TTGGCCCCTTATACTCCAACTCCTTACAAAGTTTATTTCCTGCAGACTCCAGAAGTTCCA
GGCCAAATTGATATTACTACAAGGGTAGAAGCTGGTTTCAGATGTGGAACACCCCAACTT
AGATCAGGTAGAGAGAGAGACTTCTGCCCTGCTAGGCAGGCCTACACCCATGCACAGCAG
GAAGAAGTTACAGAAGAAAGAGACCTCCGCCCCAATTCCCAAGAAATATCTTGAATATGA
AGTCTCTTGGGGGAATTGTCAGGAGAAGCAACACTGATTGAAACCGCCCACCCTGGCCAG
GCACCATAGTAACCATTTGCATGAGTTGTTTTATGATAGGAGATCCTGATAGGAATAAGG
AACTAATAAGCTACCACCAACCGGAAGAGTCTGGGAAAGGTCGAAAGGAGACACTGTATG
TCCGTCCACTTCCCAGAATCCCTCTCACTAGCATCCATCTTGGCTGAGCAATGTGTGCAC
CACCAGGAAAGACTCTGAATTAGAATGATTGGCCAAAGACCACCCGGAA
>CAP_ERV_2
TGTTGAGGCCTGATAGAGGCCATGACTAAAATGGGCTGGAGCAAGCATTCCGGAAACAGC
AGAGACACTGCCCAGGTGGGATCAAAATTAATTACCCCTGACGGGAGGACTCAGCCTGTC
AGCATGCTCCCTGTAGCCTGGTCTGTGCCAGCCGGGAGGAGGATAGATATCTCCTAAGAA
AGCCCGCATGCGCGAAAATCTAGCCAATTAGCAGATGCAAAGAAACCCTTGTAACCAATC
CAACCTTGCCAATTCCCTGTCTTTGTCCTAAACCTATAAATACTACTGTAATATAGGGCT
CTGGGCTCTGGCTCTATTCCACTGTGTTGGATGCAGCCAGGGCCCTGGCTCAAGCTAGCA
ATAAATTCCCTTTTTTGCGTTTGCGTTGCCGTGGATGTCTTGTTCTCTCAGTTCTGGGGA
TCGGGACCTTGGGCATAACATTTGGGGGCTCGTCCAGGATCCCTTGACTGAGAAGAACAA
CACCTTCCAGGCATAAGGGAAGCCTCACTAGGAGGAAATATATCTGGACGCTGGCATCAG
GTCAGGGGGCAAAAGAGTCGCCCGGCCAGCATAAGTCCGAGGCCCAGCATTTGAGCTGGA
AGGCAGCTGGTTCTGTGAGCCGTTCTGTAAAGGAATGGGGAAAAACATTTCTCCCGATCC
TGAGTCTGGTGGGCAGTGGATGCCCTTCGGTAAGGTAACTTTGCGGAACAGGACAACAGC
AACCCAGGATAAACCTAGTTCAGTGTTTTCGGCTGATATTTGTTCGTCTACTGTCTGTCG
CTTGTCTTTGTGTGCCAGTGGCGTGCTTGCTTTTGTGCGTTTATTGTGAGTGTTGCCGTC
ATGGGTCAAAGTTATTCTACACCGCTATCTGTAATGACTGACCATTTTTCCGATTTCAAG
TCCAAAGCTCAGAATCTATCAATGCTAGTAAAGAAGAGCAAGCTAAAAACCCTATGCTCA
TCTGAGTGGCCGACATTTCAGGTCGGCTGGCCACCAGAGGGAACTTTCAGCCTGCCGGTC
ATCCAGGCTGTTAAAGAAAACATAACGGCTCCTGACCCTCTGGGCCATCTGGACCAGGCT
CCCTACATCCTGGTCTGGGAAAATCTAGTAGAAGACCCTCCCGTCTGGCTGAAACCCTTT
ATTCACAACCCCCCCCCAGTGCTTCTGTTCCACAGGCCCTGGTCCTGGAGGCCTCTAAGG
AAGAAGACCGGGAGGCACGGGAGAGCCGGAAGAAACCAATACTCCAGGAATCTTCTCTAT
ACCCTAGTCTGATTGATTTAGACACTGAAATCTCGCCTCCCCCATATATCCCGCCACCCT
TCCTCCCGAAGGTACCTCAGGTGTCGTCCGGAGAACAAAGGGGGAACTCAGAACCCTCGG
CTCCACAGCAGGAGGGGGGGCCAGCCCAGGAAACTCGCGGAAGGACCCGAGGGGGTGGAG
ACTCGTCTGACTACGGGAGCCTAGAGGCCCCTTCGTCCACTGTTCGAGCGCTTCCTGTCC
AGGTCGGGCGGCAAATCCAGACGGGGAACGGAATTATCAATATTGGCCTTTCTCTACCAG
TGACTTATATAACTGGAAAGCTCAAAACCCCTCTTTTTCTGAAAAAACCCAAGGCCTTAT
TGATCTTTTAGACACTATCCTGTTTACTCACAATCCTACTTGGGATGATTGTCAGCAGCT
GTTACAGGTGCTTTTTACCACGGAGGAATGGGAGCAAATTCTGTCAGAGGCACGGAAACA
TGTTCCTGGAGTGGATGGGAGATCAACCATGCAGCCTTACCTAGTGGAAGAAGAGTTCCC
TTCCATGCGGCCGAACTGAGACTTTGAACAGGCAGAAGGTAGGGAGCGTCTCCAAGCGTA

TCGCCAGACTCTCATGGCCAGCCTTAGGGCAGCCACCAGGAAACCCACGAATTTATCTAA
GGTGAATCTGATAAGACAGGAGCCCGGCAGCCTTCTTAGAGAGGTTAATGGAAGCCTTTA
GGCAGTATACGCCCATGGACCCCCAGGCTGATGTGTCGCGCGCAGCAGTTCTGTTAGCTT
TTGTGAACCAGGCAGCTCCAGATATCAGGAGAAAGTTACAGAAGATAGAAGGGCTGGGGG
AATTGTCAATACAGGATCTGGTGAGGGCAGCTGAGAAAGTGTTTAATAACAGAGAGACCC
CTGAGGAGAGGGAGGAACAGATCAGACGGGAGGAAAGGGAATATAGGGCTGAAGAAAACT
GGAGAAATCAGAAAGAGCTGGCTCAGATCCTTTTGGCGGGGACAAGAACGGGGCCTGAAG
CCCCGAAAACTAAAGACACCCGGTCGGGAGGAAAGGAAAAACCAGCTAGACCTGCTCTAA
AGAGAGACCAGCGCGTGTACTGCAAAGAGCAGGGACACTGGGAAAATGAGTGCCCCAAAA
GAGAGCTGAAGAGAAAGACTGTGAGAAAGGAGGAACCTTCCCGGGGACCCACGTCTTATA
TGCAGGGGAAGAAAAGAGACCTGAAGAGAAAGACTGTGAGAAAGGAGGAATCTTCCCAGG
GACCCACATCTTATATGCAGGGGAAGAAAAGAGATCTGAAGAGAAAGACTGTGAGAAAGG
AGGAACCTTCCCCAGGGACCCACGTCTTATATGCAGGGGAAGATAGTGATTAGGGGGGTC
AGCGCCTGGCACCTCTCCCCTAGCCCTGGGTAACTATTAATGTGGAGGGGAAACCGATTG
GCTTCATGGTGGATACGGGAGCCCAATACTCAGTTCTCAATCAAAAATTTGGGCCGATGT
CCAAAAAGACTAGCTTGGTCCAGGGAGGCACCAGGACAGAGATATTACTGGACTACTAAA
TGAAAAGTGAACTTGGGAGCCCAATGGGTGTCCCACTCATTTCTGGTGATCCCAGAATGT
CAGGCCCCTTTATTAGGAAGAGACCTATTGGCCAAAGTCAATGCGCAAATTCACTTTGAC
TCTGGGGGAATATCAGTCACAGACGGGCTTGGACAACCAATTCATGTTTTATCCCTGGCG
CTGAGAGATGAATACAGACTACATTCACCAAAGCCCCCTGCAGCTGTGGATCCTGCTATG
CAACAGTGGATTCAGAAATACCCTCTGGCCTGGGCAGAGATAGTGGGAGTAGGACTGGCT
AAACAAAGACTTCCCATTGTTGTTGAATTAAAAGCAAATGCTACTCCTATGAGGGTGAAA
CAGTATCCCATGAGCCAGGAGCCTCGGCAAGGAGTAATGCCACACATCCAGCGCCTCCTA
AAGGCAAGAATTCTCAAAAAGTGCTGGTCCCCATGGAACACTCCCCTGTTGCCTGTGAAA
AAGCCCGGGGGAACAGACTTTAGACCAGTCCAAGATCTTCGTGAAGTCAACAAACGGGTG
AGTGACATTCATCCCACTGTCCCTAACCCCTACACCCTCCTGAGCAGCTTGCCACCAGAC
TATGTCTGGTATACAATTTTAGACTTGAAAGATGCCTTTTTCAGCTTGCCTTTGGCCCCC
CAGAGCCAGGAAATGTTCGCATTTGAATGGGCTGACGAGGACGGCCAAACTGTGGGCAGC
TGACCTGGACTCGCCTCCCACAGGGGTTCAAAAACTCGCCGACGTATTTCAATGAGGCTC
TGGGTGAAGATCCCTGTGAGTACCGAACCAGCCACCCCGAAGTCGTTCTGTTGCAGTATG
TAGATGACCTCATGCTGGCCGCTACTACTAAAGAGGTATGCCTAAAGGCCACAGGCGATC
TCCTCCAGACTCTGGGGACATTGGGGTACCGGGCAAGTACAAAGAAGGCCCAAATTGCTA
GACAGGAAGTCATTTATTTGGGATATAAAATAAAACAGGGGCAGAGATGGTTGACTCAGG
CTATGAAAGAGACTATTCTACGGATCCCCGAGCCAACGACTCCCCGGCAGGTGAGGGAGT
TTTTAGGGATGGTTGGGTACTGCAGGCTATGGATCATGGGGTTTGCTGAAAAGGCCCGAC
CTATATATGAAGGAAGCAGAGAAAATAGAGACTGGACTTGGACTGAGCCAATGAGGCGGG
CATTTCAGGAACTTCGACAGGCGTTACTGGAAGCGCCAGCCCTTGCTCTTCCGGACCCGG
CTAAGACGTTTCAACTGTTTGTGGATGAAAAGCAGGGAGTCGGGAAGGGAGCCCTGACGC
AGCAATGGGGACCGTGGAGACGGCCTGTGGCATATCTCTCTAAACGACTGGACCCAGTGG
CTGCGGGATGGCCACCCTGTCTCTGCATCATTGTGGCTACTGCTCTCCTTGTCCATGATG
CTGACAAGCTGACTTATGGACAGAGCCTCCTGGTCTACACTCCTCATGCAATAGAGGGGA
TCCTCAAACAGCCACCGGGTAAGTGGATTTCTAATGTTTGCTTAACCCGCTACCAGGCCC
TGCTGCTGGATGCTCCCCGAATATCCTTTCAGACACCTTGTTTTCTAAACCCTGCCACTT
TGCTGCCGATCCTGGAGGAGGACGGGCCCCTCCATGACTGTGTTGAAGTGTTAGCTGAGG
TAACCGCCATACGAAAAGACCTCAGCAACTTGCCATTAAAACACAGTGAGCTGATATGGT

TCACGGACAGGAGCAGTTATATAAAGGATGGACAGAGAAAAGCGGGGGCAGCCATAGTAG
ATGACACTGGGAGGGTCATCCGGGCTGAGGCCTTGTCCCCCTGGAACATCCACCCAAAAA
GCAGAACTGATAGCTGTGATACAAGCACTAGAGAGGGCAAAAGGAAAAAGAGTCACTATT
TACACTGAGAGCCGATATGCATACGGCACTGTGCACATTCAAGGCCCTATATATAAAGAG
CGGGGGCTTCTGATGGCAGAGGGGAAAGAGATTAAAAATTTGCCTGAGATTCGCAGACTC
CTGGCAGCAGTCCACTTGCCCCGGGCGGTATCCATAGTACATGTCCCGGGACACCAGAAA
GGAGAGGATGCCTGGGCCCAAGGGAACCATGCTGCCGATACAGCAGCCTGTGAGGCAGCT
GCTGGAGACTACAGGACCCGTGTACTGACTGTGGGATTGCCACCGCCAGGGATGGGAACA
CTTCCCCCCAGCCCCATATACTCCCCCTCTGATTTACGCTGGGTGCAAGATAATACCACC
CATCCTGTTGGCAAAAACGGATGGTATCGGGACCAAGATGACAACCTGTTGCTCCCTGCT
GACCTGGGCAAACATCTCAGTACCCATTTACATCAAACCACCTATTTGGGAGAGAAAAAG
ACTTTGACACTCCTACAAACGGCACAGCTGCGGTTTCCCCAACAGAAGAAAACCATCCAG
GATATAGTCCACACCTGCAAGGCCTGTCAGATGATGAGATCGGGAAAAGGACAGCATGCA
GGTGTAAGATATCGGGGGGAAAGGCCAGGACATCATTGGGAGATAGATTTCACTGAGGTA
AGACCAGGCATGTATGGGTATCATTACCTGCTAGTTTTGGTTGATATGTTTTCCGGGTGG
GTAGAAGCTTACCCCACTAAAAAGGAGACAGTGGTAGAGGTGGCTAAGAGACTCCTAGAA
GAGATTATACCTAGGTTTGGGCTGCCGGTATCTATCGGCTCTGACAATAGACCTGCATTT
GTGAGTAAAACTGAGCAGGGACTGGCCTCAGCCCTGGGGACCAAATGGAAGTTACATTGC
GAGTACAGTCCCCAGAGCTCAGGACAGGTAGAAAGAGTGAATCGGACCCTAAAAGAAACT
TTAACAAAACTGGCAATTGAGACTGGCAGGGACTGGGTGACCCTCCTTCCCTTTGTGCTT
TTTCGGGCGTGCAATATCCCCTATAAACTAGGTCTGACTCCCTTTGAAATTGTATATGGA
AGTCCACCTCCCATTTGTCCGGTGTCTGAAGGAAAGAGTAAACCACCTCTTTCGTTACGT
GCCTTCCAACAGGAGATGCTAGCTTTGAGTAAGGTGCATAAACATATCTGGTCACTGATA
CGGAAAAATTTATGAGAGCCAGAATGAAGGGACGATCCCGTCCCATGATATAGGACTGGGG
AATTGGGTTTGGGTCAAGAGACATAAGTCAAAGACCTTGGAACCAAGATGGAAGGGCCCT
TATGTTGTTCTCCTTACCACCCCTACTGCCCTGAAGGTGGACGGAATTGGACCCTGGATA
CACTGTAGCCACGCATGGTGGGCCACCCCCGAAGAACAAGAGAGGGCCCAAAGAGAATGG
AAGCCGATACTGCATCCCTCCAATCCCTTAAAACTGAAACTCGCCCAGCAGCTGGTCTCG
GACGGATCAACCTGACTCTTCTGATAATAGCCACCTTCCTGGACCCTGGGACCGTCAGCG
CTAACCCTCATCAACCAATGAACCTGACTTGGATGATTCTGAGCACAACTACGGGAGAAG
TAATTAACTCCACCTCGGCCATTCATCCCCAGAACACCTGGTGGCCGGACTTGGAGTTCG
ACCTCTGTTTTTTGGCCATGGGATCATGGGACATCGGCGACTGGGAGGTAAAGACGCCCG
GTAAGCCTGAATGTGGGGCTGGCATTAATAGGTGTAATACTCGACCCCCCACTAACTCAG
GGCCTGGGTGCAGCCACTTCATCCAATGAGCAAGCTTGCGGAAAACACCCTTCTATGTGT
GCCCCGGAGGAAGGCGGGACCAGGCGACCGTCAATAAATGCGGGGGTGCTAGTGAGTTTT
ATTGTGCCAATTGGGGATGCGAGTCAATGGGACAGTCCATTGGGAGCCCCCTATTCGTGG
GGACTTGATTACCTTGGGTCACATTCCGGGGCCTGCTGGGCTAACTGGGGGACATAGAAA
GGGGTCCCTCATAACTGGGCCTTGTATGAATTTTCTTTGCAACCCGGTAAGGCTCAAATT
CTTAACTGAAGGGAAGAGGTTCCCCAGTTGGGAAGCTGGACAATCCTGGGGCCTTCAGTT
ATATCAGTCAGGATATAACAGTGGATTGCTATTCACTGTCAGGTTAAAAGTAGGACCCAT
ACAGATCGGTCCAACTCGGGGCATAGGGCCCAATTCAGTCCTAGTGCCACGAGAAGCTAC
CCACGCTCCCATTCGGCCTGCACACACCCCCGCCCTAGCACCCACCCAAACGCCTCCCGG
GCCCTACATTACTAATACAAGCAACTCCCCTCCAGTTAGCCGTGCGGGACCTAGCATTAA
AGATGAGCAGGACCCATTATTTAATATGATAATCAACTCCTATCAGGTTCTAAATTCCAC
ACGCCCGGATCTGACTAGCAGTTGCTGGTTATGTTATGACATCAAACCCCCTTATTATGA

AGGTATAGCTGTCCCAGGAAGTTACAGTCCAACCCAAAACCACGCAGCTTGCCGATGGCA
GCAGAAAGGAAACGACAGATTAACCCTCCAACAAGTAACCGGGCGGGGCCTTTGCATAGA
AAATGTCCCCCAAACCTATCAACACCTCTGCAAGTCTATAAATTCTACAGTGACAAACAG
GTATCTGGAGCCTCCCCAGGACAACTGGTGGGCCTGCTCCACAGGTTTAGCCCCTTGTTT
TCATGGGTGGGTGTTAAATAATTCCAAAGATTTCTGTGTATTGGTACTATTAGTCCCCCG
ACTGTTCTATCACTCCAATAATAAAAATCCTCTCAAAACTAGAAGCTTCACACCGATCTA
AGAGAGAGCCAGTCTCAGCCTTAACCCTCACAGTCCTACTGGGGTCAGGGGCAGCCGGGG
CCGGAACTAGGATATCCTTTTTAATAATGCAGAATCAACACTATTCTAGCCTAAGAGCAA
CCATTGATCCTGATATTGAAAGATTAGAAAGCTCAATCAGCCACCTTGAGAAGTCCCTTA
CTTCACTATCAGAAGTGGTTTTGCAGAACAGGAGGGGCCTAGATTTGATCTTCCTGCAAC
AAGGCGGATTATGTGCGGCCTTAGGAGAGGAATGTTGCTTCTATGCAGACCACACTGGGG
TGGTGAGAGAGTCTATGGCAAAAGTGAGAGAAGGATTAGCACTGCCCAAGAGAGAGCGAG
AAGCCCAGCAGGGATGGCTCGAGTCCTGGTTCCATCAATCTCCTTGGCTAACGACACTAA
TTTCTACCTTGCTCGGACTGCTCATTATACTCCTACTCATTCTTACCTTCGGCCCACGTA
TTTCAAACAGATTAATAACCTTTGCAAGAGAACGTGTCAGTACAGTTCAGGTAATGGTAT
TAAGACAACATTACAGGCAGTGAATGGAGAGGAGGATTCCTCTCCATGATCAAAGGACAA
GGGGGAAATGTTAAGGCCTGATAGAGGCCATGACTAAAATAGGCTGGAGCAAGCATCCCG
GAAACAGCTGAGACACTGCCTGGGCAGAATCAAAATTAATTACCCATGACGGGAGGACTC
AGCCTGTCAGCATGCTCCCCGTAGCCTGGTCTATGGCAGTCGGGACGAGGATAGATATCT
CCTAAGAAAGCCCGCGCGCGTGAAAATCTAGCCAATTAGTAGATGCAAAGAAACATTTGT
AACCAATCCAACCTTGCCAATTCCCTGTCTTTGTCCTAAACCTATAAATACTACTGTAAT
CCAGGGCTCGGGGCTCTGGCTCTATTCCACTGTGTTGGATGCAGCCAGGGCTCTGGCTCG
AGCTAGCAATAAATTCCCTTTTTTGCGTTTGCATTGCCGTGGATGTCTTGTTCTCTGAGT
TCTGGGGATCCGGACCTTGGGCATAACA
>CAP_ERV_3
CCCCGGAAAAGTGCCGCAGACAAGTCCCGGAGGCGGGCCGACAACCAAGCAGTCCAATCA
GGTGCCGACACAGAGCCCTTGGACACCAACCACCGCTGTAGCCCGAGCTTTCTACCTTAT
ATGGGAGCCTGGCCCTGGCTATAAAACCTTTCCCCACCCCTCATACCTCGCAGACTCCCT
TTGCTTCCTTGCTCACCCACCCCCGGGAGTTCTGCCCGAGAGCGACCGCCCAATAAAGGC
TCTGATCAACGGTCCATAGAGGTGGCTCTTTCTTCCCGCGGCGTTTCTTACATCTGGCAC
CCAAAGTGGGGCTCAAGGTGAGGGCCCCCGGCACTAGCTGTTGAGGCCCCCTCGAGCTCC
ACCGCTGCAGTAGCCCCGGACCCAGGCGGCTGACCAACCCCCCGGACAGCAGGATACAGG
GTAAGTCCCTTCGGCTTTGGGGCCCGCCCTCCCTGGCGACCACCTCCTAGGGCCCGGTAA
CGGGTGCGCACTTACTGGGCCCTCCCGGTCACCAGCTTGTAGGGGAGACGTCCCCAGTGG
CTGAGTAGACCTCCGGCTTCGGCCCTTCCTGGTCCCCAGCTCGTGAGGAAGACGTCCCGA
ACAGCTGCACGGACCTCCGGCTTCCCTTGGCACGTCCGAAGATGTTCGGGCAGCGGGGGT
TGCCTCCACGCTCTGTGGTCGCCATGGGATCGACGGCCTCCAAACCCAATCCCCAATACT
CCACCCCCTTAGAGTGCCTGCTGGCTAACCTGCGGACCCTAAGACTAAAGGGATATATCC
GCCCCAAGCAACTCACTTTCTGTGTTCACAAGCCTGGCCTCAGTATCCCCTAGATAATGG
CTCACAGTGGCCAACCACAGGGACAATGGACTTTGACGTCCTCCATGATTTAGATAATTA
CTGCCGAAGAATGGGAAAATGGTCTGAGGTCCCCTATGTTCAGGCCTTTTGGGCGTTGCG
CTCTCGCCCCACCCTGTGCACCACGTGCGCTCCCAGCCAAATCCTACTTATCATGGCTCC
CCCGATTCCACCCTCCCGGGCCAAACCAGCTCCTCCCGTCTCTGAGTCCTCTGCTTTCTC
TGTTCCCCCGGAAGATCTGGTGGCCCCTCCTCCCTATACCTCTCCCATGGTTCCCACCCC
TTCCACTCTGGTTCCTGCCCCCTCCCCCTCCACTCCGGCCCCTCCTACCTCCGCTTCCGC

CCCCGAGACTCCACCACTGGCCCCGGATCCTCCAGCCCTTAACCCCATCCTGTATCCTCC
TCTTCCCCCTGTCACTCCCTCCCCTTCTCCGGTTAGCTCCCACACTCGCTCCCACAGCAA
CCCTCCGGGAGCCTCCCCTCCCCCTCCCCCAGCCCCGCTACTCCCTCTCCGACAAGTAGC
CGGAGCTGAAGGTCTAGCCCAAGTCCACGTCCCCTTCTCCCTCCAAGACTTAGCACAGAT
TGAGGCCAAACTGGGTTCCTTCTCCTCCAACCCCACTCAGTACATTAAGCAGTTTACTGG
TCTGACCCGCGCCTACGCCTTGACATGGCAGGACATATATGTCATCCTGGGGTCTACCAC
CACCCCCGAAGAGAGGCAGGCCATCTGGACGGCAGCCAAGGCTCAGGCCGACCAGCAGCA
CTTTGCCAACCCCTCCCCTGAGTGCCCCCCGGGGGCCCAGGCGGTTCCTGACACTGACCC
TGATTGGAACTACCAGGAAGGGGGTGGCGGCCAGCTGCGAGTACGCTATATGATAAAGTG
TATCCTCAATGGGATGGAAACGTCCTCTCATAAGGTTGTAAACCTCCTCAAACTAGATGA
GGTGACCCAGGGGCCCGACGAAAACCCAGCCATATTCCTTAATCGGCTGACCGAGGCCCT
CGTCCAATACACCAGGCTGTCCCGAGTCCCCCATTGGGGCGGCCACCTTGGCCAATCGTT
TTATCTCCCAGTCTGCCCCCGATATCCGAAAAAAGTTGGCCAAGGCAGAGGACGGCCCTC
AGACCCCTATCCGAGACCTGGTAAAAATGGCTCTTAAGGTCTACAATGCCCGCGAAGAAA
CTGCTGAGGCCAGCCGAAAGGCAAGGCTCAAGCAAAAGGCCGAATTTCAGGCAAGCCTCC
TCAACTAGCAAACCCAGGCCTTGGCAGCGGCCCTGCGGCCGGCGGCGGGCTCGGGGCCCC
AAAACCCCCCTCCGGGGGCCTGCTTCAAGTGCGGCCAAGAAGGGCACTGGGCCAAGATGT
GCCCCAATCCGCGGCCTCCTTCCAAGCCGTGCCCGTTGTGCAAACAACGAGGACACTGGG
CTAGTGACTGTCCCCAGGCCTCTCGGGCCTCGACCTCTAGGGGCCAGGGACCAGAGTGCC
CCAGGGAGACCTCCTGCCCTCCTCCGGCTTTGGAGCTGCTGAGCTTCGACGGTGACTGAC
ACCGCCCAGACTCGGGGACCCCAATAACCCAAGCCTAGCCCAGGGTAACACTCCAGGTAG
CGGGTAAGTCCATCAATTTTTTGGTTGATACGGGGGCTACCTATTCAGTCCTTCCCTCTT
TCGGGGGCACTCTACGTCTTTCCCAGGTTTCGGTTATGGGCATTGACAGCCAACCCTCGT
GTCCGCTCCAAACCCAACCATTATCCTGTCAATTAGATTCCTGTCTATTTACCCACTCCT
TTTTGGTCATCCCCTCCTGCCCTACTCCTCTCTTGAGAAGAGATATACTTGCTAAGCTGA
AGGCTACTCTTCAGCTAGCTCCAGGATCAGCTCCCACGTCAGGGGCCTTCCTAATGCTAC
TTGTTGACCCTCCAACCTCTTCTGTTAATCCTGAGGTCTGGGACACCCGAGTCCCGGTGG
TGGCTCAGCACCACCCTCCAGTCCTCATCCGGCTAAGGGACCCCACCTGTATCCCAGCCC
GGTCCCAGTTTCCTTTGTCTACTCGCAACCTCAGGGGGCTAAAGCCCATCATCGACCGTC
TCATGGGACAGGGTCTCCTGATCCCCAAGACCTCCCCCTGTAACACGCCCATCCTCCCTG
TTCAAAAGGCTTCAGGAGACTACCGGCTAGTGCAGGATCTCCGCCTGATCAACGCGGCGG
TGATCCCTGCCCATCCACTTGTCCCTAACCCCTACACCCTCCTTTCTTCCATACCTCCTC
AGACCTCTCATTTCACCGTTACTGACCTCAAGGATGCCTTTTTTACCATCCCACTCCACC
CGGACTGCCAATTCCTCTTTGCTTTCACCTGGACTGACCCCGACACCCAGCTGACCACAC
AACTCACATGGACCGTACTCCCTCAGGGGTTCAGAGACAGTCCCCACTACTTCGGGCAGG
CTCTGTCCCGGGACCTGGCCAGATGCTCGCTTTGCCCTAGTACCCTCCTCCAATACGTAG
ACGACTTGCTCCTTTGCAGCCCCTCAGAAGAGACCTCCCGACAACATACTACAACTCTCC
TCAATTTCCTTGGTTCCCAGGGTTACAGAGCCTCACAATCCAAAGCCCAACTGACTCAGA
CTTCTGTCGTCTATCTGGGCCTCCAAATCACTCCGACCACTAAAGCCCTGACAGCAGAGC
GGTGTAGCCTCCTCCGGTCCATCTGCCCTCCGGCCGATGGAGACCAGATATTGTCCTTCT
TAGGACTGACGGGATTCTTCTGACACTGGGTCCCAAATTATGCTACCCTGGCCAAACCTC
TATATGCTGCAGTTAAAGAGACTCCCACAGGGCCGCTGTCTTCCCCCACCGAAGTGACTC
AGGCCTTCCACGCTTTACGCTCAACCCTATTGGCTGCACCCCCTCTCTTTCTCCCAAATC
CCAACTATCCACACCATCTATACACTGATGAAAAGGGAGGGATAGCCTTTGGAGCCTTGG
TGCAACCGATTGGCCCCGAATTGCTGCCTATTGCTTACATATCCAAACAACTTGACCCCA

CAGCCAGGGGATGGCTCCCCCGCCTGCGGGCACTAGCGGCGGCAACAACGTTGTACGCCG
ATGCAAAAAAGCTGATTCATGGCCAACCCCTGACCGTCTTCTCGCCCCACCGTCTTGGTG
ACCTTTTAGCCTCTAGATTTCTTTCTGAACTCAGCAAGTCCAGGCTCCAACAATTTCACC
TGGTATTCCTGGACAATCCTCAGGTTTCTGTGGGCTGCTCCCCACAATTAAACCTGCTTT
CCTCGCTACCCTCACTCCCTGTCTCCTCAGAACCCCCAGCCCACTCATGCTTGGAGGTCC
TTGAGTCCCTTATGCAACCACCCCACAACCTGTTCTCCAAACCTCTACCAAATCCAGAGA
TAACTTTGTTCATCGATGGGAGCTCAAAGCGAGACCCCAATGGAAACCGAAGGGAGGCTT
ATGCTGTGGTAACCACCCGGGAAGTCCTAGAAGCTCAGCCCTTGCCACCCGGGACGACTT
CTCAGAAGGCTGAACTAACAGCCCTAACTAGAGCCTTACACCTAGCAGAAGGAAAGAGGG
CCAATATTTACACAGACTCCAAGTATGCCTTCTTGATTGCCCATTCTCACGCGGCAATCT
GGAAAGAACGGGGCTTCCTGACCACTAAAGGATCCCCCATCTGTAATGCCCCCCACATTA
CTCGACTGTTGGATGCCTTATCCCTGCCCAAAGAGGTCGCTATCATACACTGCAAGGGCC
ACCAAAATACCCATGACACTGTCACTCTGGGTAATAATATGGCAGATCAAGTGGCTCAAC
AAATTACCTTACAACAAGCTCCAGAGCCCCTGCTGACTTTGAGATCCACCCTCAACCCAG
ATTATTCAAGCAAGGAAGCCAGAGCCTTGCTGGCGCAAGAAGGGGCTCAGAGGCACCCGT
CAGGATGGATACTACTCCATAATAAACCGGTGCTTCCTGAGTGCCAGACTAAGGCCATAA
TATCCCAAATCCACAATACCCTCCACATCGGACCAAGGGCTCTTTTTTCCTTTCTCAGCC
CTGTCTTTTCTCCCCTACATCTCAGACAGACCATATATGAGGTCCACCGCTCCTGCCTAA
CATGCACCTCCACCTCTCCTCAAGGAGAACTCCAACCCCCACAGGAGACTCACCAGTTGA
GGGGACATATGCCCGGGCAGGATTGGCAGATAGACTTCACCTACATGCCCAGACATCGAA
CCTACCGCTGTCTGCTGGTCTTGGTGGACACCTTTTCAGGATGGGTCGAGGCCTTCCCCA
CCGCCCGGGAGATGGCAGCGGCGGTGGCGGATGTCTTGACAGCCCATCTCATTCCCAGAT
TTGGATTGCCAAACTCTCTCCAGTCTGACAATGGGCCTGCATTTATCTCACAGATCTCTC
AGCAGGTGGCTGTGGCCCTGGGTATCGACTGGCATCTCCACATCCCCTATCGGCCCCAAT
CGTCAGGCAAGGTAGAGCGGGTGAACGGCATCATCAAGACACATCTGACCAAGCTTGCCT
CAGAACTGCGGCTGTCTTGGGTTGACCTCCTTCCTCTGGCGCTCACTCGCATTCGCACCA
CACCACACTCCAAAACAGGTTTGACCCCTTTTGAACAGCTTGACGGCAGGCCCTATCTCC
TGACTCACCTCCCAGAGGGAGAGGCTCCCCCACTCGCGGGGTACCTCCCCCTCTTCTCCC
TCCTATGATCCTTGCTGAGGGAACACGCAGACCGGGTCCGCCACAACCGACAGACGACGA
GAGGCCCACCCAGCCTCTGGCCCCAGGAGATCAGGTACTGCTGAAAACCCTGAGTCCCCG
CCCACTACAGCCTCGCTGGACGGGACCCCATACTGTAATCCTCACCACTCCTACAGCAGC
CAAACTCCTGGGACACGAGCGCTGGTACCACCTGACCCAACTCAAACGAGCTCCCCCCGG
TCTCCCAGAGACAGGGGTGGCCCCGGCCCCGACCCCTTCTCCCAATCACTCTTACTGCTC
CACCCTCACGGGGCCGACCAAACTGACCATCACCCGGACCCCCCTGTCGTCGATTCCTGA
ATGACTGAGAGACCACAGTCCAATTCGATCTGATGCTGGCCTGGCATCACCCGCTGTGGC
TGCTGACAATTACAGCTCTGACCATACTCTTTGCCATTGGATTAGCGGCCACGGCCCCCC
GCGATTGGTCCCTCACTCTTCGACTGTCCCTGGCCCTCACATACCTAGCCACCTGCTTCC
TACTCCTGGGGTGCTATTCCCTTGGGACACCCTGACCTGGTTACCAGCTCCATCCTTACG
GGACCCCCGAGGCTGGTTGTCCCCCTTTCACCCCAACCTCGGAACGATGCGGGGCTCTCT
ACTCTGGGTTACTCTGGGGGTCCTGGAAAAAGGGGGACACACCAACCAAAACCCCCACCA
GTCCTTTCAGATCACTTGGACGCTCCGGAATGGACTGACTCGCGAGGTACTCAATTTGAC
CACCGGAATTCACCCCCCTAATACGTGGTGGCCAGATCTGTATTTCAACCTCAAAGACCT
TATAAAGACTACTTGGACAGCGGCCCAGACCAGAAACTTTGGATTCTGGGCATGTCCCGG
ACACCTAAAGACACATAATTGGGAGACTTGTGGGGGGCTGCAACATTATTTCTGTTGGTC
CTGGAGCTGTGTGACCTCCAATGATGGGAGATGGAAATGGGAGGTCGGGAACCTGGACCT

AGTCAATTTCTCGTTTGTCCAGCCCTATCACGGGCCCTGGGAGCAAGATTACCAAAATCT
GTTCGGAGATTATGTCAGGCTGAGTTGGAAGCAGCATTGGAGCCCAACTGCTCAGGTGAA
AGTAATGTTCGACCAGAAAGCGGCTAAACTGGAGAGGTCTTGGGTATCCGGGCTATCATG
GGGCCTCCAACTCTACGCTGAGTGGTTTGAAGCAAAGCCAGGGGGAATTCTGGTTGTTAG
TCAGACAATAGAACCTGTTCAGGCCCATGCTGTGGGGCCCAACCAGGTTATTGCACCTCC
CCGCCCCTTACCACGGGGGACAAACGCCACAAATGTCGTGACCTCGCCTGCCCTCACCCC
CTCCCTTCAGCTGACCCGGACTGACCCACCAGAGACCGAGGAACCCCTGTGGGCCTTGGT
CAAAGAAACCTATGGGGCCCTCAACCACTCCAATCCTAACGCGACCCAATCCTGCTGACT
CTGCTACACCCTACATCCACCTTACTACAAGGCCTCGGGCTTAAATGCTACCTACAACTT
ATCCATTCTCTCCAACCCACCGCAGTGCTCTTGGGGAGACCGCAAGGTGGGCCTTACCAT
GAAACAAGTATGGGGTTCGGGACCTGCTTAGGCACGGTCCCTACAGATAAACAAACCTTG
TGCACCCAGACTGGCGATGATACCAATTTCACCAATAAGACCTACGTCATACCCGAAACC
GGGGGGTGGTGGGTGTGCTCACAGACTGGGCTAACGCCCTGTCTCCACTTGGCTGTCTTC
AACCAGAGCAGGGAATTCTGTGTCATGGTAGTGGTGGTGCCCAAGATTACATACCATCCA
GAAGAGGTCCTCTACAACTTTTGGGACTGAGACACCCCAGCTCCTAGACATAAGAGGGAG
CCCGTTACAGCTATTACTCTGGCAACACTGTTCGCCCTGGGAGCAGCTGGGATGGGCACA
GGCATTGCTTCCCTGACCACACAACATCAGGGCCTGATCACCCTGAGGGCCGCAACTGAC
GAAGACATCGCCCGCATAGAGAAGTCCATGATGGCCCTAGAAATGTCCCTGACTTCCCTT
TCCGAAGTGGTATTACAAAACCGCAGAGGACTAGACCTGGTCTTTTTGCAGCAGGGGGGC
CTCTGTGCAGCCCTCAAAGAAGAATGCTGTTTCTACGCTGATCATACTGGGGTGGTTCGA
GAGTCCATGGCTAAAATAAGAGAGGGATTAGAACGCAGGAAGAGAGAGAGGGAGGCCTAG
CAGGGCTGGTTTGAGTCCTGGTTCCAGAGCTCCCCTTGGCTGACAAGTTTGCTATCCTCC
CTAACGGGACCACTCATCATTCTCCTGCTGCTCCTGACCTTTGGGCCCTGTATCCTAAAT
AAGCTTTTGGCCTTTATCAAACAACGGCTCAACATGGTCCAGCTGATGGTGCTGCGAAAG
CAGTACCAAGGGCTTCCAACAAACACTCTGTGACAAAATTAACGGCAGACACCCCGTCAT
CCCAGCCACACCCTACCCCTCGCCACCCCCGTTCAGCAGGAAGTAGCCAGAGAGGACTGG
CGCCCCTTGTCCTATATCAAAAAGGCCGGGATGAAAGGTCGGGCATGTTGAGGCATGCCC
GGGCGTGTTAAGGCATGCCCCCGGAAAAGTGCCGCAGACAAGTCCCAGAGGCGGGCCGAC
AACCAAGCAGTCCAATCAGGTGCCGACACGGAGCCCTTGGACACCAACCACCGCTGTAGC
CCGCGCTTTCTACCTTATATGGGAGCCCGGCCCTGGCTATAAAACCTTTCCCCACCCCTC
ATACCTCGCAGACTCCCTTTGCTTCCTTGCTCACCCGCCCCCGGGAGTTCTGCCCGAGAG
CGACCGCCCAATAAAGGCTCTGATCAACGGTCCATAGAGGTGGCTCTTTCTTCCCGCAGC
GTTTCTCACA
>CAP_ERV_4
TGTTAGGGACCAAACAACGGTCTCTAGGACCTGAGTCATGTTTACCAGAAAGAGACAGGA
TATGCGCTCATCCTGCCTGGCCAATCATGTAACGCCAGCTACTCCTGTAACGGAGACAAA
GAACTGCCTGTATATAAGCTGCCATACTCCTTTGTTCACGGCTCTTGTCAGATTCCCTTG
TGTGGGATGAGACTTGGGCCCTAGCGCGCTAGGAATAAACAAACTCCCTTCTTGCGTTTG
CAATACTGTGGTGGACTTGCTCTCTCGGTCGGTTTGGAGATACGGGCTCGGAGCATAACA
TCTGGGGGCTTGTCCGGGATCTCCGTCCCGCCAGGGAAGACAACTCTCCTGGTAGAGGGA
GTAACCTCGTTAGGAGATAAGAGCTCTGAGCACCGGTGCTAATGTTGCAGAAACCCAGAT
TAAGTCCGCGGCCTGGTATCGTACTAGGGAGGCATCTGGCTGTAAAGTGCAGATGCAAGT
CAGCGTAAGGCCGGGGCCTGGTTTCGTGCTGGGTAGGCAGCTGGCTCTGGTTACTGGATT
AAGTCAGCATAAGGCTGGGGCCTGGTTTCGTGCTGGGGAGGCAGCTGGCTCTGTGAACAT
CCTGCAGGTAAGATTGAGTGCATTGTCGGTGGCCACCTTGTGTTTGTTATCTGTTTGTCT

ATTTGTGGTGTCTGCGCTGCTCTTTGTGTGCTCTGTTGGCTCCCAGTGTACTTTTCTGTG
ATCATGGGACAAACAACTTCTACTCCTTTATCTCTTATGATTAACCACTTCTCTGATTTC
AAGTCTAGAGCTCAGAATCTTTCGTTATTGGTAAAGAAGAGCAAACTGGTGAGTTTCTGT
TCTGTTGAGTGGCCCACCTTTGACGTCGGATGGCCGCAAGAGGGAACCTTCAATCCCCAA
ATTATCCAGGCAGTTAAAGAGAGGGTGCTTACTCCTAGTCCTGCCGGGCACCCAGATCAG
ATGCCCTACATTCTGGTCTGGCAGGATCTAGTGAGGAACCCGCTGGAATGGCTTAAACCC
TTTGTTCTCGCTCCTCCTAAACCTCCCCGTCCCTCTTCCCCAACTCCAACCTCGCCAAGC
CCACAGGTGCTAGTAATGAAGGCTTCCAAAGAAAAAGAAGAAAAAAAAGGATGAAAATCGA
CCAAAGCTGGTATTCCAGGAATCTTCTCTGTATCCTAATCTGATAGATATGGAGACCGAA
TTGTTCCCACCCCCGTATGCAGATCCACATCTGCCCTTGCTTCCACAGGTTTCATTGGGA
GAAGCCAGGAGGAGAACCGAGCCTTCAGCTCATCCCAGAGAAGGGGGCCCCGCCCAGGGA
ACTCAGGGAAAAAACAAGAGAAATGGCCAACGTAGCGGAAGAAGACCCAGAAGTCCCCTC
CTCCACTGTTCACGCATTTCCGGTCCAGGCGGGACCAGCCAGAGAGGGTGGAGAGCGGAC
ATATCAGTATTGGCCCTTTTCCACTAGTGATTTGTACAACTGGAAAACCCAGACTCCCTC
CTTCTCTGAAAAACCACAGGGTCTTATTGATCTTTTAGAGTCTATCCTTTTTACTCATAA
TCCCACTTGGGATGATTGTCAGCAACTGTTACAGGTGCTTTTCACTACAGAAGAGCACGA
GCGGATCCTGTCAGAAGCTCGAAAGAGTGTGCCAGGGTTGTATGGGAGGCCCACAATACA
GCCTAACCTCATTGAGGAGGGATTCCCCTTAGTGCGACCCAACTGGGACTTTGAATGCGC
TGAAGGTAGGGAGCGTCTCCGGGTATACCGTCAGACTCTTATGGCCGGCCTTAGAGCTGC
CGCCAGAAAGCCAACTAATTTGGCCAAAATAAATTCAGTGAGGCAGGAACCAAATGAGAG
CCCAGCAGCCTTCCTTGAAAGGATAATGGAAGCTTTTAGACAGTATACCCCTATGGACCC
ACAGGCAGATGAGTCACGAGTGGCAGTTATGTTAGCATTTGTAAATCAAGCAGCCCCTGA
TATTAGAAAATTACAAAAGATAGAGAGGTTAAATGAACAATCCTTGCAAGATCTAGTGAG
GGCAGCCGAGAGAGTTTTTAATCATAGAGAGACCCCAGAAGAGAGAGAGGACCGCATTAG
AAGAAAAGAAAGAGAATTTAGAGCTGAAGAAAACCGTAAAAATCAAAAAGAGCCAGCCCA
GATATTTTTTGCTGGGGTTGAAAACAAAAACAGGTTCCAGAAAGGAAAAAAATTGGACTC
AAAAACTGAAGAAAAAATGACAAGGCGTAAGCTTGAGAAAAACCAATGTGCGTTTTGCAA
AGAGTTTGGACATTGGAAAGATAAATGCCCCAAGAAAAATCTAAAAGAGGGGCCCAAGAA
CCCCAAGAACGAGACTCCCTCTCCAGACAGTCATATCCTCTACGCGGGTGAGGATAGCGA
CTAGGGGGGTCTGGGCTCAAAGCCCCTCCCCGAGTCCTGGGTAACTATAAATCTGGAGGG
GAAACCGGTTGGCTTCATGGTGGACACGGGAGCCCAATACTCAGTCTTAAACCAAAAAGA
TGGACCCATGTCTAAGAAAAGTAGCTGGGTGCAGGGAGCAACCGGGACTAAACGATATAG
ATGGACTACAAAACGGCATGTGAACTTGGGGGCCCACCAGGTAACCCATTCTTTTCTGGT
GATACCTGAGTGTCCAGCGCCCTTGTTGGGAAGGGATTTACTGTCTAAAGTAAATGCCCA
AATTCATTTCGACCACGGACAAGTGTCAGTTTTAGATGGGACCGGGCATCCTCTTCAGGT
CTTGTCTCTGGCATTAAAAGATGAATACAGACTCTACTTGCCAGAGGCCCCAGCGACAAT
AAGCCCCGAAGTACAACCATGGGTTCAAAGATACCCTCAGGCCTGGGCTGAAACTGCAGG
AATGGGACTAGCCAAACAGAGGCCCCCTATCATTGTGGAACTGAAAGCCAGTGCTTCCCC
GGTGAGGGTACGACAGTATCCCATGAGTCAAGAGGCTCGACAAGGAATTACTCCGCATAT
ACAACACCTCATAGATGCTGGGGTCCTAAAAAGATGCCGGTCCCCATGGAACACTCCCCT
GTTGCCTGTGAAAAAGCCTGGGGGAACTGATTTTAGACCAGTTCAAGATCTACGAGAAGT
CAACAAATGGGTGAATGATATTCATCCTATGGTTCCTAACCCTTATACATCGCTAAGCAA
CTTGCCTCCAAACTACATTTGGTACACTGTTTTAGATTTAAAAGATGCCTTTTTCAGTTT
GCCTCTTGCCCCCGCAAGCCAAGAGATCTTTGCCTTCGAATGGCAGGAAGACGGTGGTCA
GTCCCCTGTGCAGCTGACATGGACTCGCTTACCACAGGGTTTCAAAAACTCGCCCACGTT

ATTTAATGAGGCCCTGGACGAAGACCTCCGTGATTATCGGGGTTGAACACCCTACCATTG
TTTTATTACAATATGTTGATGACCTTATGCTGGCAGCGGCTACAGAGAAAGAGTGCCAAG
AGGCAACAGGTGACCTTCTCCAAACCTTGGGGACTTTAGGTTACCGGGCTAGTGCCAAAA
AGGCCCAGATTGCCAAGCAAGAGGTTACATACCTCGGTTATAAGATAAAACAGGGCCAGA
GGTGGCTAACACAGGCTATGAAAGAAACCATCCTCCAGATCCCTGAGCCAGCTAACCCTA
GACAAGTGAGAGAATTTCTGGGAATGGTGGGATATTGCTGGTTATGGATCTTGGGGTTTG
CAGAAAAGGCCAGGCCCCTATATGAGGGGACCAAAGAAAACAAGGACTGGAAACGGACTG
AGCCAATGAAAGAGGCCTTCCAAGAGCTCAGGCGAGCCTTGCTAGAAGCTCCTGCCCTTG
CCCTCCCTGATCCGTCTAAGCCTTTCCAATTATTTGTAGATGAAAAGCGGGGGATAGGAA
AAGGGGTACTAACACAGAGATGGGGACCATGGAAGCGACCTGTAGCTTACCTTTCCAAGA
GACTGGACCCAGTGGCAGCCGGATGGCCACCTTGCCTCCGTATCATCGCGGCCACCACGC
TCTTAGTCCACGATGCTGATAAACTGACTTATGGACAGAGACTCTTGGTCTACACTCCTC
ATGCCATAGAGAGAGTTTTAAAGCAGCCCCCAGGTAAATGGATTTCTAATGCCCGCTTGA
CGCACTACCAGGCCTTGCTACTTGACACCCCATGGATTCATTTCCAAATGCCCTGCACTC
TAAATCCGGCCACTCTTTTGCCCAATCCGGGGGAAAATAGCCCCCTCCATGATTGTGATG
AGATACTGGCCGGGGTAACAGCAATGCGAAAGGACTTAACCGATACTCCACTGGATAACA
GTGAGCTGAAATGGTTCACAGACGGCAGCAGTTATGTAAAAGATGGACAGAGACGGGTCG
GAGCCGCAGTAGTAGATGACTCTGGACGGACGATATGGGCAGAGGCCCTTCCCCCGGATA
CCTCAGCACAAAAGGCAGAGTTAATTGCCCTGATTCAAGCATTAGAGAGAGCCAAAGGAA
AAAGAATAACTCTTTTCACTGACAGTCGCTATGCTTTTGGCATGGTACACATCCAGGGCC
TGATATATCGGGAACGAGGGTTTTTGACAGCTAAAGGAAAAAAAATTAAAAACTTGCCTG
AAATCCGTAGACTTTTAGAGGCTGTACAGATGCCTCGGGCTGTGTCAATAGTACACGTAC
CTGGACATCAGAAGGGTGACAGCCCCACGGCACAAGGGAATCGTGCCGCAGACCTGGCAG
CTCGAAAAGTAGCTGATAAAGATTTCATCACCCCTGTGTTGGTGATCGGACTTCCACCTC
CAGGTATGGGAACTCTGCCCCCAACCCCTGTGTATTCATCCACAGACTTTGCTTGGATCC
AAAAACGCACCAACCTTCAAAAAGATAAAGATGGATGGTACCGAGACTCAGACGGCTACT
TGATACTCCCTGCTCAGTTGGGACGGCAACTATGTGAGCATTTGCACTTGTCTACTCATC
TGGGAGAAAAGAAGACTCTGATGCTCTTTCAAACTGCGCGCCTGCGATTTCCCCGGCACC
AGACAACTGTAAAGAACATAGTGCATGCTTGTAAGGCATGTCAACAGATGAGGCCAGGAA
AAGGACAACATGCAGGACTGAGGTATCGGGGAGAAGGACCAGGGCAACACTGGGAAATAG
ATTTCACCGAGGTAAGGCCAGGCAAGTATGGTTACCGGTACTTGTTAGTGTTGGTGGATA
CCTTCTCAGGGTGGGTGGAGGCTTTTCCTACAAAGGGAGAAACCGCGATGATAGTGGCAA
AAAGATTTTAGAAGAAATAGTTCCCAGGTTTGGCCTGCCAGTGACCATCGGCTCTGATAA
TGGACCTGCTGTTGTGAGTCAAATAGTTCAGAGCCTTGCCCTAGCCCTGGGGACTAAATG
GAAGTTACATTGTGAATACAGTCCACAGAGCTCAGGGCAAGTAAAAAAAATGAATCAGAC
TCTAAAAGAAACTTTAACTAAATTGGCTATAGAGACTGGCGGGGACTGGGTGACCCTCCT
TCCCTTCGCCCTCTTCCGTGCGCGTAATACTCCTTATCAACTTAATCTGACCCCATTTGA
GATTCTGTATGGGAGACCTCCCCCTGTATGTCCAATATTTGAAGGGAAGAAACTACCGCC
TCCCACGTTGGGGCAACTCCAAGAGGCCTTGATGGCCTTAAGCAAGGTGCACTCTCGTGT
CTGGAAACTGCTCCAGGAAATACATGTGGGTCAAAATAAGGGAACTATTCCCTCACATGA
CATTGGCCCAGGAGACTGGGTATGGGTCAAAAGGCACCAAACCAATGCACTAGAACCCAA
ATGGAAGGGTCCTTATGTTGTTCTTCTTACCACCCCAACTGCCCTAAAGGTTGACGGTAT
CGGGCCTTGGGTGCATTGCAACCACGTACGCCCAGCTGCTTCAGCAGAGCAAGAAGACGC
TAAGAAAAAAATGGGAAGCATCTCTGCACCCATCCAACCCCTTGAGGCTAAAGCTTCGAAG
GCGCCAATAGGACCAGGACAACTCGGCTGGGCCCTCTTGTGGATGACCCAGTTACTCTGC

TCCGGAGTTGCCAGCGTGAACCCGCATCAACCCATCAAAATCACCTGGAAGCTGCAAAAT
GGACTAACACGAGAGGTGCTAAACTCCGCTACTGCAATACATCCACCAAACACATGGTGG
CCAGACTTGTACTTTGACCTTAAGCCGATGGTAAATGTGCCTTGGGCTAGGGGTTATCTC
CGAGAACAAGGGTTCTGGGCATGCCCAGGCGCACCCAGGCATGACTGGAAGACCTGTGGG
GGGGCACAAGACTCTTATTGTAAAACTTGGGATTGTGTTACTTCTAATGAGGGACCTCGG
CACTGGGAAGTAGGAAATCGAGATTTACTTAATTTTTCATTCGCCAAGCCCCTCCCTAGG
GTCCTCGGAGATCCAACATTTAGCTGTGAAAGTTGCAATTATGCACAAGTCAGAATAAGG
TTCAATCCAGAAAAAAGCAAAAAAGAGGGGACCTGGATCTCTGGCCTATCTTGGGGGCTA
CAGACGAGGGAATCAGGATGGTTTGGGGTTGATGGGAAAGGGATTATAGTAGTGAGCCAG
GTCTTAGAGCCAATTCTTGTACACAGTATCGGGCCCAACAAAGTAAAAAAAGCTGGCTGT
AACCCCCCGCCTAACGACTTCCATGCCAACATCTTTGGCAGTGAGTCCCGAAGCAGAAGC
ACTCGCTGAAATAGATCCCCTATGGAAGCTGATTAGGGCAGCTTATGCCACCCTGAATCA
GACCCATCCTGAGGCAACTAAATCTTGTTGGTTATGTTCCAACTTAATTCCCCCTTATTA
CGAAGCAGTAGGCCTCAATGCCTCTTATGACTTGGCCAACAGCACCGATCCTCCTCAGTG
TCACTGGGGGGACCGAAAGGTGGGTCTGACGATGAAAGAGGTATGGGGAAAGGGCTTATG
CATGGGCACGGTGTTACCAGCAAAGCCTCCACTTTGTGTGCATGTCGTCGAGCCTGATGA
TTTGCCTGTAGCCAAATGGTTAATACCTCAAATGGGGAGATGTTGGGTCTGTTCACACAC
GGGGCTAACTCCATGTCTGCATAGCTCAATCTTTGACCCCAAAGAAGAATTCTGTGTTAT
GGTGGCTGTCATGCCAAAGATTCTGTACCGACCAAAAAAAGCAATATATGATTATTGGGC
CCAGAAATTAACTCTAATTCCTCCAAAAAGAACTTACAAAGTTAAGAGGGAACCTCTTAC
CACCATAACTATAGCAACTATGTTCGGTCTTGGAATAGCCGGGGCTGGAACCGGAATAAC
AGCTCTGTCCCTGCAAGGCCAAGGATTTAACTCCCTGAGAGCGGCCATAGATGAAGACAT
TACCTGTATAGAGCAATCTATTAGTCATTTAGAATCGTCTCTAACTTCTCTATCTGAAGT
AGTTCTGCAAAACAGGAGGGGATTAGATCTGCTTTTTCTGCAACAAGGGGGACTCTGTGC
TGCCCTAGGAGAAGAGTGCTGTTTCTTTGTGGATCATACAGGAATAGTTAGAAAATCTAT
GGCCAAAGTGAGAGAAGGACTAGCCCAACGTAAACGAGAACGTGAGGCTCAACAGGGATG
GTTTGAATCTTGGTTTCAACAATCCCCTTGGCTGACTACCTTAATCTCCACCTTGTTAGG
ACCCCTGCTAGTACTATTACTAATGCTTACCGTCGGCCCATGTATTATCAATAGACTTGT
AGCCTTTGTAAAGGAACGCATAAATACAGTACAGCTGTTTGTGCTTCGACAACAATATCA
AACTGTGTCTCAGGACCGAGAGGAAGATTCCTCTATATGATCTAAGGACAGGGGTGGAAT
GTTAGGTACCAAACGACGGTCTCTAGGACCTGAGTCATGTTTACCAGAAAGAGACAGGAT
ATGTGCTCATCCTGCCTGGCCAATCATGTAACGCCAGCTACTCCTGTAACGGAGACAAAG
AACTGCCTGTATATAAGCCGCCATACTCCTTTGTTCATGGCTCTTGTCAGATTCCCTTGT
GTGGGATGAGACTTGGGCCCTAGCGCGCTAGAAATAAACAAACTCCCTTCTTGCGTTTGC
AATACTGTGGTGGACTTGCTCTC
>CAP_ERV_5
TGTTAGGGCCAGCGCCAGGACAGGCTCATCAGGCCACACTAGGCCTAAAACCTGGGTCAT
GATTCCAGGAACTACACACCCGAGCCAATCAAGAGGGCCCCCGTCTGAGCCATAGTTGAG
CCAATCCGGATAGAGATGCAGGATTCAAAATTCGTGGGTGCGCGTACGGCTCAGCCAATC
ACCACATGCCAACCACACTATTGCTGAGACAAAGGACCGCCTGTACATACGCAGCTATGA
TTCAGAGCTCGGGGCTCTCATCCAGACTCCACTGCGCTGGATGAGACTTGAGCCCTAGCT
CGAGCTAGCAATAAACCCCTTTATGCTTTTGCATTGCTGTGGATGTCTTATTCTCTCAGT
TTTGGAGACTCGGACACTGGGTATAACATTTGGGGGCTCGTCTGGGATGCCTTTTGACTG
AGGAGAAAAACACCCTCCTGGCAGAAGGGGTTCCTTAGCTCAAAGGGGACTAAACGTAGC
CAGTATAAGGCCGAGGCCCTGCATTGTGCTGGGAAGGTGGTTGGCTCTGTAAGGGGACTA

AACATAGCCGGCGTAAGACCGAGTCCCGGCATCGTGCTGGGAAGGCGGCTGGCTTTGTGG
ACGCCTTGTTGGTAAGATTACCTCGAGGGAAAGAGAGTTTCAGGGGGGCCCAAGAAGACC
TAGTGCTGTGTTCTTGGCCAATGTGTATTTGTTATCTGTTTGTAGTGTCTGTGTGAGTGC
CGGCATAGTCTCTGTTCTTTATGTGTTCATTTTGTCTCCTGTGTTCTCTGTAATCGTGGG
GCAAGCGACTTCTACTCCTTTGTCCCTTATGACTGACCACTTTTCTGATTTTAAGTCTAG
AGCACAGAATCTATCCGTGTTGGTAAAGAAGAACAAACTAATGACTTTTTGTTCCGCCGA
GTGGCCTGCCTTTAATGTCGGCTGGCCACGAGAGGGAACCTTCTGCCTACCAATTATTCG
AGCAGTTACAGAGAACATGTTCGCTCCTGGCCCTTCTAGACACCCAGACCAAACTCCCTA
TATTTTGGTCTGGCAAGATTTGGTGGGAAATCCGCCAGCCTGGCTGAAACATTTTATTCC
CCAGCCCTTTACTTCCCCTTCCCCCACTTCGTCCATCCCACAGGTACTTGTTGTGGAAGC
ATCCAAAGAGGAAGAGCACAAACAGTGCAACGGCTGGGTGAAGCCAGTATTCCAGGAATC
CTCACTCTGTCCTAACCTAATTGACCTGGAAACCGATCTCTCCCCACCCCCCTATGCGCA
TCCACCTTTGCCCCCTCAGGTACCTCAGATTTCATCCAGAGGAACACGAAAGGATACCGA
ACCCTCGGCCCCAACTCAGGAGGGGGGCCCCACCCAGGGAACTCGAGGAAGAACTAGGGA
ACCCTAAATGTGGCGGACGAGGACAGCCCAGAAGCCCCACCTCCACAGTCCGGGCACTTC
CTGTTTGGGCGGGGCCAGCCAGCCCGGTAGGGGAGCAAACTTACCAATACTGGCCCTTTT
CCACTAGTGATCTATATAATTGGAAAACTCAAACTCCTTCATTCTCGGAAAAGCCTCAGG
GCCTCATTGACCTTTTAGAATCCATTCTCTTCACCCATAACCCCACTTGGGATGATTGTC
AGCAGCTGTTACAAGTACTCTTCACCACAGAGGAACGTGAACGGATATTGTCAGAAGCAC
GGAAAAATGTTCCAGGGGTGGATGGGAGACCTACCACGCAACCGAATCTGATTGATGAGG
GATTCCCCTTGACGAGGCCTGGATGGGACTTTGAGCATGCTGAAGGTAGGGAGTATCTCC
GAATGTACCGCCAGACTCTTATGGCTGGTCTTTGAGCAGCCGCCAGGAGACCAACGAATT
TGGCTAAGGTAAACCTGGTTAGACAGGAGCTGAATGAGAGCCCAGCAGCCTTCCTTGAGA
GATTAATGGAAGCTTTTAGGCAATATACCCCCATGGACCCACAGGCCGACGAGTCACGTG
TGGCAGTTATGCTAGCATTTGTGAATCAAGCAGCCCCAGATATCAGGAGGAAACTGCAGA
AAATAGGTTAGGGGAACAATCCATACAAGACCTGGTGAGGGCAGCAGAGAGGGTTTTTAA
CCATAGGGAAACTCCAAAAGAAAGGGAAGAACGTGTTAGATGGGAAGAGAGAGAGTTCAA
GGCCTAGGAAAACCACAGAAATCAGAAAGAATTAGCCCAGATATCCTTTGCAGGGGTGAG
AAAGGGAGCTGATTCTCAAGGGACTAATGAAGTTAGGCTGAGAGACGAAGAAAAGCTAGT
CGGACAGAGACTAAACAAAGACCAATGTGCACATTGCCAGGAGTGAGGACACTGGAGAAG
AGAATGCCCTAAAAGAAAGTTGGGGGGAAAGCCCACCAGGAAAGAAGCACCTTCCCAGGG
GGCCCACGTCTTATATGCAGGAAACGATAGCGATTAGGGGGGGTCAAGGCCCGGCCCCCC
TCCCCGAGTCCTGGGTAACTACATGTGGAGGGGAAACCATTCTGCTTCATGGTGGACATG
GGAGCCCAATACTCAGTCCTTAACCGAAAAGACGGACCGATGTCCAAAAAGACCAGCTGG
GTACAGGGGGCCACCAGAACTAAACGATATGGATGGACTACTAAGCGTCAAGTGGACTTG
GGGGCCCAACAAGTGTCCCACTCATTTCTCGTGATACCAGAATGCCCAGCTCCCTTACTA
GGAAGGGATTTATTATCTAAGGTCAATGTTCAGATTCACTTTGACCACGGAGGGATATCA
GTTATGGATGGGACCGGATACCCTATACAAGTTTTGTCCTTGGCACTGAGGGATGAATAT
AGACTATACCAGCCGGGGCCTCCCATGGCTATTGACCCTAATGTGCAACCTTGGGTCCAA
AAATACCCCCTGGCCTGGGCAAAAACAGCGGGGGTAGGGCTAGCCAAGCAGAGACCCCCC
ATCTTTGTTGAACTGAAAGCAGATGCCACCCCTATACAGGTAAAACAGTACCCCCTGAGT
CTGGAGGCCCAATGAGGAATCATGCCACATATCCAGCGGCTCTTGAAGGTAGGGATTCTC
AAAAGGTATCGATCTCCGTGGAACAATCCCCTGTTACCAGTGAAAAAGCCTGGCGGAATG
GACTTTAGACCAGTCCAGGATCTTCGTGAAGTCAACGAATGGGTGAATGACATTCATCCC
ACCGTCCCTAACCTGTACACCCTCTTGAATGGCCTGCCACCAGACTATGTCTGGTATACA

GTCCTAGACTTGAAGGATGCTTTTTTTCAGTTTGCCGCTGGCCCATTCGAGCCAAGAGAT
CTTCGCTTTCGAATGGACCAAGGAGGGCAGCCAGACTACAGGACAACTAACCTGGACTCG
CCTCCCTCAAGGCTTCAAAACTCACCAATGCTATTTAATGAGGCTCTGGGTGAAGACCTC
CGTGAGTACTGGGCTAACCACCCCAATGTTGTCTTGTTTCAGTATGTGGATGATCTTATA
TTAGCCGCTGCTACTGAGGAGGCATGCCTAGAGGCGACAGGCGACCTCCTCCAAACTTTG
GGGACATTGGGCTACCAGGCTAGTGCAAAGAAGGCCCAAACTGCTAAACAGGAGGTCACC
TACTTAGGATATAAGATAAAGCAGGGGTGGAGATGGCTGACACAGGCTATGAAAGAGACC
ATTTTGCAGATCCCTGAGCCAGCAACTCCTCATCAAGTGAGAGAATTTCTTGGGACTGTT
GGGTATTGCAGGCTATGGATTTTGGGGTTTGCGGAAAAGGCCCGGCCATTGTATGAAGGG
AGTAGGGAGAGTAAAAATGGGACTTGGACTGAGCCAATGAGACAGGCATTTCAAGAACTT
CGGCAGGTGTTGCTGAAAGCCCCGGCCCTTGCCCTCCCTAACCCATCTAAGCCCTTTCAA
CTGTTTATGGATTAAAAAACAGGGAGTAGGAAAGGGAATCTTGATGTAGCAATGGGGGCC
TTGGAGGCAACCTGTAGCCTACCTCTCTAAAAGACTGGACCCGGTGGCCACAGGGTGGCC
ACCCTGCCTCCGAATCATTGCCACCTCTGCTCTCCTGGTCCATGATGCTGACAAGTTAAC
ATATGGACAACAGCTCCTGGTCTACACTCCCCATGCCATTGAAGGGATTCTCAAACAGCC
ACCAGATAAATGGATCTCCAATGCCTGCTTAACCCATTACCAGGCCTTACTGCAGGATAC
CCCCCAGATACACTTTCAGACGCCCTGCTTTCTGAACCCGGCCACTCTCCTGCCTGTCCC
GGAGAAAGACAGCCCCCTCCATGATTATGGTGAGATATTGGCTGATGTGATGGCCATACG
AAAAGACCTAAAAGATGTGCCCTTAAAAGACAATGAACTGGTATGGTTTACAGACGGAAG
TAGTTTTGTAAAAGATGGACAAAGAAGGGCAGGGGCAACCATTGTGGATGACTCTGGAAG
GGTCATCTGGGCTGAAGCTCTGCCCCCTGGGACATCCGCCCAAAAAGCAGAATTAATAGC
CTTGACACAGGCACTAGAGAGGGCAGAAGGAAAAAGGATCGCCATTTATACCGACAGCTG
GTATGCATTTGGAACAGTGCATATTCAGGGCCCAATTTATAGAGAGCAGGGGTTTACAAC
AGCAGAGGGAAAAGAAGTTAAAAACCTACTCGAGATCCTCAGACTCCTAGCAGAAGTGCA
CCGGCCCCGAGCAATGTCCATAGTACATGCCCCAGGACACCAAAAAGGAGAAGATATCAA
AGCTCGGGCAACGAGGCTGCCGATGTGGCGGCCTGGGAAGCAGCCCTTGGAGACTGCAAA
ACCCCCATATTGACTGTGGGATTGCCACCCCTGGGAAAGGGAACCCTGCCCCTGACCCCC
AAGTATTCCTCTTCCGATCTAAGTTGGATTCAAGAGAATGCTAACTGTCCAGAGGGCAAA
GACGGATGGTATCGAGACCAAAATGGCAACTTGTTGCTTCCGGCTAACTTGGGTCGACAC
CTTTGCACGCACCTATATCAGACCATCCATCTGGGAGAAAAAAAAGACTCTAGCACTCTT
ACAGACAGCGCATCTGCAGTTTCCCCGACAAAAGGCAACTATACAAGACATAGCCCGTGC
CTGTAAGGCATGCCAGATAATGAGACCAGGAAAAAGACAACATATGGGTATAAGGTACTG
GGGAGAAAGGCCAAGACAACACTGGGAGATAGATTTTATAGAGGTAAGGCCAGGCAAGTA
TGGGTACCGTTACCTGTTAGTTCTGGTCGATACTTTCTCTGGGTGGGTGGAAGCATATCC
CACTAAGAGGGAAACAGCAACAATGGTAGCCAAAAGGCTCCTAGAAGAGATAGTGCCTAG
GTTTGGGCTGCCAACAACCATCGGCTCCAATAATGGACCTGCTTTTGTGAATCAGATTGT
TCAGGGACTGGCCTTAGCTCTGGGGACCAAATGGGAGCTACATTGCAAATACAATCCCCA
GAGCTCAGGACAGGTTGAAAGAATGAATCGGACTCTAAAAGAAACTTTGGCAAAACTGGC
AATAGAGACTGGCGGAGACTGGGTAACTCTCCTTCCCTTCGCTCTCTTCCGAGCGCATTA
TACCCCCTACAAGTTGAACCTAATCCCCTTTGAAATTGTTTATGGGGGGTCCCCTTCAAT
GTGTCCTATCCTTGAGGGAAGAATCCAGCCACCTCCTTCATTGTGGCAATTCCAGGAAGC
CCTAATGGCCTTAAGCAAGGTGCATGTGCACATCTGGACTTCAGTCAAAGAAATCCATGA
AGGCCAAGAAAAGGGGACAATTCCTTCACACAACATTGGTCCGGGGGACTGGGTTTGGGT
CAAACGGCATCAATTCAAGACATTAGAACCTAGATGGAAGGGCCCTTATGTTGTTCTTAC
TACCCCTACTGCCCTGAAGGTCGATGGCATTGGACCTTGGGTGCACTGTAACCACGTGTG

CCATGCCACTACGGAAGAACAGGAAAAGGCCCAAAGAGAATGGAATGTGACACCACACCC
CTCCAACCCTTTAAGGATGAAGCTCATCCATTGACAGGACCCAGACGAATCACCCTGACC
TTCCTGTTGGTGACCATCCTCCTCGACCCGGGAGCCACCAGTGGAAACCCACACCAGCCA
GCCAAAATCACCTGGAAACTTCAAAATGGACTAATGCAAGAGGTGCTTAACTCAACCTCA
GGAATACATCCCCCAAACACCTGGTGGCCAGATCTGTACTTTGACCTTAAAGAGGTAGTA
GGCGTACCCTGGGCGAAAGGTCTCCTCCGACATTATGGGTTCTGGGCATGTCCTGGCCAC
CAAAGGAGTAACTGGAAGACTTGTGGAGGTCTACAGGACTACTTCTGTAAGTCCTGGACT
TATGTTACTTCTAATGATGGGAATTGGCATTGGGAAGTAGGAAACCGAGACCTACTCAAC
TTCTCGTTCACAAGGCCCTCGGCCCAAGCCCTCGGAGACCCATACTATAACTGTGACGAT
TATGACGATGCACAGGTTAAAATAAGGTTCAATCCAGAGGCTGCGAAAAAAGAGAGGTCT
CGGGTTTCCGGTCTGTCCTAGGGAATACAAACAAGTATCAAATGGACCCACGGGTATTTT
GGAGGAATAATAATTGTCAGCCAGATCATAGAGCCAACACAGGTGCATAGTATAGGACCC
AACCCAGTCGAAAAGACTGATTCACTAGAAATAGCTGAGACATATCTGACACCGAGTCTT
GCTACACTAGATTCTACATCCTCCAGTTCCCTCGGTCCCCCAGTCCCTACCCCCAACATC
GGGAAATCAGACCCATTATGGAAGCTTGTTAAGACAGCCTATACAACACTAAACCAAACC
AACCCAGAAGCAACTGAATCCTGTGGGCTCTGCTACACTCTATACCCTCCTTATTATGAA
GCAATAGGCTTAAATGCTTCTTACAGCCTGACCACTAGCATAGATCCACCTAAATGCCAC
TGCAGAGAATGAAAAGTGAGCCTCACAATGAGAGAAGTATGGGGAAAAGGACTTTGTGTA
GGTAAAGTCCCACCAGAGAAATCCCCTTTGTGTGCTCGGTCAGCCAAGCTCACAGAGATA
GATGAAACAAAACGGATTATACCAGAAGCAGGGGGTTGGTGGGTCTGCTCTCATACCAGA
CTGACTCCATGCTTACATGTCTCAGTTTTTAATCAAAATAGGGAGTTTTGCGTATTGATG
GCTGTTGTGCCCAAAAATCTTATACCACCCCGAAGAAGTTATATATAGCTATTGGACTGA
AAAAATAACAAATCAGCTGGCAGGGAACAGAGTTACAAGAGAGCCCATTACAGCCATCAC
ATTAGCAACCATGTTCAACCTTGGGGTTGTCGGAGCAGGAACCGGAATAATGGCCTTGTC
CCTCTAAGGCCAAGGGTTTACTTCATTGCAGGCAGCTATAGATGAAGACATCACTCACCT
AGAAGAGTCAATTAGTCACCTAGAAAAATCATTGACTTCCCTGTCCGAGGTAATTTTACA
AAACCAGAGGGGGTTAAATCTGGTTTTCTTGCAGCAAGGAGGACTCTGCGCTACTCTGGG
AGAAGAATGTTGTTTTTACGCAGATCATACCAGGGTAATGAGAGAATCCATGGCCACAAT
AAGGGAAGGATTAGCCCAACGAAAAAGAGCGAGGCCCAGCAGGGATGGTTTGAGTCTTCG
TTCCAACAATCTCCATGGCTAACAACACTGATCTCCACCCTGGTGGGGTCTCTTATGGTG
CTATTATTAATACTCACCTTTGGCCCATGTATCCTAAACAAACTTGTAATTTTTGTGAAA
GAACGTATTAATACAGTTCAACTACTCGTACTCAGGCAACAATACCAAGCCTTGCCCCAG
AACAAAGAGGAAGATTCCTGTATATGATCAAGAGACAGGGGGGAAATGTTAGGGCCAGTG
CCAGGACAGGCTCAGCAGGCCACACTAGGCCTAAAACCTGGGTCATGATTCCAGGAACTA
CACACTCGAGCCAATCAAGAGGGCCCCCGTCTGAGCCATAGTTGAGCCAATCTGGATAGA
GATGCAGAATTCAAAATTTGCGGGTGCGCGTACAGCTCAGCCAATCACCACATGCCAACC
ACACTACTGCTGAGACAAAGGACTGCCTGTATATACGCAGCTATGATTCAGAGCTCGGGG
CTCTCATCCAGACTCCACTGCGCTGGATGAGACTTGAGCCCTAGCTCGAGCTAGCAATAA
ACCCCTTTATGCTTTTGCATTGCTGTGGATGTCTTATTCTCTCAGTTTTGGGGACTCGGA
CACTGGGCATAACA
>CAP_ERV_6
GGACAGTAGCTCCTGCGTTAAACATTCCATAACCAAATAAGGAGGTCAGTAGCTCCTGCG
CTAAACATTCCATAACCAAATAAGGAGGTCAGTAGCTCCTGCGCTAAACATTCCATAACC
AAATAAGGAGGTCAGTAGCTCCTGCGCTAAACATTCCATACCAAATAAGAAAAATCTATA
GGGAAGCAGCATCAGGGGAATGTATGAGCTCAGCTTCGCATGTAACCAATCAGGTAGTGC

CAACTATGCCTGTTGCTGGACTAAGGGACAAACTGTATATAAACCGCCATACCTCTTTGT
TCGGGGTCCAGCCGCATTCTGCTGTGTCGGAGAGGCTAGGACCCTGGCGCGCCAGAAATA
AACTCCCTTTATGCCTTTTGCATTACTTTGGTGGACTTGTTCATTCGGTCGGGTAGGGGA
CACGGACAAGGAGCATAACATTTGGGGGCTCGTCCGGGATCTCCGCCCCACCGGAGGAAT
AATTCCCCTGGTAAGAGGGAGAAGCCCCGCTGACAGGCAAAGAGCTCTGAACTCCGAAGC
ACCGGTGCTAATAGTACAGGCCTAGATTAAGTCCGGGGCCCAGTATCGTACTGGGGAGGC
ATCTAGGTGTGAGAGACAAGGCAAGCCCAGCGTAAGGCCGAGTCCCCGGTAGCGTACCGG
GAAGGCGGCTGGCACTGAGGACGTCCTGACGGTAAGACATTTGCAAATAGAAAAGGGGTC
TCTAGTGCTAAAAGAGACTGAATTGGAATCTGTTTGTTGTGTCATTTTTGTGACTCTGTG
TGCCGGCACTGTCTGTGTGAGTGTCTTGTTGTCACTGTCTGTGTTCTCTGTGCCCATGGG
GCAATCTACTTCTACTCCGCTGTCCCTGATGACTGACCATTTTTCTGATTTTAAGTCTAG
AGCTCGGAATCTTTCGTTACTAGTAAAGAAGAGCAAACTGGTGACGTTCTGTTCCGCCGA
ATGGCCCACCTTTGATGTCGGATGGCCACAAGAGGGAACCTTCAATCCCCAGATCATCCA
GGCAGTTAAAGAGAGGGTGCTTACTCCTGGTCCTGCTGGGCACCCAGACCAGACTCCCTA
CATTCTGGTCTGGCAGGATCTAGTAAAGAACCCACCGGAATGGCTTAAACCCTTCGTTCT
TGCTTCTCCTAAACCTCCCCGTCCCTCTTCCCCGGCTCCAACCTTACTAAACCCACAGGT
GCTAGTCATGAAAGCGTCTGAAAAAACGAAAGAAAACCAGGACGAAAAACGACCCAAGCC
GGTGTTCCAGGAATCTTCCTCTCTGTACCCTAATTTGATTGACTTGGAAACCGAACTGTC
CCCACCCCCGTATGCGGATCCAAATCCACCTTTGCTTCCACAGGTTCCTCAAGTCTCGTC
GGGAGAAGCCCAGAGGAGGGCCGAGCCCTCAGCTCCCCCTAGGGGAGGAGGCCCCGCCCA
GGGAACTCGGGAAAGGGCAAGGGAGATGGCTAGCGCAGCGGAGGAGGAAGGCCCGGAATT
GCCCTCCTCCACTGTTTACGTGTTTCCGGTCTGGGCGGGGCCAGCCAGAGAGGGTGGGGA
ACGCACATATCAGTACTGGCCTTTTGCTACTAGTGATTTGTACAATTGGAAAACCCAAAC
CCCCTCTTTCTCTGAGAAACCTCAAGGTCTTATTGATCTGTTAGAATCTATCCTCTTTAC
TCACAACCCCGCTTGGGATGATTGCCAACAACTGCTACAAGTACTTTTCACTACAGAAGA
GCGTGAACGGATCCTGTCAGAAGCCCGGAAAAATGTGCCGGGGGCAGATGGGAGGCCCAC
AATACAGCCTCACCTCATTGAAGAGGGGGTCCCCTTGGTGCGACCCAACTGGGACTTCGA
ACGCGTTGAAGGTAGGGAGCGTCTCCGAGTATACCGTCAGATCCTCATGGCTGGCCTTAG
AGCGGCCGCCAGAAAACCAACTAATTTGGCCAAGGTAAATTCGGTGAGGCAAGAGCCCAA
TGAGAGCCCGGAAGCCTTCCTTGAAAGGATAATGGAAGCTTTTAGACAGTATACCCCCAT
GGACCCCCAGGCAGATGAGTCTAGGGCAGCAGTCATGCTAGCATTTGTAAATCAGGCAGC
CCCCGATATTAGGAGAAAGTTACAAAAGATAGAGAGGCTCGGTGAACAGTCCCTACAAGA
TTTAGTGAGGGCAGCAGAGAGGGTTTTCAATCATAGAGAGACCCCGGAAGAAAGAGAGGA
CCGCATTAGAAGGGAAGACAGAGAATTTAGGGCTGAAGAAAACCGTAAAAATCAAAAGGA
GCTGGCTCAGATATTTTTCGCTGGGATTGAAAACAAAAATAGATCCCAAAAAGGGAAAAG
GCCAGATTCAAAAACTGAGGAGAAACCTGCAAGGCGCAAGCTTGAAAAGAACCAATGTGC
ATTTTGTAAAGAGTTTGGACATTGGAAAGATAAATGCCCCAAGAAAAACCTAAAAGAGGG
GCCAAAAAATTCCAAGAATGAGACCCCCCCTCCTGACAGTCATATCCTCTATGCAGGAGA
GGATAGTGACTAGGGGGGTCAGGGCTCGACGCCCCTCCCCGAGTCCTGGATAACTATAAA
TGTGGAGGGGAAACCGGTTGGCTTTATGGTAGACACGGGAGCTCAATACTCAGTCTTAAA
CCAAAGAGATGGGCCTATGTCTAAGAAAAGTAGCTGGGTACAGGGAGCAACCGGGACTAA
GCGATATGGATGGACTACAAAACGGCATGTGAATTTGGGGACCCACCAAGTAACTCATTC
CTTTCTGGTGATACCCGAATGCCCAGCGCCCTTGCTGGGAAGAGATTTACTGTCTAAAGT
GAATGCCCAAATTCATTTTGGCCATGGACAAGTGTCAGTTTTAGATGGAACCGGGCACCC
TCTACAGGTTCTGTCTCTGGCATTGAAGGATGAATACAGACTGTACTTGCCAGAGGCCCC

AGCGACAGTAAGTCCTGAAGTACAGCCATGGGTTCAAAGATACCCTCAGGCCTGGGCTGA
AACAGCAGGAATGGGATTGGCCAGACAGAGACCCCCTATTGTTGTGGAGTTAAAAGCGGG
TGCCACACCAGTGAGGGTACGGCAATATCCCATGAGTCAAGAAGCCCGGCGAGGAATCAC
TCCTCACATACAACGCCTCACAGACGCTGGGGTCTTAAAGAGATGCCGGTCCCCATGGAA
CACCCCCCTGCTTCCCATAAAGAAGCCTGGGGGAACTGATTTTAGACCAGTTCAAGACCT
GCGAGAGGTCAACAAGCGGGTGAGTGACATCCATCCTACGGTCCCTAACCCTTATACATT
GCTAAGCAGTTTGCCACCAAGCTACGTATGGTATACTGTTTTAGATTTAAAAGATGCCTT
TTTCAGTCTGCCTCTCGCCCTGGCGTGCCAAGACATCTTCGCCTTCAGATGGCTAGAAGA
CGGTGGACAGACTCCTGTGCAGCTGACGTGGACTCGCCTACCACAGGGGTTTAAGAACTC
ACCCACGTTGTTTAACGAGGCCTTAGATGAAGACCTCCGTGAGTACCGGGTTGAACACCC
TACCATTGTTTTATTACAATATGTTGATGACCTTATGCTGGCGGCGACTACAGAAAAAGA
GTGCCAGGAGGCAACAGGTGACCTTCTCCAAACCCTTGGGACTTTAGGTTTCAGGGCTAG
CGCCAAAAAGGCCCAGATCGTCAAGCAAGAGGTTACATACCTTGGTTACAAGATAAAACA
GGGCCAGAGGTGGCTAACACAGGCTATGAAAGACACCATCCTTCAGATCCCTGAACCTAC
CACTCCTAGGCAAGTGAGAGAGTTTCTGGGAACTGTAGGATATTGCCGATTGTGGATCTT
GGGGTTTGCAGAAAAAGCCAGGCCCTTATATGAAGGGACCAAAGAAAACAAAGACTGGAA
GTGGACTGAATCAATGAAAACGGCTTTTCAGGAGCTCAGGCGTGCCTTGCTGGAAGCTCC
TGCCCTGGCCCTTCCTGACCCATCTAAGCCGTTCCAATTATTTGTGGATGAAAAGCGAGG
GATAGGAAAAGGGGTACTAACACAGAAATGGGGACCTTGGAAGCGTCCCATAGCCTACCT
TTCAAAGAAATTAGACCCAGTGGCAGCCAGGTGGCCACCTTGCCTCCGAATTATTGCAGC
CACCGCGCTTCTAGTCCATGATGCTGATAAGCTGACTTATGGCCAGAAACTCTTGGTTTA
CACTCCTCATGCTATAGAGAGAGTCCTGAAACAACCTCCGGGCAAATGGATTTCCAATGC
CCGCTTAACACACTACCAGGCCTTGCTGCTCGACACCTCCCGGATTCATTTTCAAACACC
CTGCACTCTAAACCCAGCTACTCTTTTGCCCAATCCAGAAATAGATAGCCCCCTCCACGA
TTGTGACGAGATACTAGCCGGAGTAACAGCAGTACGAAAGGACTTAACAGACACGCCACT
GGATAACAGTGACCTAATATGGTTCACAGATGGAAGCAGTTATGTTAGAGATGGACAGAG
ACGGGCGGGAGCCGCAATAGTAGATGACTCTGGACAGACGATATGGGCAGAGACTCTTTC
CCCAGACACCTCAGCCCAAAGAGCAGAGTTAATTGCCCTGATTCAGGCATTAGAGAGAGC
TAAAGGTAAAAGAATAACTATTTTCACTGACAGTCGCTATGCTTTTGGCACGGTACACAT
TCAGGGCCCGATTTATCGGGAACGCGGGTTTTTAACAGCTGAAGGAAAAGAGATCAAAAA
CTTACCAGAAATCCGTCGACTTCTGGAAGCTGTGCAGTTGCCTCGAGCTGTATCAATAGT
ACATGTACCTGGACATCAAAAGGGGGACAGCCTCACAGCCCGAGGAAATCGTGCTGCTGA
TTTGGCGGCTCGGAAGGCGGCTGACAAAGAGTACACCGCTCCAGTGCTGGCAATCGGACT
TCTGCCCCCAGGTATGGGAACTTTGCCCCCAACCCCTGAGTATTCGTCCACAGACCTGGC
CTGGATCCAGGAATATCCCAACCTCCAACAAGGAGAGGATAAATGGTACCGGGACTCCGA
TGGCTACTTGATACTTCCTGCTCAGTTGGGACGACAACTGTGTGAACATCTGCACTCATC
TACTCATCTGGGAGAGAAAAAGACTCTGCTGCTTTTTCAAACCGCACGCCTATGATTTCC
CCGGCACCAAACAACCGTAAAAAACATAGTACAAGCTTGTAAGACATGCCAACAGATGAA
GCCAGGAAAGAGGCAACACGCAGGACTGAGGTATCGAGGGGAAGGCCCAGGACAGCACTG
GGAGATAGATTTTACTGAGGTAAGGCCAGGCAAGTATGGTTACCGCTACTTGCTAGTGTT
GGTAGATACCTTCTCAGGGTGGGTAGAGGCTTTTCCTACTAAGGGAGAAACTGCAATGGT
AGTGGCTAAAAAGATTTTAGAAGAGATAGTACCCAGGTTTGGCCTGCCGGTGACCGTTGG
CTCTGATAATGGCCCTGCTTTTGTGAGCCAAATAATACAGAACCTTGCCCGGGCTCTAGG
AACTAAGTGGAAATTACATTGTGAATACAGCCCACAGAGCTCGGGGCAAGTTGAAAGAAT
GAATCGGACCCTAAAAGAAACTTTAACTAAATTGGTTATGGAGACTGGCGGGGACTGGGT

GACTCTCCTTCCCTTCGCCCTTTTTCGCGTGCGTAATACTCCTTATCAGCTCAATTTGAC
CCTATTTGAAATTCTGTATGGGCGCCACCCTCCCGTATGTCCAAAATTTGAAGGGAAAAA
GTTTCCACTTCCCACCTTGGGACAATTCCAGGAGGCTTTGATGGCTTTGGGCAAAGTACA
CTCTTGTGTCTGGAAACTACTCCGAGAGGTACATGAGGGTCAAGGTGAGGGAATTAGCCC
CTCACATAACATTGGCCCCGGGGACTGGGTGTGGGTCAAAAGGCACCACGCCAGGGCACT
GGAACCCAGATGGAAAGGTCCTTATGTTGTTCTTCTTACCACCCCAACTGCCTTGAAGGT
CGACGGTATCGGGCCCTGGGTGCATTGCAATCACGTACGCCCAGCTACTTCAGCCGAACA
AGAAGACGCGAAAAGACAATGGGAAGCGTCTCTACACCCGTCCAACCCTTTAAAATTGAA
GATCCAGCGCCGGCCGCAGGACCGACGAGACTCATCTGGACCGTCATCTGGATGACTGTG
TTGCTTTGTCCTGTGACTGCCAGTGTGAACCCCCACCAACCTGTTAAGATCACCTGGACG
CTCTGGAACGGACTGACTCGAGAGGTACTCAGTTTGACCACCGGAATTCACCCCCCTAAT
ACGTGGTGGCCGGATCTGTATTTCAACCTCAAAGACCTTATAAAGACTACTTGGACAGCG
GCCCAGACCAGAAACTTTGGATTCTGGGCATGTCCCGGACACCTGAAGAGACATAATTGG
GAGACTTGTGGGGGGCTGCAACATTATTTCTGTTGGTCCTGGAGCTGTGTGACCTCCAAT
GATGGGAGATGGAAATGGGAGGTCGGGAACCGGGACTTAGTCAATTTTAAAACCTCCCTA
TGTGTCAATGTCACAAGATCTGTAAACGGGCCTCTGGCTAGCTGGATGATTCCCCAGATG
GGAGGATGGTGGGTATGTTCCAGAACCGGGTTGACCCCTTGTGTACATGAGTCAATTTTT
GATCCCAAAGAAGAATTTTGTGTCATGGTAGCAGTAGTACCTAAAATAATATACAGGTCG
GAAGAAACTGTGTACGACTATTGGGCCCATAGGTCAACCCTCAATCAGCAAGAAAGAGCA
TATAGAATTAAAAGAGAACCCCTTACTGCAATTACTATAGCCACTATGTTTGGCTTAGGG
ATAGCCGGAGCTGGAACTGGAATTACAGCTTTGTCTATGCAAAGTCAAGGATTTAACTCT
TTAAGGGCAGCGATAGATGAAGATATCACCCGACTAGAACAGTCTATAAGTCATTTAGAG
TCTTCTTTAACTTCCTTGTCTGAAGTAGTTTTGCAAAATAGAAGAGGGCTAGATTTGCTA
TTTCTACAACAGGGGGGACTTTGTGCCGCCCTGGGAGAGGAATGTTGTTTTTACGCAGAC
CACATGGGTATAGTTAGAGAATCTATGGCCAAAGTGAGAGAGGGGCTAGCCCAGCGTAAA
CGAGAGAGGGAGGCCCAACAAGGATGGTTTGAGTCTTGGTTTCAACAATCCCCTTGGCTG
ACTACTTTAATCTCAACCTTACTAGGACCCTTGATAATCCTTCTAATAATGCTCACGTTC
GGTCCCTGCATCATCAACAGACTTGTAGCCTTTGTAAAAGAACGTATTAACACAGTGCAG
TTGTTTGTGCTACGGCAACAATATCAAACTATATAACTGGGAACAGAGGAAGATTCCTCT
GTATGATCAAAGAACAGGGGGGAATGTTAGGTTATCGGAAGACTTAGTGAGGTGTGTAAC
TACATCAGGGACCTGGGACAGTAGCTCCTGCGTTAAACATTCCATAACCAAATAAGGAGG
TCAGTAGCTCCTGCGCTAAACATTCCATAACCAAATAAGGAGGTCAGTAGCTCCTGCGCT
AAACATTCCATAACCAAATAAGGAGGTCAGTAGCTCCTGCGCTAAACATTCCATAACCAA
ATAAGGAGGTCAGTAGCTCCTGCGCTAAACATTCCATAACCAAATAAGGAGGTCAGTAGC
TCCTGCGCTAAACATTCCATAACCAAATAAGGAGGTCAGTAGCTCCTGCCCTAAACATTC
CATAACCAAATAAGGAGGTCAGTAGCTCCTGCGCTAAACATTCCATAACCAAATAAGGAG
GTCAGTAGCTCCTGCGCTAAACATTCCATAACCAAATAAGGAGGTCAGTAGCTCCTGCGC
TAAACATTCCATAACCAAATAAGGAGGTCAGTAGCTCCTGCGCTAAACATTCCATACCAA
ATAAGAAAAATCTATAGGGAAGCAGCATCAGGGGAATGTATGAGCTCAGCTTCGCATGTA
ACCAATCAGGTAGTGCCAACTATGCCTGTTGCTGGACTAAGGGACAAACTGTACATAAAC
CGCCATACCTCTTTGTTCGGGGTCCAGCCGCATTCTGCTGTGTCGGAGAGGCTAGGACCC
TGGCGCGCCAGAAATAAACTCCCTTTATGCCTTTTGCATTACTTTGGTGGACTTGTTCAT
TCGGTCGGGTAGGGGACACGGACAAGGAGCATAACATT
>CAP_ERV_7
TGTTAGGGACCAAACGACAGTCTCTAGGACCTGAGTCATGTTTACCAGAAAGAGACAGGA

TATGTGCTCATCCTGCCTGGCCAATCATGTAACGCCAGCTACTCCTGTAATTGAGACAAA
GAACTGCCTGTATATAAGCCGCCATACTCCTTTGTTCGGGGCTCTTGTCAGATTCCCTTG
TGTGGGATGAGACTTGGGCCCTAGCGCGCTAGGAATAAACAAACTCCCTTCTTGCGTTTG
CAATACTGTGGTGGACTTGCTCTCTCGGTCGGTTCAGAGATACGGGCTCCGAGCATAACA
TCTGGGGGCTCGTCCGGGATCTCCGTCCCGCCGGGGAGGACAACTCTCCTGGTAGAAGGG
AGTAACCTCGTTAGGAGATAAGGGCTCTGAGCACCGGTGCTAACGCTGCAGAAGCCCAGA
TTAAGTCCAAGGCCCGGTATCATACTGGGGAGGCATCTGGCTCTGGTTACTGGATTAAGT
CAGCGTAAGGCCGGGGCCTGGTTTCGTGCTGGGGAGGCAGCTGGCTCTGGTTACTGGATT
AAGTCAGCGTAAGGCCGGGGCCTGGCTTCGTGCTGGGGAGGCAGCTGGCTCTGGTTACTG
GATTAAGTCCAGCATAAGGCCGGGGCCCAGTTTCGTGCTGGGGAGGCATCTGGGTGTAAA
GTGCAGAGGTAAGCCCAGCGTAAGGCCAGGGCCCAGTTTCGTGCTGGGGAGGCGGCTGGC
TCTGGTTACTGGATTAAGCCCAGCGTAAGGCCAGGGCCCAGTTTCGTGCTGGGGAGGCGG
CTGGCTCTGGTTACTGGATTAAGTCCAGCGTAAGGCTGGGGCCCGGTTTTGTGCTGGGGA
GGTGGCTGGCTCTGTGAACATCCTGCAAGTAAGACTGAGTGCATTGTCGGTGGCCACCTT
GCGTTTGTTATCTGTTTGTCTATTCGTGGTGTCTGCGTTGCTCTTTGTGTGCTCTGTTGG
CTCCCAGTGTACTTTTCTGTGATCATGGGACAAACAACTTCTACTCCTTTATCTCTTATG
ATTAACCACTTCTCTGATTTCAAGTCTAGAGCACAGAATCTATCATTGCTGGTGAAAAAA
AAAGCAAGTTAGTAACTTTTTGTTCTGCCGAGTGGCCTGCTTTTGATGTCAGCTGGCCAC
AAGAAGGCACCTTCAGCCTGCCTACTATTCAAGTGGTCAGAGAGAAGGTGCTCACCCCCT
ACCCTTCAGGACACCCAGCCAGACCAAACTCCATACATTTTGGTCTGACAGAACCTGGTG
GAAAACCCCCTGGCCTGGCTAAAACCTTTTGTTTTTCAGTCCCTCACTTCCCTTCCCTCT
TCCCCTCCCTTGCTTCCACAGGTTCCACAGGTTTCATCAAGAGAAGCCAAAAAGAGAACC
AAGCCTTCAGCTCCTCCCAGAAAAAGGGGCCCCGCCTAGGGAACTCGGAGAAAAGGCAAA
AAAAAAAAAAAAAAAAAAAAAAAAAAAAATGGCCGGCGTAGCAGAAGAAGACCCGGAGGTT
CCTTCCTCCACCATTCATGTGTTTCCGGTCCGGGCAGGACCAGCCAGAGAGGGTGGAGAA
CGGACATATCAGTATTGGCCCTTCTCCACTAGTGATTTGTACAATTAAAAAACCCAGACT
CCCTCCTTCTCTGAAAAACCACAGGGTCTTATTGATCTTTTTTAGAGTCTATCCTGTTTA
CTCACAATCCCACTTGGTATGATTGTCAGCAACTGTTACAGGTACTTTTTACTACAGAAG
AGCACGAACGGATCCCTGTCAGAAGCCCTGAAAAATGTGCCAGGGGTAGATGGGAGGCCC
ACAATACAGCCTAACCTCATTGAGGAGGGGTTCCCCTTGGTGCGACCCAACTGGGACTTC
AAACGCGCTAAAGGTAAGGAGCCTCTCCGAGTGTACCGTCAGACTCTCATGGCTGGCCTT
AGAGAGGCCACCAAAAAGCCAACTAATTTGGCCAAAATAAATCCAGTGAGACAACAGCCA
AATGAGAGCCCAGCAGCCTTCCTGGAAAGGATAATGGAAGCTTTTAGACAGTATACCCCT
ATGGACCCACAGGCAGATGAGTCACGAGTGGCAGTTATGTTAGCATTTGTAAATCAAGCA
GCCCCCCATATTAAAAAAAAGTTACAAAAGATAGAGAGGTTAAATGAACAATCCTTGCAA
GATCTAGTGAGGGCAGCCGAGAGTTTTTAATCATAGAGAGACCCCAGAAGAGAGAGAGGA
CCACATTAAAAGAAAAAAAAAGAAAATTTAGAGCTGAAGAAAACCGTAAAAATCAAAAAG
AGCTGGCCCAGATATTTTTTGCTGGGGTTAAAAACAAAAACAGGTTCCAAAAAGGGAAAA
AATTGGACTCAAATACTGAAGAAAAAATGACAAGGCGTAAGCTTGAGAAAAACCAATGTG
CATTTTGTAAAGAGTTTGGACATTGGAAAGATAAATGCCCCAAGAAAAATCTAAAAAAGG
GGCCCAAGAACCCCAAGAACAAGACTCCCTCTCCAGACAGTCATATCCTCTATGCGGGTG
AGGATAGCGACTGGGGGGGTCAGGGCTCAAAGCCCCTCCCCGAGTCCTGGGTAACTATAA
ATGTGGAGGGGAAACCGGTTGGCTTCATGGTGGACACGGGAGCCCAATACTCAGTCTTAA
ACCAAAAAGATGGACCCATGTCTAAAAAAAGTAGCTGGGTGCAGGGAGCAACTGGGACTA
AATGATATGGATGGACTACAAAATGGCATGTGAACTTGGGGGCCCACCAGGTAACCCATT

CTTTTCTGGTGATATCTGAGTGTCCAGCACCCTTGTTTGGAAGAGATTTACTGTCTAAAG
TAAATGCCCAAATTCATTTCGACCACGGACAAGTGTCAGTTTTAGATGGGACCGGGCATC
CTCTTCAGGTCCTGTGTCTGGCATTAAAAGATGAATACAGACTCTACTTGCCAGAGGCCC
CAGCGACAATAAGCCCCGAAGTACAACCATGGGTTCAAAGATACCCTCAGGCCTGGGCTG
AAACAGCAGAAATGGGACTGGCCAAACAGAGGCCCCCTATCATTGTGGAACTAAAAGCCA
GTGCTTCCCCGGTGAGGGTACGACAGTATCCCATGAGTCAGGAGGCTCGACAAGGAATTA
CTCCTCATATACAATGCCTCATAGATGCTGGGGTCCTGAAAAGGTGCCGGTCCCCATGGA
ACACTCCCCTGTTGCCTGTGAAAAAGCCTGGGGGAACTGATTTTAGACCGGTTCAAGATC
TACGAAAAGTCAACAAACGGGTAAATGATATTCATCCTATGGTTCCTAACCCTTATACAT
TGCTAAGCAACTTGCCTCCAAACTACATTTGGTACACTGTTTTAGATTTAAAAGATGCCT
TTTTCAGTTTGCCTCTTGCCCCCGCAAGCCAAGAGATCTTTGCCTTCGAATGGCAGGAAG
ACGATAGTCAGACCCCTGTGCAGCTGACATGGACTCGCTTACCACAGGGTTTCAAAAACT
CGCCCACGTTATTTAATGAGGCCCTGGACGAAGACCTCCGTGAGTATCGGGTTGAACACC
CTACCATTGTTTTATTACAATATGTTGATGACATTATGCTGGCAGCGGCTACAGAGAAAG
AGTGCCAAGAGGCAACAGGTGACCTTCTCCAAACCTTGGGGACTTTAGGTTACAGAGCCA
GTGCCAAAAAGGCTCAGATTGCCAAGCAAGAGGTTACATACCTCGGTTATAAGATAAAAC
AGGGCCAGAGGTGGCTAACACAAGCTATAAAAAAACCATCCTCCAGATCCCTGAGCCGGC
TAACCCTAGACAAGAGAGAGAATTTCTGGGAACTGTGGGATATTGCCGGTTATGGATCTT
GGGGTTTGCAGAAAAGGCCAGGCCCCTATATGAAGGGACCAAAGAAAACAAGGACTGGAA
ATGGACTGAGCCAATAAAAGAGGCCTTCCAAGAGCTCAGGCGAGCCTTGCTAGAAGCTCC
TGCCCTTTCCCTCCCTGATCCATCTAAGCCTTTCCAATTATTTGTAGATAAAAAGCAGGG
GATAAAAAAAAAGGGGTACTAACACAGAGATGGGGACCATGGAAGCGACCTGTAGCTTACC
TTTCCAAGAGACTGGACCCAGTGGCAGCCGGATGGCCACCTTGCCTCCGTATCATCGCGG
CCACTGCACTCTTAGTCCATGATGCTGATAAACTGACTTATGGACAGAGACTCTTGGTCT
ACACTCCTCATGCCATAGAGAGAGTTTTAAAGCAACCCCCAGATAAATGGATTTCTAATG
CCCGCTTGACGCACTACCAGGCCTTGCTACTTGACACCCCACTGATTCATTTCCAAATGC
CCTGCACTCTAAATCCGGCCACTCTTTTGCCCAATCCAGAAAAAAAATAGCCCCCTCCAT
GATTGTGATGAGATACTGGCCAGGGTAACAGCAATGCGAAAAGACTTAACCGATACTCCA
CTGGATAACAGTGAGCTAAAATGGTTCACAGACGGCAGCAGTTATATAAAAGATGGACAG
AGACGGCTGGGAGCCGCAGTAGTAGATGACTCTGGACAGACGATATGGGCAGAGGCCCTT
CCCCCGGATACCTCAGCACAAAAGGCAGAGTTAATTGCCCTGATTCAAGCATTAGAGAGA
GCCAAAGAAAAAAAATAACTATTTTCACTGACAGTCGCTATGCTTTTGGCATGGTACACA
TCCAGGGCCCAATATATCCGAAACGGGGGTTTTTGACAGCTGAAAAAAAAAAAAAAATTAA
AAACTTGCCTAAAATCCGTAGACTTTTAGAGGCTGTACAGATGCCTCAGGCTGTGTCAAT
AGTACACGTACCTGGACATCAGAAGGGTGACAGCCCCACGGCATGAGGGAATCATGCCGC
AGACCTGGCAGCTCGAAAAGTAGCTGATGAAGATTTCATCACCCCTGTGTTGGCGATCAG
ACTTCCACCTCCAGGTATGGGAACTCTGCCCCCAACCCCTGAGTATTCATCCACAGACTT
TGCTTGGATCCAAAAACACACCAACCTTCAGAAAGATAAAGATGGATGGTACCGAGACTC
AGACGGCTACTTAATACTCCCTGCTCAGTTGGGACGGCAACTATGTGAGCATTTACACTC
GTCTACTCATCTGAGAAAAAAAAGAAGACTCTGATGCTCTTTCAAACTGCACGCCTGAGA
TTTCCCCAGCACCAGACAACTGTAAAGAACATAGTGCATGCTTCTAAGGCGTGTCAACAG
ATGAGGCCAGGAAAAGGACAACATGCAGGACTGAGGTATCGGGGAGAAGGACCAGGGCAT
CACTGGGAAATAGATTTCACCGAGGTAAGGCCAGGCAAGTATGGTTACCGGTACTTGTTA
GTGTTGGTGGATACCTTCTCAGGGTGGGTGGAGGCTTTTCCTACAAAGGGAGAAACCGCG
ATGATAGTGGCAAAAAAGATTTAAAATATATATATATATAGTTCCCAGGTTTGGCCTGCC

AGTGACCATCGGCTCTGATAATAGACCTGCTTTTGTGAGTCAAATAGTTCAGAGCCTTGC
CCTAGCCCTGGGGACTAAATAAAAGTTACATTATAAATACAGTCCACAGAGCTCAGGGCA
AGTAAAAAAAAATAAATCGGACTCTAAAAAAACTTTAACTAAATTGGCTATAGAGACTGG
CGGGGACTGGGTGACCCTCCTTCCCTTCGCCCTCTTCCGTGCGCGTAATACTCCTTATCA
ACTTAATCTGACCCCATTTAAAATTCTGTATGGGAGACTTCCCCCTGTATGTCCAATATT
TAAAAGAAAGAAACTACCGCCTCCCACGTTGGGGCAATTCCAAGAGGACTTGATGGCCTT
AAGCAAGGTGCACTCTCGTGTCTGGAAACTGCTCCAGAAAATACATGTGGGTCAAAATAA
GTGTTCCCTCACATGACATTGGCCCAGGAGACTGGGTATGGGTCAAAAGGCACCAAACCA
AGGCACTAAAACCCAAATGGAAGTGTCCTTATGTTGTTCTTCTTACCACCCCAACTGCCC
TAAAGGTCGACGGTATCGGGCCTTGGGTGCATTGCAACCACATACGCCCAGCTGCTTCAG
CAGAGCAGGAAGACACTAAAAAAAGAATGGAAAGCATCTCTGCACCCGTCCAACCCCCTG
AGGCTAAAGCTTCGAAGGCGCCAACAGGACCAGGACAACTCGGCTGGGCCGTCTTGTGGA
TGACCCAGTTATTCTGCTCCAGAGTTGCCAGCGTGAACCCGCATCAACCCGTCAAAATCA
CCTGGAAGCTGCAAAATGGACTAACACGAGAGGTGCTAAACTCCACTACCGCCATACATC
CACCAAACACATGGTGGCCAGACTTGTACTTTGACCTTAAGCCGATGGTAAATGTGTCTT
GGGCTAGGGGTTATCTCCGAGAACAAGGGTTCTGGGCATGCCCAGGCGCACCCAGACATG
ACTGGAAGACCTGTGGGGGGGCACAAGACTCATTGTAAAACTTGGGATTGTGTTACTTCT
AATGATGGACCTCGGCGCTGAGAAGTAAAAAATCGAGATTCATTTAATTTTTCATTCGCC
AAGCCCCTCCCTAGGGTCCTCGGAGATCCAACTTTTAGCTGTAAAAGTTGCAATTATGCA
CAAGTCAGAATAAGGTTTAATCCAGAAAAAAGCAAACAAGAGGGGATTAGATCTGCTCTT
TCTGCAACAAGGGGGACTCTGTGCTGCCCTAGGAGAAGAGTGCTGTTTCTATGGGGATCA
TACAGAAATAGTTAAAAAATCTATGGCCAAAGTGAAAAAAGGACTAGCCCAACGTAAACG
AAAACGTGAGGCTCAACAGGGATGGTTTGAATCTTGGTTTCAACAATCCCCTTGGCTGAC
TACCTTAATCTCCATCTTGCTAGGACCCCTGCTAGTACTTTTACTAATGCTTACCTTCAG
CTCATGCATTATCAATAGACTTGTAGCCTTTGTAAAGGAACGCATAAATACAGTACAGCT
GTTTGTGCTTCGACAACAAAATCAAACTGTGTCTCAGGACCGAGAGGAAGATTCCTCTAT
ATGATCTAAGGACAGGGGGAAATGTTAGGGACCAAACAACGGTCTCTAGGACCTGAGTCA
TGTTTACCAGAAAGAGACAGGATATGCACTCATCCTGCCTGGCCAATCGTGTAACGCCAG
CTACTCCTGTAACTGAGACAAAGAACTGCTTGTATATAAGCCGCCATACTCCTTTGTTCA
GGGCTCTTGTCAGATTCCCTTGTGTGGGATGAGACTTGGGCCCTAGTGCGCTAGGAATAA
ACAAACTCCCTTCTTGCGTTTGCAATACTGTGGTGGACTTGCTCTCTCGGTCGGTT
>CAP_ERV_8
GTTACAGCCTCACCAGAGCCATGACAGGCTCAGAGAGGTGCACTAGGCCAAAAATAATCC
CTGAGTCATGCTTCTGGGGCTTCTGGGGATGATAAATCTGGCCAATCAGAGAGATGATAA
CTCTGGCCAACCAGAGGGATGATAACTGGCCAATCAGTAAATACCAGGAAACCCTTGCAG
CCAATCAACCCTTGCCAACTCCCCGTCTTTGCTCTAAACTTATAAATACTGCTGTAAATC
TGGGCTCAGGGCTCTTGCTCCACTCCACTCCGTTGGATGTGGTGGGAGGCCCTGGCTCGA
GCTAACAATAAACCCCTTTATGCTTTTGCATTGCTGTGGATGTCTTATTCTCTCAGTTTT
GGGGACTCGGACACTGGGCAAAACATTTGGGGGCTCATCCGGGATCCTTTAACTGGGAAG
AATAACATTCTCCCGGTAGAAGGGGTTTCCTCACTAGGAGGAGAGATATCTGAACACCGG
TGTTAGTTTTGGATTAAGTCCAACGTAAGGCCAAGGCCCAGCATCGAGCTGGTGGGGTGG
GGGTGGGGGGGCAGCTGGCTCTGTGAACATCCTGCAGGTAAGACTGAGTGCATTGTTGGT
GGCCACCTTGCGTTTGTTATCTGTTTGTCTACTTGTGGTGTCTGCATTGGTCTTTGTGTG
CTCTGTTGGCTCCCAGTGTACTTTTCTGTGATCATGGGACAAACAACTTCTACTCCTTTA
TATCTTATGACTAACCACTTCTCTGATTTTAAGTCTAGAGCACAGAATCTATCATTGCTG

CTGAAGAAAAGCAAGTTAGTAACTTTTTGTTCTGCCGAGTGGCCTGCTTTTGATGTTGGC
TGGCCACAAGAAGGTACCTTCAATCTGCCTGCTATTCGAGTGGTCAGAGAGAAGGTGCTC
ACCCCTTACCCTTTGGGGCACCCAGCCCAAACTCCATACATTCTGGTCTGGCAGGACCTG
GTGGAAAACACCCCCGGCCTGGCTAAAACCTTTTGTTTTTCAGCCCCTCACTTCCCTTCC
CTCTTCCCTGTCCTCGGCTTCATTATGTCCACAGGTACTAGTTGTGGAAGCATCCAAAGA
GAAAGAAGACAAAAAGCACAGCAACCTGGTGAAACTGGTATTCCAGGAATCCTCACTATA
CCCCAATTTACTCGACCTGGAGACAGAACTCTCCCCGCCCCGCTATGGGGATCCACTTTT
GCCCCCGCAGGTTCCTCAGGTCTCTTCTGGAGGGATACAAAGGGACACCGAGCCTTCAGC
CCCAGCCTGGGAAGGAGGCCCCGCCCAAGGGACTCGGGGAAGAACCAGGGGCATCACCAA
TATGGCGGAAGAAAACAACCCAGAGGCCCCCTCCTCCACAGTCCGGGCATTTCCAGTTCG
GGCGGGGCCTGCCTGAGCAGACAGAGAACAGACATACCAGTACGGGCCCTTTTCCACCAG
TGATCTTTACAACTGGAAAACTCAGACCCCTTCATTCTCTGAAAAAACTCAGGGCCTCAT
TGACCTTTTAGAATCTATCCTTTTCACCCATAACCCCACCTGGGATGACTGTCAACAGTT
GTTACAAGTGCTCTTTACTACAGAGGAACGCAAACGGATCTTGTCAGAAGCACGAAAGAA
TGTGCCAGGGGTAGATGGGAGACCCACTACACAGCCTAACATGATTGATGAGGGATTTCC
TCTGACGCGGCCCTGCTGGGACTTCGAGAGCACTGAAGGTAGGGAGCATCTCCGAGTGTA
CAGCCTGACTCTCATGGCCGGCCTCCGAGTGGCTGCCAGGAGGCCAACAAATTTGGCTAA
AGTAAATCTTTTTAGACAAGAGCCAAATGAGAGCCCGGAAGCTTTCCTTGAGAGATTAAT
GGAAGCTTTTAGGCAGTATACCCCCCACGGACCCACAAGCCGATGAGTCGCACGCAGCAGT
CATGCTAGCATTCATGAATCGGGCAGCCCCGGATATCAGGAGAAAACTACAAAAAATAGA
GAGGTTGGGAGAGCAATCCCTGCAAGACCTGGTGAGAGCAGCAGAGAGGGTTTTTAATCA
TAGAGAGACTCCAGAGGAAAGGGAGGAATGTGTTAGGCTAGAGGAAAGAGAATTCAGGGC
CAAAGAAAACTGTAAGAATCAGAAAGAATTAGCCCAGATATTCTTTGCAGGGGTGGAACA
GGGAGCTAGTTCTCAGAGAACAAGGGAAGTCCATTCGAAGGGTGAAGGGAAGCCAGCGAG
GCAAGGACTTAAAAAGGACCAGTGTGCTTTTTGCAAGGAGATAGGACACTGGAAGAGTAA
ATGCCCCAAGAGAAACCTAAGGGAGAGACCCACCAAGCAGGAGGTGTCCTCCTCCTCCAC
AGGGGCCCACATCCTATATGCAGGAGAGGATAGTGATTAGGGGGGTCAGGGCCCGGCACC
CCTCCCTGAGTCCTGGGTAACTATACATGTGGAGGGGAAACCGGTTGGCTTCATGGTGGA
CACAGGAGCTCAATACTCGGTCCTTAACCAGAAAGACAGACCAATGTCTAAAAAGACCAG
CTGGGTACAAGGGGCCACCGGGACTAAATGATATGGACGGACTACTAAACATCAAGTGAA
CTTGGGGGCCCAGCAGGTGACCCACTCTTTTCTTGTGATACCAGAATGCCCAGCACCCTT
GCTGGGAAGAGACTTACTATTTAAACTCAATGCCCACATTCACTTTGACCACAGAGAGAT
ATCAGTTCTAGATGGGACCGGACATCCCATACACGTTTTGTCTCTGGCATTGAGAGACGA
ATACAGACTGTATCAGCCAAAGCCGCTCATGGCCATTGACCCCAATGTACAACCTTGGGT
CCAAAAATACCCTCTAGTCTGGGCAGAAAATGCGGGGGTAGGACTAGCCAAACAGAGGCC
TCCCATCATTGTCAAACTGAAGTCAGACGCCACCCCTATCCAGGTAAAACAATACCCTTT
GAGCCTAGAAGCCCGGTGAGGAATCACACCACATATACAATGGCTTCTGGAGGCAAAAAT
TCTTAAAAGGTGCCGATCTCCATGGAATACTCCTTTGTTACCTGTGAGAAAGCCAGGGGG
AATGGACTTTAGGCCTGTGCAGGATCTTTGTGAAGTCAACAAACGGGTGAATGATATACA
TCCAACCGTCCCTAACCCGTACACCCTTTTGAGTGGCTTTCCTCCAGACTACGTCTGGTA
TACTGTTCTGGACTTGAAAGATGCTTTTTTCAGTTTACCCTTGGCCCCCTTGAGCCAAGA
GATCTTCGCATTCGAATGGATAGAAGAAGGCAGCCAGACCTCAGGACAGCTAACTTGGAC
TCGACTTCCGCAAGGCTTCAAGAATTCACCAACGCTATTCAATGAGGCTCTGGGTGAAGA
CCTCCATGAGTACCAGGTCGATCACCCCAACATTGTTCTATTGCAGTATGTTGATGATTT
ATGCTAGCCGCAACCACTGAGGAGGCATGCCTAGAAGCAACAGGCAACCTCCTTCAAACT

TTGGGGACCTTGGGGTACCGGGCTAGTGCAAAGAAGGCTCAAATTGCTAAGCAGCAAGTC
ATATACCTAGGGTATAAAATAAAACAAGGACGGAGATGGCTGATGCAGGCCATGAAAGAG
GCCATATTGCAGATCCCTGATCCGGCAACTCCTCAACAAGTGAGAGAATTTCTTGGGGCT
ATTGGGTATTGCTGGCTATGGATCTTGGGATTTGCTGAAAAGGCCCGGCCACGATATGAA
GGAAGTAGAGAAAATAAAAACTGGACTTGGACTGAACCAATGAAACGGAATTTTCAAGAA
CTCAGACAGGCTCTGCTGGAAGCCCCAGCCCTTGCTCTCCCTGACCCGTCCAAGCCCTTC
CAATTGTTTGTAGATGAAAAACGGGGAATGGGAAAAGGAGTCTTGATGGGAAAGGTCGCC
TTGGAGGCGACCAGTGGCCTACCTATCCAAACGACCGGAGAAAGGCCAGGGCAACACTGG
GAGATAGATTTTACAGAGGTAAGACCAGGCAAGTATGGGTACCGTTATCTGTTAGTTTTG
GTGGATAATTTTTCTGGGTGGGTGGAAGCATTCTCTACTAAGGGGGAAACAGCAATGATA
GTAGCTAAAAAGATCCTAGAAGAAATAGAACCTAGGTTTGGGCTGCCGGTGACTATTGGC
TCTGATAACAGGCCTGCCTTTGTGAGTCAGATTGTACAGGGACTGGCCTTAGCTCTGGGG
ACCAAATGGAAGCTGCATTTTGAATACAATCCCCAGAGCTCAGGACAGGTTGAGAGGATG
AATCAGACTCTAAAAGAAACTTTGGCAAAATTGGCAATAGAGACTGGCGGGGACTAGGTG
ACTCTCCTTCCCTTCACTCTCTTCCGTGCGTGTAATACCCCCTACAAGCTGAACCTAACC
CCTTTTGAAATTATGTATGGGAGGCTCCCTCCCATATGCCCTATCTTTGAGGGAAGAAAA
CAACCACCCCCTACTTTAGGACAATTCCAGGAAGCCCTGATGGCATTAGGCAAGGTGCAT
ATGCATGTCTGGAAATTGATCAGGGAAATCCACGAGGGTCAGAACAAGGGGACCATCCCC
TCACATAATGTTGGTCCTGGAGATTGGGTCTGGGTCAAGCGACATCAACCCAAGACATTA
GAACCTAGATGGAAAGCTCCTTATGTTGTTCTTCTTACCACCTCCACTGCTTTAAAGGTC
GACGGTATCGGACCTTGGGTGCACTGCAACCATGTACGCCAAGCCACTCCAGAAGAGCAA
GAAAAGGCCCAAAAAGAATGGGAGGTAACGCCACACCCTTCAAACCCCTTCAAGATAAAG
CTCATCTGCCAACAGACAAGTCATCCTGACTGTCCTATTGGTGACCATCCTTCTCGACCC
TGGAGCCACCCATAACAACCTGTACCAGCCGGCCAAAATCACCTGGAAGCTTCAAGACGG
ACTAACACGAGACATGCTCAACTTAACCACAGAAATACATCCTCCAAACACCTGGTAGCC
AGACCTATATTTTGACCTAAAGCTGGTGGTGGGTGTACCTTGGGCAAGGGGTTTCCTCCG
GATGCAGGGGTTCTGGGCATGCCCGGGCTACCAGAGGACCAAATGGAAGACTTGTGGGGG
GGCACAAGATTATTTTTGTAAATCATGGGATTGTATTACTTCTGATGATGGGCCTCGGGG
CTGGGAAGTAGGAAATCGGGACCTACTTAACATCTCGTTCGCAAAGTCCACCCCCAGAGC
CCCTGGGGACCCAACTTTTGAATGTGGAGAGAGTTGTAACTATGCACAGGTTAAGACAAG
GTTCAATCAGAAAAAAGCACAAAAAAGAGAGGGCTTGGATTTCTGGCTTATCCTGGGGAA
TACAAACAAGAACGGTGGCAGGCGCGCAAATTTACGGAGGAATTATAATTATAAGCCAGA
TTCTAGAACCAACACAGATACACAGTATAGGTCCCAACCCAGTGGAGAAAATTGACGTAA
CTCCCAGCCCGACCACAGCGGTTTCCACATCCTTAGTCTCACTTGATCCTCTGGGTCCCA
TCCGCAACCTCAAAAACTTAGACCCATTATGAAAATTAGTTAAGGCAGCCTATATAACCT
TAAATCAAACTAACCCGGAAGCAACTAAATCTTGCTGGCTTTGCTATAATCTATACCCCC
CCCTCCTTACTATGAAGCAGTGTGTCTTAACGCCTCTTATGGCCTCACTGATAGTATAGA
TCCTCCTCAATGTCGCTGGAGAGAATGGAAAATGGGCCTCACAATGAAAAAGGTTTGGGG
AAAGGGACTCTGTGTGGGCAGAGTTCCACCTGAAAATTCCCCCTTATGTGCCTACACAAC
CAAACCCACAGAGCTAACTGAGATTAAATGGATTATCCCAGAGGTGGGGGGATGGTGGGT
CTGCTCACGTACTGGTCTAACCCCTTGTTTACACATCTCAGTATTCAATCCTAAAGAAGA
GTTCTGCATCTTGGTGGCAGTTGTGCCAAAAATCCTGTACCACCCTGAAAAGATAACGTA
CGACTACTGGGCCCAAAAAACAAACCCCCAGTCAAATGATACAAGGCAGAACCAAGAGAG
AACCTATTACAGCCATCACCATAGCAACCATGTTCGGCTTTGGGATCGCTGGGGCAGGGA
CTGGGATAGCAGCCTTGTCCCTCCAAGGCCAGGGATTTACTTCATTGCAGCCATAGATGA

AGACATTACCCACCTAGAAGAGTCAATTAGTCACCTAGAAAAATCGCTGACCTCCCTATC
CGAGATGGTCTTGCAAAACCGAAGAGGGCTAGATCTAAGTTTTCTGCAGCAAGAGGGGCT
CTGCGCGGCCCTGGGAGAAGAATGTTGTTTCTAAGCAGACCACACCGGGATAATAAGAGA
ATCTATGGCAAAGGTGAGAGGAGGACTAGCCCAGCGAAAGAGCGAGAAGCCCAGCAGGGA
TGGTTTGAATCTTGGTTTCAACAATCTCCGTGGCTGATGACATTAATTTCCACCCTGGTG
GGACCTCTTATGGTGCTTTTACTAATACTCACCTTTGGCCCATGCATCCTCAACAGGCTC
ATTACGTTTATAAAAGAGCATATTAATACAGTTCAACTATTTGTGCTTAGACAGCAATAT
CAAACTGTACCCCAGAACAAAGAGGAAGATTCCTCTATATGATCTAAAGACAAGGGGGGA
ACGTTACAACCTCACCAGAGCCATGACAGGCTCAGAGAGGTGCACTAGGCCAAAAATAAT
CCCTGAGTCATGCTTCTGGGGCTTCCGGGGATGATAACTCTGGCCAGTCAGAGAGATGAT
AACTCTGGCCAATCAAAGGGATGATAACTGGCCAATCAGTAAATACCAGGAAACCCCTGC
AGCCGGTCAACCTTGCCAACTCCCCATCTTTGTTCAAAACTTATAAATACTGCTGTAAAT
CTGGGCTCGGGGCTGTTGCTCCACTCCACTGTGTTGGATGTGGCGGGGAGCCCTGGCTCG
AGCTAGCAATAAACCCTTTTATGCTTTTGCATTGCTGTGGGTGTCTTATTCTCTCAGTTT
TGGGGACTCGGACACTGGGCAAAACATTT
>CAP_ERV_9
GCTCCATGACAGGCTTCAGGGGGCCAAGGTAGGCCTAAGTCTCCTTCACAGAGGGGCTGA
GTTATTGCCAAGGAGGAGCTGAGAGCCTCCTCTGCCAGTGCCAGGACTCGACCAATCAGT
GTACTCTCTGTAGCCTGGTTCAAGCTTATCAGGACGAGGATGAAAACCCCCTGAGAAAGC
CCGCGCACGCGAAAATCTAGCCAATTAGTAGATGCTAAGGAACCCTTGCAACCAATCCAC
CCCTGCCAACTCCCTGTCTTTGTCTTAACCCTTTAAATACTGCTGTAATTCAGGGCTCGG
GGCCCTTGTTCCACTCCACTGCGTTGGATGAAACTTGGGCCCTGGCTCGAGCTAGCAATA
AATTCCCTTTTTGCATTTGCATTGCTGTGGATGTCTTGTTCTCTCAGTTCTGGGGGTTGG
GACCTTGGGCATAACATTTGGGGGCTCGTCCGGGATCCCCTGACTGAGAAGAACAACACC
TTCCAGGCATAAGGAAAGCCTCACTAGGAGGAAGTATATCTGGACGCCAGCATCAGGTCA
GGGGGCGAAAGAGTCACCCGGCCAGCATAAGTCTGGGGCCCAGCATTCGAGCTGGAAGGC
AGCTGGTTCTGTGAGCCATTCTGTAAAGGAACAGGGGAAAACGTTTCTCCCGATCCTGAG
TCTGGTGGGCAGTGGACGCCCTTTGGTAAAGTAACTTTGGGGAACAGGACAACAGCAACC
CAGGATAAACCTAGTTCAGTGTTTTCAGCCGATATTTGTTCGTCTACTGTCTGTCGCTTG
TCTTTGTGTGCCGGTGGTGCGCTTGCTGTTTGTGCGTTTATTGTGGGTGTTGTCGCCATG
GGTCAAAGTTATTCTACACCACTATCTGTAATGACTGACCATTTTCCCGATTTTAAGTCC
AAGGCTCAGAATCTATCCGTGCTAGTAAAGAAGAGCAAGCTGAAAACCCTATGCTCATCT
GAGTGGCCGACATTTCAGGTTGGCTGGCCGCCGGAGGGAACTTTCGGCCTGCCGGCCGGT
CATCCAGGCTGTTAAAGAAAAGATAATGGCTCCTGACCCTCGGAGCCATCCGGACCAGGC
TCCTTACATCCTGGTCTGGGAAAATCTAGCAGAAGACCCTCCCGTCTGGCTAAAACCCTT
TGTTCACAACCCCCCAGTGCTTCTGTTCCACAGGTCCTGGTCATGGAGACCTCTAAGGAA
GAAAACCGGGAGGCACGGGAGAGCCGGAAGAAACCGATACTCCAAGAATCTTCCCTATAC
CCTAGTCTGATTGATTTAGACACTGAAATCTCACTTCCCCCATATATCCCGCCACCCTTG
CTCCCGCAGATACCTCAGTTGTCATCCAGAGAACAAAGGGGGAACTTAGAACCCTCAGCT
CTGCAGCAGGAAGGGGGGCCAGCCCAGGGAACTTGCGGAAGGACCCAAGGGGGCAGAGAC
TCGTCTGACTATGGGAGCCGAGAGGCCCCTTTGTCTACCGTTCGAGCGCTTCCTGTCCGG
GTCCGGCCGGCAAATCCTGACGGAGAATGGAATTATCAATATTGGCCGTTCTCTACCAGC
GACTTACATAACTGGAAAGCTCAAAACCCCTCTTTTTCTGAAAAGCCCCAAGGCCTTATT
GATCTTTTAGACACTATCCTGTTTACTCACAATCCTACTTGGGATGATTGTCAGCAGCTG
TTACAGGTGCTTTTCACCACAGAGGAACGGGAGCGAATTCTGTCGGAAGTGCAGAAACAC

GTTCCCGGAGCGGATGGGGAGACCGAGAATGCAGCCCCACCTAGTGGAGAAAGGGTTCCC
TTCTATGCGGCCGAACTGGGACTTTGAGCATGCGGAAGGTAGGGAGCATCTTTGGGTCTA
CCGCCAGACTCTAATGGCCAGCCTCCGGGCAGCCACCAGGAAACCGACAAATTTATCTAA
GGTGAATCTGGTAAGACAGGAGCCAAACGAGAGCCCAGCAGCCTTCTTAGAGAGGTTAAT
GAAAGCCTTTAGGCAGTATACGCCCATGGACCCCCAGGCTGATGAGTCACGCGCAGCAGT
TCTGTTAGCTTTCGTGAACCAGGCAGCTCCAGATATCAGGAGAAAGTTACAGAAGATAGA
AGGTCTGGGGGAACTGTCAATACAGGATCTGGTGAGGGCAGCTGAGAAAGTGTTCAATAA
CAGAGAGACCCCTGAGGAGAGGGAGGAACGGATTAGACGGGAAAAAAGAGAATATGAGAA
ATGGATTAGATGGGAGGAAAGGGAATATAGGGCCGAAGAAAACTGGAAAAATCAGAAAGA
GCTGGCTCAGATCCTTTTTGCGGGGATGAGAAAGGGGCCCAAAGCCCTGAGGACTAAAGA
CACCCGGTCGGGAGGAAAGGAAAATCCAGCTAGACCTGCCCTAAAGAGAGATCAATGCGC
TTACTGTAAAGAGCAGGGACACTGGAAAAAATGAGTGCCCCAAGAGAGATCTGAAAAAAA
AGACTGTAAGAAAGGAAGACTCTTCCTCGGGGACCCACGTCTTATATGCAGGGGAAGATA
GTGATTAGGGGGGTCAGGGCCCGGCACCTCTCCCCAAGTCCTGGGTAGCTATCAATGTGG
AGGGGAAACCGGTCGGCTTCATGGTGGATATGGGAGCCCAATACTCAGTCCTCAACCAAA
AATTTGGGCCGATGTCCAAAAAGACTAGCTTGGTCCAGGGAGCCACCGGGACAAAACTAA
ACGAAAAGTGGACTTGGGAGCCCAACGGGTGTCCCACTCATTTCTGGTGATCCCGGAATG
TCCAGCCCCTTTATTAGGAAGAGACCTATTGGCCAAAGTCAATGCACAAATTCACTTTGA
CTCTGGGGGAATATCAGTCACAGATGGGCTTGGACAACCAATTCATGTTTTATTCCTGGC
ACTGAGAAATGAATACAGACTATATTCACCAAAACCCCCTGCAGCTGTGGATCCTGCTAT
GCAACAGTGGATTCAGAAGTACCCTCTGGCCTGGGCAGAGATAGCAAGAATGGGACTGGC
TAAACAAAGACCTCCCATTGTTGTTGAATTAAAAGCAAATGCTATTCCTGTGAGGGTGAA
ACAGTATCCCATGAGTCAGGAGGCCCGGCAAGGAATTATGCCACACATCCAGCGCCTCCT
AAAGGCAGGAATTCTCAAAAAGTGCCGGTCCCCATGGAATACTCCCCTGTTGCCTGTGAA
AAAGCCCAGGGGAGCAGACTTTAGACCAGTCCAAGATCTTCGTGAAGTCAACAAACGGGT
GAGTGACATTCATCCCACTGTCCATAACCCATACACCCTCCTGAGCAGCTTGCCACCAGA
CTATGTCTGGTATACAATTTTAGACTTGAAAGATGCCTTTTTCAGCTTGCCTTTGGCCCC
CCAGAGCCAGGAAATCTTCGCCTTTGAATGGGCTGACGAGGACGGCCAAACTGTGGGGCA
GCTGACCTGGACTCGCCTCCCACAGGGGTTCAAAAACTCACCGACATTGTTCAGTGAGGC
TCTAGGCGAAGATCTCTGTGAGTATCGAACCAGCCACCCCGAAGTCGTTCTGTTGCAGTA
TGTAGATGACCTAATGCTGGCCGCTACAACTAAGGAGGTATGCCTAAAGGCCACAGGTGA
TCTCCTCCAGACTTTGGGGACATTGGGGTACTGGGCAAGTGCAAAGAAGGCCCAAATTGC
TAGACAGGAAGTCATTTATTTGGGATATAAAATAAAACAGGGGCAGAGATGGTTGACTCA
GGCTATGAAAGAGACTATTCTACGGATCCCCAAGCCAACAACTCCCCAGCAGGTGAGGGA
GTTTTTCGGGACGGTTGGGTACTGCAGGGTATGGATTATGGGGTTTGCTGAAAAGGCCCG
ACCTCTATATGAAAGAAGCAGAGAAAACAGAGACTGGACTTGGACTGAGCCAATGAGGCG
GGCATTTCAGAAACTTCGACAGGCGTTACTGGAAGCCCCAGCCCTTGCTCTTCTGGACCC
GGCTAAGCCGTTTCAACTGTTTGTGGATGAAAAGCAGGGAGTAGGGAAGGGAGTCCTGAC
GCAGCAATGGGGACCATGGAGACAGCCTGTGGCATATTTCTCTAAACGACTGGATCCAGT
AGCTGCGGGATGGCCACCCTGTCACCGTATCATCGCGGCCACTGCTCTCCTTGTCCATGA
TGCTGACAAGCTGACTTATGGACAGCGTCTCCTGGTCTACACGCCACACGCCATAGAGAA
AATCCTCAAACAGCCACCTAAAGAAAACCATACAGGATACAACCTGCACCTGCAAGGCCT
GTCAGATGATGAGACCAGGAAAAGGACAGCACACAGGTGTAAAATATCGGGGGGAAAGGC
CAGGACATCATTGGGAGATAGATTTTACAGAGGTAGACAGAATTGGACCCTGGATACATT
GCAACCATGTGCGACAGGCCACTCCAGAAGAACAAGAGAAAAAAAAAAAAAGAGGAAAAAA

AAAAAAGAACAAGAGAGGGCTCAAAGAAAATGGAAGTCAACACTGTATCCCTCCAATCCC
TTAAAACTGAAGCTCGTCCGACAGCAGATCTCAGACGGATCATTCTGATTCTCCTGATGA
TGGCTGTCCTCCTCGACCCCGGAACTGCCAGCACTGACCCACATCAACCTGTCAAAATCA
CCTGGAGACTTCAAAATGGGCTGATGTGAGAGGTACTCAATACGACCACTGAGATACATC
CTCCAAACACCTGGTGGCTGGACTTATATTTCAACCTCAAGGACTCAGTGGACACCCCTT
GGTCAGTGCCTTTGATCTGACTTAGAGGTTTTTGGGCGTGTCCTGGTCATAAAAGTGACA
GGGAAACTTGTGGTGGTATGCAACATTATTTCTGTAGGTCATGGAGCTGTGTGACTTCAA
ATGATGGGGTAAGGAGATGAAAAACTAGCAACCGAGATTTAGTCAACTTCTCCTTCACTC
AGCCCTGTGCCAAGAGATAAATTCTATAAATTCTATGATCAAGAAGATGAGATACCTAAA
GATGAGATTGCTCAGGTAAAATTAACGTTTAACCAGAAGTTGGCTATGCAGGAAAAGTCT
TGGGTGTCTGGATTATCTTGGGGCTTCCAGCTACAGGCTGATTGGTTCAGTGTAAAACCT
GGGGGAATCCTGATTATGAGTCAGATCATAGAACCTATCCAAACCCAAAGTGTGGGCCCT
AATCAGATTGAAAATCTGGGCCCACAAAGGGTGTTTAAGGCAGATCCGACTCAGAGGGCT
ACAGTTCTGAGCCCTACACCCTCCGTTTCCCGACCAGGAGACCAGCCTAATATAGCAGAG
ACACTAGACCCCTTGTCCCCCACCTCCAACCTAGAAATGTCAGACCCGTTGGTGGGAACT
AGCTAAAGTAGCCTTTAAAGCCCTAAACCATTCTAACCCCAGAGCAACTCAATCCTGTTG
GCTCTGCTACACTCTTCACCCTCCCTTTTATGAAGCAATAGGCTTAGATGTTTCTTACAA
CCTGTCCATCAGCTCAGGTCCACACCAATATCACTGGAAAAAATATAGGGTCGGTCTCAC
TATGAGGAAAGTTTGGGGAAGAAGACTTTGCACAGGTAAAGTTCCACTTGAAAAGACTCC
CTTGTTCGCCCGAACAGTCAATCTCACAGAACTAGACCAAGTAAAATGGATCGTACCAGC
GGTAGGGGGATGGTGGGTCTGCTCACATACCGGGCTGACTCCGTGTCTAGATGCATCGGT
TTTTAACCTGAATAGAGAGTTTTGTGTATTAGTGACTGTAATGCCCCAAATCTTCTATCA
CCCTGAAAAAGTTATGTATGACTGTTGGGCCAAGGACACTATAGATCAGTTAGGGAAAAG
CAGAGTTAAGAGAGAGAGCCTATCACAACCGTCATGCTAGCGAACATGTTCGGTCTCAGGAT
TGCTGGGCCAGGGACAAAAATAACATCCCTGACTATGCAAAATCAGGGATTCACTTCCCT
ACGAGCGGCAATAGATAAAGACATTATCGGTATAGAACAGTCAATCAGCCACCTTGAAGA
ATCCCTAACTTCACTGTCAGAAATGGTTTTGCAAAACAGAAGGGGCCTAGACTTGATCTC
CCTACACCAAGTCGGATTATGTGCGGCCTTGGGAGAAGAATGTTGCTTTTATGCCGACCA
CAATGGGGTGGAGAGAGAATCCATGGCAAAAGTGAGAAAAAGATTAGCACAGCAAAAGAG
AGAACCGGAAGCCCAGCAAGTGTGGTTCGAGTCCTGGTTCCATCAATCTCCTTGGCTAAC
AACACTAATTTCTACCCTACTTGGACGACTCATTATACTCCTACTCATTCTAACTTTTGG
CCCATGTATTTTAAATAAATTAATAACCTTTGTAAAAGAACAAATCAGTACCGTTCAGGT
AATGGCACTGAGGCAACAATATCAGGCAGTGTCTCAGAATGGAGAGGAAGATTCCTCTCA
AGGATAAAAAGACAGGGGGGAATGTTAGGATCAAACAGGCACCATGACAGGCTTCAGGGG
GCCAAGGTAGGCCTAAGTCTCCTTCACAGAGGGGCTGAGTTTTTGCCAAGGAGGAGCTGA
GATCCTCCTCTGCCAGTGCCAGGACTCAACCAATCAGTGTACTCTCTGTAGCCTGGTTCA
AGCTTATCAGGACGAGGATGAAACCCCCCTGAGAAAGCCTGCGCATGTGAAAATCTAGCC
AATTAGTAGACGCTAAGGAACCCTTGCAACCAATCCGCCCCTGCCAACTCCCTGTCTTTG
TCTTAACCCTATAAATACTGCTGTAATTCAGGGCTCGGGGCCCTTGTTCCACTCCACTGC
ATTGGATGAAACTTGGGCCCTGGCTCGAGCTAGCAATAAATTCCCTTTTTGCGTTTGCAT
TGCTGTAGATGTCTTGTTCTCTCAGTTCTGGGGATTGGGACCTTGGGCATAACA
>CAP_ERV_10
GGACTCTGAACTTTGTACCCTGGTCTATGGTAATGGCATACCAATGGAAAACCAGACCCC
TGGGATAAAAGAGCCTCAGGACTTGTACTTGGACTCTCCGTTGCCTAAAAGAATATGCTA
ATTATCTCTGTAACAGAACAAAGTCATTAATTCCATTATGTTCATTGGGATATGACCACA

GTCCTATTGATAAATGTCCACTATTTATCTAGTCTTGTGGCACATGAATCATGGGCTAAC
TTTGATCATATCTCTCTTTTACCTTGTCCAGACTAGTTTCAAGGAATTTGGGGAGGTGGG
TTTGAGCAAGTACACTTAGGGTATATAAGGTTTTCACAAAAACTGGTCGAGTCCTTAGCT
AAGAGGAGACTCTGCCTTGGCCCACCAGTGTAATAAAGTGCACTCCGCTATCTGCATTGT
CCTTCTGAGTGAGTTTGTTTCCCGGAACACGTGGCTACAATGTTTGGTACATGGGCCAGG
AATCTCCTCACTTTGAGGAGATAAGTCCCATTTACTACTCCGAGGCTTTGTGGCTCAGAT
CTTCTAGAAGGGGGAAGGCACCTCACCCTTTGGAAGGATTCTGCTTCTCAACGCCCGGAC
CTATCATGTTAGCAGGTAGTGGACAGCAGCAGGGGAACTGAACACTCAGGTGAGGAGGAA
CCCACCCGGCAGGGTGGAAGAGGGGGCCTGAGCATCCCCCTGGGAGGGACTAGAAGGGGC
ACGGACCCACAGGAGACTGGAATAGGCAGGCGGCGATTGCTTGGTACACAGGTCGATGAG
CCTGCTAGGGCTTAGGAAGTGAATTTGTAAAGGTCATTTAGGAGGTGTGTCCACGCCGAC
TTAGGGAAAATTATTACCAGCGATCGCCAGGAGATTTTTAGAATAGGAAACTGGTCCTTG
TATGCCTGTATTCTGCCTTTCCCCAGGAGGTGTCCCATCTGCTGTGAAATTCTTGACCCC
CTCGGAATTGTTAGGCTAGATAGGGGTATACATAAGTGAGTATGAATTGGCTTTTCCGGA
GATGGCCTGGGACATGGGATATTTAACCCATCTGTGTTTTCATCTGCACCTGATCAAGCC
CACCAAGGTAGAATGAACTTTAAGGGACAAACAATCTTGGACCACGTGGTGTTCTGGTTC
ATCTATGCTGACCAGCAGGTGAAGACACACCAATCCCCTTCTCCCTCTGGGACCTGGCAG
GTGAGGCTCTTCTTACCCCAGTTAGGAAGGAGGTAGAATGGCAATTTAAGTGTCACTGAG
TGGCAATACCAGAAGTACACATGGTACTGAGAACTTCATAGACAGAACTGCTGGGGTCCA
GCCCTGGTGGATCCAGGGAATTAGAAGCGGGGACAGCGTTGGCGAGGAAAACTTATTTAT
TTATTAATATAAGATTAGATTAGGAAGAAATAGTGTAGTAGGAAAATTAAGTGGAGAAAG
AGGGCTGAATAACTTGGATTACGTGGAAGACCAATAAAATTGCAGACAAGGAATCTGCAC
CATCTACGTTGGGCCACCGGCTCTTGCTTGAATATCTAAGGGTGCCTCGCCTTAGGCTCC
CTTTTGTGCGGGTTTTAACAGCCAGGGCAAGTAAGTAGACTTAGCGAGCCTCCGCGCCCC
AGATGGGAATTCAGCCTGAAATTTAAGCAAAGAGGAGAGGGAGGGAAAGGGTAAAAAGGA
GAGAGAGACACGGGGAAACCAGTCCAGCGACCTGCTCCAGAGCCCCTATTGTCTAGAAAG
GCCTTTTATACCTTTTTTTGTACATAGAGATCAATGGGTAACACAAAGTTATGCATCGTT
AGCAGTCCAGATTCTTATCAAAACGAGGCTTTTCTCTCTGCATACCTAATTGTATACACA
AGTCTTAGGTAATTTACATCATCTTCTGGCCAAAAGGGCCAATTAACATTTTACAGCCTT
TTTTCTGATAAGGGTTTGTCAACCAGAAGACTTATTTGCGTTGATCCACCCAAAGTCTGG
TGCCATTCTCAGAAAGCACTAAATAAAGTTACATTCTTACATAGCAAGGACACAAGAGGA
GTGCAGTGATATATAACAAAGAAAAAAGTAATTAACTCAAAAGTCTAGTGTTGCTAACAT
CAAAACTACTATATATCTTTTTTCAATCCCTTTTACATTGATTAACATCCTCCCAGGTGC
CTAAAAGATAGAGAATATGGAGGTCTGGCAGGCAATCATTAAGTCAACAGTGAAAACTCG
TCACCAATATGATTTTTAACTCTTTAGAAAAGGCTCTGTTTCTTTAAGATGCTTTTAAGC
TTTGTGCCTCTCACAGTTGGAGGGAGGGGGGGTGTAAACCATTCACAAGCTGTAAGAGGT
CCAGGGAACCTGTTAGGCAAGCTAGAGAGCTATCAGAGGGGGTTTAACTGAAACATCCCT
TTCAAATGCAGAAGACTAAAGCCCTGAGTTGACTTTTTTCCAGAAAATATCAGAAGAGTG
GAAAAGCAGAGTACAAAAGCCGGCAGATTTTTGTTGTTTTTTTTGTTGTTGGGTTACATG
CTCAGGAAATTCCAGGGGGACCCCTGAAGTCTGATCACGACCTTGCGTATGTCAGCTTCC
TTCCTCATGACCTTGTCACGGGCGGAATTCCTCACACTGGCTTCCCGCACAGAACGAAAA
GGAAAAGTAGAAGAAGGCCCGAGAAGGTAAGCAAGATGAGGGGAAGTGAATCTAGGACAA
CTGTACTGGAGTGCAGGATTAAAAATTTAGAGAAGGGATTTGGAGGAGACTATGGGGTGA
AGATGACGCCTAACCGCCTTCACATACTCTGTGAGGTGGAATGGCCCCCTATGGGAGTAG
GACGGCCGCCAGAGGACACCATGAACTTAAAAATAGAGGAAGCAGTCTATACAGCAGTCA

CAGGAGAGCCAGGACACCCAGATCAATATCCATATAGGGACTCATGGCTGGGTTTAGCTC
AAGACCCTCCTACTTGGACAAGGTTCTGTATCCAGGAGGGAAAGGGAAAAATATCAATGG
CACAAAAATTGACTGATGATAGAAAAGGAAATTCTATGGGATGTGGACGGGGATGAACTG
ACCCCTCCCCCGTACTGGACAATGACGCGCCCACTGGCCCAGTGCTCGACCAGGATCGGA
GGCTGCCTTAATGCCCGATCCAGGGCCAGGTGAAGTCCTGCAGCAGCCGCTGCTCCTCCG
CCAGCTCTCCCCGAGGTCACAGAGCCACCACCTTGGCAGGCTCCGATCCTAGTGACAGAA
GCCTCTGGCCAGCACTTCCAGGATCCGGCTTCAGCCAGACCGCCCAAGCTATACCCACCT
CTCCTGGTGAGTACTGACAAGAAGGGGAGGGAGACATTGGAATTAAGCAGAGACTGCGCT
CTCCCAGAGCACCGGAGGGAACCAAAGAGAACAGACAAGAGATTTGCAATGCTAGCTATC
TATCTCGGGCCCTCCGTTAAACCTCCTTTGCCCCGGGGACAGGACAGTCCTTCAACTGGT
CCCAACAGAGACCCCAGGCCCAAAACCATTGTGCCCAGTGCCGAGCTTTTGGCCATTGGA
AGAATGAATGCCCTAAGGTAGGGAAAGAAGAGGAAGCTCCCCCAGTTGTGGGGCTTGCTG
ACTTGGAAATTAAATAGGGCTGCCGGGGCTCAGAGATACCAGGTCCCGGAGAGCCCATGG
TAACCTTAAAAGCAGGGGACCAAAACATTGACTTCATGGAGGATACAGGAGCAGAACTGT
CAGTAGTAAAACCTGTGGCACCACTGTCCAAAAAGACTACCGCTGTAACTGGGGTATCGG
GAGAAGAGAGGATTAAATCGTTTTGCCAGCCCAGAAAATGTCAGATGGGGGGCACCAAG
TGATTCATGAATTCCTCTACATTCCTGAGTGCCCAGTACCCCTGTTGGGAAGAGACTTGT
TCTCCAAACTAGGAGCACAAGTGACTTTCTCCCCTGAGGAGAGGCCCACCTTCTGGATGG
ACACTTCGACTTATTTGCTCTCTTTCTCAAGAGCCCCCCAAGATGAGTGGAGGTTGAATG
AGCCTCTGAAGGAAGAACCGGGTGGGCCGGAAGAGCAAGAGATAGAGCTAACTCAATTAT
TCCCCGAGGTCTGGGCTAAAGACAAGCCCCCTGCCCCCTAGTCTGGCTAAACATCAAGCC
CCAGTGATAATAGAACACAAACCAGGCACCATCCTGTTTAGAAAGCACCAGTACCCGCTA
CCGATAGACGCATGGGCCGGCATACTGCCCCACATCAATAGACTGAAACAGGCGGGCATT
CTAGTAGAGTGCCAATCGGCTTGGAATACGCCAATCCTGCCAGTCAGAAAGGAAGGAGGA
CAGGGCTATAGGCCTGTACAGGATCTCAGGCTAGTCAACCAGGCTGCTGTGACTTTACAC
CCCACTGTTCCAAACCCCTGTACCTTACTGAGCCTCCTCCCACCGAGGACTCAAGTTTAT
ACCCGCCTGGATCTCAAGGATGGCTTCTTCTGCGTATGCCTCGCCCCAGCGTCACAGCCC
ATCTTTGCCTTTGAATGGGAAGATCCATTGGGGGCACCAAACCACAGCTCACCTGGACTC
CCCCACAGGGGTTTAAGAACTCCCCAGCCATCTTCAGGGAAGCCTTGGCTTCTGGCCTGG
ACTCATTCCATCTGGAAGAGTATGGATGTTGGCTCCTACAATATGGGGATGGCCTGCTGC
TGGCCGCTGAGACCAAGGAAAAGTGCTGGGAAGGGACAAAAGCACTGCTCCAGCTGCTGA
TGGAAGCAGGTTCCCGGGTGTTGAAGAAGGAGGCACAGATCCGCAGGGAGGAGGTAAGGT
CGCTGGGGTTTGTTTTAAGGAAGGACACAAGGGTCCAGACCCTAGTTGGGTCCTATACAC
TGATGGCACCAGCCTGATACAACAAGGACAACAGCCGTCAGGTTAGCCAAAGCGGAAGGG
CCGTCAAGACTGAAAAGGGGTGGTGGGAATTGCCAAGTGGCAAATTATTGGTACCAAAGG
AGCTGGCACACACTCTGGTAGCCAAACACACCAAGCGACCCGCCTAGGCCAGGCTGCCTG
CTCACAGGTTAACGCTGCCTCTCGGCTATTCAGACAAAAACCTCCGGGCATTCAGCTGAA
AGGCACGCTGCCCTTTGAACACCTGGGAGTGGACTTCACTGAAATGAAACCTCACCGACA
CTACTGTTACCTGCTGGTCAAGGTGTGTACGTTCTCAGGATGCGTAGAAGCTTTTCCTAC
CTGGACTGAAGGAGCATCAGAAGTAGCCCACTGCCTGCTTAGGGAAATAGTTCCCAGATT
TGGACTTCCTACCAGCATTGGTTCAGACAATGGTCCGGCTTAAAGTAGCTGATTTAGTAC
AACAAGTAAACAAAACTTTACACATCAAATGGAAACCGCACACTGAATATAGGCCCAGAG
ACGGTGGAATGAACCAACCGGACATTAGAGAGACTCTCCAAGTGGATCATAGAGACTGAC
TGCTCCTGGGTGGACTGGCTTCCGACGGCTCTGCTCAGACTCAGGATGACCCCACAGTCC
CAAGGCTATTCTCCATACGAAATTGTGTATGGGAGGCTCCCTCCCATAATAATACAGGTG

TCAACAAATTTGCCTCAGGTAAGGGGGGATAGGATTTCACAGCAGATGGAACTGGGTAAG
GTAATAAATCGGGTAACTAAGTTTGTACAAGAAAGGGTGCTGTTCCCCCTTGGGGAACAG
ATTCATGAGTTTACGCTTGGTGACCAAGTACGGGTCAAATATTGGACACATGATCTGCTA
GCCCCTTGGTGAAAGGGCCCTCATGTTATTCTAACTACCCCTACTGCAGTTAAAGTGGCA
GGTACTGCCCCTTGGATCCATCATACGAGGGTGAAGAGGACATACCGCGCAGACCCAAAA
AACGCTGAGTGGACTGCACAAAGGGACCCCGCTGACCCTCGAGAGACTAAGACCATCCTT
AAGAAGAAGGGAAAGAAGACCCTGGACGAGCCCCTTCAGGATAAAGCTGCACAATCAGCT
CCTGCTGCTTGGCTCATCAACGTGATTTTGATTTTAACTTCCCTTTCAACTCAGGACAAA
GTTTTCATCTCATGGGCACATTCCTACGCAGACTTCCGCAACACTTCCAGCTGCTGGTAT
GTGGGGCTATGCCTCTGACAGTGATGGAGGGTCTTCCTTGGTGGGTGTCACCGCTCTGCC
AAGGAGATTTTAAACCACTCTGCTCTTTTCTGGGATGACAAAAAGAGACTTTCCTCTCTC
TTGTCAATCATAACCTCTCCTTGCTCTCTTGGTGTAAGACCTACAGTCAGTCAATAGACT
CGGGTCATGGGGTTACATTTGACACAAATGCCAGTGTAACAAAAGACTAACCTACAACAA
GCCCCGGTAAATCTACCTGATTTACATGCTAGGTGGACAAGATCTGTGTTTCAATGGTCC
CTTATTCGTACCCTCTATCGGGACAACAGATATTATGATTAAAGTAGAGGCCTTGACTAA
TTTTACAAAACAGGCCCTCCTAGATAGAACAATAGCCATCCAAGCCTTAAATGAAAGCAA
ATCCAAAATGAGAAAAGCGGTAATTCATAATGGAATGGCTTTGGACATACTCACAGCTGC
TCAAGGAGGGACCTGTGCCATAATTAAGGTTGAATGTTGTGTATACAGTCCTGACTTATC
TGGCAATTTTCGCCTGCTTTAGATGACATGAAAAACCAGGTACAAGCAATGTCAAATGAA
AACCTTCCTTTCTGGACTTCGGTCCTATCTTGGGTGAAGGGCGATTGGTGGAAAACTATA
TTAGCCACTGTTGTAGTTGCCTTGATAGTTCTGCTTTGTGGACCCTGAATTTTACAATGT
ATTATGAACTTTGTAACCCAAAGGTTGATGTCATTCTCCCAAATTGGCGGTCGGAGAGCC
AGGGTGCAATATATCCCTATGAGTGATGCTCATAATATGAGTTAAGAGCATCAAGAGGGG
GGAATGAAGGAGGAAACAGACAGAGCTGGACTCTGCCTCAGGCCAGGCTGCGAACATTAG
GCTACACGCATGGTTCCCCTCCACCATAGGACTCTGAACTGTGTGCCAGGCCTAGAGAAA
TGGCATACCAATGGGAAACCAGATCGCCCTGGATAAAAGAGCCTCAGGACTTGTACTTAA
CTCTCCATTGCCTAAAAGAATATGCTAATTATCTCTGTAATAGAACAAAGTCATAAATTC
CATTATGTTTATCGGATTATGACCACAGTCCTATTGATAAATATCCACTGTTTATCTAGT
CTTGTGACACATGAATCATGGGCTAACTTTGATCAATCTCTCTTTTACCTTGTCCAGACT
AGTTTCAAGGAATTTGGGGAGGTGGGTTTGAGCGAGTGCACTTAGGGTATATAAGGTTTT
CACAAAAACTGGTCGAGTCCTCGGCTAAGAGGAGACTCTGCCTGGGCC
>CAP_ERV_11
CAGTCACCACTTTTACTTCTGTGATCTTGCTTGCTCCTTACATCCATGGGAATTAGGGCT
GCTGCGGAGGGGAGGGATCTGAGCTGTTTGAGTGGGAAGTTTACAGTGTAGGAAAGATAT
CCAGAACAAAGCCGGGGCTCCCAGCCTTTGGAGGTGACTCCGCTGGGCCTGCACCAGTGC
TGAATAAACCCTGCTGTTCTGTATCTCTGAGTGATTCTTGTGTCTTTCCGACACTATGGT
TTCTATAATATTTTCTGCTGCTTTGGCCAGGAAGCTGACAACCGAATTGGCCCCTTGCAC
CACCATTGTCAGGGAACCAGAGAAAGCTGTGGCCGCCGACCTGGTGATAGACGGATAGGC
ACCACCCGGCCATTTGCTGGAGCCCGATTCTCACTCAAGTCGCGCTGGCCTTGGCCCATG
CTGCTTCCCCAACAGACTCCGAAGGAGAGCAGAAGCGGGTTCCAGTGCAGGACGTCAGAT
GGGTGAGTGCCCTGGGGACATCTGCCTGAGTCAGGACCCACCATATGGCAGAAGAGGCAC
CTGATCACCTCCCAGTGACCAGAGAGTCACTCAGAATAGGGTATGTTCCCTCGGGTGGTC
TGTCAGTTCTGGTGTGTGTGGGAGAATGATTGGGCGGAGTGAAAGCCAGCGCTCCACCGT
CTTTGGACTATGGAGCCTGAGAGGGTCCTAACCTGTGGTTCCACAATGAGCTCATTCAGT
TCAGGGCAGTGCCAGCCTCTGGGGGATTTAATCTGTCCTTGCGAGGCTGGTCTGCGTTGG

GAGTTTATATTGACCTGCCCATGCTAGGTGGTATCTAAATTTCCATATGGGGAGCAGCCA
GATGGATGAAAATGAGTGTCTTTGTTTTGTTGTGGGACACCACCTCAGGGTGGGAACTTT
CACATAACGGGCAACTCGATTTCTAAACTCACTATCTTAGAGTGTATGGTTAAAAATTTT
AAGAAAGGATTTTTCTGGGGATCAGGCTTCCCTCATAGCTCAGTTGGTAAAGAATCTGCC
TGCAATGCAGGAGACCCCAATTCTCTGCCTGGGTTGGGAAGATCTGCTGGAGAAGGGATA
GGCTACCCACTCCAGTATTCTTGGGCTTCCCTTGTGGCTCAGCTGGTGAAGAATCTGCCT
GCAATGCAGGAGACCTGGGTTCATATCCCTGGGTTGGGAAGAGCCCCTGGAGAAGGGAAA
GGCTACCCACTCCAGTAATCTGGCCTGGAGAATTCCATGGACTGTACAGTCCATGGGGAT
CGAAAAGAGTCAGACATGACTAAGTGACTTTAACTTTTCCTGGAGATTATGGTGTAAAAT
TGAGTCCTGAAAAACTTTACAGTCTTTGTACCCTGGCGTAGCCCTCCTTTGGGGTCGGTG
ACCCCCAGAAGGTGCCTTAGATATACCCACTGTCCGAGTAGTGTCATAGCGGGAGACTCC
AGCCACCTGCACCAATTCCTATACACCAATTAATGGTTGGAAATTGCCCAGTGAGGGCCC
CCTTGGGTTCGATTCTATGTCTCAGGTCAGGGATAGTGTAAAGTCCTAGTAGTTCAATCA
AAGAAAAGGATCTCGTGCCAAGAGAAGGAACCACCTATCCTCCAGGGAGAGGCTGAAGAG
CTGCCTCCAGTGTCCCCACCTTACGTCCCCACGGCTCCTGCACCCAGAGCAGCAGATCCC
CATTTACCAGACAGCCCACCGCCTTTCATTCCTTCTCCTCCTGACGTTCCTGAAGCTGTT
GTGCCCCCTCGGCCAGCCATCCCAGAACCAATGGGTAGGCGCCTCTGGTCGGCTCAGGGA
GCAGCTGACCCAGCACTACAAATGCCCTTACCAGAGACTCAAGGGCTGCAGCACTTTGCC
ACCGATAGGATCCTACATGAGGGAGGACCCGTTTTTACTACCAGCCTTTTTCCATGACT
GATTCCCTTAGCTGGAAACATCACACCACCTCTGCTCTGAAAAGCCCCAAGGGCTGGTTG
ATCTCTTAGAATCCACCTTCCAGATCCACCACCCTTCCTGAGGAACCCGCCAACAGCTTC
TCCTCACCCTATTCAATACAGAGGAGCACCGAAGTCACGACAGAGGCTGGGAAGCGGCTC
TGGGCCAATGCCCCAGGGGGCCAGTTAGGCGCAGAGAGCTGGGATCGGGAGAATTTCCCG
GAGGAAGAGCCTTGCTGGGCCCCAATACCGAGGGAGGGGAATGAACCAGTTGGAGAGGTA
TCGACAGGCCCTCCTACAGGGACTAAGAGCCAGGGTTAAGAAGCCCACTAACATGGCTAA
GACCAGTGAGGTGCTACAAAAGCCAGATGAAAGCCCGGCCGATTTCTATAAAAGGCTATG
TGAAGCCTTTCGGGTCTTCACCATTTTGATCCTGAAGCCCCTGAATATCAACGGTTGATA
AATGCAGCCTTTGTAGGACAGGCCCAATATGACATCCAGAAAAAAGTTACAGAAGTTAGA
AGGGTCTGCAGGTAAAAATGCCACAGAACTGCTAGAAATAGCTAACAAAGTCTTTGTCAA
TCGCAACCAAAAAGCACGCCGAGAAGCAGAAAAGAGGATGAAGCAAAAGCGGCACTCTTG
GCAGCTGCCCTGTCAAAGCCTTCCCCCACCCCACGCCCCTACCTTGGGACCACCCCATAA
GGCAAGGGGCCCAGATCCGAAAGGGAGCGTTCCCCTCAGCCGTGATCACTGTGACTATTG
CAAGGAAAAGGGACACTGGAAAAATGAGTGCCCTAGCACCCTGAGAAGAAAACCAAAGCC
CCAGGCTCCCTGAGCTGATATCAAACCAAGCCACCCAGCGCTAATCTGATTGGGCTAGCT
GGAGCAAAATCCGACTGGGGGGACCGGGCTCTCTCCAACTGCGCCCCCAAGAGCCCATGG
TCAGAATTCAAGTAGGGGGCCATCCTATGGACTTTATGGAGGACACAAGTGATGAGCATT
CAGCGGTCACCCAGCAGACAGCCCCCTCTCGGGAAAAGAAGCTACCATCCTCGGGGCCGC
AGGTGCCCAGACCCACAGGCCATTCTGCAGTCCCTGACAGTGCCTCCTAGGGGACCATGT
GATAATGCACGAATTCCTCTACCTGCTCAACTGCCCCGTTCCCTTGTTGGCAAGAGAGCT
GTTTGCAGAGATGGGGGCCCAGATTTCCTTTTCTGCTGATGGATCACCTCAGCTAAAGCT
AGCCCCACCGCCTTCCTCCTTGATTACGACTCTCATTATAAGGAGAGAAGAAGAATGGCA
CCTGTATTCCTCCCCTCCAGGAGAAGGGACCATTCCTCCTGAATTAGAGACTGAGTGCCC
GCTAGTTTGGGTGGAAGAAAATCCTCTGGTTCTGGCAAAACACCATGCTCCCATCCTGGT
TGATCTGAAACTGGGAGCTCAGCCTGTAAAACTACAACAGTATCCAATTTCCCGGGAGGC
CTGACTGGGAATCCAGACTTATTCAGACAGACTACTCCAACACAGACTTCTGATGAAATG

CCAGTCACCCTGGAACACCCTGGAACACCCCATTACTACCTGTGAAGAAGCCAGGGACTC
ATGACTACCATCTAGTTAAGGACTTAAGAGCTGTGAATGAGGCAGCGATTACTCTGCATC
TGGCAGTGCTTAATCTGTACACCCTACTAGCACTGATCCCGTCCCCAGCTGAATGGTTTA
CTTGCTTGGACCTGAAAGATGCCTTCTTCTGCCTCTGGGTTGCACCTGTCAGCCAATCGC
TATTTGCATTTAAATGGGAAAACCCACACATGGGAGACAAGGAACAGCTCACTTGGACGC
GGCTTCCTCAGGGGTTCAAGAACTCCTCTACCCTCTTCAGCAGAGCTTTGACTGTCAACT
TGGCTAACTTTCCTGGACAGGAGTTAGACTGTGTCCTGCTGCAATATGTCAATGACCTCA
CGCTGGCCAGGACAACACAGGCTTGCTGCCCAGAAGGAACAAAGGCCCTCCTCTCCTTGT
TAATAGAAGCTGGATACCAGGTATCAAAAGAAAAAGGCACTCCTGAGAGACATTGTCCCC
AGATGTGGAATGCCTCTGACCATAGAGTCAGACAATGGACAGGCATTTGTAGCCGAGACA
GTACAATAGACGGCAAGGGCTTTGAAGATTCAGCGGAAATTACATACTGCCTACCTACCC
CAGCGCAGAAGGTGATGACACCCCACTCCAGTGCTCTTGCCTGGAAAATCCCTTGGATGG
AGGAGCCTGGAAGGCTGCAGTCCATGGAGTCGCTGAGGGTCGGACACAACTGAGCGACTT
TACTTTCACTTTTCACTTTCATGCATTGGAGAAGGAAATGGCAACCCACTCCAGTGTTCT
TGCCTGGAGAATCCCAGGGACAGGGAAGCCTGATGGGCTGCCATCTATGGGGTCACACAG
AGTCGGACATGACTGAAGTGACTTAGCAGCAGTACCTGCCCCAGAGCTCAGGGAAAGTGG
AATGCGAGAATCGAACCCTCAAACAAACCCTAGCAAAACTATGCCAAGGGAACAGCTTAC
CTTGGGTAGGCATGTTACCCATGGCCCTCTTGAAGGTGAGGTATCCACCCTGGGCAGGGA
TAGGGTTTTCACCATTAGAAATCCTGTATGGATGGCCACCCCAGCTAGTCAACCTAAGGG
GAGACACCAGAGAACTGGGGAACTTAGACGTATCTAGGCAGCTGCAAGGGTTATGACACA
CAATCTCTCAAATACATACGTGGAGAGTTGATAGAATACCAACCCTTAGGCATAGCAGTA
CATCCCCACCAACCAGGGGACCAAGCATGGGTGAAGGACTGGAAAAAAAGAGCCTTTAGA
ACCCTTGTGGAAAGGGCCCTATTCTATAATCTTAACTGCTCACTGGTCTCAAGGTGACAG
GAATAGACGCTTGGATCCATTACTCCAGAGTCAAACCTACAAGTCTGACCAATAGCTGAG
GGGAGTGGGAAGCAGCCCTCAGTCTGGAGGAGCCCCTGGGCTTGATCTTGTGAAGGAGGA
AACAGCCACGCCGAAGCCCTGCTCGGACCACACTGGAAGCTGGTCAGTCAACGCATGGCT
GAAGCGTGAGGAACCCACTGGACTGACAAAGGCAGATATATTGGGTATTAACTGGACTGG
GGCTCGCCCTGCTCATCTTATGCAGAATAAAGCTTTACTTTTTCTCTCCCCTGGAGTGGA
CTTGTATCCAAAAACTCTCCCTCTGTGTTGTCTGCTTCTTCATAGTTCTTGATTTTTGGA
GTTTTCTCCTACTGTGATCAACTTAAATATGAAAATGACGTATTTTTGCCTCATAGTCCT
GTTAAGTTGTAAAATCCCCTCCTCATCTGAGAGTCAGGATGATTGTTCGGAATGTATAGA
ATCTTTCTATTATGAGAAAGGGTGGGTCTGCTGTGCCTTTGCTTATTATAAGCACCAGTT
GACCGCATGTCATAGTTCAGCCACTACACGCACTAGAGAAGGGAAACATTGTTGGAAGGG
AGGAATTAAAGCCCAAAATATAGATGGCCAAAGATGTTACAGGTTTCCAGGACAAGCAAC
TTGTTGGAACCTAGGCAAAGAGAAAACAAAAATTGACGTCGACCTAGGAAAATGGCCTAT
TGAGTATATCTGGCTGGACCCCGACCCCTTAGTATCCACCCCTGGCCCACCGACATTAAG
CCCTGGGCCAGGATTCAAGGGACTATATGATCAACTCATTTCCCTAAAAGATGAACCAAA
AGTGCCTACTGTTGAAAATAATCTCTTTATCCATTTAGCCGAGCAGGTTACCTGAGAATT
GGGTATCACTAACTGCTGGGTTTGTGGAGGAGCCCTGATGAGTGAAACATGGCCCTGGAA
GGGCACCAGACTGGACGCCTTTCTGCTACTGCAATGGGATCAAACAATTAGTAGGCAGGC
AGGAGGTCCTATTTGGGGTTGGCCCTGTCCTCAGAGACGATAGGCAAAAAATGCTTGAGC
CGAGAAAGGATGTTATACCTGGATCAGAAAAACAAAATGCAGAGGAACCCTGGTCTATAG
TGACACCAGTAACAGCCTTTTGTGGTGACCAGGGGCCCCAAGCTGGTATTGGGCTCCCCA
AAAAGGAGGGTATAGAAATCACACTTGCATTCCCTTCAACTCCACCACCAGGATTCTAGT
GTAAAACTGTACAGGAAACCAACCTGGTATTAATCCCTTTCTTGCAATCCCAGGCATTAG

CCCATATTGGAAACATCTTAAATCCACAAACCCAGACTTATGGCAGGCCCCAGAGGGTCT
CCTTTAGATCTGTGGGAGAAGGGCCTACTCATGACTCCCATCCAGATAGGGAGGGGAGGA
ACCTGCACCATAGGAATGATTCAGCCAGATTTTTTCCTCCTACTTGGCTCACAAGGAGAC
ACGCTTGGTGTCCCAATCTATGATGACTTAAAATGTTGAGAAAGGCGGTCTCTAGAGATA
GGAGGCCATCACCCCGAAAATGACTGGCCTCCCCAGTGAATACTCGATATTATGGTCCAG
CCACAAAGGCACAAGATGGGAGTTGGGGATACAGGACACCCATTTATGTGCTAAATCGAA
TAATCTGACTACAAGTGATGCTACAGATTATAACCAATGAAACTGCTGCTGCCTTGGAAC
TGCTGGCACAGCAACAATCCCAGATGAAAACTGCCATCATTCAAGATCACTTGGCACTAG
ACTACCTCCTAGCAGAGGAAGGAGGAGTCTGTGGGAAATTCAGTCAAACTGACTGTTACC
TCCAAACAGATGATAATGGAAAAGCTGTGATAGACATAGCTAAACATAGCAGGAAGATTG
CCCATGTTCAGTTCAGACCTGGAAGGGATGGGATTCAGATGGCCTCTTTGGAGGATGGTT
CTCTTGGCACGGGGGGTTTAAAACAATGATTGGGATGGTCATAGTCATACTTGCTGGAGG
TCTGCTTATTCCCTGCCTCATATCCCTCCTAATCAGAGTTGTAACAGGGTTCATAGAGGC
AGTTGTCCCACGGAGGACGGCCACCCAGTTGCTGCTTTTAAAGGAATACCAGCAAGTGCC
AGAAGATGATGTTCTGTGACAGCTGCAGATGCATCCTAAGCATCAAGATGGGGGGATGAG
GTAGGAACGAAATCAAAACCCCCAATTAAAGTGACAGTTGCTTGCTTTCCCTTCCCTTAT
CTTGTTTTTAAACAGCTGCTGCTCATCAATCACCACTTTCACTTCTGTGATCTTGCTTGC
TCCTTACATCCATGGGAATTAGGGCTGCTGGGGAGGGGAGGGATATGAGCTGTTTGAGTG
GGAAGTTTACGGTGTAGGAAAGATACCCAGAGCAAAGCGGGGGCGCCCAGCCTTGG
>CAP_ERV_12
CAGTATGTTATACTCCGATATCGTTCCTCTAATCTATGTAAAGGACACTATTTGTATGGT
GCTCTGCCCTTCTTCAAGATTCAAGCTAATCCTTTTATGGCCCAAGATGAACCATTTGGA
GCCAAGATTATCCCAAACTACATCCTATGGATGAGGGGCCTGGTTCCATTCTAAATTTTG
AGACATTTCTTTCTTTCATTAACAGACTGCTGGTGACTATATAACATCCAGCTGAAGACT
AGCAGGGGGGTACTCTTTCTGCCCCCTGCTGATGCCTATGTCAGAAGCTTTCTCTATCTC
TTTTATGCTTTAATAAAACTTTGGAACCCAAAAGCTCTGATCGATCAAGCCTCGTCTCTG
GCCCCGGATTTTATTCTTCTCCTCTGGGGACCAAGAATCCTGGCGTCGTAATTCAACAAC
AACCTTTCATCTTGGGGACTCGTCCGGGATCCTTCAGGACAAGGTAAGGATGCTTGGAGC
TCTAGTTCTTTGTTCTCTTAGCAAACACATTTTCTGCTGTGCTTTACTAACTCTACGGTG
TGCTTGTGTGAATGAATGACATGCCCTGCGCGAGGCAAGTGAGGAGCCCTGCTCTGCGGT
TCCACGGTGATCTCATACGGCTTATGGCAGAAACCTGTCGGGGGTTATACCGACCTGCCA
AGGCCAAGAGGCACCCAGTGTCTCCTTTGGGAACCGACCAGAAATGGGCAAAGCGTGTGG
ACCGAACTCTCCTTTCTCGGTCAAACTTTCCGGTCTCTTTGACCTTTTCATAACTCCTTG
GGAATTAGAAGTACTAACCTAATCTATCGGATCATAGACTTTCCAGGGACTTGTGATCTA
TACTGTTACTGTGTACTGTGGCAAACTTGGATTGGTAGTCAAGAAAGCGCCTAGCCTCGC
TAGGAATCACAAGCTCAGAAGCTAGATGGAGCTCTGGCCCCAAGAACATCTCCGAGGTTG
AAGGTTACTCAGATTGGGACTGCAATGGGTTTTTTTCCTTTGGTAACGCTGGCTCTTAGT
GGACCAGAGGAGGCTCTCATACTGGTGTGGTGATGCTTGCAAAGAACATCCCAGCTTGAT
GTTCGTATCGGTCTTATTGTGGTCAGGACTCAGGGTCGTGCACAGGCACTCAGGTGATGA
ATGTTTCCCCCAGCGGCCTTAGCCTGGGAGGCATTCCGGAAGGTGACTCTGATTGCACCC
CGGGTGGCATCAGAGGCAAACAAGGTTAAAGGTGAAGAGCTGGACATCAGGTAGAGATGC
TAGCAGGTCTCCCCCTGGTACATCCCCACCCCGTCTCGGTGGTAGAACCGAGAGGGTCAA
GACGGCACTGCATCGGTAAGGGAGAGACTAAGTCCGACCAGGAAGGAAAAGCTTTTGGTG
TAAAGTCTGTCTACACCCCCATCTAGAGCAGGGAGGGACGCCTCCGGTAGAAAAATGGCA
CTGGTCGCTTTTTTCTCTCTTACAGATGGGAGCTAACAATGCCAGCCTCACTCCTTTGAA

CTGTATCCTGAAAAACACTGGGATAGATTCGATCCCCAGGCCTTAAAGAAGACACACCTG
GTCTTCCTATGCGATACTGCATGGCCACGGTATCCATTGGAGGATGGCGAACGGTGGCCA
GTTGGAGGGTCTCTTAAGTATAATACTGTTTTACAATTAGACCGGTTCTGTAAGGAACAA
AGGAAAAGTGATCTTAAAAGATAGATTTATCACTCACTTGGCTCCAGATATCCTCCATAA
GCTACAAAAATGGGCGAATGGACCAAATCAGTCTTTAGATACTCTGTTACAACTGGCTTA
GATGGTCTATTACGGTAGGGAATATGAGGAAAAGAAAGAAAGGCAAAGAAAGACAAAGGA
AAAGGCGGAAGCCTTCGCCATGGCTATGGACCCAGGTGAAAAGAGAAGGGCTTGCTATTA
CTGCGGAAAGGAGGGGCACCTCAAGCGGGATTGCCCTCAGGCATCTAAGCCGGCCCGGCT
CCAAGTCCGGTCTGCAAAGGACCACACTGGAAGAGAGACTGCCCTCAGAGGCGTAGGTCT
CCGGGGTCGGACTCTCAAGACAATCAGGACTGAAGGTGCCCGGGGGTCCCCACACAAGCT
CCCGTCCTAATTACACCTGAGGAACCCTGGGTATTAATAATTGTGGGGGGCCAATCCGTC
GATTTCCTTCTAGATACTGGGGCAACTTACTCTGTGCTTACTGAAGCCCCTGGCCCACTT
TCTTCCCGATCCGCTTCCGTAATGGGACTGTCTGGATGAGCCAAAAGGTATTATTTCAGT
TATTCTTTATCTTGCACCTGGGATTCTGTGCTGTTTTCACACAAGTTTCTGATCATGCCA
GAATCTCCCTCACCCCTTTTGGGGAGGGATATACTGAGCAAGGTCCATGCCTCTGTTTTC
ATGAATATGGAGCCCTTTCTTTCTCTACCTTTAATAGAACAAAATGTAAATCCTAGAGTC
TGGGCTGATGGAAAATCTGTGGGTCGAGCACAAAATGCTATTCCTGTAGTTGTCAAGCTC
AAAGACCCACACTTATTTCCACATAAGAAGCAGTATCCTCTGAAACCTGAGGTTAAGGAA
GGGTTAAAACCCATCAATGAAAATTTAAAGGAACAGGGACTATTAATTCCCTGTAACAGT
CCTTGCAACACTCCTATTTTGGGTATAAAGAAATCAAGTGGTAAATGGAGACTTGTCCAA
GATTTACGTATAATAAATGAGGTTGTAGTTCCTTTACACCCCGTGGTGCCTAATCCTTAT
ACTCTATTGTCTGAAATTCCTGAATGAGCCAAATATTTCTCAGTAATTGATTTAAAAGAT
GTCTTCTATTTAGTGCCTTTGGCAGAGAAAAGTCAATTTCTATTTGCCTTTGAAGACCCT
ACGCAGCCAGCTTCTCAGTTAACCTGGACAGTTTTGCCCCAGGGATTTCATGACAGTCCT
CACTTATTTGGACAAAGTTTGTCACGGGATCTACAAAACTTTAACAGCTCTGAAGCAGTG
GTGTTACAATATGTAGATGATATTTTGCTCTGTGCTGAGACAGAGGAAGCTTGTTCGCGA
GCCTCAGAAGATTTCTTAAACTTTCTGGCAGGCTGTGGTTACAAGGCATCAAGAGAAAAG
GCTCAGCTTTGTCAACAATCAGTTAGATATCTGGGCCTAATCATATCAGAAGGGACTAGG
GCCATAGGCCCTGAGAGAATTAAGCCTATACTAAATCATCCCCTACCTATGACTTTAAGA
CAATTGAGAGGATTTTTGGGAATCACAGGTTACTGTCACATTTGGATTCCGGGTTATGGG
GAACTTGCCCTGCCTTTATATAAACTTATAGCTGAAACTAACTCAGCAGGCCCAAACTGA
CAAACTGGTTTGGTCACCAGAAACTCAAAAGGCTTTTAAGGTTCTTCAGACTGCTCTCCT
GCAAGCTCCTGCTCTGAGCTTGCCCACAGGGTCAGAACTTAATCTGCAGCTGAAAGGAAA
GGTGTGGCCTTGGGAGTTTTGACACAACCCCGAGGGCCTCACCAGCAACCTATTTCTTAT
CTAAGCAGAGAATTAGATGTAATTTCACCTGGGTGGCCCCACTGCCTAAGAGTAATTGGG
GCAGCGGCTTTATTAGCACCTGAAGCTTTAAAAATAATTATTGGATGAAACCTTACTGTA
CTGACTTCTCATGATGTGAGTGGAATCTTAAATTCTAAGGTTAATATTTGGATGACAGAC
AGTAGGCTTCTTAAATATCAGTCATTGTTGTTAAAAGGACCAGTAACTAAGCTTAAAGTT
CGTGGAAATTTAAATCCAGCCGCTTTCCTTCCTGAGAAGGAAAATAAAACACCTGATCAC
GATTGTTCTCAATTCCTAACTTTAAACTATGCAGCTCGGGAAGATCTAATGGATACCCCA
TTAGACAATCCTGACCTGAAAATATTTACAGATGGCAGTTCTTTTGTTCAAGATGGAAAG
CGTAAAGCAGGTTATGCCGTGGCGACTACTGAACAGGTTTTGGAAGCAAAATCTCTCCCC
CAGGGAACCGGTGCTCAGTTAGCAGAGCTTGTGGCTCTGACCCGAGCTCTAGAGTTAAGC
AAAGGGCAGCAGGTAAATATCTACACAGATTCTAAATATGCTTATTTGACTTTACATGCT
CATGCTGCAATATGAAAAGAAAGTTTAAAACAGCAACAGGTGAACCTATTAAGCATTTCA

GAGAGATCGATGCTATATATTGTCCTAAAGAAGTAGCTGTTATGCATTGCAAAGGGCACA
GCAGGGATGGGAGTAAAGTAGCTGAAGGTAATCAGTTGGCTGACTCTCAAGCCAGAAAAG
CGGCACTTTACGGAAACCCCTTCACTGCAGACGCCTTTGATCTGGACAGGTTCTGTGGAA
CAGGAAAAACCACAATACACTGAGGAAGAATTAGAAAGGTATGAAAAAAGAGGAGCACAG
ATTACTGATAAAGGATGGTTACAGTCTGAGGATGGACGATTAATAATTCCTGAAAATGCT
CAATGGAAAATTCTTAAGGGTTTACATCAGAGTTTTCATTTGGGTCCAGAGTACTTACCA
AATGGCTTCTCGTTTGTTTGAAGGTAAAAATGTAATGAAAACTTTAAAGAATATAATCAA
AAGGTGTGAAGTTTGTCAGAAAAATAACCCAAAGACTGAAAAACTAGCAAAATCTGGATT
ACAAAGAAGTGGGAAGTATCTTGGAGAGGACTGGGAATCGATTTTACTCATATGCCAAAA
GCTAATGGATATTCTTGCTTATAAGTTTGGGTAGATACTTTTACTGGATGGATTAAAGCT
TTTGCCTGTCGCAGTGAACAGGCTAAGGAGGTTATAAAGATTTTAATCCATGAAATTATC
CCCAGGTTTGGGCTGCCACGGAGCCTTCAGAGTGACAATGGCTCTGCCTTTAAAGTTGCT
GTAACTCAGGGGGTATCTAAAGCTCTAGGAATAGAATATCACTTACACTGTTCCTGGAGA
CCCCAATCCTCAGGAAAGGTTGAAAAAGCTAATGATACTATCAAAAGACATCTGCGCAAA
TTAACTCAAGAGACGCAGGACAATTGGATTAAAGTCCTACCCATAGCTTTAATGAGGGCT
TGAACTGCCCCCAGAAAGGAGGGACTGTCCCCCTTTGAATGTATTTATGGAAGGACTTTC
TTATGCACAGACATTGTTATAGACCCTGAAGCCTTGGAATTAACTAGTTATGTAACTCAG
CTCTCAGCTTTTCAACAGGCATTAACAGAACTCCGGGAGATGACTCCTGACCCCGCCTCT
GAGTCAAGCAAGCCTCTATTTGAGCCAGGAACTGAGGTCCTCATAAAAACTTTGGGATCT
GGGGGCCCATCCCTCGAGCCCCTTTGGGAAGGCCCTTACCAGGTTATTCTTTCTTCTCCC
ACAGCTGTCAAAGTGCCAGGAATTGATTCGTGGGTACATCACACTCGAGTAAAGAGGTGG
CACCCTGACCAGAACTAAGTGACATCTTTTTATGTCTTTATGTTCTATGCTCTGACTTTG
TACTTTTCAGATGGGCCTGATAACCTATGTGAGCTTACTTCTGCTGACTCCAAATATCCT
GAGTCTGCCGTTTGATCCTCCAGACAATGCCTTCCTGTCCTGGGCTCACTCCTACGCTGC
ATTCCACAATCGGTCTAACTGCTGGGTCTGTGGAACACTCCCCTCTTCATCAGTGGAAGG
CTTCCCGTGGTGGGCATCTCCACTTCAAGGAAAAGACTTTCGCCAAGTGTGTAAATACCT
TCGACAACAATTACATGCGATGCCTCTTCTTCATCTGATGACACCTACCAACCCTAAAAT
AGACTGGTGCAGCACTTTGTACTTTAACTATATGGACATAATGTGACTTTTAATTTTGAT
TATACATTGTCTCAGTTCAATGACTATTTTGCTACACATAAGGCAATTAGGTCTAGATCT
AATGGTTTTTTACCTGACGTTTATCAAATATGGGATGAGGTTATGTGGCTAACTCCTGAA
AGAGGACGTTTAATATCTACTGCCCCTATATGCTGGGAACAAACAGAGCCATCCCCAAGA
GTTAGCCAACGACTTAATTACAATGATTGGAAACAATTGGGATTTTTGCCTCAAGAAAGA
TGCAATGTAATCATTCCCATGTTTTCCGACCCCAGTTCAGGTTCTCTTTGTCTGGCCAGG
CACTGATCGGGACTGGATATCTCAGTCACGCTGCCTTGCTCCAAATGGGACTTATTGGAT
ATGTGGCTCTTACCTATGGGCATGGCTTCCCCCTGGTTGGATAGGGAGATGCACCCTGGG
TCTAGCCTTTACTCTCAGCTTTATATTTTCAGAGCTCCCAGAAAAGCCTGCTAATTTACC
CCACCTTAAAACTCGGTGGGCAAGGTCCGTATTTCACTGGGATGATTATTTGGCTGCAGT
GTTTGTTCCCTCTTTGGGAACTACAGATGTTATGCTACAAGTGGATGCTTTGACTAATTT
CACTCAACAGGCATTACAAGATTCTCAAAAGGCTATTTCAGCTCTTAATGCTAAATAAAC
ACAAATTAAAAAGGTGGTTTTACAAAACAGATTGGCTTTAGATATTCTGACAGCTGCACA
AGGGGGAACGTGTGCTATTATTCATACCCGATGCTGTCCCTATATACCTGATAGGAGCAC
GAAGGTTACTCATTTTACTAAACACATGAACAGGATGACTGGGGCCATGGCTACTCCTGA
AGCCTCAATTGCCTCACTTTGGGAGACATTAAGTAGTTCCCCATGGTGGACAACTATCTT
AATTACAATAATTCTGATTGTTTTGTTTTTATTGTTTGCTCTCTGCATCTGTAAGTGTAT
AACTGGATTTGTTTCTAGCCTCATGAAAGCTTTCAAGTTACAAATGGTTGCTCAAACTCC

TGCTACTGTTGCAGCTGCCTCCAGCTACTATTTGGGGCCCCTGGATCAGATATCCTCAAT
ATAAGGATTAGGAGAATATGTTGCCTCCCCAATTTAGGGACAACACCCCTTCTCAGCTCC
GAAGCAGTTATGGAACGAAAACGGTGCCCATTTTCCCTAGGCAACATAATTCTCCTGAAA
GAAAAGGGGGGAATGAGAGGGTAACAGGCAGGAAGGCCAGGGGTCTCCAAATGGAGGAAA
TAGCCTGCAAGTGTCAGACTTTTTTCTCTCTCTTAAGCAGCAGGAGGAAACAAAGTAGCG
ATATTTTTTCCTTCTCTATACAAATTTAAAAGGAGGTTTTTCTCTTAAAATGCTGTGTTG
CCACGACATCTGGTTTCACCTGAAGTTAACCAATGCCTTTTTCTTATGGAAATGTTTATC
TTAAGCTATGGTAATGTACTATGCATTTACCCCAAACTCTGTCTTCAAGTCGGTTCCGCC
TTTTGGCTCAGTATGTTATACTCCGATAATCTATACTTCCTCTAATCTATGTAAAGGACA
CTATTTGTATGGTGCTCTGCCCTTCTTCAAGATTCAAGCTAATCCTTTTATGGCCCAAGA
TGAACCATTTGGAGCCAAGATTATCCCAAAATACATCTTATGGGTGAGGGGCCTGGTTCC
ATTCTAAGTTTTGAGACATTCCTTTCTTTCATTAACAGACTGCTGGTGTCTATATAACAT
CCAGCTAAAGACTAGCAGGGGGTACTCTTTCTGCCCCCTGCTGATGCCTATGTCAGAAGC
TTTCTCTATCTCCTTTATACTTTAATAAAACTTTGGTACACAAAAGCTCTGAGTGATCAA
GCCTCGTCTCTGGCCCCGGATT
>CAP_ERV_13
TCTCCCCCTCTCCCTCCCCCATGCACTCCTCCACTCTCTTCTCTTCAAGTCTTTGGGTTG
GCATGCCCTCACCCTTCGAGGATGGAGTCTCCTGCTATCTTCTAAATAAAATAGAGCTGT
AACACTGATTTACCTGAGAGCTATAACACAGTTTGTCCAAGACCCGAGAGCTGTGATGCA
CCGAGGGCTTTAATGTCTGTCGCTCCAAATCTTTGTTGTGATGAGACAAAGAACCGAGGA
ACATACACTTGCGTGACATCTATGGTGCTGTGACTCGGATATAACCTGGCTGAAACAACC
TCCGCGTGGAAGAGGCCAGGCACAGCAGGAGCCCAACTCAGCGAAGCTCCCGCAGTAGAG
GCGGAAGGTGAAGAAAATCCAGCGCAGGGGAAGGCCCATCATGCTGGAAACCGGAACAAC
GGAAAACAAACTCAGCGGAAAGCTCACGTGGCCCAGTCTCAGATCCCAGAAGACCTCTGG
TTAAGGTAAGAGGTCCTCACTGGAGGGACATGCCTAATGAAATCTTAATTCCTTTACAGT
CTCTCGTTTTTTGTTCCCGCAAACCTCCTGCGAACAGGCGGGCGGCAGGGGGCACAACTG
AGGGACTCTGGAGAGGCTGCTCCTCAGCATGTCTCAAAGGCACTATCTGCTGAGCCCCAG
TAGCTGTTACACAAGCCAGTGGGGGTTCTTCTGTCCTTTCTCTTCTCTGTGCCAAGGATC
AGACCTATGAAATTGTGAGCACCGGTCAGATATTTAGCAAATTTTCCAGCGGGCTATGAA
GGGGATTCCTTGGCACGTTTTTCCCACTGCTTTTTCCTCCTGTCCTTCAGCTCTCTCCCA
GGACTCAAGCTCGGCCAAAACTAATGAAACTCAGGCCCTGATGTCTCATTGCAAAAATTC
ATTGAGAGACAAAGAGATAAGAGGTGGATTTGTTAGGATTCAGAGAGAAGCCACTCTTCA
GGGTGTGAACCATTGCCAAGGGCAAGGGCTGGGGCCACGGGATTAGGCCTGGCTAGGTTT
TGTGAAGGGGTGGAATTCATATGCTAATGAGTGGGAGGATCATCCCTACCATTGGGGAAC
CACCCACTCCTCCCTCTTTTGCCTTGGAGCTGTCCTGCCACCTCTGGGTGTGTCTGTTGG
CTTATAGATTGGGGATTAAGTACTTGAATTTCACTTTTCATCTTGGACCCAGTTGGTTTT
AATTGGTTTACATTATCCCCTTGTGCTATGTCATTCTTTCAAATGTTGTGCTCTGCCCCT
TTCCCTCCTGTTTCATGCTCTTTGCCTGAGCCCCATCCAGACCCACAAGGTTGCCTCTAC
GATCTTCTGGAGAGACAACCAGAAAATAGATGGCCTTGGGAGCGAAATACTGTATAATAT
CCAACCCCCTAATCCTACCCAGTACTGGTCACACTGGAGATCTTGGGAACGCCCAACTCC
ATCAGTCCAGGGGCCCCAGGAGGTCATCACGCCTTGACCTGCCTAGGATCTGGTCCTCGA
AATGTTGTCATGCCTTAACCTGCTCGGGGATCCGCTAGTCCCAGGGGTCCCAGGAGGTCG
TCACGCCTCGACCTGCCCAGGGATTTGATCTCTAGGAGGCTGTCACGCCTCAGCCTGCCT
GGGGATCTGCACCCTGACCTGGGGACGCCTGGCTCTCAGGTTACAACAGTACTAGGTAGG
ATGAGCTTACATCATATCTATTCCCATCCACGGTGGGCAAACTAGCAACGAGTTACAAAC

CCCTTAATTCTATCCAGCATTGGTCATGCTGGAGACCTTGGGGATGCCCAAACTCCAGTC
CGGGGGTCCCAGGAGGTCATCACGCCTTGACCTGCCTAGGGATCTGGTCCTCGAAAAGTT
ATCACGCCTCAACCTGCTCGGGGATCCGCCAGTCCCAGGGGTCCCAGGAGGTCGTCATGC
CTCAACCTGCCCAGGGACCCAATTCTCGGGAGGCCGTCATGCCCTGACCTGCCCGGGGAT
TCGATCTCTAGGAGGCTGTCACGCCTCAGCCTGCCTGGGGATCTGCACCCCTGACCTGGG
GATGCCTGGCTCTCAGGTTGCAACAGTTCTGAGTAGGATAATCTTCAGAAAAACTCACCC
CTAAGGAAGTCCACCCATGGAAACAGAAGGAAGCCTATTACTTGTGGGACTGTGCCCAGT
CTCAGCAATAACTCAGGAGCCCGGTGAGAACCCCATTGCCTTTCTGGAAAGGCTGAAAGA
GGCACTCCAAAAGTTTACCAATCTGGACTTAGACTCTTACGAGGGACAGGTGATTTTAAA
GGAAAAATTCCTGTCCCAATGTGCATCAGATATCAGAATTAAGTTACAACAACTACAACA
GCAGGACCCTGCTGCCTCTTTAGATGAGATGGTCCAGACAGCCACCAATACCTTTTATAA
CAGAGAACAGGAGAAGGAGGCCAAGGCCCAGGAGAAGGAGGGAAAGAAAGAGACAAGCCA
TGCCCAGATGCTGGCTGCCCTCCAGAGAAGCCCTATTGCAAACCCCGAGTCCTTGAAGGA
CAAGGCATGAGACAAATGCCTGATCTGTAGACAGGCGGGGCATTGGGCCAAAGTGTCCAA
ACCATGACAAGTCTCCTAAAACAGCTTGCCACAAATGGCATCAACTGGGACATTGGGCGG
CACTCTGCCCTCGGGACCCAAGAGCCTCAAGGTCAAGTGCCAAGCCTACCCTCACGATGG
TTCAACAAGACTGAAGCGGCCTGCTCCAGCCAGCCCACCTGTCACAGATAACCATCACGG
GGCTGGAGCCAAGGGTGCAACTGGATGTGGCAGGTAGGTCCGAGAATTTCTTGGTTGACA
CAGGGGCTGCCTACTCTGTCTTGATCTCCTACTCCGGACGCTTCTCCTCCCAAACCTGTA
CCATTTTGGGTGCTACAGGAAAAGCAACTACTAAAAGATTCACCCGAGCACTTATTTGTT
GCTGGGATGGACAAATATTTTCCCACCAGTTTCTGGTGGTCCCTGAGTATCCTACTCCCT
TATTGGGAAGATATATATTCACTAAACTGGGGACCACCCTTGTGATGGGAAGTTTTTCAG
CCCCTAGAGCTCTACAGCTCCTGGTTACTACTGAGGAACCCATTACACTTTCAATAGAGA
GGGACCAAAAACCATAGGAAGACAAAATTAACCCCCAGGTGTGGGACCAGGGGATTCCCA
GACGAGCTTACCAAGCCGAAACAGTCATCATTGTCCTCCGAGATCCCACTCGGTTTCCTA
ACCAGAAACAATACCCACTCAAAAGAGAGGCTCAGAAGGGACAGCCTTTAATAAATAAAT
TCCTTGCTTGTGGGCTATTGGTCCCCACCAGCTCGCCATGTAACACTCCAATCCTCTCAG
TAAAGAAAAAAGACGGAACCTGGTGAATGGTTCAAGATCTCCAGATCGTAAATGAAGCTG
TAGTCCCCCTCCGTCCCACAGTACCCAATCCCTATGTAATCTTGGGAGAAATCCCACCCA
GTGCCAAGTGGTTTACAATCTTGGATCTCAAAGATGCATTTTTTGCATACCACTGGCTAA
ACAATCCCAATATCTTTTTGCCTTTGAGTGGGAGGCCCCAGGAGAAAAACGCCAACAGAT
GACTTGGACAGTATTACCTTGGGGTTCAGAGATAGCACCCACCTGTTTGGACAGGCCCTT
AGCCAGGATCTCCTAGATCTGGACCTGGGACCTAATGGAAAAATATTACAATACGTAGAT
GACCTACTAATCTGCTCTCCAGATGAGAAAAGTGCCCAACAACATGCAATTCAGGTTCTA
AACTTCTTGGCAGAAAGGGGATATAAAGTCTCCCGTGCTAAGGCACAGATGGTCGAGACA
AAGGTCATTTACCTGGGAGTTCAGATTACACACGGGTCCAGGAGGCTGTCCTCTGATCGG
GTACAAGGAATCCTCCAGTTGCTCTCCCCCACGACTTGAAAACAATTGCGAGCTTTCCTG
GGACTAACTGGATATTATAGAATCTGGATACCCAACTATGGTCTAATTGCCCAGCCCTTA
TATGAAAGCTTAAAGGGGTGAGACGATTCAATCCCACTGATGTGGGGAACTCCTCAAAAG
AAGGCAGAGGCTACACTAAAACAGGCCTTAACTCAGGCACCTGCCTTGAGGTTGCCAGAC
CCAGAAAAAGCATTCCAACCTTATGTCCATGAAAGAGAGGGAATAGCTTTGGGAGTGTTA
ACTCAAAGGTTGGGATCTGAGCCCCAGCCTGTAGCTTACTTATCCAAAAAGCTTGATCCA
ACTACCTGAGGCTGGCCCCCCTGCCTTCAAAATCTTGCAGCTATTGCAATCATGATAGAA
GATGCTTTAAAACTCTCCTTTGGGGGCAAACTAACTATTTTTACCAGCCACCAAGTAAAA
CAACTCTTAAATGGAAGAGGCCATTTATGGATGTCTGGGTCGCTAAGAGTTGGGCGTGAC

TGAGCGACTTTACTTTCACTTTTCACTTTCATGCATTGGAGGAGGAAATGGCAACCCACT
CCAGTATTCTTGCCTGGGGAATCCCAGGGACAGAGGAACCTAGTGGGCTGCCATCTATGG
GGTCGCACAGAGTTGGACACGACTGAAGCGACTTAGCAGCAGCAGCAGCAGATCAAAGAA
TCCTCAGATATCAAGTAATGCTGATGGAAAATCCAGGCCTCACTATATCCCCTTGTGAGG
GTCTTAACCCAGCCGCCCTCATGCCTACCCCTGAGGGCTCTCTCCCCTTTCACTCATGTC
TAGAAACCTTGGACCACTGGACAAAACCCTGAGAGGGATTGTCAGAAGATCCTCTGACCA
ATCCTGAGGAAATCTGGTACACTGATGGAAGCAGCTTTGTCTTGGATGGAAAAAGAAGAG
CCAGGTATGCAGTAGTCTCCAATTTTGAGACCATAGAGGCTAAGCCTCTGTCACCAGGTA
CTTCAGCCCAGTTAGCTAATCTCATAGCCCTGACTCGAGCTTTAGAGCTGGGAAAAGGAA
AAAGAATAGCCATTTACACTGACTCCAAGTATGTCTGCCTGGTGCTACATGCACATGCTG
CTATTTGGAAAGAAAGGGGCCACTTGACCACCCGAGGGTCCCCAATCAAATATGGTGATC
AGATTCTTTGACTCTTGGAGGCAGTCCATCTGCCCACTGAGGTTTCAGTCTCCCACCGTA
AAGGACATGAGGGAACCAAGCAGCCAATCAGGCAGCTAGGAGAGCAGCATTACAGAACCA
TGACCTAACAGGGGTTGCCACCTTAGTTCCACAGACTAATTTGCCAGAAACTCCTTCCTA
TACTGAAGGTGAGACTCTCAAAGCTAAGAGCGAGGGCTTCCAAGAAGATCATATGAGGTG
GCTCCAAAAGGAGGGACTCCTTTTTCTGCCTGGGAACCTCCAATGGAAGTTGGTTAACTC
CTTACATGCCACTACTCATTTAGGAGAAAAGGCCCTCCAAAGATTACTAGAAAGGTCCTT
CAGAGGAACAGGCTTCCAAACAACTATAAGACAGGTGGTCTGTTGTCCCACTTGCCAATT
AAACAACCCCCAAGGAGCTCGAAGACCCCAGCTGGCCCAGCCCATCCAACGACATGGGGC
CTACCCAGGAGAGGACTGGCAGATGGACTTCACCCAGATGCCAGTTTCTCAAGGGTATAA
ATACCTATTAGTCATGATAGATACATTCACAGGATGGATTGAAGCTTTCCCACCCAGACT
GAGAAGGCTGAGGAGGTGATATAAAAACTGCTCCATGAAATCATTCCAAGATTTGGTCTG
CCCAGGTCATTACAAAGTGACAATGGGACATCATTTACTTCCAAGGTCACCCAAGGGGTC
TCAAAAGCATTGGGCATTACTTATTATCTCCATTGTGCCTAGCCAATCAATTCTTAAAAT
CAGCTATAAAAAAGATAACCCAGGAGACCTCCCTGGGGTGGAAGGAGGCTTTACCGATAG
CTCCTCCGCACCCGCATTGCCCCTAAGGAACAGGCTGGTCTTGGTCCTTATGAGATGCTA
TATGGGAGACCTTTTGTTTATGTCAATGACCTCTTCCTAGATCCAGAGGCTCAGACCCTC
CAGTCTTATACCATGGCCATTGGGCAATTCCAACAGGATATACAGTTGTGGGGTATGAAC
CAGCACCCAAAAGATTATAAGGAGTCACCACTATATGCTCTGGGGACTCAAGTCCTAATT
AAAGTCTGGAAAGATGGGTCCCCAAAAGCTCAACTCCAGCCCACATGGAAGGGCCCCTAC
CCTGTAATACTTTCTACCCCCACAGAAGTCAGGGTACCGGGACATGACTCCTGGATTCAC
TACCATGAGTCAAGCCATGGAAGAAGACAGAAGAGGACAGTCAATACACCTGTGAGCCCC
TCGGAGATCTCAGATACCTATTCAGGACTACCAATGAGTGCCATTCTAATGAACACCCCC
CAAATCTGGTTTCTGGGGATAAGATTTCTCAGGATAACTCTAAACAGCCAACATAGCTTG
ACAGAGATTGTACTCCAAAACAGACAAGAGATAGATCTTCTGATCCCTGAACAAGGAGGG
ACTTGAGTCATCCTGGCGATGTGAATTTAAAACTGACTTTCATGTTTGGGAATGCATGTA
AGAATTAAAGATTTTAAAATTTAAAAAATAATAATAAATTTAAAAAAAACTGACTAATGC
CCCTACTAGTCCTTGTTACGCTACATTGATGATGCTTATGATTATTCCATGAACTGTCAA
TTGTCTAACCTGTCTTGTCTCTGCCTAGGTCAACAAGCTACAACATGCAGTGCCAGTTCA
ACAAAGATATAAAACTACAGCCGACCATGAAAAATATCACACCCTTAGATGGACACCGCT
ATAAGCACTCTGAGGCTTGAGACTAACAAGAGGGGGAGGCCCAATACCCCTCACCGCCCC
AGTTCAGCAGGAAGTAGCCAGAAAGACCTCGACGCCCCTATTCCCAAAGAATTGGGCCTC
CCATCTCTTGAGGGGGGAATGTTAGGTAGGTAGAATAGGGAAAAGGAGTCCAAAATGGCG
GTGGCTAAAAGACAAGGAAGGGAAAAGCCCGCGAAAATAGAACAAAAGAAGGTCCGAGGA
CCAGAGTGAAGACTTCAGGTAGAACAAACAGCACTCCTGGCTAAGCCCAGTTTGCACAGG

GCAGGCCCAGGTGGAGGAAAACATAAAAGGAGGAGCCAAAGCGCTTTCTCTCTCTCTTTC
TCTCCTGCATGCATGTGCTCTTTCCCTCTCTCTCTCTCCCCCTCTCCCTCCCCCATGCAC
TTCTCCACTCTCTTCTCTTCAAGTCTTTGGGTTGGCATGCCCTCACCCTTCGAGGATGGA
TTCTCCTGCTATCTTCTAAATAAAATACAGCTGTAACACTGATGTACCTAAGAGCTATAA
CACGGTTTGTCCAAGACCCGAGAGCTGTGATGCACCGAGGGCTTTAATGTCCGTCACTCC
AAATCTTTGTCATGACGAGACAAAGAACCGAGGAACATACACTCGGGTGACA
>CAP_ERV_14
TGAAGAATCTAAGCTACTCCATTTTGTTAACAGAAAAACTCCATTTTGTAAGGTTCCAGG
AAAAGAGCCTTTGGAAATCCCCTGACCTCACCCCACCACCCATGAGCCAATCGGACCCCA
GACCAGAATCTCCTGACAATGTTCAGGAACTTGTGGTCTACCTAGGTAATAGGGACCAAT
CAAAAGCCAACACACAACCCTTCGAAAGAAAGTGACCAATCAGACGTCCCCCTACCTAGA
AATTCTTTTTTTCATGAACACTCCCTACATAAGCAATGTAATCCAAAACCCGGGGCTCCT
CCCTATAGCCGCTGCGTTGGTAACGGTGGGAGCCCCAGCTCGAGCTTGGTGATAAAGACT
CTCTTGCTTTTGCATTGGATATTGGCTCCCTGGTGGTCACTGGGAGATTTCGCGACTTGG
GCATAACATTTGGGGGCTCGTCTGGGATCTCCCACTGGCTCCATGGGAAGACCAATCCGG
AGAGTCATCTATGGCCGGTAAGCTTTTTTTCTTTCTTTCTTTTCGTTTCTGACAGGGCCT
ATTTTCTGAGACCAGTATATGACCCAGGTGGACGCGCTGACTGGTCACCGGTGTGGGTCA
TTGGGAGATGTCCCCGACCCCCTCGGAGGGGATGGAGTGCCCCTGTACTCTGTAGACAAT
AGTGGGTCGCTCCTGCTGAACGGCTGGAGGCCGTCATCTTACTTTCAGTTTTGCCTCCAG
ACTCCCGGAGGTCTGTTTCTCCTGTCTCTCTGTCTGTCTGTGTGGCTGTTTGTATTGTGG
TATTGTCTGTGTTAATTTACCAACATCTGACCGATCCAGGAAAGATGGGGCAACATCAAT
CTAGCCCTACTCCTCTATCTCTGATCACTGACCATTTTAAGGAGGTCAAGATGCACGCTC
ATGACCTCAGTGTAGAAATTAAAAGGAATAAATTAATCACTTTTTTCAGCTCAGAATGGC
TGACCTTCCATGTAGGCTGGCCCTCAGAAGGCACCTTTGACTTGGGAACTGTACACCAAG
TCCAGGATATCACCTTCTGACCACGGATAGGACACCCTGACCAAGTTCCCTATATCATAG
TATGGGAAAATATCATACTGCACCCCCTCCTTAGGTAAAACCTTTTTTGCCTCAACAGGG
ATTTTCTACTGCTTAGGGGTTAGTGACACGTGTAGGAAAGAACCTGAAGAATCCCAAGGA
GCCTGAAGCAACGGCACCGCTATACCCCATCTTGCAGGGGGGAACAGAAGAAGAACTCCT
CTTCCCTCCCCCTTACCAACCCCTTGAACCACCATGGGCCCCTGCCATCCCTCCACCCAG
GGCCGAAACACAGGAAGGGGGTCCCGCACAAAATACTCGCCATAGGAGAGCCATGTCCCT
GGAGGGGCCAGCAGACTCAACAGTGGCCCTCCTCTTACGAGTGGCCAGCCCCCCCGATGA
GGAAGGAAACCAACCTCATCAATATTGACCCTTCTCTACTAGTGACCTATATAACTGGAG
AGCCCAGAATGCTAAGTTCTCAGACAACCCTAGAGATCTAACTAACCTTCTAGAGACTGT
GCTTTTTATTCATCAGCCCACCTGGGACGACTGCCAACAGCTGCTCCAGATTCTCTTCAC
CACAGGAGAGAGGGAGAGAATACAGAATGAGGCCCGTAAAAGGGTCCCAGGGGCAAATGG
AGAACCCACCACTAATAGAGATGAAATTAACACCTCTTTTCCTTTATCGCGACCTGACTG
GGATTACAATATGGCACAAGGTAAGGAGAGGCTCCGGGTCTACCGCCAGACTCTTTTGGG
GGGGCTTAAGGCAGCTGCACGGAAACCTACTAACCTAGCTAGAGTAGGAGATGTTCAACA
AGGCCCCACTGAGAGTCCCGCCGCTTATCTGGAAAGGCTGGTGGAGGTCTCTAGACAATA
CACCCCCATGGATCCAGAAGCAGAAGGCACTCAGGCTACTTTGATAATGCATTTTGTCAA
CCAGGCAGCCCCCAACATTAGGAAGAAGTTGCCAAAACTAGAATGCCTGGGTGAAAAGAG
TATCCAGGATTTAATAACAGTAGCAGAAAGAGTCTAGAACACCCAAGAAACCCCTGAGGA
AAAACAAGCCAAAGCAGCAGATCGCCAAACCCGTAACATGGAATGCATCCTGCTGGCTGC
AACAGTTCCTGACTCGGGAGAGAGGGAGCGCCAGTTGTGCCGCCTGGCTGCTGAAGGTAA
GAGCTGTCCCCGTATGCGACCAGCATTAGGAAAGAACCAATGTGCCTATTGTAAAGAGAA

GGGACATTGGGCCAAGGACTGCCCTAAGAAAAAGAAGGAACCATGAAAGACCCCCATCCT
GGCCATCGAAGAACTGAGCGATTAGGGAGGGCAGGGTTTGGCGCCCCTCCCCAAACCCAG
GGTAACCCTAAGAGTGGAGGGGACCCCAATTGACTTCCTTGTTGATACGGGGGCTCAATG
TTCGGTTTTGCTAGAGCCCCAGGGAAAACTGGATGGAAAGACCTCTTGGGTGCAGGGGGC
TACTGGAATGAAACAATACCAATGGACTACCCAAAGATCAGTGGACATGGGTGCAGGCCG
GGTATCCAACTCTTTCATGGTCATTTCGGAATGCCCCTTCCCTCTGTTGGGAAGAGACCT
GTTGACCAAAATGGGGGCCCAGATTCACTTCCTCCCGGGGGAAACTAAAATACCTGACCA
GTTGGGCAGACCGATTCAAGTACTGACAATTCAATTAGAAGATGAATATCGGTTGCACCA
AAAACTGATGCTCCCCGAAGCGGACATTCAAAAATGGCTAGACGAATTCCCTGATGCGTG
GGCTGAGACAAGGGGAACCGGTTTGGCTGGACACCGTCCTCCTATATATATAGATCTCAA
GCTGGGAGCTTTTTGCCTTTGAATGGTCTGACCCAGAAGAAGGAATCAACGGCCAGCTGA
CCTGGACTCGGCTGCCACAGGGATTTAAGAATTCTCCCACAATCTTCGATGAGGCCCTCC
ATGAGGACCTGGGTGAGTTCAGATAACAACACCCCCAACTAACCTTGTTACAATATGTAG
ATGACCTCTTAATTGCAGCAAAGGACCAACAGACTGGCCTTATGGGCACCCGAGAACTAT
CGTAGACCCTTGGAAAATTAGGGTACCGAGCCTCAGCCAAAAAGGCTCAGATATGCCAAC
CAGAGGTCACCTACTTAGGGTATGTATTAAGGGAAGGACAGAGATGGCTGTCAGAGGCAC
GAAAGGAGACGGTACTCAAAATTCCTATCCCAGATTCACCTCGAAGGGTGAGAGAATTTT
TGGGGTCTGCTGGTTTCTGCCATCTTTGGATCCCAAATTATACAGAACTGGCTACACCTT
TATATGAAGCCACAAAAAGTACCACCCCTTTCAGTTGGACAGAGCAGATGGAGACCACTT
TCAAGACTATTAAAACAGCCCAGCTGTCAGCCCCCACCCTAAGGATGCCTGACGTTACAA
AACCCTTTCTCCTGTATGTAGATGAAAAACAAGGAGTGGCAAAGGGGGTACTGGTGCAAC
ACCTGGGACCTTGGAAGTGGCCAGTGGCTTACTTGTCTAAACGCCTAGACCCCGTGGCGT
CAGGCTGGCCCCCATGCCTCCGCATGATTGCAGCAGTGTCCCTAATGGTCAAGGATGCAG
ATAAACTGACACTGGGGCAAGAGTTGCAGGTCACCACCCCACACGCCATTGAGGGTGTCC
TAAAACAACCCCCAGATAGGGGGATCAGCAATGCTCGGCTGACCCACTACCAGGGATGAC
TGCTAAACCCCTCCAGAGTCATCTTTCTGCAGCCTACTGCTCTCAATCTGGCTACACTGC
TTCCTAAGCTGGATTTGGAAGCCCCAATCCATGACGGCAGCGACATACTAGTCCAGGTAC
ATGGGACGCAAGAGGACTTGCAAGATCGCCCTCTAGCGGATGCTGAAGTTACCTGGTATA
CGGAAGGAAGCAGTTTCGTCGGAGATGGGCTCAGGTATGCAGGGGCAGCCATAACCACGG
AAACCTAAATTGTGTGGGTAGAAGCACTACCTCCGGGCACTTCGGCTCAAAGGGCTGAAC
TAATTGCCCTAGCAAAAGCCCTCCAGCTGGGGAAGGACAAAAAACTTAACATCATTACTG
ATAGCCGATACGCCTTTGCTACTGCCCATATACACAGAGCCATCTACAGAGAGAGAGGAC
TCCTCACAGCCAAGGGTAAAACGATCAAAAATAAAGAAGAAATAAAAGCCCTCCTCTCAG
CATTATGGCTTCCTAAAAAACTAGCCATAATACACTGCCCGGGACACCAAAAGTCAGACA
CCCTGACCTCAAGGAGAAACAATTTAGCAGACAGAGCAGCCCGAAATGCAGCCCAGGGCA
CCATGATAGAAGCCACCCTTCAATTGCCCGACCCCAGGAGCTCTGTTCTGCCAGCTTTAC
CCAACTACTCACCAAGAGACTTAGACTGGATAAAAAGTTTACCTATGACCCAACAACTGG
CCGGGTGGTGTCAGGCAGTGGACAGCTCCCTGATCCTCCCGGAGGAACTAGGAAAGCAGG
TGTTACTGAGGATGCATCACGCCACTCACCTGGGAACCCGCAAGATGCAGGACCTGATTA
GACATGCCAAGATTACCATGAGAGACATCAGATCGACAATAGAAAATATCGTATCAACCT
GCAAGGCCTGCCAGCTCACCAACACTGCCCGTCATCCAGCCAACCATGGGTCTAGAGAAC
GCAGGAGTCGGCCAGGAGCATACTGGGAGGTGGACTTCACTGAGGTAAAACTGGGTAAAT
ATCGATACAAATATCTACTGGTATTCATAGATACATTCTCAGGGTGGGTAGAAGCCTTCC
CCACCAAGAGAGAAACAGCACAGGTGGTGGCCAAAAAGCTGATTGAAGACATCTTGCCAC
GGTTCGGGTTTCCTGCACAGGTGGGGTCAGACAATGGGCCAGCCTTCGTATCTCAGGTAA

GCCAGGGGGTAGCCCAGGCTTTGGGGGCTAGGTGGAAGTTACATTGTGCTTATAGGCCCC
AGAGCTCAGGACAAGTAGAAAGGATGAATAGGACACTCAAAGAAACATTAACAAAACTAG
TTGCTGAGACTGGCAGGGACTGGGTGGCTCTTCGCTTTACCGGGTGCGAAACTCCCCCTA
TCAACTGGGACTTACCTCCTTCGAAATTATGTATGGGATACCTCCCCCAATAATTCTAAC
CTAAAAACAGAGGTGCTAAAAGAAATAGATGATCAAAGATTACTCTTTTGTCTCCAGTCC
TTACAATACTCCTACAGGGACACGTGGAAGAGGCTCAAAGCATTATATGAATCAGGCCCA
CCAACCTGAATCCCACCGCTACCGACAAGGAGACTGGGTGTACATGCGCTGCCATTGTCA
AGAGACCCTAGAGAAGAGCCCAGGTGGAAAGGGCCCTTCCTAGTTGTCTTGACGACATCC
ACTGCTCTAAAAGTTGACGGCATATCCACTTGGGTTCACTACACCCACGTATGGCCTGCG
GACCTGTTCTCCCTAAGGGAAGAATTCCTTCCCCAATGGAAGACCAAGCTGGACAAAACA
AATCCACTCAAACTCAAGCTACAAAGGCGATAAAATTACTGTTTATGCTGTTGTCCCTAC
AGCTTACTGCTGCCGCTAACCCTCATCAACCGATGAACCTGACTTGGATGATTCTAAGTG
CAACTACAGGAGAAGTAATTAAGTCCACCTCGACCATTCATCCCAAAAACACGTGGTGGC
CAGACTTGGAATTCGACCTCTGTCTTCTGGCCGCTGGATCTTGGGACATTAGCGAATGGG
AGGTAAAGACACCCGGCAAGCCTGAATGTGGGGCTGGCACTAATAGGTGCAATACTCGGC
CCCCTACTAACTCAGGACCTGGATGCAGCCACTACATTCAATGGGCAAGCTTACGGGAAA
CACCCTTCTATGTGTGGCCTGAAGGGAGATGGAACCGGGCGACCCTCAATAACTGCGGGG
TGCTGACAAGTTTTACTGTGCCACTTGGGGATGCGAGTCAACGGGGATGGTCGATTGGGA
GCCCCCTATTCGTGGTGACTTGATTACCTTGGGTCGCGTTCCGGGGCCCACTTGGTCAAC
TGGGGGACATGGGAGGGAGTCCCTGGCCAAGATAAGAGAAGGGCTGAGTCAGAGAAAAAG
AGAAAGAAAAAAGTCTCAAAATTGGTTTGAATCCTGGTTCAACTCATCCCCCTGGTTCGC
AACTCTCATTTCTTCCTTAGTGGGGCCCCTGATTATCTTGTTACTGCTACTCACCTTCAG
GCCATACTTACTAAACAAATTGGTGGCCTTCATTAAAAGCCGCATCAACACGGTGCAGCT
CATGGTACTCAGATCCCAATATACGGCCCTGCCAATGGTCCCCTTAGTGGGAGACAATAT
AGAGCTGACCCCAGACCCATGATTGGGTCTGTCCTGGATCCTAGAGAAGGGGGGAATGAA
GAATCTAAGCTACTCCATTTTGTTAACAGAAAAACTCCATTTTGTAAGGTTCCAGGAAAA
GAGCCTTTGGAAATCCCCTGACCTCACCCCAACACCCAAAGCCAATCGGACCCTAGACCA
GAATCTCCTGACTTAACGTTCAGGAACTTGTGGTCTATCTAGGTAATAGGGACCAATCAA
AAGCCAATACATAACCCTTCGAAAGAAAGTGACCAATAAGACGTCCCCCTACCTAGAAAT
TCTTTTTTCATGAACACTCCCTACATAAGCAATGTAATCCAAAACCTGGGACTCCTCCCT
ATAGCCTCTGTGTCAGTAACGGTGGGAGCCCCAGCTCGAGCTTGGTAATAAAGACTCTCT
TGCTTTTGCATCGGATATCGGCTCCCTAGTGGTCATTGGGAGATTTCAC
>CAP_ERV_15
GTCAGGGCAAGGTCAGGATGAAAGGTCAGGGCAAGGTCAGGCATGTCGGGACATGTCCCG
GCAGAGGGCGCTGCAGACAAGCCCCGGAGACAGGCCGGCAACCAAGCCAACCAATCAGGA
GCTGACACGAAGCCCTCGCCGTCCAATCAGGAACCGACACGGAGCCCTTGGACACCAATC
ACCGCTGAGCCCGCGTTTTCTTCCTTATATGGGAGCCCCTGCCCGGGCTATAAAATCCTT
CCCCACCCTCACAGACTCCGCAGACTCCCTTTACCCCGCAGACTCCCTTTGCTTCGCAGA
CCCCCCTCGCTCCCTTGCTTACCCGCCCCGGGAGTTCTGCCCGAGAGCGACCGCCCAATA
AAAGGCCCTGATCAACGGTCTATAGGGGTGGCTCCTTCTTCCTGCGGCGTTTCTTACATC
TGGCGCCCAACGTGGGGCTTGAGGTGAGGGCTTCCGGCACTCGCCATAGAGGCCCCCTCG
AGCTCCACCACCGCGGTAGCCCCCCCGACCCAGGCGGCTGACCAACCCCCCAGATGGCAG
GATACGGGGTAAGTCCCTTCGGCTTTGGAGCCCGCCCTCCCTGGCGACCACTTCCTAGGA
CCCAGTAACGGGTGCGCACTTACTGGGCCCTCCTGGTCACCAGCTCGTGAGGGAGACGTC
CCGAACGGCTCTGTAGACCTCCGGCTTCGGCCTCCGGCTTCGGCCTTCCTCGGTCCCCAG

CTCAGGAGGGAGACGTCCCGAACGGCTGTGCGGATCCCCGGCTTCCCTTGGCACCCAAAG
ACATTCGGGCAGCGGGGGTTAGTCTCCGTTTGGTGATCGCCATGGGATCGACGGCCTCCA
AACCTGATCCCCAATATTCCACCCCCTTAGAGGGCCTGCTGGCTAACCTGCGGACCCTAA
AACTAAAGGGATACATCCGCCATATTGGCTCACAATGGCCAGCCACAGGGACATTTGACT
TTGACGTCCTCCGTGATTTAGATAACTACTGCCAGAGAATGGGAAAATGGTCTGAAGCCC
CCTATGTTCCGGCCTTTTGGGCATCTCACCCCACCCTGTGCACCACGTGCACTCTCCGCC
AAATCCTGCTTATCCTGGCTCCCCCTATTCCACCCTCCCGGGCCAAACCAGCTCCTCCCA
TCTCTGAGTCCTCTGCTTTCTCTGTTCCCCTGGAAGATCTGGTGGCCCCTCCTCCCTATA
CCTCTCCCATGGCCCTTACCCCTTCCACTCCAATTTCTGCTACCTCTCCCTCCACTCCGG
ACTCTCCTACCTCCGCTCCCGCCCCCGAGACTCCACCACCGGTCCCGGATCCTCCGGCCC
TTAACCCTATCTTGTGTCCTCCTCTTCCCCCCGTCACTCCCTCCCCTTCCCCGGTTAGCT
CCCACACTCGCTCCCACAGCAACCCTCCGGGAGCCTCCCTTCCCCCTCCCCCAGCCCCGC
TACTCCCTCTCCGACAAGTAGCTGGAGCTGAGGGTCTAGCCCAAGTCCATGTCCCCTTCT
CCCTCCAAGACTTAGCACAGATTGAGGCCAAACTGGGTTCCTTCTCCTCCAACCCCACTC
AGTACATCAAGCAGTTTACTGGTCTGACCTGCGCCTACACCTTGACATGGCAGGACATGT
ATGTCATCCTGGGGTCTACCACCACCCCCGAGGAGAGGCAGGCCATCTGGACGGCAGCCA
AGGCTCAGGCCGACCAGCGGCACTATGCCAACCCCTCCCCCAAGCGCCCCCCAGGGGCTC
AGGCGGTTCCTGACACTGACCCTGATTGGAACTACCAGGAGAGGGGTGGCGGCCAGCTGC
GAGTATGCTATATGATCGAGTGTATCCTCGACGGGATGGAAACGTCCTCTCACAAGGTTG
TAAACCTCCTCAAACTAGATGAGGTGACCCAGGGGCCCGACAAAAACCCAGCCATGTTCC
TTAATCGGCTGACCGAGGCCCTCGTCCAATACACCAGGCTGTCACCTGAGTTCCCTATCG
GGGCGGCCACCTTAGCCAATCGTTTTATTTCCCAGTCTGCCCCCGATAACCGGAAAAAGT
TGGCCAAGGCCAAGGACGGCCCTCAGACCCCTATCCGAGACCTGGTAAAAATGGCTTTTA
AGGTCTACCATGCCCGCGAGGAAACTGCTGAGGCCAGCCGAAAGGCAAGGCTCAAGCAAA
AGGCCGAATTTCAGGCAAGCCTCCTAAACCAGCAAACCCAGGCCTTGGTAGCGGCCCTGC
GGCCGGCGGCAGGCTCAGGGCCCCAAAACCCCCCTCTGGGGGTCTGCTTCAAGTGCGGCC
AAGAAGGGCACTGGGCCAGGATGTGCCCCAATCTGCGACCTCCTTCCAAGCCGTGCCCAT
TGTGCAAACGACGAGGACACTGGGCTAGTGACTGTCCCCAGGCCTCTTGGGCCTCGACCT
CTAGGGGCCGGGGACCAGAGAGCCCCAGGGAGACCTCCTGCCCTCCTTCGGCTTTGGAGC
TGCTGAACTTCGACGGTGACTGACGCCGCCCAGACTCAGGGACCCCAATAACCCAAGCCG
AGCCCAGGGTAACGCTCCAGGTAGCGGGTAAGTCCATCAATTTCCTACTTATTCGGCCCT
TTCCTCTTTCGAAGGCATTTTACGTCGGTTTCGGTTATGGGCATTGACGGCCAACCCTCG
TGTCCGCTCCAAACCCAACCACTATCCTGTCGGTTAGATTCCTGTCTATTTACCCACTCC
TTTCTGGTCATCCCCTCCTGCCCTACTCCTCTTTTGGGAAGAGATATACTGGCTAAGCTG
AAGGCCACTCTTCAGTTAGCTCCAGGGTCGGCTCCCACGTCAGGGGCCTTCCTAATGCTA
CTGGTTGACCCTCCAACCCCTTCTGTTAATCCTGAGGTCTGGGACACCCGAGTCCCGGTG
GTGGCTCGGCACCACCCTCCAGTCCTCATCCAGCTAAGGGACCCCACCTGTTTCCCAGCC
CGGTCCCAGTTTCCTTTGTCTACTCGCAACCTCAGAGGGCTAAAGCCCATCATCGACCGT
CTCATGGGACAGGGTCTCCTGGTCCCCACGACCTCCCCCTGTAACACGCCCATTCTCCCT
GTTCGGAAGGCTTCAGGAGACTACTGGCTAGTGCAGGATCTCTGCCTGATCAACGCGGCA
GTGATCCCTGCCCATCCACTTGTCCCTAACCCCTACACCCTCCTTTCTTCCATACCTCCT
CAGACCTCCCATTTCACAGGGATGCCTTTTTGCCATCCCACTTCACCCACATGCCCAGAC
ATCGAACCTACAGCTATCTGCTGGTCTTGGTGGACACCTTTTCAGGATGGATCGAGGCCT
TCCCCACAGCCCGGGAGACGGCAGCGGCAGTGGCAGAGGTCTTGATGGCCCATCTCATTC
CCAGATTTGGGTTGCCAAACTCTCTCCAGTCTGACAATGGGCCTGCATTTATCTCGCAGA

TCTCTCAGCAGGTGGCTGCGGCCCTAGGTATTGGCTGGCATCTTCACATCCCCTATCGGC
CCCAATCATCAGGCAAGGTAGAGCGGGTGAACGGCATCATCAAGACACATCTGACCAAGA
CAGCCGCAGTTCTGAGGCAAGCCTGTCTTGGGTCGACTTCCTTCCTCTGGCGCTCACTCG
CATTCACACCACACCACACTCCAAAACAGGTTTGACCCCTTTTGAACTGCTCTACGGCAG
GCCCTATCTCCTGACTCACCTCCCCGAGGGAGAGGCTCCCCCACTCGCAGGATACCTCCC
CCTCTTCTCCCTGCTATGATCCTTGCTGAGGGAACATGCAGACCGGGTCCTGCCGCAACC
GACAGACGATGCAGGGCCCACCCGGCCTCTAGCACCAGGAGATCAGGTACTGCTGAAAAC
CCTGAGTCCCCGCCCACTACAGCCTCGCTGGACGGGACGCCATACTGTAATCCTCACCAC
GCCTACAGCCGCCAAACTCCTAGTACACGAGCCCTGGTACCACCTGACCCGACTTAGACA
AGCTCCCCCGGGTCTCCCGGAAACAGGGGTGGCCCCGGGAAGACATTAGGGAGAGCAACC
GAGAGAGAGGACAGAAGGAGTAGGACAAAGAGAAAGGGAGACGAGGGAAGGAGAGAACAA
GGGGGAAGAGAGCAAGAGTAGAGAGGAAGTGAGAGAAAGCAAAAGAAAGTCAGAGAAAGG
AAAGAGAAGAGTTGAAGAGTGAAAAGAAGAGAAGGCAAAAGAAAGTAGGAAAGAGAGATA
GAGTGGTGGAGAAAGGGGAAGGAAGGAAGAAGGATAAAGACTGAAACTGAAAGAGTGAGA
GACGAGAGACAGAAAGGAATTAGAGAGTGTGACAGAAGGGAAAGGAAAGAGCAAAGAGAG
ACAGAATCGAAGGAAAAGAGAGAGACGGAGAGGAACCGAAGGATTGGTCCCCCACTCTTC
GACTGTCCCTAGCCCTCACATACCTGACCACCTGCTTCCTACTCCTGGAGTGCTATTCCC
TTGGGACCCCCTGACCTGGTTACCAGCTCCATCCTCACGGGACCGCCAAGGCTGGTTATC
CACCGCACCCCTTTTCACCCCAATCTCGGAACAATGCGGGGCTCTCTACTCTGGGTTGTT
CTGGGGGTACTGGGGGTCCTGGAGGAAGGGGGACACACCAACCAAAACCCCCACCAGTCC
TTTCAGATCACTTGGACGCTCCGGAATGGACTGACTCACGAGATACTCAATTTGACCACT
GGAATTCACCCCCCTAATACGTGGTGGCCAGATCTGTATTTCAACCTCAAAGACCTTATA
AAGACTTCTTGGTCAGCGACCCGGACCAGAAACTTTGGGTTCTGGGCATGTCCCGGACAC
CTAAAGACACATAATTGGGAGACTTGTGGAGGACTGCAACATTATTTCTGTTGGTCCTGG
AGCTGTGTCACCTCCAACGATGGGAGATGGAGATGGGAGGTCGGGAACCGGGACCTAGTC
AATTTCTCGTTTGTCCAACCCTATCGCGGGATTGCTCAGGTGAAAGTAATGCTCAACCAG
GAAGCGGCTAAACTGGAGAGGTCTTGGGTATCTGGGCTATCATGGGGCTTCCAACTCTAT
GCTGAGTGGTTTGAAGCAAAGCCAGGGGGAATTCTGGTTGTTAGTCAGACAATAGAACCT
GTTCAGGCCCATGCTGTGGGGCCCAACCAGGTTATTGCACCTCCCCGCCCCTCACCACGG
GGGACAAACGCCACAAATGTTGTGACCTCACCTACCCTCACACCCTCCATTCAGCTGACC
CGGACTGACCCGCTAGAGACCCAGGAACCCCTGTGGGCCTTAGTCAAAGAAACTTACAAG
GCCCTCAACCACTCCAATCCTAACACAACCCAATCCTGCTGGCTCTGCTACACCCTACAT
CCACCTTACTACGAGGCCGTGGGCTTAAGTGCTACCTACAACTTATCCATTCTGTCCAAC
CCACCACAGTGTTCTTGGGGAGACCGCAAGGTGGGCCTTACCATGAGAGAAGTATGGGGT
TCGGGGACCTGCTTAGGCACGGTCCCTACGGATAAACAAACCTTGTGCGCCCAGACTGGC
GATGATACCAACTTCGCCAATAAGACCTACGTCATACCTGACACTGGGGGATGGTGGGTG
TGCTCACAGACTGGGCTAACGCCCTGTCTCCACTTGGCTGTCTTCAACCAGAGCAGGGAA
TTCTGTGTCATAGTAGCGGTAGTGCCCAAGATTACATACCATCCAGAAGAGGTCCTCTAC
AACTTTTGGGACCAAAACACCCCAGCTCCTAGACATAAGAGGGAGCCCATTACAGCTATT
ACCCTGGCAACGCTGTTCGCCCTGGGAGCAGCTGGGACAGGCACGGGCATTGCTTCCCTG
ACCACACAACATCAGGGCTTCATCACCCTGAGGGCCGCAATTGATGAAGACATCGCCCGC
ATAGAAAAGTCCATGACGGCCCTAGAAAAGTCCCTGACTTCCCTTTCCGAAGTGGTATTA
CAAAACCGCAGAGGACTAGACCTGGTTTTTTTGCAGCAGGGGGGCCTCTGTGCAGCCCTC
AGAGAAGAATGCTGTTTCTACGCTGATCTTACTGGGGTGGTTTGTGAGTCCATGGCTAAA
GTAAGAGAGGGATTAGAGCACAGGAGGAGAGAGAGGGAGGCTCAGCAGGGCTGGTTCGAA

TCCTGGTTCCAGAGCTTCCAGAGCTCCCCTTGACTGACAACTTTGCTATCTTCCCTAGTG
GGACCGATCATCATTCTCCTGCTGCTCCTGACTTTCGGGCCCTGTATCCCAAACAAGCTT
TTGGCCTTCATCAAACAACGGCTCGACACGATCCAGCTGATGGTACACCGACAACAGTAC
CAAGGGCTTCCAACAAGCACTCCGTGGCAAGATTAACGGCAGACGCCCCCTGTCATCCCG
GCTGTACCCTACCCCTCATCGCCCCCGTTCAGCAGGAAGTAGCCAGAGAGAGTCGGCACC
CCTTGTCCTATATCAAAAAGGCCGGCATGAAAGGTCAGGGCAAGGTCAGGATGAAAGGTC
AGGGCGAGGTCAGGATGAAAGGTCAGGGCAAGGTCAGGCATGTCGGGACAGGTCCCGGCA
GAGGGCGCTGCAGACAAGCCCCGGAGACGGGCCGGCAACCAAGCCAACCAATCAGGAGCC
GACACGAAGCCCTCGCCGTCCAATCAGGAACCGACACGGAGCCCTTGGACACCAATCACC
GCTGAGCCTGCGTTTTCTTCCTTATATGGGAGCCCCGGCCCAGGCTATAAAACCCTTCCC
CACCCTCACAGACTCCGCAGACTCCCTTTACCCC
>CAP_ERV_16
CAGTGAACTTTGGACTATATGCCTGCTTGCACTTTCAGTGAACTTTGGACTATGTGCCTA
GTAGCCATAGGGATAACATACCTATGGCCTCCTGGACCGATAAAACACCAGATTCATACG
AAGATTTCCCAGTGCCAAAAAGAATGCAATAATCCCCTATGTAATCACACCTTTGTAATC
TTTATGGCACCCATTGGTGTAGGCTACAACGTATAACCAGCTGACCCTTCTGATTATGAA
TCATGGTTGTAACCCGATTGTATGTCCCTTTAACATTTTCCAGGCTAGGTTTAAGGAATT
TGGGGATGTGGGCTTGAGCATGTACACTTAAGGTATATAAGGTTTTCACAAAAGTCAGCC
GAGGACCCTGGGGTCCCTGGCCTGGGTCCCAGACCTGCTGGTGTAATAAACGACACTCAA
CTATCCACACTGTCCTTCTGAGTGAATTTGTTTCCCAAAGGGTTTGGCTATAACATTTGG
TGCATTGGCTGGGAAACTCCTCACTTTGAGGAGACAAGTCCTGTTTGGGGCCACACCAAG
GCCTTGTGGCTCAGATATTCTAGAGGGGGGAAGGTGCCTCGCCCTTCTGGAAAAATTCTC
TTTCTTGACGCCTGGACCTTCCAAGTTAAGTGGCCAGTGGATGACAGCAGGGGAATTGAG
CTCTCGGCTAAGGAAGGTTCCCACCCTGCGGGGTGGAAGAGGGGGCCTGATCAACTCCTG
AGAGAGAGCAGGAAATGCACAAACTGTAAATGTCAAGGGAGGCAGTGGAGGTAGTGTCCA
CACGGTCCTGAGTTAAATTTGTCAGGTGGTCATGAGAAGGCTTTTCGAAGAAAATTGAAT
GTCACACCGTCTTGGGACAAATTATTGCCAGACCATTGTGAGAGGGCTTTGAGGGGAAGA
AACTTGGTCTTTGTTGTGTGCTCATATTCTACTCTCCCCTAGGAGATGTCCCATCTGCCA
TGGAATTCTTAATCCCCTCAGAATTGTCAGGCTAGAAGGAGGGGGATACATAAGTGAGTA
CAAATTGGCTTTTCCAGAGATGGCGTGGGACGTGGGATATTTAACCCATCTGTGTTTTCA
TCTGCACCTGATCAACCCCACCAAGGCAAAGTGGACTTTAAAAGTCAAGGGAGAAACAGT
CTTGGACCACGTGGTGTTCTGGTTTGGCTGTGCTGACCAGCAGATGAAGACATGCCAATT
CCCCTTCTCCCTCTGGGATCTGGCAGGTAAGGCTCTTCTCACCCCAATTAGGAAGGAGGC
AGAATGGCAATTTAAATGTTTCATTGAGTTGAAATATCAGAAGTACATAGAGTACCGAGA
ACTCCGTACACAGAATGGAAAGGAAATGTAGAAGAAGGTCCAAGAAGGTAAGCAAGATGG
GAGGAAATGAATCTCAGACAACAGTACTGGAGTGCATGAGTAAAAATTTTAAGAAGGGAT
TTGGAGGAGACTATGGGGTGAAGATAACACCTAACCGCCTCCACACACTCTGTCAGGTCA
AATGGCCCTCTATGGGAGTAGGGTGGCCACCAGAAGGCATCATGAACTTAAAAACAGTGG
AAGCAATCTATACAATAGCCACAGGAGAGCCAGGACAGCCAGATCAATTTCTACTTATTG
GCTTATGGCTAGGGTTAGCTCAAGAGCCCCCTCCTTGGATGAGATTCTATATCAAGAAAG
GGAAAGGAACAATATTAATGGCACAAAAATTGACTGATAAAAAAGAAATTCTGCAGGATT
TGGACGGGGACAACTTGCCCCCTTCCCCATACTGGATGATGACGTGCCCATCACCCAATG
CTCCACCAGGACTGGAGGCCACCTTAATGCCCAATCCAAGGCCAGGTAAAGTTCCTGCAG
TGGCCACTGCTCCTACACCGACTGTCCTGGAGATCATAGAGCCACTGTTTCAGCAGCCTC
TGATCCTGGCAATGGAGACCTCTGGTCAACGCCCCCAGGATTCCAGCTCAGCCGGACCTC

CTAGACTGTATCTACCTCTCCCGGTGAGTACTGATGGGAAGGGGAAAGAAAATTAGGAAC
TAGACAGAGGCTGCACTCCACCAAGGAACAGGGGGAAAGAACCCCACTACAGATGCCTCT
CAGAGAGCTATAACAGCCTCCTGTTCAGGACGCAGGCGGGCACTACCATCAGCCCTCTGT
AGCCTATTATTACCAGCCATTTTCCTCTATGGATATATTAAACTGGCAGAGACACACTCC
ACCATACTCGGGGGAGTCACAAGTCATCATTAGGCTAATGGAGACTATTTTTCGAACCCA
GTGCCTTACATGGGATGCCATAATCCAACTACTAGCATCCCCTTTCATCACTGAGGAAAG
ACACAGGATCCTAACTGAGGCCAGAAAATGATTAAGAACTGGCGCTTGAGAGTACCAGAA
TACGATTAAGAAATGATTAAGATGGCACTTGAGCATACTGCAGCAGTGGGCAGAACTAGC
CACCCCTGATAGAGGCCCAACTGGGACTATAACACAGAGGAAGGAGGGGGGGCCACCTGG
AGAGATGTTGGGTGGCTATTTTACAAGGCCTCAAGGGGGGGCCCGAAAACCTATGAATAT
GGCAAAACCCTCCAAAGTGATTCAAACCGAATCACCCTCTGGGTTCTATGAAAGACTGTG
TGAAGCCTACAGACTTTATACACCAATAGATCCAGAGACCTCTGGGTCTCAGATGGTGAT
AAATGCAGCCTTTGTGTCTCAAGACTACCCTGATATCAGACATCAACTTCAAAAGTTGGA
CAGGGTATTGGCCACGACTAGCTTACAAATAATTGAGATCGCTGATAAGGTATTCAGAAA
TAGAGACGTGGAGTCTAAGGGGGAAGCTGAGAAAAGACAGAAGATAATATGAGGGCAGAC
CAGAGGATCGTGGTACTGGCCATGGCTTTGGGAAGGTCTCCTCTCTGAGTGGGGGCCAAT
ATCTCGGCAAGCCCTCTACAAGAAGGCCCACAGCCACCCTGCAACAAAACCAATGTGCTC
CATGCTGAGACTTTAGTCACTGGGAAAATGAATGCCCTCAAAATAATGGAGAAAAAGAAT
TGGCATCGGCTGTTATAGGGCTGGTTGGACTAGAGGCTGAATAGGGGTGCCAGGGTTCAA
AGACACAAGGTCCCCGAGAGCCCATGGTGAAACTAAAAGTAGGGGACCAAATTACTGACT
TTACGGTGGACATTGGGGCAGAAATGTCAGTGGTGACTGAACCGGTAGTACCCCTCTCAA
TAAAGGCCACTGGCATAGAAGGAGTAACCAGAGAAAAGATTATCAGATCATTCTGTTTAC
CCCAGAAATGCCAAATAGGGGGCATCAAGTGACTCATGAATTCCTGTATATTCCTGAATG
CCCAGTACATCTTTTGGGAAGAGACTTGTCATCTAAATTGGGGGCACAAGTGACCTTTCC
CCCTCAAGAAAGACCTACTTTCCGGGTGTGGATATTTACTCTATTATTTAAACTATCTAT
TTACTCTCCCTCTCAGTAACCCTTCAAGATGAATTGAGGTTACATGACCCTCTGGAAGGA
AATCTGGATGGACTAGACAGCTGAGAGAGGGAACTAATCCGATGATTCCCCAAGGTTTGG
GAGGAAAATTAACTCCCAATCCCCACACTGGGCTTGCCATACATCAAGCTCCAGTGGTAA
TAGAACTCAAACCCGGTGCCATCCCAGTCAGAAAACATCAGTACCCATTGCCACTAGAGG
CTCGAGTTGGCATTTTGCCACACATAAGCAGACTGAAACAGGAAAGCATCCTGATTGAAT
GCCAGTCGGCCTGGAACACACCAATTCTACCAGTCAAGAAAGAAGGGGGACAGGACTATA
GACCTGTGCAGGACCTCAGACTGGTCAACCAGGCCACTGTGACTCTACATCCCACCGTTC
CTAACCCCTATACTTTAACTAGCCTCCTTCCACCGAGTGCTAATATCTATACTTGTTTAG
ATCTCAAGAATGCCTTCTTCTGTATATGCCTTGCCCCAGTGTCTCAACCCATTTTTGCTT
TTGAATGGGAAGACCTAGCAGGGGGCACCAAACAACAGCTCACCTGGACTCACCTCCCAC
AAGGATTTAAGAATTCCCCAACCATCTTTGGGGAAGCCATGGCTTCCAAACTGGACTCAT
TCCAGCTAGAGTTCAGATGCTGGTTGCTACAACATGTGGACGACTTGCTGTTGGCTGCCA
AGAACAGCGAAGAGTGCTGGGAAGGGACCAAGGCTTTACTAGAGCAGTTGATGGAGTCCG
GCTACCAAGTCTAGAAAAAGAAGGCACAGATCTGCAAAGAGGAGGTAAGATATTTGGGGT
TTGTTCTGAGAGGAGGGACAAGGCTGTTAGACCAGTTCAGAAAAGAGGTCATTTTGAGAA
TCCCACAACCAAGAACCAGATGATGGGTCCAAGAGTTCTTGGGAGCCACTGGGTTTTGTA
GGATTTGGATTCTGGGCTATTCCAAGACGGCTCAGACGTAATATGAACTCCTAACGGGAT
CAGAAGGAGATTCACTAAATTGGACTGCTAGACAGCAACAGCCCTTTGAGGAATTAAAGC
TGGCAATCACATCAGCACCCGCCCTGGGCCTGCCAGACCCTAAGCCGTTTACTCTTTATG
TGACTGAAAAGGACAAGGTGGCTATGGGAGTGCTGTCCCAGACTATGGGGACATGGGACA

GACCCGTGGCTTCTCTCTCAAAACGGCTGGACAATGTTGCCACCGGGTGGCCGGGATGCT
TATGGGCAGTTGCTGTGGTTGCCTTACTGGTCCAGGAGGCAACCAAGCTGACTTTGGGCC
AAGATTTGATCATAAAAGTCCCCCATGAGGTCAACACTCTCCTGTGAGGGGACCCCCATA
AATGGCTGTTGACATTCTGGAATACTCAATACTAGGGACTGTTATGTGAGAACCCTCATG
TTACTATTGAGCCTCGTCAGGCCCTGAATCCAGCCACTCTCCTTCCTGAGGGAGAAGGTG
GGCCCTCACATGATTGCAAGGAAATCCTAGAAGAAGTTTATGCCAGCAGACCTGACTTGA
GAGACCTGTCAATCCTGGACCTGCCTTGGGTTCTGTACACCAATGGCACCAGCCTGATGA
AACAAGGACAGTGACTGTCAGGATATGCAGTAGTCACAGAAGAAACCATCTTTGAGGCTA
GCTCTCTGCCGTCACCGGTCTGCTCAACGGGCCAAACTATATGCTCTAATCCGGGCCCTC
CAGCTGTCAAAAGGCAAGAAGACAAACATTTGCACAGACTCCAGGTACGCTTCTGGTACT
CTGTGTGTACATGGGGATTATATGAAGAGAGACGTCTTCTGACAGCCCACAGAAAGGACA
TTAAAAACAAGAAATCTTGACTCTATTAGATGCTGTATGGGAACCTGAAAAGGTAGCAGT
GATACATTGCTGGGGTCACCAAAAGGAAGACACCCACAAGCACAGGGAAACTGACTAGCA
GATAAAAGTGCAAAACATGTGGCTGAGAAATTTGGGGCTGCTGGTGGGGGACCTCAAAAA
TGCCTGAGTTAATGTTGATTCTCCCACAGTATACCCTGGCCCAAGATCAGCTGGCTGAAG
CAGAAAGGGCCACCATGAATGAAAAACGTTGGTGGGAACTGCCAGATGGCAGGTTACTGG
TACCCGAGGCATTAGCCCCCCATACTGGTGTCTCAGGTTCACCAGGCAACCTACTTGGGA
CATGACAAAATGGAAGAATTAATTCAAAAATATTTCTTAATTCCATGACTTTCCTCCCTA
TGTAGACTGTTCTGCTTGCTCAGATGTCAATGCTGCCCTTCGACATAAACAAAAACTTCC
AGGAATACAGTTAAAAGGCACTCTACCCTTTGAACATTTAGAAGTGGACTTTACCGAGAT
GAAACCCTGCTGACACTATCACTATTTACTGGTCATGGTACGTACCTTCTCAGGATGGGT
AGAGGCCTTCCCCACTTGAAATGAAGTAAATGAAGTGGCTTGCTGTCTGCTCCGAGAAAT
AACCACCAGATTTGGGTTCCCAACCAGTGTAGGATCAGACAACGGCCCTGCCTTTGGTAA
CCGACTTAATTCAACAGGTCTATAAAGGTCTAAATATCAAGTGGAAATTACATATGGTGT
ATAGGTCCCAGAGTTCCAGAGTGGTGAAAGAACCAACCAAACTCTTAGACACTTTCAAAA
TGAATCATAGAGACTAACTGTTCATGGGTAGACTTGCTTCTGGCAGCCTTACTCAAATTA
AGGGTAACCCCGCATTCCCAAGGTTTTTCTTATGAAACTGTCTATGGGAAGCCCTCTCCC
ATAGTAAGACTGGTTTGGGCGAATCTGCCACAGGTAAGGGCAGATGGGATTTCACAGCAG
ATGGAACAGCTGGGTCAGGTAATAAATCAGGTAACTAAGTTTGTACAAGAAAGATTGCCA
GTCCCCCTTGGGGAACAGATTCACGAATTTGTGCCCAGTGATCAGGTGTGGGTCAAGGAC
TGGAAACACGACCGACTCCTTGGCCCCTCATTGGAAGGGTCCGTATACTGTTGTTCTAAC
CACCCCTACAGCCTAAGAGCTATTCAAGCACTAAATACTGAACAAATACAAATGAGAAAA
GTGGTAATTCAAAACAGAATGGCTTTAGACATACTTACAGCCACTCAAGGAGGGACCTGT
GCTATAATTAAAGCCAAATGTTGTGTATATATTCCTGATTTGTCTGGCAATATATCAGTC
GCTCTAGATGACATGAAGGATCAAGTAAAAGCTATGTATGATGAAAATCTTCCTTTTTGG
ACTTCTGTTCTATCATAGGTAAAGGGTGATTGGTAGAAACCTATATTAACCATTGTTACA
GTTGTTCTGTTGATTCTGTTACGTGGACCTTGTATTCTCCAATGTGTTGTTAAGTTTGTA
TCACAGAGGATGACTTCGTTTACCCAAATATTTACCAGGAAGCCTAAAATGCAGTACATC
TCAGTAAATGATGCTTGCACTGGAAGTTGAGAGCATCAAGAGGGGAGAATGAAGGAGGAA
AGCAACAGGCCGAGCTGACTCCATTTTGAAAAAGAAGCAAACTACACCTTACATTCCCAG
TGAACTCTGGACTGTACGCCTGCTTGCACTTTCAGTGAACTTTGGACTATGTGTCTAGGA
GCCATGGGGATAACTACCTACTACGGAACTGGCCTCCCGGACTGATAAACACCAGATTCC
ATACCAAGATTTCCCAGTGCCAAAAAGAATGTAACAATCTCTTATGTAGTCAATCACCTT
TGTAATCTTTATGGCACCCATTGGTGTAGGCTACAGCATATAACCAGCTGACCCTTCTGA
TTATGAATTATGGCTGTAACCCAATTGTATCTCCCTTTAACATTTTCCAGGCTAGGTTTA

AGGAATTTGGGGATGTGGGCTTGAGCATGTACACTTAAGGTATATAAGGTTTTCACAAAA
GTTGGCTGGGGTCCCTGGC
>CAP_ERV_17
TACACATGGGGTATAAAAGATTTTCACAAATGCTGGTTGAGGTCCTTGGCTAAGAGGAGA
CTCTGCCCTGGGCCCGCCGGTGTAATAAATTGCACTCCACTATCTGCATTGTCCTTCTGA
GTGAGTCTGTTTCCTGGAACGCGTGGCTATAACATTTGGTGCTTTGGCTGGGAAACTCCT
CACTTTGAGGAGACAGGTCTCATTTGAGGCCACCCCGAGGCTTTGTGGCTTCAATCTCCT
AGAGGGGGGAAGGCGCCTCGCCCCTCTGGAAGAATTCAGCCTTTCAACGCCTGGTTTCTT
CGCTTTGGCAGGTAGTGAATGGCAGCAAGGGAACTGAGCGATCAGATGAGGATGAACCCA
CCCGGCAGGGTGGAAAAGGGGGCCTGATCACCCCTCTGGGAGGGACTAGAAGGGGCATGG
GCCCACAGGGGCCTAGAATAGGCAGGCAGCAGCGATTGCTTGGTACATAGATCAATGAGC
ATGCTACGGTTTAGGAAGGTAATTTGTGAAGGTCATTTAGGAGGTATGTCCACGCCGTCT
CAGGGAATATTACTACGAGCAATTGCCAGGGGATTTTTAGGATAGGAAACTCGTCCTTGT
ACGCTCATATTCTGCCCTTCCCCAGGAGGTGTCCCATCTGCTGTGAAATTCTTGACCCCC
TCGGAATTGTTAGGCTAGAAGGAAGGGGATACATAAGTGAGTATGAATTGGCTTTCCCAG
AGATGGCCTGGGACATGGTATATTTAACCCATCTGTGTTTTCATCTGCACCTGATCAAGC
CCACCAAGGCAGAACGGACTTTATGGGAGGAACAGTTTTGGACCACGTGGTGTTCCGATT
TGGCCATGCTGACCGGCAGGTGAAGGCACACCGATCCCCCTTCTCCCTCTGGGATCTGGC
AGGTAAGGCTCTTCTCACCCCAAATAGGAAGGAGGCAGAATGGCAATTTAAGTGTCATTG
AGTGGAAATATCAGAAGTACAGAGGGTACTGAGAACTTCGTAGACAGAATGAAAAGGAAA
ATTAGAAGAAGGTTCAAGAAGGTAAGCAAGATTGGAGGAAGTGAATCTAAGGCAACTGTA
TTGGAGTGCATGATTAAAAATTTTGAGAAGGGATTAGGAGGAGACTATGGGGTGAAGATG
AAGCCTAACAGCCTCCACATACTCTGTGAGGTCAAATGGCCCCCTATGGGAGTAGGATGG
CCACCAGAGAACACCATGAACTTAAAAATAGTGGAAGCAGTCTATACAGTAGTCACAGGA
GGGCCAGGGCACCTGGATCAATATCCATATATTGACTCATGGCTAGGGTTAGCTCAAGAC
CCTCCTGCTTGGACAAGGTTCTGTATCCAGAAGGGAAAGGGGAAAACATTAATAGCACAA
AAATTGACTGATGATAAAAAAGGAAATTCTACAGGATTTGGACGGGACGACCTGACCCCT
CCCCCATACTGGATAATGACACTCCTGCCTCCGTGCTCCACCAGGATTGGAGGCGGGCTT
AATGCCCAGTCCAGGGTCAGGTGAAGTCCTGCAGCAGCCGCTGCTCCTCTGCCAGCTCTT
CCCAAGGTCGTAGAGCTGCCACCTCAGCAGGCTCCAATCCCAGTGACAGAGCCTCTGGCC
AGCACTTCCAGGATCTGGCTCCAGCTGAACTGCCCAAGCTATACCCGTCTCTCCCTGTGA
GTACTGACAAGAAGGGAAGGGAGACATTGGAATTAAGCAGAGACTGTGCTCTACCAGAGA
GCTGGAGGGAATGGAAGAGACCAGACAAGAGATTTGCAATGCTAGCTGCTGCTCTGGGAA
AGTCTATCTCGGGCCCTCCGGAAAACCTCCTCTGCCCCAGGGACAGGACAGTCCTTCAGC
CGGTCCCAACGGAGGCCCCGGGCCCCATTACAGCCAAACCAGTGTGCCTGGTGCCAAGCT
TTTAGCCACTGGAAGAATGAATGCCCTAAGGCAAGAAAGGAAGAAGAAGCTCCCGCAGTT
GTGAGGCTTGCTGACTTGGAAATTAAATAGGGCTGCCAGGGCTCAGAGATATCAGGTCCC
CGAGAGCCCATGGTAACCTTAAAAGTGGGGGACCAAAACATTGACTTCATGGTGGATACA
GGAGCAGAACTGTAGGTAGTAACAAAATTTGTGGCACCACTGTCCAAAAAGACTACCGCT
GTAACTGGGGTATGGGGAGAAGACATGATTAAATCGTTTTGCCAGCCCAGAAAATGTCAG
ATGGGGGGCACCAAGTGATTCACGAATTCCTCTACACTCCTGAGTGCCCAGTACCCCTGC
TGGGAAGAGACTTGCTCTCCAAACTGGGAGCACAAGTGACTTTCCCCCTGAGGAGAGGCC
CATCTTCTGGATGGACTATGACTTATTTGCTCTCTCTCTCAATACCACCCCAAGATGAGT
GGAGGTTGCATGAGCCTCCGATGGAAGAACCAGGTGGGCCGGAAGAGCATGAGAGAGAGC
TAACTCAATTATTCCCTGAGGTCTGAGTGAAAGACAAACCCTGCTCCCCACCCAGGCTGG

CTAGACATCAAGCCCCAGTGATAACAGAACTCAAACCAGGCACCACCCTGGTTAGAAAGC
ACCAGTACCTGCTACCGATAGAGGCCTGGACCGGCATACTGCCCCATATCAATAGATTGA
AACAAGCGGGCACTCTAGTAGAGTGCCAGTCGGCTAGGAATACAGCGATCCTGCCAGTCA
AAAAGGAAGGAGGATAGGACTATAGGCCTGTACAGGATCTCAGGCTAGTCAACCAGGCTA
CTGTGACTTTACACCCCACTGTTCCAAACCCCTATACCTTTTAGCCTCCTCCTGCCGAGG
ACTAAAGTTTATACTTGCTAGATCTCAAGGATGCCTTCTGCGTACTCCTCGCCCCAGCGT
CACAGCCCATCTTTGCCTTTGAATGGAGGATCCATTCGGGGGCACCAAGCAACAGCTCAT
CTGGACTCCCCCACAAGGGTTTAAGAACCCCCTAGCCATCTTTGGGGAAGCCTTGGCTTC
TGACCTGAACTCATTCCATCCGGAAGACTACGGATGTTGGCTCCTACGTTACGGGGATGA
CCTGCTGCTGGCCGCCGAGACCAAGGAAAAATGCTGGAAAGGGAAAAAAGCACTGCTCCA
GCTGCTGATGGAAGCAGGTTACTGGCTGTTGAAGAAGAAGGCACAGATCTGCAAGGAGGA
GGTAAGGTATCTGGGGTTTGTTTTAAAGAAGGACACAAGGGTCCAGACCCTAGTTGGGTC
CTGTATACTGATGGCACTAGCCTGATAAAACAAGGACAACGGCTGTCAGGTTAGCCAAAG
CGGAAGGGGCCATTAAGACTGAAAAGTGATGGTGGGAATTGCCAAGTGGCAAATTATTGG
TACCAGAGGAGCTGGCACACAATCTGGTAAGCCAAACACACCTAGCGACCCACCTAGGCC
ATGCTGCCTGCTCACAGGTTAATGCTGCCTCTCGGGTATTCAGACAAAAACCCCTGGGTA
TTTAGCGGAAAGGCACGCTGCCCTTTGAACACCTGGGAGTGGACTTCACTGAAATGAAAC
CTCACCGACACTACCGTTACCTGCTGATCATGGTATGTACGGTCTCGGGATGGGTAGAAG
CTTTTCCTACCTGGACTGAAAGAGAATCAGAAGTAGCCTGGTGCCTGCTTAGGGAAATAG
TTCCCAAATTTGGACTTCCTACTAGCATTGGTTCAGACAGTGACCTGGCTTTTGTAGCTG
ATTTAGTACAACAAGTAAGCAAAACTTTAAACATCAAATGGAAACTGCACACTGCATATA
GGCCCAGAGTTCTGAGATGGTGGAATGAACCAACTGGACATTAAAGAGACTCTCCAAGTA
GACCACAGAGACTGACTGCTCCTGGGTGGACTTGCTTCCTAGGGCTCTGCTCAGACTCAG
GATGACCCCACAGTCCCAAGGCTATTCTCCATACGAAATTGTAGTAGGAGGCCCCCTCCC
ATAATAAAACAGGTATCAACCAATTTGCCTCAGGTAAGGGGGAATAGGATTTCACAGCAG
ATGGAACTGGGTAAGGTAATAAATTGGGTAACTAAGTTTGTACAAGAGAGGGTGCCGTTC
CCCCTCGGGAACAGATTCATGAGTTTACGCTTGGTGACCAAGTATGGGTCAAAGGTTGGA
AACATGATTTACTAGCCCTTTGGTGAAAGTGCCCTTATGTTATTCTAACTACCCCTAGTG
CAGTTAAAGTTGCAGGTATTGTCCCTTGGATCAATCATATGAGGGTGAAGAGAACATACC
ACGCAGACCCAAAAAACACTGAATGGACTGCACAGAGGGACCCCGCTGACCCTCGAGAGA
CTAAGACCATCCTTAAGAAGAAGGAAAAGAAGATCCTGGAGGAGCCCCTTCAGGATGCAG
CCACACAACCAACTCCTGCTGCTTGGCCTCACCAACGTGATTTTGAATTTAACTTCCGTT
TCAACTCAGGACAATGCTTTCATCTCATGGGCACATTCCCACGCAGACTTCCACACTCCC
AGCTGCTGGGTGTGTAGGGCTGTGCCTCTGTCAGTGACGGATGGACTTCCTTGGTGGGTG
TCACCGCTCTGCCAAGGAGATTTTAAGCCACTCTGCTCTTTCTTGGATGACAAAAAGAGA
CCTTCCTCTCTTTTGTCAATAACCTCTCCTTGCTCTTTTGATGTAAGACCTACACTCAAT
AGACTCAGGTCATGGGGTTACGTTTGATATAAATGCCAGTGTAACAAAAGCCTAACCTAC
TTGTAAATCTACCCCGGTAGCCCTGGTAAATCTACCTTATTTACATGCAAGATGGACAAG
ATCCATGTTTCAATGATATGAGTATATTGCTGCCTTATTCGTATCCTCTATAGGGACAAC
AGATATCAATGATTAAAGCAGAGGCCTTGACTAACTTCACACAACAGGCCCTCCTAGATA
GAACAAAAGCCATCCAAGCCTTAAATGAAGAGCAAATCCAAATGAGAAAAGCAGTGATTC
ATAATAGCATGGCTTTGGACATACTCACAGCTGCTCAAGGAGGGACCTGTGCCATAATTA
AGGTTGAATGTTGTGTATACATTCCTGACTTATCTGGCAATGTATTGGCTGCTTTAGATG
ACATGAAAAACCAGGAAAAAGCAACGTCAAATGAAAACATTCCTTTCTGGACTTCGGTTC
TATCTTGGGTGAAGGGTGGAAAACTGTATTTACCACTGTTATAGTTGTCTTGATAGTTCT

GCTTTGTGGACCCTGAATTTTACAATGTGTTATGAACTTAGTAACCCGAAGGTTGATGTC
ATTCTCCCAAATTGACGGTCGGAGAGCCAGGGTGCAATATATCCCTATGAATGTGCTCAT
AATGTGAGTTAAGAGCATCAAGACGGGGGAATGAAGAAGGAATTCATAGGGCCTGGACTC
CATCTTAGGCCTGTTCATGCTGATCATGCTCAGCCACCTTTCCAATGGGCTCTGAACTCT
GTGTTTAGTGCCTATGAAAACAACAACAGAAGGATAAGACCCCCTCCAGACAGGGGAACC
TTGAAGATCGTATCTAGGTTACTCATCGCCTAAAAGAAAACATACACTAATCACCCTTCC
TCCAGACAGGCCATAAATTTTTCTGTGTCTATCAGAGTGTAACCTCAGATTTATTGATTA
TTGGCTAATTGTTTGACTGTTTGAGCACATGAGCACATAGCACGTGAATGATGGGTTATT
GGGATTGTATTTTCCTTGGTTTATGAAAGTCTCAAGGAATTTGGAGTGGTGGATTCAGAC
ATGTACACATGGGGTATAACAGATTTTCACAAATGCTGGTCGGGGTCCTTGGCTAAAAGG
AGACTCTGCCTTGGGCCCGCCGGTGTAATAAACTGCACTCCACTATCTGCATTGTCCTTC
TGAGTGAGTTTGTTTCCTGGAACGTGTGGCTACAACAATT
>CAP_ERV_18
AATGGCTCATGATGTTGACCTCGCAAACAAAGAATAAAATCACAGTGACCCTTGAGACTG
ACCAAATTGCCCAAATGGCACACTGTTTTCCCACTGGCACCTACCTTCTCAAAGCTGCAA
GACCACAGGATTCCAGACCTCTGGCTACGAGACCACAGGACTCACCTACAGGCTAAAAAT
TAACTGTCCCTTCAGAGAATTCTTCATCGGCAGATTATAAGAAAGACCCCATTGGAAAGG
GAGGACACTGGTTCTGCTAAGGGCCAGTCACCTTCTTTTTTCCCTCTAATAAATTTCCTT
GTCTTGCCTGACTGCCCAGCTGGCTCTGTTTTTCTCTGCACTCATCTTACATTCTGGTGC
TGAAACCTGGGACGGAAGGTCCACCGCAGCTGGGTGGTGGGCTCCTCTCACAAGCACACC
TCCGGTGGTCCCTTCTGTTGGACCTGGCCGAGAAACTGAGATGAGCTCCGGGATGGGACC
ATCCACCTGTCTTGCTGGACTGACATCCACCCACCAGCCCACATTTCATCCATCTGTTTC
AAGGGGGAGTGAAATCCCATTCCCTTGGATCTTCTCTCTCTCTCTCTCTCTCTCTCTCTC
TCTCTCTCTCGCCTTGTTTCCAAGCCCTAGCGCTAGGCAGGAGGATCTCCATGGCCCTCG
GGCCCTCGGCCTTGCGCTCCATCTGACTTTGAAATTGGGGGGCACCCATTTCAAAGCAGA
CTGTTATTACTCTCTGAGAAGGTCCTGAGAACTGGGATGCCTTTTCTCTCGGCCCTTTCT
CCTTGCTCTCCCTCACCCTTGTCTACCCTTCCCATTCGGCCACAATGGGAAATTCTCAGT
CCCAGCCCTCAAAATCTGCTCCTCTAGGATGCCTTCTCTGAGACCTAAAAGCCTTGGGCT
TCCAAGGGGCGATTGGACCAAAAAGACTTATTTACTATTCTAACACTGTCTGACCACAAT
ACACTTGATAATAGGTCCCAATGGCCAGAAAACAGAATTCTGGATTATAATCCTCTACGG
GACATCAGTAACTTCTGCCGCCACAACAGCAAGTGGTCAGAGATCCCCCATGTCCAGGCT
TTCTTCGCTTTCCGCTCACGACCCTCCCTCTGTGAATCCTGTTCTACTTCTCAAATACTC
TTAGCCCACTCTGGGCCACATCCTCCTCATACAATGTCTCCTGATCCCTGTTCAGACTTT
TCTTCTTCTTCCTTCGATCCTTCTGACCACAGTCCACCCCCTATCGCTCCCAATCCCCTT
GCAGCTGCTCCAGACCCAGTTTCACAGCGTCCACCTTACGCCCCTTCCTGCCTCCCCCCT
TCTCAAGCACAGGCCTCCACCACCACTCCCAGCCCCACTCCCCTGCCCTTGCCTGAGATA
AGCTCTGAGACCTCAGCCCCACGCTCTACCGCAGGGTCCCAAGGCTTTACCCTGACCTCC
CTAGATCCCCTCCCATATCCAGTCCTGAACAAGCAGGAAACTTTACAGCAGGACCCCTCG
CCTTACCCTGCTCCACTCCTCCCTGTATGGGAAGCAGCTGGAGCAGAAGGCATTGTTGGG
GTTCATGTCCCATTCTCCCTCACCGATCTATCTCAAATCGAAAAGCAGCTTGGCTCTTTC
TCTTTAGACTCTGATAATTATCTGAAAGAAATCAAGTATCTTACCCAGTCTTACAACTTA
ACCTGGCATGATATTTACATCATCCTTTCCTCCACTCTTCTCCCAGAAGAGGAGGAATGA
GGATGGCAAGCCTCTCAGGCACATGCTGATGAGATACAGAGGACAGACGACACTAAGCCC
ACAGGGGCCATGGCTGTCCCCCGAGATGATGCCAACTGGGGTTTTCAGGCAGGGAGACCT
GGAGGAGCAGCCCGTAGTCACATGGTTGCTTGCCTCATCACGGGCCTTCAAAAGGCAGGA

CATAAAGCCGTCAACTTTGATAAGCTCCAGCTAATAACTCAAGGACTAGATGAAAACTGG
GCACAATTTCTGGCCAGGTTAATGGAAGCCCTACAAAAATATACAAGATTAGACCCCACT
TCAACAGAGGGCATCATTGTCCTTAACAGCCATTTTATCTCCCAGTCATCCCCAGATATC
TGCAAGAAACTAAAAAAGGCAGAAGGCCCTCAAACCCCTCAATGAGACCTTTTAAATACA
ATTTTCAATAACCAGGAAGAGAAGACAAATCTAGAGAAGGCCCAGGGTGATCAGGTTAAA
TACCGCCTATTAGCCACTGCCCTACATGGCTCCACATTTCCACCAATCAACAAAGACAGG
AAGCCCCCTGGGCCCTGCTTCAAATGCAGCAAAGATAGTCACTGGGCCCTCATGCCTAAA
CCAAGGCCCCCTCCAGGTCCGTGTCCCAGCTGTGGCATAAAGGGACATTGGAAGGTCAAC
TGCCCAAATCCCCCTCCAGGGATCCGGACATCTCCTCCTGGTCCCGAGCAGGAGTCCTCC
GACCCAGCTCTGCCCAGCCTCCTTGGACTTGCTGCTGAAAACTGAAGGTGCCCAGGGCCC
CGGCTCTCATCACCTCTACGGAGCCCAGGGTAGCTTTCTTAGTGTCAGTTAAGCCGATCT
CTTTTCTCATTGACACGGGTGCCGCTTACTCTGCTGTGCCTGCCTACTCTGGAAACCCCA
AGGTCTCTCAGGTCTCTGTTACAGGGGTTGATGGTTTAATATCTACACTACGAACAACCG
AGCCTCGACTTTGCACACTTCAGGGTACCCCATTTTCCCATTCTTTTCTCATACTCCCAA
AATGCCCCACTCCTATTCTTGGAGAGACCTGCTCTCAAAATCCAAAGCCTCTATTACTGT
CCCAGGCCCATCTTCTGATCTAGCCTGGCTACTGCTCCTCAACCCCACCTTCTCTTCCCC
CACCCCATTGCCCTCCTCGTCCATAAACCCCATAGTTTGGGATACAGACAACCTATCTGT
CACCTCTCACCATGCTCAATTCATATCCGCCTCAAAAATCCCTCTAAATTCCCCCAATCA
CCCACAATATCCAATCCAAAAACATCAACAAGGGTTAAAGCCTATCATCACCAAACTCCT
ACATCAGGGTCTCTTGTGCCCAAGCTACTCTCCCTGTAACTCCCCTATCTTACCCATCAA
AAAGCCAAAACGGCTCCTATTGCCTGGTCCCAGGCCTGAGAGTTATCAGTGTGGCTGTCA
TCCCCATATACCCAGGAGTTCCAAATCCCTATACTCTTCTCTCGTTCCCTCTTCCACCAC
CCACTTCACTGTTCTACACCTCAAGGATGCCTTTTTACCATCCCTTTACACCCAGACTCT
CAAGGCCTCTTTGCCTCTATCTGGACCGATCCAGACAATCATCGCTCCCCACAGCTGACA
GGGACAGTCCTCCCACAAGTCTTTCGTGATAGCCCTTATTTCTTTGGCCAAGCTCTAGCA
TCAGAACTAACCTCTCTTGACCTTCCTCTGAGTACTGTCCTCCAATATGTGGATGATCTC
CTCCTTTGTAGCCCTTCACTTACACACTCTCAGCAACACACTGCACAACTCTTCAATTTT
CTAGCCAATCGAGGCTATTGAGTATCTCCTACCAAGGTTCAGCTCTCTCTTCCTAGAGTC
ACTTATCTTAGAGTCCTTTTATTACGAACCAAAAGATGTATTACTACTGATAGAAAGTCC
CTTGTATCTACCCTACCACTCCCTACATCAAAAACAGATCCTGTCCTTCTTGGAGTTAGC
TGGGTATCTACACTTCTGGATTCCTAATTTTGCCCTCTTGGCACAACCCCTGTATCAAGC
CACTCAAGGAGATCTTTTAGAACTAGAGATAAAATCAAATATCCGTTCAGCCTTTAAAAC
CCTTAAGCAAGCCATTCTCTCAGCCCCAGCTCTCACACTTCCTGACCTGTCTTGCCCCTT
TATACTCTACTGAAAGACACAAAATTGCCCTAAGAGTTTTGAGACAAAATTAGGGCCCCT
CCTTCACCCCTGTTACTTATCTGTCAAAGCAATTAGATGCCACTATTCGAGGACGGCCAG
CCTGCCTACATGCTCTGGCAGCAGCTGCGCTCCTCTCTCAGGAAGGTAAACAACTTCCTT
TTGGAGCACCCACGGTCATTGATTCACCACATGACTTTAAGGACTGTCTCACAAATCCAT
GACCCTTCTATCTCCTTCACACATTCAGCTAATTCATGTCACCCTTCTTGAATCTCCCGA
GTTTTCCTTTGAACTCTGTCCTACTCTCAACCCTGCCACCCTTATCCCGCATTCTTCTGA
GCCTCCCACCCATACTTGCAAAGAGGCATTAGAGGACCTGATGCCCCATTTCTCCCACAT
TTCCTCAATACCTTTAAATAACCCTGACTTTACTTGGTATATTGATGGCACTCCTCTACC
ACATCAGAAGGAAAAAAGGCAGCTGGATGTGCCGTTGTCTCTGACACCGAAATTATTGAA
CCCCAACTTGGCCTCTCGGGAACCTCTTCCCAAAAGGCAGTACTTATTGCACTAACTAGA
GCGCCTTCCCTCGCAGCTAACAAGAGAGCAAACATATACACTGATTCTAAGTATGCTTTC
CACATCATACACTGACATGCTGCCATCCAGAAGGAGCGAGGGCTCCTATCTGCCAAAGGT

TCTCCCATAACTAACACCCTGCTCATACTCCAACTTTTAAAAGCAGCTAATATGCCAACT
GAAGTAGGCATCCTACATTGCCAAGGTCACCAGAGGGCCTCAGACCTCATTTCATGGGGC
AACAACACCGCAGACAGTGAAGCAAAACAAGCCTCACTCCAATCACCAGCTCAACAACTT
ATAGTAATCTCCAACATAAAACCCCTTCACCTCCCCGAAGACACACGATTGTTACAAGAG
GAAGCACAGCCACAAGGGGACTGGGTACAAAACCAGGGCTGCCGTGTCCTTCCCCAACCT
CAGGCCACACCGATTCTTACAGACATTCATCAGGCCCTACAGGTAGGCACTAAACCTCTT
CATCACCTCTTAAGACCCCTTATGGCGTCACTGACTCGATGACCATGAGTCTGAGCAAGC
TCTGGGAGTTGGTGATGGACAGGGAAGCCTGGCGTGCTGCAGTCCATGGGGTCACAAAGA
GTCGGACACGACTGAGCAGTTGAACTGAACTGAAGACCTCTTATCACCTATCCTAACATT
CTTTCACTTCTATACGTCACTCACTCTTGCATTATCTGCTCCCCAGTCTTACCACAGGGG
GCCCTAAAGCCTGTTCTCTCACTCTCAACATGTCAGTCTTGAGGGCATATCCCAGGAGAG
GACTGGCCAATTGACTTCACGCACATGCCTCCAACACGAAAATTAACACTCATGTTCACT
GTCAGAGACACTTTCTCAGGGTGGATTGAGGCTTTTCCTATGAGATCAGAAACTGCCTCA
GAAACTGAGTAACACTTGAGTTTCTCATATGAGAAGTCATCCCTTGCTTTCATCTCCCAC
TCTCTCTCCAATCTGATAATGGGCCAGCTTTCATTTCCCAAATCCCTCAACAAGTAGCCC
AATCTTTTGGCATAACCTGACAACTACATATTCCTTACAGACCCTCCCTATCCCACTAAT
TCATAACCGACCGAAATGGGCAATTCAATTCATTCCTCTATAAATAAGTATAGGAATAGC
AGCTGGGATTGGAACAGCGACTGCAGGACTCACAGCCTCTTTAAATTAGTACCGAAGCCT
TTCTAAAGACCTCACTGAAAGCCTAGAAGAAATGGCTACTAGCCTTATCCCTGTCCAAAA
CCAGCTAGATTCCCCGGCAGTCGTGGTCCTCCAAAACAGAAGAGGACTAGACCTTCTGGC
GGCAGAAAACAGGGGCCCGTGTCTATTTTTGGAGGACGTGCGCTGCTTCTACACCAGCAA
ATCAGGCGTTGTAAAGAAAGAAAGTGAAAGTTGCTCAGTTGTGTCCCACGCTTTGCAACC
GGACTATTCAGTCCATGGAATTCTCCAGGCCAGAATGCTGGAGTGGGTAGCCTTTCCCTT
CTCCAGGGGATCTTCCCAACCCAGGGATCGAACCCAGGCCTCCTGCATTGCAGGCGGATT
CTGTACCAGCTGAGCCACTGAGTTGTAAAGGAAGCAGCAAGAAATCTGACAGAAAGAGCC
TCTAGAGCATATAAACACCCCAGTCACTCATGGGAAAACTGGCGAAGCAATTGGAATTGG
CTGCCCTGGGTCCTACCTTTGCTAGGCCTTCTTCTTCTCCTTAGCCTTATTTTAACTTTT
GGCCCATGTTTAATACATCTTTTTTCAAAATTTCTTCAGGACCGCTAATGAGCATTTACC
AACTGAACTACCCATAAGCTACTTCTAGCTCGCTCAAACACTTTGACCCTACACAAATCC
CCTTGACTCACATTCCAGACTTTTCTCATCTCACCCCCATGTGCCCCCGTCCTGCAGAAA
CCAGTTAGGTAAAATTGACCTTAGGCTCTTATCCCAAATCAAAAAGGCTGAATGTTAGAG
TCTATTTGGGGGTTCATGGTAGAAATGGCTCATGATGTTGACCTTGCAAACAAAGAATAA
AATCACAGTGACCCTTGAGACTGACCGAATTGCCCAAACAACATTCTGTTTTCCCACTGG
CACCTACCTTCTCAAAGCTACAAAACCACTGGATTCCAGACCTCTGACTATGTGACCACA
GGACTCAACTACAGACTAAAAATTACC
>CAP_ERV_19
TATGGTGATCTGCCCCTCTTCAAGATTCAAGTTAATCATTTTATGGCCCAAGATAAACCA
TTTGGTGCCAAGATTATCCCAAAATGCATCTTATTGGTGAGGGGCCTGGTGCCATTCTAA
GTTTTGAGACATTCCTTTCTTTCATTAACAGGCTGCTTGTGACTATATAACATCCAGCTG
AAGACTAGCAGAGGGGTACTCTTTCTACCCCCTTCTGATGCCTACGTCAGAAACTTTCTC
TATCTCCTTTATACTTTAATAAAACTTTATTACATAAAAGCTCTGAGCGATCAAGCCTCA
TCTTTCGCCTGGAATTGAATTCTTCTCCTCCAGGAGCCAAGAATGCCGGCGTCGTAATTC
AACAAAAACCTTTCATCTTGGGGGCTTGTCCGGGATCCTTCAGGACAAGGTAAGGATGCT
TGGAGCTGTAGTTCTTTGTTCTCTTAGTGAACAAGTTTTCTGCTGCGCTTAAGCAACACT
ACGAACAAAGCTAATGGAGGTGATGGAATTAAAGTTGATCTGTTTCAAATCCTGAAAGAT

GATGCTGTGAAAGTGCTGTACTCAATATGCCAGCAAATTTGGAAAACTCAGCAGTGGCCA
CAGGACTGGAAAAGGTCAGTTTTCATTCCAATCCCAAAGAAAGGCAATGCCAAAGAATGC
TCAGACTACAGCACAATTGCACTCATCTCACATGCTAGTAAATTAATGCTCAAAATTCTC
TAAGCCAGGCTTCAGCAATACGTGAACTGTGAACTCCCTGATGTTCAAGCTGGTTTCAGA
AAAGGCAGAGGAACCAGAGATCAAATTGCCAACATCTGCTGGATCATGGAAAGAGCAAGA
GAGTTCCAGAAAAACATCTATTTCTGCTTTATTGACTATGACAAAGCCTTTGACTGTGTG
ATCAGAATAAACTGTGGAAAACTCTGAAAGAGATGGGAATACCAGACCACCTGACCTGCC
TCTTGAGAAACCTGTGTGCAGGTCAGGAAGCAACAGTTAGAACTGGACATGGAATAACAG
ACTGGTTCCAAATAGGAAACGGAGTTAGTCAAGGCTGTATATTGTCACCCTGCTTATTTA
GCTTGTATGCAGAGTACATCATGAGAAACGCTGGACTGGAAGGAACACAAGCTGGAATCA
AGATTGCTGGGAGAAATATCAATAACCTCAGATATGCAGATGACACCACCCTTATGGCAG
AAAGTGAAGAAGAACTAAAGAGCCTCTTGATGAAAGTGAAAGAGGAGAGTGAAAAAGTTG
GCTTAAAGCTCAACATTCGGTAAACGAAGATCATGGCATCTGGTCCCATCACTCCATGGT
AAATAGATGGGGAAACAGTAGAAACAGTGTCAGACTTATTTTTTGGGGCTACAAAATCAC
TGCAGATGGTGACTGCAGCCATGAAATTAAAAGATGCTTACTCCTTGGAAGAAAAGTTAT
GACCAACCTAAATAGTATACTCAAAAGCAGAGACATTACTTTAACGACTAAGGTCAGTCT
AGTGAAGGCTATGGTTTTTCCTGTGGTCATGTATGGATGTGAGAGTTGGACTGTGAAGAA
GGCTGAGCGCCAAAGAATTGATGCTTTTGAACTGTGGTGTTAGAAAAGACTCTTGAGAGT
CCCTTGGACTGCAAGAAGATCCAACCCATCCATTCTGAAGGAGATCAGCCCTGGGATTTC
TTTGGAAGGAATGGTGCTAAAGCTGAAACTCCAGTACTTTGGCCATGACATGAGAAGAGT
TGACTCATTGGAAAAGACTCTGATGCTGGGAGGGACTGGGGGCAGGAGGAGAAGGGGATG
ACAAAGGATGAGATGGCTGGATGGCATCACGGACTCCATGAACATGCATCTGAGTGAACT
CTGGGAGATGGTGATGGACAGGGAGGTTTGGCGTGCTGCGATTCACGGAGTCGCAAAGAG
TCAGACACGACTGAGCAACTGAACTAAACTGGCCTGAACTAACTAACTCTACGGTGTGCT
TATGTGAATGAATGACATGCCATGCGTGAAACAAGTGAGAAGCCTTGCTCTGTGGTTCCA
TGGTGATCTCATACAGTTCATGGCAGAAACCTGTAGGGGGTTTATACCGACCTGCCAATG
CCAAGAGGCACCCAATGTCTCCTTTGGGAACCAACCAGAAATGGGCAAAGCGTGTGGACT
GAACTCTCCTTTCTCGGTCAAATTTTCCAGTCTCTTTGACCATTTCATAACCCCTTGGGA
ATTAGAAGTTCTAACCTAATCTATTGGATCATAGACTTTAAAGGGACTTGTGATCTATAC
TGTTACTGTGCACTGTGGCTTAGTTCCCAAACTTGGATTGGTAGTCAAGAAAGCGCCTAA
CCTCACTAGGAATCAGAAATTCAGAAGCTAGATGGAGCTCTAGCCCCAAGAACATCTGTG
AGGTTAAAGGTTGCTCAGATTGGGACTGCAATTTTTTTCCCCCCTTTGGTAATGCTGGCT
CTTAGTCGACCAGAGGAGGTTCTTATACTGGTGTGGTAATGCTTGAAAAAATCATCCCAG
CTTTATGTTCGTATCAGTCTTATTATGGTCAGGAATATACTCAGGGTCGTGCACAGGCAC
TCAGGTGGTGAATGTTTCCCAACCAGTGGTCTTAGCTTGGGAGGCATTCCGGAAGTTTAC
TCTGATTGCACCCCAGGTGGCATCAGAGGCAAGCAAGGTTAAAGGTGAAGAGCTGGATAT
CAGGTAGAGATGCTAGCAGGTCTATCCCTGGTACATCCCCACCCCATCTCGGTGGTAGAA
CCGGAAGAGTCGAGTACAGCACCTGCATCGGTAAGTGACAGACTAAGTCCGACCAGGAAG
GAAAAGCTTTTGGTGTAAAGTCTGTCTACACCCCATCTACAGCTGGGAAGGACGCTTCCA
GTAGAAAAATGGCACTGGTCGCTTTATTTCTCTCTTACAGATGGGAGCTAACAATTCCAT
CCTCACTCTTTTGAACTGTATCCTGAAAAACTGGGATAGATTTGATCCCCAGAGCTTAAA
GAAGACACACCTGGTCTTCCTATGTGATACTCCATGGCCACAGATTCCATTGGAGAATGG
AGAATGGTGGCCAGTTGGAGGGTCTCTTAAGTATAATACTGTTTTACAATTAGACTTGTT
CTGTAAGGAACAAGGGAAAAATGATCTTAAAGATAGATTTCTCACTCAGTCGGTTCCTGA
TATCCGCCGTAAGCTACAAAAATGGGCATATGAACCAAATCAGTCTTTAGATACTCTGTT

ACAACTGGCTCAGACAGTCTATTATGGTAGGGAATATGAGGAAAAGAAAGAAAGGCAAAG
AAACACAAAGGAAAAGGCGGAAGCCTTCGCAATGGCTATGAAAAGCTTCCTTAAACTGCC
GGAGAAAAATGCCCAGAGGGACCCAGGTGAAAAGGTATGAGCTTGCTATTAACTGTGGAA
AGGAGGGGCACCTCAAGCGGGATTGCCCTCAGGCATCTAAGCAGGCCCTGGCTCCATGTC
CGACCTGCAAAGGACCACAATGGAAGAGAGACTGCCCTCAGAGGTGTAGGTCTCCGGGTT
CGGACTCTCAAGACAATCACGACTGCAGGTGCCTGGGGGTCCCCACACAAGCTCCTGTCC
TAATTACACCTGAGGAACTCTGGGTATTAATAATTGGGGGGTTCAATTGGTCGATTTCCT
TTTAGATACTGGGGCAACTTAATCTGTGCTTACTGAAGCCCCTGAGCCACAATCTTCCGA
TCCGCTTCCATAATGGGACTGTCTGGATGAGCCAAAAGGTATTATTTCACTTATTCTTTA
TCTTGCAACTGGGATTCTGTCATGCCAGAATCTCCCTCACCCCTTTTGGGGAAGGATATA
CTGAGCAAGGTCCATGCCTCTCTTTTTATGAATATGGAGCCCTCCCTTTCTCTCCCCTTA
GTTGAACAAAATGTAAATCCTGGAGTATGGGCTGATGGAAAATCTGTGGGTCGAGCACAA
AATGCTATTCCTGTAGTTTCAAGCTCAAAGGCCCACACTTTTTTCCACATAAGAAGCAGT
ATCCTCTGAAACCTGAGGTTAAGGAAGGGTTAAAACCCATCATCGAAAATTTAAAAGAAC
AGGGACTATTAACTCCCTTTAACAGTACTTGCAACACTGCTATTTTGTGCATAAAGAAAT
CAAATGTTAAATGGAGACTAGTTCAAGATTTACGTATAATAAATGAGGCTGTAGTTCCTT
TACACCCCATGGTGCCTAATCCTTATACTCTTGTCTGAAATTCCTGAACGAGCCAAATAT
TTCTCAGTAATTGATTTAAAAGATGCCTTCTATTCAATGCCTTTGGTGGAGGAAAGTCAA
TTTCTATTTGCCTTTGAAGACCCTACACAGCCAGCTTCTCAGTTAACCTGGACAGTTTTG
CCCCAGGGATTTCGTGACAGTCCTCACTTATTTGGACAAAATTTGTCACGGGATCTACAA
AACTTTAATAGCTCTGAAGCGGTGGTGTTACAATATGTAGATGATATTTTGCTCTGTGCT
GAGACAGAGGAAGCTTGTTTGTGAGCCTCAGAAGATTTCTTAAACTTTCTGGCAGGCTGT
GGTTACAAGGCATCAAGAGAAAGGATCAGCTTTGTCAACAATCAGTTAGATATCTGGGCC
TAATCATATCAGAAGGGACTAGGGCCATAGGCCCTGAGAGAATTAAACCTATACTAAATA
ATCCCCTATGTATGACTTTAAGACAATTGAGAAGATTTTTGGGAATCACAAGTTACTGTC
GCATTTGGATTCCAGGTTATGGGGAACTTGCCTGGCCTTTATATAAATTTATAGCTGAAA
CTCAACAGGCCCAAACTGACAAACTGGTTTGGTCTCCAGAAACTCAAAAGGCTTTTAAGG
TTCTTCAAACTGCTCTCCTGCAAGCCCCAGCTCTGAGCTTTCCCACATGGTCAGAATTTA
ATTTGTTTGTCACTGAAAGGAAAGGTATGGCCTTGGGAGTTTTGACCCAACCCCGAGGGC
CTCACCAGCAACCTACTGCTTATCTAGGCAGAAAATTAGATGTAATTTCACGTGGGTGGC
CCCACTGCCTAAGAGTAATTGGGGCAGTGGCTTTATTAGCACCTGAAGCTTCAAAAATAA
TTAATGGATGAAAACTTACTGTACTGACTTCTCATGATGTGAGTGGAATCTTAAATTCTA
AGGTTAATATTTGGATGACAGACAGTAGGCTTCTTAAATATTAGTCATTGTTGTTAGAAG
GACCAGTCACTAAGCTTAAAGTTTGTGGAAATTTAAATCCTGCCACTTTCCTTCCTGAGA
AAGAAAATGAAACACCTGATCACGATTGTTCTCAATTCCTAACTTTAAACTATGCAGCTC
GGAAAGATCTAATGGATACCCCATTAGACAATCCTGACATGGAAATATTTACAGATGGCA
GTTCTTTTGCTTGGGATGGAAAGCGTAAAGCAAGTTTTGCCCTGGTGACTCCTGAACAGG
TTTTGGAAGCAAAATCTCTCCCCCAGGGAACCAGTGCTCAGTTAGGGGAGCTTGTGGCTC
TGACCTGAGCTCTAGAGTTAAGCAAAGGGCAGCGGGTAAATATCTACACTGATTCTAAGT
ATGCTTATTTGACTTTACATGTTCATGCTGCAATATGGAAAGAAAGACAGTTTAAAACAG
CAAAAGGAGAACCTATTAAGCATTTCAGAGAGATCAAGAGACTTTTAACTGCTATATATT
GTCCTAAAGAAGTAGCTGTTATGCATTGCAAAGGGCACAGCAGGGATGGGAGTACAGTAG
CTGGAGGTAATCAGCTGGCTGACTGTCAAGCCAGAAAAGCAGCACTTTACAAAGCCCCTT
CACTGCAGATGCCTTTGATCTGGGCAGGTCCTGTGGAACAGGAAAAACCGTAATATCCTG
AGTAAGAATTAGAAAGATATGAAAAAAGAGGAACAAAGATTACTAATAAAGGATGGTTAC

AGTCTGAGGATGGACGATTAAAAATTCCTGAAAATGCTCAATGGAAAATTCTTAAGGGTT
TACATCAGAGTTGTCATTTGGGTGTGGAGAGTACTTATCATGGCTTCTCGTTTGTTTGAA
GGTAAAAATGTAATGAAAACTTTAAAAAATATTATCAAAATGTGTGAGGTTTGTCAGAAA
AATAACCCAAAGACTGAAAAGCTAACGAAATCTGGATTACAATGAAGTGGAAAGTATCCT
GGAGAAGACTGGGTAATTGATTTTACTCATATACCAAAACCTAATGGATATCCTTGCTTA
CAAGTTTGGGTATATAGTTTTACTGGACGGATTGAGGCTTTTCCCTGTCGTAGTGAACAG
GCTAAGGAGGTTATAAAGATTTTAATCCATGCAATTATCCCCAGGTTTGGGCTGCCACGA
AGCCTTCAGAGTGACAATGGCTCCGCCTTTAAAGCTGCTGTAACTCAGGGTTATCTAAAG
CTCTAGGAATAGAATATCACTTACACTGTTCCTGGAGATCCCAGGAAAGGTTGAAAAAGC
TAATGACATTATCAAAAGACATCTGAGCAAATTACCTCAAGAGAGGCAGGACAATTGAAG
TCCTACCCATAGCTCTAATGAGGGCTCGAACTGCCCTCAGAAAGGAGGAACTGTCCCCCT
TTGAATGTATTTATGGAAGGCCTTTCTTACACACAGACATTGTTATAGACCCTGAAGCCT
TGGAATTAATTAGTTATGTAACTCAGCTCTCAGCTATTCAACAGACATTAACAAAACTCC
AGGAGACGACTCCTGACCCCGCCTCTGAGTCAAGCAAGCCTCTATTTGAGCCAGGAACTG
AGGTCCTCATAAAAACTTTGGGATCTGGGGGCCCATCCCTCGAGCGTCTCTGGGAAGGCC
ATTACCAGATTATTCTTTCTTCTCCCACAGCTGTCAAAGTGCCAGGAATAGATTCGTGGG
TACATCACACTCAAGTTAAGAGGTGGCACCCTGACCAAAACTAAGTGACATCTTTTTATG
TCTTTATTTTCTATGCTCTGACTTTGTACTTTTCAGATGGGCCTGATAACCTATGTGAGC
TTACTTCTGCTGACTCCAAATATCCTGAGTCTGCCATTGGATCCTCAAGACAATGCTTTC
CTGTCCTGGGCTCATTCCTATGCTGCATTCCACAATCGGTCTAACTGCGGGGTCAGTGGA
ACAAACCCCTCTTCATCAGTGGAAGGCTTCCCATGGTGGACATATCCACTTCAAGGAAAA
GATTTTCTCCAAGTGTGTGAATACCTTCAACAACAGTCACAAGCGATGCCTCTCCTTCAT
CTGATGACATCTACCAAGCCTAAAATGGATGGGTGCAACACTTTGTACTTTTAACTATGG
ACATAAAGTGGCTTTTAATTTTATTATACATTGTCTTGGTTCAATGACTTTTGCTACACA
TAAAGCAAATAGGTCTAGATCTAATGGTTTTTTACCTGACGTTTTTCACATATGGGATGA
GGTTATATGGCTAACTCCTGAAAAAGGACTTTTAATATCTACTGCCCCTATATGCTGAGA
ACAAACAGCTGCTGCTGCTGCTAAGTCGCTTCAGTCGTGTCTGACTCTGTGCGACCCCAC
AGACAGCAGCCTACCAGTCTCCGCCACTGGGATTCTCCAGGCAAGAACACTGGAGTGGGT
TGCCATTTCCTTCTCCAATGTATGAAAGTGAAAAGTGAAAGTGAAGTCGCTCAGTCGTGT
GCGACTCTTTGCGACCCCATGGACTGCAGATTCCAGGCTCATCCGTCGATGGGATTTTCC
AGGCAAGAGTACTGGAGTGGGGTGCCATTGCCTTCTCCGGAGGACAAACAGAGCCATCCC
CAAAAGTTAGCCAACAACTTAATTACAATGATTGGAAACAATTGGGATTTTTGCCTCAGA
AAATACGCAACATAATCATTCCAATGTTTTCCAACCCTGGTTCAAGTTCTCCCTTTGTGT
GGCCAGGCACTAATTGGGACTGGATATCTCAGTCACGCTGGCTTGCTCCAAACGGGACTT
AGTGGATATGTGGCTCTTAATATGGGCCCGGCTTCTCCCTGATTGGATAGGGAGATGCAC
TCTGGGTCTAGCCTTTACTCACGGCTTTGTATTTTCAGAGCTTTTAGAAAAGCCTGATAA
TTTACCCCACCGTAAATCTCCATGGGCCGGATTGGTATTTCACTGATATGATTATTTGGC
TGCAGTGTTTCTTCCCTCTTTGAAAACTACAGATGTTATGCTACAAGTGGATGCTTTGAC
TAATTTTACTCCACAGGCATCATAAGATTCTCCAAAGGCTATTTAAGCTCTAATGCTGAA
CAAGCACAAATTAAAAAGGTGGTTTTACAAAACAGATTGGCTTTAGATATTCTGACAGCT
ACGCAAGGAGGAACGTGTGCTATTACTCATACCCAATGCTGTACATATATATCCAATATG
AACACAAATGTTACTTATTTTACTAAACACATGAACAAGATGATTGGGGCCATGGATACT
CCTGAAGCCTCAATTGCCTCACTTTGGGAGACGTTAACCAGTTCCCCATGGTGGACAACT
ATCTTAATTATAATAATTCTGATTGTTTTCTTTTTACTGTTTTCTCCCTGCATCTGTAAC
TGTATAACTAGATTTGTTTCTAGCCACATGAAAGCTTTCAAGTTGCAAATGGTTGCTCAA

ACTTCTGCTACTGCTGCAGCTGCCTCCAACTACTATTTGGGGCCCCTGGATCAGATATCC
TCAATATGAGGATTAAGAAAATATGCTGCCTCACCAATTTAGGGACAACACCCTTCTCAG
CTCAGAAGCAGTTATGGAATGAAAACAACGCCCCTTTTCCCTAGGCAACATAATTCTCCT
AAAAGAAAAGTGGGGAATGAGAGGATAACAGGCAGGAAAGCAGGGGTCTCCAAATGTAGG
AAATAGCCTGCAAGTGTCAGACATTTTTCTCTCTCTTAAGCAGCAGGAGGAAACAAACTA
GTGATATTTTTTTTCCTTCTCTATACAAATTTAAAAGGAGGTTTCTCTTAAAATTCTATA
TTGCCATAATGACACCTGGTTTCACCTGAAGTTAACCAATGCCTTTTTCTTATGGAAATG
TCTATCCTAAGCTATGCTAATGAACTATGCATTTACCCCAAACTCTGTCTTCAATTCGGT
TCTGCCTCTTGGCTCAGAACCTACTTGATAAACCAGTGTGTTATACTCTGATATTGTTCC
TCTAACCTATATAAATGAAACTATTTATATTGTGATCTGCCCTTCTTCAAGATTCAAGTT
AATCATTTTATGGCCCAAGATAAACCATTTGGTGCCAAGATTATCGCAAAATACATCTTA
TGGGTGATGGGCCTGGTGCCATTCTAAGTTTTAAGACATTCCTTTCTTTCATTAACAGGC
TGCTTGTGACTATATAACATCCAGCTGAAAACTAGCAGGGGGTACTCTTTCTCCCCCATC
TGATGCCTATGTCAGAAACTTTCTCTATCTCCTTTATACTTTAATAAAACTTTATTACAC
AAAAGCTCTGAGTGATCAAGCCTCGTCTTTGGCCCCGGATTGAATTCTTCTCCTCCGGGG
GCCAAGAATCCCGGCATCGTAATTCAACAATAACCTTTCA
>CAP_ERV_20
TGAAGGGTTAATGCAGCCACAATAGGGAAAGTGGAGAAGTGCCTGCAGACGGGGCTCTCT
GCTCGGGCTGAACGTACTTGCAAGTGAGGCGTTCTGCCAAGGAGTCTGGACATAGCCTTG
AGTTTGATGGTCCCTTGCAAACGAGGGAACATTCCCTTCTTGTGATAAGAAGAAGGAGGA
GGGCTCTGGACAGACTCTGCAGTAAGCAGAAATTTCAGTCCCTTTTGCTGTATGATAACA
TTTATGCACATGCGCTATACTGAAAAGGCTTGGTCATACAGTCTGGAATTCCGCCCAGGG
GGGCTATATAAAAGTGAACCACAAGCTCGCTTGCTTGCGCAGTTCTTTTTCCTCTGGCCA
GAATGGTGTCTGTCTTTTGTGTGTGTTGTCTTTGTGTCATTTCACTGGCAATCTCCAACA
TCTGGAGCCCAACGTGGGGCTCGAGTGAAACCGAAAGGGTGAGTAACCCCGGGGGGATTT
TAAATCCATAGCAGGGGAACTTTTGGGAAAATCATGGGGAATTCCTCACCCTAGCAGGGG
AACTTTCAGGAAATCATGGGGAATTCCTCATCATTAAGGACACAGTACATGGAGTTAGTC
AAAGGACTTCTCCACTCCATAGGCGTTAAGGTCTTGACTCGTCGATTGAGTGAGCTCTTT
CGCTTGGTGGAGAAATATTGTCATTGGTTTCAATATCAAACTAAGTTACAGTTAAATTTG
AAGGAATGGAAAATAATTCAAAAAGAATTGAGAAAGCAACATCAGAAGGGTAATGTGATT
CCTTTGAAGTTATGGACTTTATGTAATGCTGTAACACAGGCTTTGACTTTGCTATCTACT
GATAATGAGAGTAAATCTAATGCTTCAATGAAGGAAGAGGCAATTTATGAGGATGTGCCA
GACGTTGGTGGGGCTTCTGTGTTGCCTGAAGGCAAGGATACAGGTGAGCCTCCTCCTGTA
AATGGTGAAACATCTGATTGTTCAGAATCAGATTCGGAGGCTTCTTCGGTTTCGTCAGAG
GAGGGTAAAGAGATTAAAGAAATGACCCATCTATTCCAGGAGTGGTGGAAATCCCATAAG
GAGGAGAAGAAATCTGCGCCTTCTGCTCCTCCTTGTGCTTCTCTTTTCCCCACTGCGGTT
GGTCGGCCCGATGTGCGCAGGGAACATTGTCGGTTCTCCTTTCCTTTGTCTATGCTTCAT
GATGATGACTCGCCTGCTCCCCCTGGTGGTTTTATCGATCCTCCACAATTATTTCCCATC
CAGAGACAGCAAGATGACAACGTGATAAATGTTCAATACACTCCTTTGGAATATAAATTT
TTTAAAGATCTTAAAGCTACAGTAGCGCAGTATGGTCCTCAATCTCCCTTTGTTTTGGCT
ATGCTGGAATCATTGGGAAAAGGCAAATTAATTATTCCATTAGATTGGGAATCTATTGCC
CAAGCTGTCTTGGAGGGTTCTCAATGGTTGCAACTTCGTAGCTGGTGGGAAGAAGAAGCT
AGAAAGCAGGCTCGGATTAATGAAGGACAGAATCCCCCTGGTCCTCTTGAGGATAAGTTA
ATGGGAGAGGGCTGTTATCGGGCTTTAAGAGAACAGGCTCAGTACTCTGATCAGGACTTA
CAGCAAGTCCGCCAGGTCTTTTTACGAGCATGGCATCGTGTGGTGCCTACTGGCCACACC

CAGCCCTCCTTTGTTAAAACAATGCAAGGCCCCAGTGAGCCATATACTGATTTTCTAGCA
AGACTGAGGGTAGCTATGGAATGAGCTGTAGGGAGGGATGAAATTTCAGAGATATTATTA
CAAACTTTAGCATTTGAAAATGCAAATACTGAATGCAAACGTATCCTGGGACCATTAAAA
GGACAGGGTGCATCTATAGCTGAATATATCAGAGCCTGCTTGGGAGTAGGAGGAACAGAG
CATCAGGCTAGTGTCTTTGCTACAGCCTTGGCCAAAGCTATGAGACCACAAAAGGGAGGT
AACTGCTTCCATTGTGGAAAACCTGGTCACATGAGAAGAGAATGTTGGAAATTAAAAGCT
GATCAAGGTGCAATTCCTAAAGACAGATCTTTTGCTGGGAGGAATAAGACTCCTCCCGGA
CCTTGCCGTCGATGCGGGAAGGGGCTTCACTGGACCAATGAGTGCAAATCTAAAACAGAC
AAAATGGGCAACCCGATACCGGGAAACTATCCTGCGGGCCTGAGTCCTTGGGGCCCAGGA
ACAATACCGGAGGCTTTTCCTCCTTGCCCTCCTTCCCTCCCAGCAACAGTGACCAGTCAA
TGCCTCATTAAAAGGACCTCAAATGATGATTTCAGACTTACGGTCTGCTACTTCAGGAAG
TGCTGCTGCTGATTTGCCACTAGCTGAGAATGTTCTTTTGTCACCAGGAGGAGGCATTTA
CAAATTAAAAACAAATGTATTCGGACCACTGCCTAAAGGCACCTTTGGCTTAATATTAGG
CCGTAGCAGTGCGGCTTTGAGAGGTCTAACCATAATTCCTGGGGTAATAGACCCCGACTA
TGTTGGGGAAATCTTGATTATGGTTTCTACTTCTACCACGCTTTCATTATTAGCTGGGGA
ACGTATTGCTCAAATACTTCTCCTACCTTATCATCCCTTTTTGGCTCTTCCTAATGAACG
AACAGGAGGATTTGGAAGTACTGGGCGACATATATTTTGGGAAATGCTTATCAAAGATTC
TCGCCCTGTTCTCTCCTTAATTATACAAGGAAAAAACTTTGAGGGACTAGTAGACACAGG
GGCAGATGTTTCAGTTATTTCTTCTCAACAATGGCCCCAAGATTGGAAAAAAGAAAAAAG
CCCTCTAATGCTGACGGGACTGGGCTCTATTGCAGATGTCTGGAAGAGTACCCATCCCTT
GCAATGCCAATTCCATAATGCAAGATCAGTGTCTGTTACCTTTTATATTGTAAACATACC
TATTACTATTTGGGGAAGAGATCTTCTCTCTCCATTAGGGGCTTTTGTAACCATTCCACC
GGAAAACTAGTAGCCACTGCTCAAATTCTTCGAGCACTCCCATTAAAATGGTTAACTAAT
ACTCCAAAATGGGTTGAGCAGTGGCCATTACCACAAATGAAGCTCGAGGCGTTAGAACAA
TTAGTACAAGAACAACTCCAACTTGGTCATATAGAGCCCTCTACCTCCCCCTGGAATTCT
CCTGTTTTTGTTATAAAAAAGAAATCTGGAAAATGGAGAATGTTAACCGATTTACGAGAA
GTTAATAAATGTATTGAACCTATGGGAGCATTACAATTGGGACTCCCCTCTCCAGCTCTT
ATTCCTCAGAATTGGTCCTTAATGGTGCTAGATCTTAAGGACTGTTTTTTTGCCATTCCC
CTACAATTACAAGATAGAGATAAATTTGCTTTTACAATTCCTGTTCTTAATCATGCTCAG
CCTGTTAAGCGTTATCAATGGACAGTCTTACCACAGGGAATGACAAATAGTCCTACTTTA
TGTCAAGAATTCATAGCTTGCTCTTTACAATCCCTCCGTCAAGAATACCCCAATTATATT
CTATATCAGTATATGGATGATCTCCTATTAGCAGCTCCTAGTATTGTCGAACGTGATGAA
TTCTTTCTAAAAGTACAGGAGGCTTTAAGACTATACAATTTGCAAACAGCCCCAGAAAAA
ATTCAAAAGGACTTTCCTATTTCGTATTTAGGGACAATATTGGAACAACATAGAATAATG
CCCCAAAAGTTGCAATTCAGAAGAGACCACCTCAAAACCTTAAATGATTTTCAAGTTATT
GGGAGATATCAATTGGCTACGCCTGGTACTTGGGATTCCTACTTATCAATTACGACATTT
GTTTTCTTCTTTAGCGAGAGATAACAGCTCTGGATAGCCCCCGGACTTTAACCCCATTGG
CTTTACAGGAACTTCAATTTGTTGAGCAACGACTAAATGACGGCTTTTTTATTTACTTAC
ATGCGTCTCAACCTATTTTGTTTATAATATTTCATACCCCTTATTCTCCATCTGGTGTAA
TTGCTCAAGAAAAAGGATTAATAGAATGGATTTTCTTACCTAACAGCTTTTCAAAAAAAA
TGACTACATATATGGATAAATTAGCCTTCCTTATACAGAAAGGTCGCCATCGCATTTTAC
GATTGTCAGGATGTGAACCACACCAGATTGTTACTCGGTTAACAACTGCTCAAATATCTC
GATGTTTACAATTTAATGAAAACTGGCAAATTTCTCTTGCCTCATTTCCTGGTTCGTTTT
CTAATCACTATCCATCATCTAAATTGATTGATTTTCTTCGGGCTAACTCTATGATATCTC
AATCCCCAATTTCAGATGTTCCAGTTAAGGGACCCACTATTTTTACAGATGCAAATAAAA

ATACCACTGGATATTGGACCCTGGAAAGTTCCAAGGTTCTCCCCCATTCATTTTCTTCTG
TACAGCCCGCTGAATTGGGGGCTATCTATTTAGTTTTACAAGATTTTCCCCAACTTCCTA
TTAACATTGTTTCAGATTCTCGATATGCTGTTCTCTCTTGCTTACAGCTTCCCCATGTCT
CCCTTCCACTGACCCTTAAAACAGCTATTGATAAATTGTTTTACCAAGTACAACAATTGC
TCTTGCAGCGTTCAGAGTTAATTTTCTTTACTCACATTCGTGCACATTCCGCCCTTCCTG
GACCCTTATCATTCGGAAATGCTACAATTGATGCCTTAATTTATCCTATAGAAGCAGCAA
AACAAGAACATCTCTTACAGCATACCAACTCCAAAGGGTTACAAAAATCTCATACTATTA
CTCGAAAACAAGCTCAAAATATTGTTCGCTCTTGTTCCATATGTGCACCTTTTACTTTGC
CATTTACCCCACCAGGTGTCAACGTAAGAGGACAACAAGCAAATCAGATATGGCAAATGG
ATGTAATTTACGTACATCATACTATAGACACATGCACACGTTTTCAATGGACCACTGCAT
TACATTCTGAAAAGGCTGATGCTGTTATTACTCATTTGTTATCTTGTTTTGCAGTTATGG
GATTACCAATTGAATTGAAAACTGATAATGCACCTGCCTACCAATCCGCGAAATTAGCTC
ACTTTTTATCCCAATATCATATAACTCATACTTTTGGTATTCCTTATAATAGTTAAGAGC
AAGCTATCATTGAAAGAGCTAATCGTACCTTGCGTGAATATCTTGAAAAAATAAAAAAAG
GGGGAACAGGAGAGATATATGAAACCTAAAGACATTTTGAATAAAACCTTACTTACCCTA
AATTTTTTGAATATTTGGAGCAAGGGAAATCTATCAGCAGCAGAGTTGCATTTTCAAGGG
AAAGAAGAGGATAAGAAGATCTTGAATATGCCTATTTGGTATAAAGATAAAGAGAAAGGT
TGGATCCCAGCATCATTAATATATCTGGGACGAGGGTATGCTTTCATTTCTGTTAATAAT
TACAGGTTTTGGACCCCAGCGAGATTGATCAAAATCGACAATGGCTGATCCCTTTGTTCA
AAAATTCAAAGAGCTTACTATGCAGAGAAGCTTTACTTTTGGTACAAGGGAAGCAACACC
TCCTACATGGGGTCAAATGAAGAAGTTGACCCAGGAAGCAGAAAAGACGTTAATGAAAGC
GGGACAGCCTCTGAATCCTACCAATCTTTTGCTTGCCATGATGGTGGTGGTAACATGTCA
GGTAATCGGTGTCTCGGCAAGTAATCATACGTATTGGGCATATATACCTAATCCCCCATT
AGTAAGAGTAGTTTCCTGAGGGGAACCAGAAGTGCAGGTATGCACTAATGAGACTGCCTT
CTTTCCCCCACCAGCTTGCGGGGGAACAGAACAACTATCTCATCATAAACAACAGTATAA
TATTAGTAGTTTGACCATTGCAGCGGAAAGTATTCCTTTGTGCATAGGAGGACACACTTT
TGTCTGTCCGCTAAGGAACATTCTCATCATTCCTATAATACATGGGGGGTAAAATATAAT
AATTATCATTTTGCTACTTTTACTATGCTTGTTTCCACCAGGGGCTTTTAACACCTCGAT
AGAACCGCTAGACATTCATAATGGAATATATATGTCACTATGTCCTGTTAACTGTTTTGC
TCCTTCTCTAGAATCTTTGGAATGGGAATGTTGCCAAGGTCATCAACCCTTTAAAGTCAT
GAATTATTCTGGGTCTGTCATTGTAGATTGGAGTCCAGATCATGGGCAATTCTTAGAAAA
ATGGTCAAATAAGTCTCTTAGGTGGCATCGTGCAAATAGCACTTTGATGGGCAATGGTAA
TGAAACAGTTAAATGGCAGCAGTTTGCACTTGTCCCTCCTCAATTACAATTGCAGGGATA
TCCACACATTCAAGGGGATATTTGGAAACTATGGGCAGTTTCTGGTAATCTCACTGTCTG
GTCAGGAAACTATACTTTGGACAGTGGTGACTCTTCAGGTCCATTTCATGTTAATTTACA
CGTTAATAAATCTTATTCCGCAATGGCATGTGTAAAATATCCTTTTGCATTGTTATATGG
AAATTGGACCTGGAATGATACTGTGGGGTCTGTGTCATGTGACTACTGTAATCTAACTCA
ATGTGTAAATCAGTCTTGGTGGAAAGAATTTGAAAGACGAGTGTGTAACTCCAATTTTTC
GTTAGTAATTGTTAAAGCTTGGACAGAAGTATGGTTGCCTATAAATCTGACTAGACCATG
GTCAGATTCTTTTGCTGTTTCTCATCTAGTAACCGCTGTACAGACTTTGTTACATCGATC
TCAACGTGTGCTTGGTGTGGTCATTACTTCGATTCTAACAGTCGCGTCAGTAACTGCAAC
AATGGCGGTAGCAGGCCTCGCGTTACACCAAGGAATTCAAACAGCTGATTTTGTTCAGGA
CTGGCATAAAGACTCTCATTTGTTATGGCAACAACAGCGAGATTTGGATGCACAACTTGC
TACTGACGTGCTCAATCTTCAACACACCGTTTCCTGGCTTGGAGATCAATTAGCTGTTTT
ATCTACACAAAGTGTGTTGAAATGTGATTGGAATTCTTCTCAGTTTTGTATAACACCTGT

ACCATTTAACATGAGTGAGGGATGGAAAGAGTAAAACGATCTTTGACTGGACATCAAAA
TCTCACTACAGAAATTATGGACCTGGAACGACAAATTTTGTCTACTTTTAGCGGGACTTT
ACCTGACATTGTGGGGTCTGGTTTGTTGAAAAGTCTTCAAGAAGGATTGAATAACTTAAA
TCCATTAGGGCATGTATCCACACTAATTGGGACTACCTTTGGGAACACTATGTTTATATT
ACTTTTATGTTGTGTTGCTTTTCTAGTCTTCCAACGATGGCGGAAAAGGAAACAACTAAA
GCGTGAAGCAGAGAAGATCCAGACCATGCCACAATTTATTAAAGCAAATAAAAAAGGGGG
AGATGAAGGGTTAATGCAGCCACAATAGGGAAAGTGGAGATGTGCCTGCAAACGGGGCTC
TCTGCTCGGGCTGAATGTACTTGCAAGTGAGGCGTTCTGCCAAGGAGTCTGGACATAGCC
TTGAGTTTGATGGTCCCTTGCAAACGAGGGAACATTCCCTCTTGTGATAAGAAAAAGGAG
GAGGGCTCTGGACAGACTCTGCAGTAAGCAGAAATTTCAGTCCCTTTTGCTGTATGATAA
CATTTATGCACATGCGCTATACTGAAAAGGCTTGGTCATACAGTCTGGAATTCCGCCCAG
GGTGG
>CAP_ERV_21
TGTAGGGAACTAGATTTAAGGAAGGTTGAGAAGTGCTTGCAAACGAGACTCTCAACCAGG
GCTGGACATTCTTGCAAACGAGATGTTCTGCCCAGTGTAGGGAACTAGATTTAAGGAAGG
TTGAGAAGTGCTTGCAAACGAGACTCTCAACCAGGGCTGGACATTCTTGCAAACGAGATG
TTCTGCCCAGTGTAGGGAACTAGGTTTAAGGAAGATTGAGAAGTGCTTGCTAACGAGATG
CTCAGCTAAAAATCCTGTTTGTTCCCGTGGGAAAAGAACATTGCATTGCACACAAGGATG
TTTCTCTGATTCCTCAATAGGAACAGACCTTGGGACAAAACGGATTCTAAGTTGATAAGG
AAGTTCCCCAAAACAAAGTCTTAGCTGCAGTAAATAAGCCAAGGTAAAGGTTATCTCGCC
CTGCGCCTGCGCACTGTATAGTCTCTAAATCATAGTGCTTGGCAGCTTGCCCTGTAGGGG
GTTGTACAAAGGATATAAAAATTAAGCAGCTGTAAGAAGCAGGTGTTAAAATATAAAGAT
TAAAACCACTCTGCAGTGTTGGTGTGTCATTCCGTCGCCGACATCTGGCGCCCAACGTGG
GGCTCGACGGAACCGAAAGGGTAAGCACCCCGGGGCAAAAGGCTAAAAGGGGGACTTTCG
GGAAATAGAGAAAAAGAGAAAAATAAAAATCCGAAAGGGTAAACACCCCGGAGCAAAAGG
CTGATAGGGGGACTTTCGAGAAAAAGAGAAAAATAAAAATCCGAAAGGGTAAACACCCCG
GAGCAAAAGGCTGATAGGGGGACTTTCGAGAAAAAGAGAAAAATAAAAATCCGAAAGGGT
AAACACCCCGGAGCAAAAGGCTGATAGGGGGACTTTCGAGAAAAAGAGAAAAATAAAAAT
CCGAAAGGGAGAGCACCCCGGAGCAAAACGCTGAAAGGGGGACTTTCGAGAAAATAAGAA
AAATAAGAGAAAAAGAACAGAAAAAAGAAAAAAGAAAAAGAAAAAACTATGGGGAATTCC
CCGTCTTTAAAGTCACAATATATGGAGCTAGTTAAGGGGCTCCTGCATTCTATAGGAATT
AAAACGTCTACTCGCCGCTTGAGTGAGTTGTTCTGTCTTATAGAGCAGCATTGTTATTGG
TTTCAATATCAAACAGAAGTACAGCTGAATTTGAAGGAATGGAAAGTGGTGCAGAAAGAG
TTAAGAAGGCAGCATCAAAAAGGAAATGTGATTCCTTTAAGGCTGTGGACTCTATGTAGC
GCTATAACACAGGCTTTAAAATTAATGGCTGTTGATAGTGAGGCCGTCTCATGTTCTCTT
AAAAAGGAAGATCCCCTATATGAAGATGTGCCTATGAGTGAGGAGAAGGAAGATAAAGGT
GAAAGGGGGGCTTCATCTCGGCCTCCGCTCAAACCCCCAGATGATAGTAAAGATTCATCC
ACTGAGTCAGATAGAGATGTAAGTTCTGGGGAGGATTCAGATTCTGTAGAGGCAATGACT
ACTGCCTTTCGGAAGGTTTTATCAAATAAAAAGCGAACTTCCAAGGCTGTTAAATCCTCT
GCACCACTCTACGCATCCTTATTTCCAGTAGCTGCCGACAAGGCTGAGGTAGGGAGAGAG
AATCAGCGATTTACATTTCCTGCTACTAAGTTCTATGATGATGATGATCTTTCAGCACCT
CCTGGAGGTTTTGTGGATCCACCTCGCCTCTTTCCCATTATACGTCAGCATGATGCACGG
GCAGGAATGATTAATGTTCAATATATGCCTTTGGAGTATAAATTTTTTAAAGATCTTAAG
GCCGCTGTTTCTCAATATGGTCCCCAATCTCCTTTTGTATTATCAATGTTGGAGAATGTA
AAAACTTCTAAATTAATTCTTCCTCTTGATTGGGAATCAATAGCTCAAGCTGTTTTAGAA

GTATCTCAATGGTTACAGCTTCGTAGTTGGTGGGAAGAAGAAGCCCGAAAACAAGCTAGG
ATTAATGAGGGGCAAAATCCACAAGGTCCTTTAGAAGATAAGTTGATGGGAGAAGGGCAA
TATCGAGCCTTGAGGGAACAAGCTCAGTACACAGATCAGGAATTGCAACAGGTGCGTCAG
GTTTTCTTAAGGGCGTGGCGTAGAGTGGTACCTACTGGACAGGCTCAACCGTCTTTTGTG
AAAACTATACAAGGTCCTAATGAGCCTTATACAGATTTTTTGGCCCGCCTTAGGGTGGCT
ATTGAACGGACAGTTGGTAGAGATGAGATCTCAAAAATTTTGCTTGATACATTGGCATAT
GAAAATGCTAATCCAGAATGTAAAAGGATTTTGGGACCTTTAAAAGGACAAGGGGCTTCT
GTGGCTGAGTTCATAAGAGCATGCTCTGGAGTTGGAGGAATTGAACATCAAGCCTCTGTT
TTTGCAGCAGCTTTAGCAAAGGCTGTGAAACCTCAGAGGGGAGGAAATTGTTTTAATTGC
GGGAAGCCAGGACATTTTCAGAAAGATTGTAAGAGACGAAGAAATGAGCCAAAAAATGAG
CGATTGCCAGAAAAGCGACAGCCCTTAGGTCTTTGTAGGAGATGTGGCAAGGGAAAGCAT
TGGGCTCAGTAGTGTAAATCTAAAACTGATCGGGAAGGGAATCCCTTAAGAGCCCCTTTG
TCGGGAAACTTCCCAGTGGGCCTAAGCTCCTGGGGCCCAGGAACAGTACCGGGAGCTCCT
TGCCAGTTTCCTGCACAGGTCAATGCCAATCTCGACAGCCAGTTTCCTCGAATGGTGCTC
AAATGATGATTTCAGATTTGAAGGCTGCTACATTAGGTAGTGCAGCTGCAGATCTTCCAT
TGGCTGAAAATGTTATTTTATCCCCAGGAGGAGGTATTTATAAATTACGGACTAATGTTT
TTGGCCCATTACCAAGAGGAACTTTTGGGTTAATTTTGGGACGAAGTAGTGCAACATTAA
GAGGATTGATTGTATACCCAGGAGTAATTGATTCTGATTATGTTGGGGAAATTTTAATTA
TGGTTTCTACATCACAGACTCTTTCTTTACTAGCAGGGGAACGGATTGCACGACTTCTCT
TGTTACCTTATCACCCGTTTTCTTCATATTCTAATGAGAGAGTCGGTGGTTTTGGAAGTA
CGGAAAAAAGTATATTTTGGGAAATGCTGATTAATGACTCTCATCCTTTAATGTCACTGA
TTATTGAAGGTATACAGTTTGAAGGATTAGTGGATACAGGAGCAGATGTTTCGGTTATTT
CTTTCCAGCAGTGGCCTAATGACTGGAAAAAAGAAAAAATTCCTCTTGTTTTAACAGGCT
TAGGATCAATAGCAGATGTGTGGAGAAGCACTCAACCTTTGTCATGCCAATTGTCTAATG
GAAAGAAAGTATCTATTTCTTTTTACATTGTTAATATACCTATTAATATCTGGGGAAGAG
ATCTTTTATTTTCTTTAGGAACAACACTCACCATCTCGTCGGAAAACTTGTAGCCACTGC
TCAGATTCCTCGAGCTCTCCCATTGAAATGGTTAACTAATGTCCCTAAATGGGTTGAGCA
GTGGCCGCTTCCAAAAGTGAAGCTCGAGGCTTTAGAACAATTAGTAAAAGAACAGCTTCA
GTCTGGCCATATTGAACCTTCTACGTCTCCTTGGAATTCTCCTGTTTTTGTCATTAAGAA
AAAATCTGGTAAATGGAGAATGTTAACTGATTTAAGGGCAGTTAACAAATGTATAGAACC
TATGGGAGCTTTGCAATTAGGTCTCCCTTCTCCTGCTTTGATTCCACAGGATTGGTCTTT
GATGGTTTTAGATTTGAAGGATTGTTTTTTTAATATTCCTTTGCAAATAAAAGATAGAAA
TAAATTTGCTTTTACTATTCCAGTGTATAATCATGGGCAGCCCGTAAAACGTTATCAATG
GACAGTGTTGCCTCAAGGAATGATTAATAGCCCTACACTTTGTCAGGAGTTTGTTAATCG
TGCTCTTATTACTGTGAGACAACAATTTTCTAATTGTCTTCTCTATCATTATATGGATGA
TCTTCTATTGGCAGCTCCTAGTAAAGAGGAACGAGATACATTTTTTATTCATGTAAAGAA
AGCTTTAAGTGATTTTAATCTTCAAATTGCTCCTGAAAAAATTCAAACTGAATTTCCTAT
TTCATATTTAGGTGCTATTTTGGAACGACAAAGAATAAAACCTCAAAAGGTCCAAATTAG
ACGAGATAATTTGAAAACTCTTAATGATTTTCAGAAGCTTTTGGGTGATATTAACTGGTT
ACGTCCCATGTTAGGGATTCCTACACATCAATTGCGTCATTTATTTTCTACTTTAGAGGG
TGATACTGCTCTTAACAGTCCTCGATCTCTTACTTCTCAAGCAAAAGAGGAATTACACTT
TGTAGAGCAACGGTTGAATGAAGGGTTTCTTATCTACAACAGGATCAACCTATATATTTC
ATAGTGTTCCATACTCCCTATTCTCCTACTGAAGTTATTGCTCAATCTGCAGGGTTGATT
GAGTGGGTATTTTTACCCAATAACTATACAAAAAAGCTTACTACTTATACGGATAAAATT
GCTTTCCTTATTTTGAAAGGTCGAGGCCGTATTACTCAGTTAATTGGAAGTGACCCTCAA

ACAATTATTACTCAACTTACTTCCACCCAAATTTCTAATTGCTTACAGTTTAATGAAAAT
TGGCAGATAGCTCTTGCATCTTATTCAGGGACTTTTTCTAATCAATATCCTCAATCTAAA
ATGATAGATTTCCTTCGACATACATCAATGGTTTGTAAATCTCCTATATCTAATATTCCA
GTTGAAGGAAAAACTATTTTTACAGATGCCAATAAAAATACTGCTGGCTATTGGACTGAT
ACTACTTCTAAAGTTGTTCCACATTCCTTTTCTTCTGTGCAGCCTGCTGAACTTTGGGCC
ATTTGTTTGGCATTACAGGATTTTTTTGATATTCCTATTAATATTGTTTCTGATTCTAAA
TATGCAGTCTTTTCATGTATTTATCTTCCTGAAGCCACTTTACCGGTAACTGTAAAAACT
AATATTGATAAATTATTTTTTCAGGTTCAACAATTATTAATTAGGCGAACTAACCCTGTC
TTTTTTACTCATATACGAGCTCATTCTTCTCTCCCCGGACCATTATCTCAAGGAAATGCA
AATATTGATGCCTTACTTTATCCTTTACAGTCTGCAACACAAGAACATTGTTTACATCAT
ACTAATTCTAAAGGGTTACAAAAAACTTATTCGTTAACACGAAAACAAGCTCAACGCATT
GTTCGTTCTTGTTCCATATGTGCACCGTTTATTTTGCCCTTTGCCCCTCCTGGAGTAAAT
CCCAGAGGACTACAATCTAACCAAATTTGGCAAATGGATGTTGTTTTTATTTCTTCATTC
GGTAAACAGAAATATGTTCATCATACTATAGATACTTATTCTCATTTTCAATGGGCTACA
GCTTTAAGTTCAGAAAAGGCAGATTCTGTTATTACTCATTTATTATTTTGTTTTGCTATT
ATGGGCATACCTATAGAATTAAAAACTGATAATGCTCCCGCATATCAATCCTCTAAATTA
TCTCAATTTTTAGAACAATATCATATTAAGCACACTTTTGGTATTCCTTATAATAGTCAA
GGACAAGCTATAATTGAAAGAGCTAATCGGACTCTTCAAAAATACATTAAAAAATAAGAA
AGGGGGAAAAATGTATGATATCACTAAGGGGCCATTTCAAGTTCATTTGCATATTAATAA
ATCTTACTCTGCTATAGTATGTGTAAAATTTCCTTATTCTTTACTGTATGGGCAATGGAT
TTGGAATGAAATTTTGGGAGTTATATCATGTTCTGATTGTAACTTGACTCAATGCATAAA
TCGTTCTTGGTGGGAAAATGTTGAAAACAAGATGGTCCATTCCAATAATTATTCTTTGGT
CATTGTGAAAGCACGGACTGAACTTTGGTTGCCTGTTAATCTTACTCGACCTTGGTCGGA
CTCTTTTGCTGTCACTCATCTTGTGAATGCTGTACAAACTCTTCTCCATCGATCTCGAAG
AATGCTTGGCATTGTCATCGCATCAATTCTCGCCGTGGCTTCAGTAACAGCAACAGCAAC
TGTAGCAGGTCTTGCTTTGCATCAAGGCATACAGACTGCTGATTTTGTCAGGGAATGGCA
TAAAGATGCTCATTTATTGTGGCAACAACAGCATGATTTGGACGCTCAATTGGCTACTGA
TGTATTGAATTTACAACACACAGTTTCCTGGTTAGGAGATCAAGTGACTGTGTTAGCTAC
AAAGAGTGTGTTAAAATGTGATTGGAACTCATCTCACCTATGTGTAACCCCTGTTCCCTT
TAATATGAGTGAGGGTTGGGAAAAAGTGAAAAGGTCTTTAGTGGGACACCAAAATTTAAC
TGCTGAAATTATAGAATTGGAACAAACTATATTGTCAACTTTTAGTAAGACGCTGCCTGA
TATTCTTGGTTCTGATATACTGAAAAGTCTTCAAGAAGGATTAGACAACCTTAATCCTTT
AGGGCATGTATCTACATTGTTAACAACATCCTTTGTGAATACTTTGTTAATTGTTGCTTT
ATGTTTTATTGCTTTTATAGTCTACCGGCGCTGGCGAAAAGGGAAACAATTAAAAGAAAA
AGCTTTGCATATTCAAGCGCTTATTCAGCATCTACAAGAAAAGAAAGGGGGAGATGTAGG
GAACTAGATTTAAGGAAGGTTGAGAAGTGCTTGCAAACGAGACTCTCAACCAGGGCTGGA
CATTCTTGCAAACGAGATGTTCTGCCCAGTGTAGGGAACTAGATTTAAGGAAGGTTGAGA
AGTGCTTGCAAACGAGACTCTCAACCAGGGCTGGACATTCTTGCAAACGAGATGTTCTGC
CCAGTGTAGGGAACTAGGTTTAAGGAAGATTGAGAAGTGCTTGCTAACGAGATGCTCAGC
TAAAAATCCTGTTTGTTCCCGTGGGAAAAGAACATTGCATTGCACACAAGGATGTTTCTC
TGATTCCTCAATAGGAACAGACCTTGGGACAAAACGGATTCTAAGTTGATAAGGAAGTTC
CCCAAAACAAAGTCTTAGCTGCAGTAAATAAGCCAAGGTAAAGGTTATCTCGCCCTGCGC
CTGCGCACTGTATAGTCTCTAAATCATAGTGCTTGGCAGCTTGCCCTGTAGGGGGTTGTA
CAAAGGATATAAAAATTAAGCAGCTGTAAGAAGCAGGTGTTAAAATATAAAGATTAAAAC
CACTCTGCAGTGTTGGTGTGTCATTCCGTCGCCGACA

>CAP_ERV_22
GGAACTTCTTGAGTTGCAGCAAGGGTGTGAAAGACCCTTTCGAAGTTCAAGAGGGAAGGT
GTGATTAGCCTCGAGACGTCAGTGGAAAAGGGCCTCATCTCGCCTGGAGGAGAGAACCTC
CTTGATTTTCTCGAGTTGCGATAGGTTCCTCTCGAGTTACGACAGGGACCTCATGGACCC
ACTCGTGTGGCCTCAGGAAAGGCCAGTCTCCATATGAGTTGCCAGGGGACCCTCGGGATT
CCTCTCCAGTCTGTGTCGGGACCTAAGTCTTCGTCTGGAGCCGAGGCCAGAACCTGAAGT
TTCCTCTCCAGTGCTGATATTGATCTTTGGGTTCTTCTGGCGTCTCCACAGGGGAGTCAG
GCCTCGTCTTGACTGGAGACATCCACGTCCGCTTTCCTCCCTAGCTGTAGCAGCAGTGTT
CAAATTTCCCCTGCAGTTTACACAGGGATTTGTGTCTTTCCCTCTAGGCTTTACCCCAGG
GTTGTCAAAAGTGGCACCGTGGTGTGTGTCGATTTTCGGGATGACAGTCAAGGTAGTACT
GGGAAATCAGTTCCTCTGGAGTGGACTGAGACATTTTGGGGTCTTTTGGAAGGGTGGCAC
GATCCTTGAAGTTCCTCTCGACTTTCCTGTTGAGAGCGCCTCCTCTTGAGATGCGACGGG
AATGCCGGAATCCTTTCCCGAAGAAGCAAGGAAAGGTACCCGCATCTCGAGCGGAGGAGG
GGAATCGGGGCTCCTCTTGTGTTGTGGTGGGACCCTCGGTGTTCCTCTCAAGTGAGACGG
GTATGTCGGGGAACTTTTTGAGTTGCAGCAACAGTGTGAAGGACCCTTTTGAGGTTCAAG
GTGGAAGGTGTGATTTCCCTCGAGACTTGCAGCAAGAAAGGGCGTCATCTCACCTGGAGG
GGAGAACTTCCTAGTTTTTCTTGTGTTCCTGCAGTTTCCTCTTGAGTTACGACGGGGACC
TCAAGGACCTGCTCCTGTAGCCTCAGGAAAGGCCACTCTCCATGTGAGTTGCTAGGACCT
CTCGGGATTCTGCTCCAGTCGTTTCTGCGTCCTCAGTCCTCGTCTGGAGCTGAGGCCAGA
ACCTGAGGTTTCCTCTCTAGTGCTGACATGTTTCTTGGGATTCCTCTGGAGTCTGCACAA
GGAATCAGGCCTCATCTCTAGTGAAGTTATGCATGTCCAGTTTCCTCCCGATCTGGAGCA
GCAGCTTCAGGCTTCCTGTCAAGTTGACACAGTGATCTGTGGCTTTCCCTCGAGGCTTTC
TCACAGGGCTGTCACACGTGCCACCATGGTGTGAGTTGATAGTCGGTGTGACAGTCGAGA
CAGTGCAGGGGAATCAGGTTCCTCTTGTGGAGGAGCTGGAAGGCTTTAAGCCAATACTGA
AAATGTATTTTCTCCATTTATGATGAAGAGTCAATACGGCCAGCCTTAAGGCATTTGAAG
GCTTCCTTCTGACCACCTGTTTCTGAGAACAAGGACTATTGTTTATGATAAGACTCCCTT
TAATGTTTTGCCAAAGCCATGTTATGTCTTGGGCGATGAGAACTGTATTTTATGCTTGAA
TGCTTTAATGTTTATCTGGAATGGCTATGTACATGTCTGCCTTATGCTCTATTCCCTGAG
CATTATAGAACTGTGCAGTTAGATAATAAACTTTGTCAGACCACTAGAGGCTGTCCCTGA
GTGTCCTTTTTAGAGTGTGGTTCTCTGAGCCTTACAACTGGCGCCCAACGTGGGGCTCAA
AGCAGCAGACTGACTTTGAAAAAGGGCTACACTCCTGCAGAAGCGAGGTAAACATGATGA
GACATCAGTCAAGTAAAATTCCTTTTGCTCACCTCATGCATCATTTCTTGAAACGATACA
GAGTTAATCTGCTTAAAGAGCAGTTAACTAGCTGTTACCAGACAGTGGTTAAATATAATC
CATGTTTTCCGGAGGAAGGAACCTTGGATTTATAAACTTAGACAAAATAAAAGATAATAT
TTTAAAAGCTTATAGACAGGGGGTAAAAATTCCCCCACAATGGTGGGTAACTTGGTCTTT
TTTATGGGCTGTTATAAAACAATTAGATGGCTCAAGAAGTAATTTAGAGGTTAAAACTGT
TGGATCCCTACATAAATACAAAATTTAAAAAAAAGATCTATCAGAGGTTTTAAATCAAAA
ATATCATGCTAGAGCAGATTACAGACAAACAAGAGTCACATATGCTAAAAGCAGTCAAAC
AATCAACTTTGCCAGAGGCCCTGATCTGCCTTTACCTTGCTTTTAGCGAGCTCATATTAA
CAAGCCTCTAACACTGGCCTTTCTGCTCACTGCAACGCTGTCTGATGCTGCTCTTGCTCC
CCTATTTCCTAGTATTCCTCCTCAGGACATGCCTCTTTGTTCGTGGCCTTTTCCGGCCCA
ACTTAACAATCCTCAACCTAGACATAATCAATGGCAATCACCTGATTTTGGCTTGCTCAT
GAAATTCAAAAAGGCATATACGTAAAAATAAAAATTAGAAAAAAACAGTATTGTAAAGTA
AAATAAAGCAAAAATAAAAATTAAAAAAGTACTGTAAAGTAAAATAAAGTAAAAATAAAA
ATAAAAATAAACAGTATTGTAAAGTAAAATAAAATTAAAATTAAAATTAGAAAAAAAAAA

AAAGGCATATACGTTATATAGACCTACCTCACCCTACTGTATGAAATTTCTTAGGGGCTG
GGCAGATCAATGGCTTCATGCAGATTTTTTTACAGTTGCTAAAATGGTTATGACTCCACA
ATAGCTACTACAAAGGCAAATGTGGGTCACAGATAAAGCCAAATTAATCTTGCAAGAACA
ACAGAGCAGGAAAAACCCTACTGGACTATATTTTAAAATTCTCACTGGCACCAAAGCTAT
GGCTAAAACTGCTGTGCAATTACAATTTGTGCAGCCGCCCATGTTATAATAGATTTAAAA
AGTAGCTATCAGAACATGGGCTAAAATCGACAGTTCCACCTATAATAGATCTTTTTTAAA
AATATTGCAGGGACCAACTAAAAAATATACTCAATTTATTGATAAATTAAAAGAGGCCAT
TGATAACAGTCTTAAAGACACATCTTTGCAAAAAATCATTTTAAAACAATTAGCCTTTAA
TAATGCTAATAAAGATTGTCAGGCTATTATCAGACCCATTAGGGGGGAAAAAAGTTATAA
AATACTTAAAAGCCTGCAAAAATGTGGAGATGATTCAACATAAAGCTAAAATAGCCACTT
TAAAAACCTTATAAATTTCCCCAAAGTCTAAAGTTAAATGTTTTAACTACAGCAAGCCAA
GACACATGCAGGAGCAGTGTCACTTGCCTCGACAAACAGGTCTTTCTCCTGACAAAGGAG
GGGCTATTAAAACTAAGCCCCTCAGGCTCTGCCCAAGATATAAAAAGAAAAACCACTGGC
TGACTAAATGCCGCTCTAGATTTGATAAACTAGGTAATGTTTTACCCATTCAGACACTTC
CTTCAAAACACTAAAACAGGGGCTCTCCTCTAGCCCCATTAAACAAGGAAGACAATCAAA
TATTACTAAATTAACAGCGACTACCAGACATAACACATTTATAGACATTCCTACTCCCCG
AGATATAAAATTATTAATGATAAATAATTCTATAAAAATCTTAGCGAAATATTTTGGCCC
TATACCAAAAAATACTATAGGCCTCCTATTAAAACAAAACACCATGCACGAGATAATTGT
ACATACTAAGATCATTAATAAAGATTACACGAATAAAATTACAATAATGTTGCATGTGAC
TCACAACTTGTATTTACAAAAGGGTAACAGATTTGCTCAGCTATTGCTATTACCTTATGT
GCCCCACTTAAAAAAAAAAAAAACAGACACCAAAACAGGTGGCTTTGAGAGTACCAACATT
ACTACAGCCCTTTCTACTGTTATAAAAAAATCAATAAACCCATGTTAAAATTAAAAATTA
AAACAAAAACTTTCAAAAAAATGTTAGACACTGGGGCAAATGTTTCCATCATAAAAACAA
AAAAATGGCCTTTGGACTGGCCTACAATTTTAATCTCACACCAGTTGGTAAAAATAAAAA
CTACAGATGCAGCTCAAACTTATGTTAGTTCATCTTACTTACAAGTCCTGGGCAGTAATC
AATTAGTCACTTACATTAAACCGTATATCGCCCATTACCATTAAATTTGTGGGAAAGAGA
CTTTCTACAACAAGTTCAAGCAACTATACAATTAAATAAACTTTTTCTTAGGAGTCACTG
AGATAAAGCAACTAAAGTTAAAATAAAAGTCTGATAAACCTATCTAGACAGCTCAGTGGC
CCCTATCAAAAAAAAATAATAAAGTCCGCTTTACATACTGTGGTGGCTAAACTACTATAA
CAAAATAAAATGAAAACTACTCAGGCACCATAAAATTCACCAGTTTTTGTCATTAAAAAA
ATCAAATAAATAAAAAAATTCTAAAAGATTTAAAAATATAAACACTATAATGATTCCTATG
GGAACATTACAACCAAGACTCCCAAGCCCTGTTATAGTCCCTAAAGACTGGGCTATTGTG
ATTATTGATTTACAAGATTGCTTTTTCACTATACCTTTACATGCAAATGACAGATAACGC
TTTGCTTTCTCAATACCTTATATTAATAATCAATCCCCTGCTCAACGATATCAATAAAAG
GTCCTGCCCCAAGATATGATAAACTTCCCTAGAGTCTATCAATTTGTTGTTGATAAAATT
TTACATCCTATCAGACAACAATTCCCTGAAGCATATCTCATTCATTACATATATGACATT
TTATTGGCTTCTCCCTCAAAATCTCAATTAAGTTTAATAGGTAATGAGGTCATAACTAAT
TTAACTAATCATGAGCTACTAATAACAAAAGATAAATTACAACACCTTTCCCCTTTTAAA
TATCTTAAATATCTTATGGACCGCTCTACTGTAAAGCCACAAAAACTTTCTATTAAAAGA
GATAATTTACAAACACTTAATGATTTCCAAAAACTTCTTGGAGATATTAATTGGCTACAA
CCTACCTTAAAAATTCCCACATATGCTTTACAAAACTTATTCAAATTATTAAAAGATTCC
TCTGATTTAAATAGTCCCCGACAACTTACCCCGATGCTAAAAAAATTACAATTGTTAAAA
CAGAAAATTCAACGAACATTTGTTTACCATATTAATTACAATTCCCCTTTTCAGATATAT
GTCTTTGATACTAAGATATCTCCTACTGCCATTATAAAGCAAGATAATCACCCTATTAAA
TATGTATATCTCCATTCCAAATAGACTAAACACATTGTTTCCTATATAGACTTAATTAAA

AAAAAATCATTTTTCTTACACGCTCTTACTTGTACACTATAGCTAGATATGACCCTACTC
AAATTTACCTACCTTTGACAAAAACAAAAATTGATAATCTCTCCAGGTGTCCACTACCAT
TAAGATAGCCCTTGCTGATTATTCAAGAGAGTTATTGGCCAACCCACCTAAAAAAAATAT
GAAATTTCTTACAAAATACTTCTTTTATCATCAACAATATTATTTCTAAACACCGTCTCA
TAAATGCACCTAATTATTTTATAAATAAAAATAAGGCAAGATGGACAACGATAATAGGTC
CCAACCTACAAAAAAAATTAAAAATCCTTATCAATCTGTTAAAAAAACAAAATTATTCAC
ATTATATTGCTTACTTACTCTAATAAAAACCCCATTAAATGTTTTAACTGATTCTCGCTA
CTTGGCACATCCTTTCCCATCCTTTGTAACAGCTCATTTTATAGCCAATAAAAATGATCT
TATACATTTGTTCTTATTGATTCAACAAAAAATAAGAGCTAGACTCCATCCTTTCTTTAT
TACTCATATTTGTGCTCATTCCCATTTACCAAGACCCCTCAATTTAGGCAATGATTTGGC
TGATCGCCTCATCGCCCCTATATTTTCTTCCCCCAAACAAAAACATCAGCTCTTCTATAC
TAAAGCTAATAGACTACACGTTCAATATAAGATACCATTATAAACAGCTAAAAAAAACTT
GTTCAAGACTGTGCCACATGTGCCCCCTTTCATTTGACCAGTAGTCCCCAAGAGACAAAT
CCTAGAGGCTTACAAACAAATAAATTATGACAAGCTGATTTTACACATTCCAATCTGCCC
CCTTTTAAATTGTTATTTGTACTCATAAATACCTTTTCCAACTTCATTTAAGCAGTTCCT
TCCACCACCGAAACTACTAAAGCTGCCGTCACAGCTCTTCTGCAATATTTTTCAATGATG
GAGATCCCTGCTTCCATCAAAACTGACAATGGTCCTGCTTTTACAGCCAATGCTTTTCGT
AATTTCATACATCAATGAGATATTTGCCATCTTACTGGCATCCCGTACAACCCTTAATGT
CAAGCCATCATTAAATGGGCCCACCGTACACTCAAGTTCATTCTTAATAAACAAAACAGG
GAAAATAAACCAAGGGACACCTATGGACCTAAAGCCATTTTGCCTATAGCCCTTCTAACA
ATCAATTATTTTTAATTTGCCTCAATCAATTATTTTAATTTGCCTCTACATAGTCAGGAA
ACACAAACAGAGGGACATTTTTCTGATTCACCCTCACATATGCCTAAACAAACTGCTGTT
TGGGTTAAATGTCTTAATCAATGGTGGCCAGGAACACTTAAGTTCCTAGGCAAGGGATAT
TATCTTGTCATTTTAGATGATGGAACAGAGCAGTGGGTCCCACTCAAAAGAGTCAGAGGA
TGGACAGACCTTGCTCCACACCCCCTGACAGGTCACAGCACTAAAACAAAAATCGCAACA
GATGCCTTCTGAGCCTCCTTATGACAATGTATCTTAAAAAAAAAAAAATTGTCTGTGCCGG
GAATGGACTGGTCCACCTGTACAAAAAGGTTTGAAAGTTTCTGTTCACACCAACATGACT
TTTGTTGATTGGGGGCCTCATGGACTCTTTGTAAATTGCTCAAAATCACACACACACACA
CACACACACACAAATAATAATAACACCTGCCAATGGTATAATGTTTCAGCACCACGTTTT
AACAGAACGCAAACAAGACTTTGGCATCAACGCGATGAACTTTTGAACTGATACAATGGA
GATCTTTCACCTCCCAGACCCCAGATAATCAGTCCAGTCCTGGGGCCTGAACACTGGAAT
CTTTAGAAAATTCCTGCTTCTTTGTTTCAGTTTAGGGTCTCATATGCTTAAGCACATGTT
TCCATCCCACAAAATTATACTATTGAGTATAATTATACTGGTTATGTTCTGGCTTGTGTA
AATGCGCCCTATGTTTTTGCTATTAGACAATTTAAGAATAATGGCTCAATTCTCTCCTGT
ACTGATGGTCATTTGTATACTTGTTTAAATCATAGTGTGCCAATTAATGTTACTAAAAAC
AGTGTATTTTTAGTCTGGAAAAGGACTGATTTGTGGGTTCCAGTTAAGATTTCTGAACCT
TGGTCAGATTCCATGTTCTTGTCTTTTGTTCTGAGAAAATCCCTAAAAAAACCAAGCAAA
TTTTTTTTTTTTATTGGTTAGACTATAACTACCATAGTGGGTATTATTTCAATTGTAACT
GTTGGTACAGTTTCTAAAATGGCATTATATAATTCTATTCAAAATCATGATTTCATTACT
GCTTGTAAAAAGGATTCTTATGATCTCTGGGCCCAACAAGCTCAGATAGATCAACAAATA
CAAATCACATTTACCTTCAACAACATTTCCCCTGTGTTATATAACACGGACTTCTAAAAT
ATATTATCAGATAGTACTTCCTTTTTAAATCCAAAATATTGGGTACCCCATAGGATGGCT
TTTCATGCGCTGATCTATTTGTTTGTTATTGTTCTTATAAGATTACAAACATTATGTGCT
CAAGCCACCGTCACCCAAAAAGCGAGAGTAACAATGGCTACAACAGTCCTTACTCTTAAA
AAGAAAAATGTGGAAGAGCTTGACGGATTTAAGTAAATACTGAAAATGTATTTACTCATG

ACGAAGGGTCAATACACCCGGCCTTGAGGCGTTCGAACTTCCTTCTGACCACCTGTATCT
GAGAGCAAGGAATGTGGCTTCCTGATAAGACTCCCTTTAGAGGTTCGCCAAAGCTATGTT
ATGTCTGGGGAGATGGGAACTGTATTTTATGCTTGAATGCTTTAATGTTTACCTGGAAAG
GCTACGTACAAGTCTGCTGTAGCTATATTCTCTGAGAATTATAAAACTGCCCAGTTATAT
AATGAACTTTGTCAGTCCGATAGAGGCTGAGCCTGAGTGTCTTTTTCAGAGTGCAGTTAT
ACAAGCCTTATACCTCTGGAGTGGACTGAGACATTTGGCAGTCTTTTGGAATGGTGGCAG
GAACCCTGGAGCTCCTCTCGACTTTCCTGTTGAGAGCGCCTCCTCTTGAGATGTGACGGG
AATGCCAGGAATCCTTTCCCGACGAAGCAGGGAAAGGAATCCTCATCTCGAGCGGATGAG
GGAGAAACGTGGCTCTTCTTAAAGTTGGTGTGACCCTCGGTGTTCCTCTCGAGTGGAGAC
GGGTATATCGAGGAACTTCTTGAGTTGCAGCAAGGGTGTCAAGGACCCTTTCGAGGTTCA
ACAGGGAAGGTGTGATTTCCCTCGAGATGCCACAGTGGAAAAGGGCCTCATGTCGCCTGG
AGGGGAGAACCTCCTGGTTTTTCTCGAGTTGCGGTTGATTTCTCTCCAGTTATGACGGGG
ATCTCAGGGACTCACTCGTGTGGCCTCAGGAACGGCCAGTCTCCATGCGAGTTGACCAGG
GCCTCTCTGGATTCCTCTCCAGTC
>CAP_ERV_23
ATCAGGGAATGTTTAACAGGAGGATTCCTACATGCTGTTTCTCATAAATCCTCTGTTCCT
TATCAGTTTCTGTTGCCAGCAACCAGTGGTATGGCATGCCGCTCCCAGGACTGAGGTCAT
AGGAGGAGACAGGCTTGAATCTCCTTCAGTAAACATTCTTACAACAAAACTGCTTAGTCT
CGATCCTCTTTCTTGAGGTGTGGATTGTCTCCTGATCTTGTGACCATTATTAATCCCTGT
TCCCTTGGTAACGGTTGCTGTACGTTTGGTTTTCTGATCTTTATCATTGTCGAAAGAAAT
TTCTTGTACAACAGCCTATATATACTCACAGAAAGATCATGAAAGCACCTTTGCTCCATC
AGAGCTTGAGTCTTCGTGTCTTTCTTTCTTTCTTTTTCTCTCTCTCTCTTTCACTTTCTT
ATCATCGACTCCAGACTGCCAGGTTCCGGTCCATTAAAGGACCCCAACAAGTGGCGCCCA
AACAGGGACTTGGCATACGTGGCATTTCGGACAGAGTGGCTCAGGGGCTACTGAGGTTGG
GACCTATTGAGGTCGGTAAGTACTAAGACAGGGGTCAAACGGCCAGAAAGCCCTAACATT
TCTCTACTTTACTTCACCATTTGCTTAAAGCTCAGGAACTTTTGGTTTTGCGCCAATGAA
TAGAGGCTTGCTTTCAAACAGTGGTTAAATGCAGTCCTTGGTTTCCTGATAAAGGCAGTT
TTGATTTAGAAACTTGGCACCAGGTCAAAGAAAATGTTTAATGAGCTGCCAGGAAGGGAA
AAAACATTCCAATTGATTTATGGCCCCTATGGGCCCTCATTAAAGCTGTAATTCTGCCAT
TTCAAGGTAAATTCTAGCCCTCCCAATATTCGACAACAGACAGAACACTTATTACATGAA
TATAAGTTAAATGATAAAACTTTACAAAAGGTCCAATTAAAATAACGTAAAATATTTCAA
AAATTTCGTACTAATCCTATCCTGGCTACTCCAAATGCTCCTCCTCTCCCAACAGGCGCA
AGTCCAAAAGTCTCTCCTTTGCTAGAACCTAATAATTCTGATAACGTCTCTTGAGACACC
TTTTGACACCAAAACCGGCAATGCTTTTCTAGATAATAATGATAAGTCCTTAACACAGAC
TCATATTTGCAAAAAAATTGGCACTTACTTGCTCTCCTCCATGACAGAGGCTCTCTCAATT
ACTGGCTTTGCAATCACAAATCTCTGAAGCCAATGGTTTCTCTGCCTTTCCAGTTTTAAG
AAATCCTGATGCTCGAGGGCACATAATACCGCAATATGAATGTATTATTATTATTTTTTT
TACAAGCAACAGATGAAAAAGGCTATAACTATGTATAGTCCACATTCGCCTTTTACTAAA
GAGCTTCTAAATGTTGTGGCATTTTCTATTGGAAATTATTCTCTATGATTGGTGAGTTTT
AATAAAAGCTCTCCTTAAACCAGGAGAATATCTTCAATGGACAATGTGGTTTCATGATAA
TAGCCAGAGATCACGCTAACAAGAATGCTTGAGCTGGCACTCCCCCAAACCAAATTACTT
TTGAAATGTTAACTGGTGCCCGACAATTTGATACCATAGAAGCTCAAATACAATGCCCTC
CCTTGTTGCATGAAAATTAAAAACAGTGGCCCTTGAAGCTTGGGATCAAATTACTCCTCA
AGGAGAGCCTACAGGTAGCTACACTAAAACATTACAAGGACCTAATGAACTCCGGGAGTT
GGTGATGGACAGGGAGGCCTGGCGTGCTGCGATTCATGGGGTCACAAAGAGTCGGACACG

ACTGAGCAACTGAACTGAACTGAACTGAATGAAAATTAGGCCGATTTTTTTTAGCTAGAT
TAGAAACTGCTATTTCCCGTACTGTAATCGGAGAAGAAACCAAAAAACAGCTAGAGAAAT
TACTTGCTTATGAGAGTGCAAATCAGAGATGTCAAAAAGCTATTGCTCCAATTCGTGAGA
CTGGGACTATTACTGATTGTTTGAAGGCTTGTTGCAATCTAGGATCAGAAGCTCAAAAGA
TGCAAATGCTAGCTGAGACAATGGCTGCTGCCTTTAGAAAGGGAAATGAAGGAGGAGTTA
CATGTGGAGATAAAAACCATTTAAAAAGGGACTGCCCTAAGAAGGCTAATAAAAAACTTC
CAAGAACCTGCCCTCACTGCCATCGATATACCAGCCCTAAATGATTTTTTTCCTTTACCC
TCAAGCAGTCCCTTCTAGAGTACCTACTGGACTTTTTGGACCCCTACCCCCACAAACCTT
CGGTCTTTTACTTGGTCAATCTAGTTTGATTACTAAAGGAATTACTGTTCACCCTGGAAT
AATTGATTCAGATTATAAAGGAGAGATTAAAATTATGATGTCATCTCAGAGTCTATGGCA
ATTCAAAAAGGGGGATAAAATTGCTCAATTGCTTCTTTTGCCTTACATTTCTATTAACTC
CTCTAATAATGTACAGACAGGTGGATTCAGCAGTACAGATCAAAAACAATCCTTATAGAC
ATCATTGGTATCTAATTATGCCCTACCAAATATAAATATCAAAATTAATGGTAAAAGATT
TTCTGCTCTCCTCGACACTGGATCTGATATTACTATTATTTCAAACACTTATGGCCCAAA
TCCTGGCCTATATAAAAGGTCTCTTGCCAGATTGTGGGGATTTCTCAAACTAAAGTACAA
TAAGTCTATCAAAGTGTTCAAATATACCCATGTGAGGGACCAGAAGGCCAACCTGTAACA
TTAAGACCTTATGTGATAAATGCACCCCTTAATCTAATAGGAAGGGACTTACTTATGCAA
TGGCAAACTCAGATATACATTCCACATTTTTCCTAGGGGCCACTGCTCATTTAACAAACA
AAGCAATTATTAAAATAACTTGGAAGAATAACAAGCCTATTCGGACAGAGCAATGGCCCA
TTGTGACAGAAAAATTACAGGCTGCTAAAGAACTTACAGACACACAATTAGAATGAAAAC
ATATTGAGGAATCTTGCTCTCCTTGGAACTCTCCTATTTTTGTTATATATATATATATAA
AATCTAACAAATGGCATCTCTTAACAGACCTTAGAAAAGTTAATGCATCTATGAAACCTA
TGGGTGCATTATAACCAGGGATCCCATCACCTACTACTAGTCCTCAAAATTGGTACATTA
TCATTACTGATTTACAAGACTGCTTTTTAAATATACCTTTACACTCTTTAGACCGAGAGA
GATTCACTTTCTCTCTCCCTTTTCCTAATCACATTGGGCCTCATAAAAAAATTTCAATAGA
CTGTGTTACCTCAAGGTATGCTTAATAGTCCTACTATTTGTCAAAATTTTGTAGCCAAGG
CTTTATATCCAATGTGACAGCAATTCCCTCATGCTTATGTCATTAATAATACACTGTAAA
TACACAAAGGAGAAATGATATTTTTGACACTGAATAGCCATGATATTCTTTAGACTCCAG
AGAAAACTGATTAAAATAAAATCTGTTTTGAAATTGCAAAACTGGTTCTTCTTACACGTG
CAGTTAGGAAAAGGTTAACTTGTTAAAATACTGTTTTGGAGCTCAAATTCTGCCTGGGAA
ACAAAAACAGGCCTAAGAAAAAAACCTAAAGTTAACTGTTATCATGTCCTTGGGATACACA
GCCCACTCTGTCTGGGAGTCCAGCCATAAACAAATTGGTGGAACTCAGAAAAAAAAAAAA
TCAAAAAGATATTTTAAATTTCAAGTGGAAAATGATGTCTGTCTGTCTATCTAAAATTAT
CTATGTCTCAATGTATGTCTTTGTTTTTGGACAATATGAAGTTAATGAGCTCTATTTAAA
TTCAAGTTCACGTGAACGGAAAAATATTCAATAATAAATATAATGTTTATTTAAATATAA
ATATAATTTAAATACAATTGTTAATCTAATTAAGACATATCTTAAATTATCAACATTATA
TTATACTTTTGTTGTGCCTAGATTTAAGGTAAACTAAGTTTGTCAACAAAAAAGTAACTC
TTTATATAAATAAATATATAAATGAGATGAAAACTTTTAGATAAACTCTATTAAAAATAA
TTATATTTTAAAAATGTCTATCTAAAATAATCTCTCAGAATTGGGGTAACTTAAATTTCT
AGAATTGTACTAAACTAAATGATAAAAGTTTATTAAATAGCTAGGTCATTTCCAAATAAA
ATAAGATTTTAAAACATTAATTACTGAACACTAACTTCCTCTTACAAAAAGTTTTTCTTA
CAGAAAAACTAAAGAGATTTTAGACTATTAATAAATATATAAATATAAATATATATTTAT
ATAAAATATAAATATATATTTATATGAAATATGTTAATATAATAAATATATTCACCAATG
CTAATATACAAGATACTTCATAGTTGCTAAAGAAAAGTAAGATGTATGCTTTTAATAAAA
GGATATAAGAAATGGCAAATAAAATGATGAATACAAAAATATAAAAAAGGTTTATGACAA

ATGAAATAGAATTTTATGGCAAATGAATGTAACTCATTGCCCTGAACTTTCTTTCTCTTC
CTTCTTACATGTCTCATTCGATACAAATTCTTCTTTTATATGGGCCACACCTCTCCGAGG
TGAAGCTACATGACATGTTATAACCCACCTGTTAGCTTACTCTGCAATAATGAGAACACC
TAATTCTATAAAAACAGACAATGGCCCTGCCTATATTTCTAGGCAGTTCAAATAATTTTT
ACATTCATTCTCTATTAAACAGGTTACAGACATTCCTTATAATCCACAAACACAAGACAT
AGTTAAACAAACACAGTACACACTGAAACTACAAATAAAAAATTAAATAAAGGGGAATAA
ACAGGAACACTTTTATCTTCCTCATCCAGAAGAGACTTTGCTAGATTCCAATGCAATATA
TTCTTTAAGCCTATAACTATTGTTAATATAGCTTTATTTGTTTTAAATTTTTAAACTTAC
CGCAAGGAGAAATTTTGACCAAAGCAGAAAAGCATTTTGAGACACTGAAGGACACCTCCC
TTCTTTTGCCCATTTGGTATCAAGACGGGTTAACTAATCAATGGAAATCTAGGAAACTAA
TCTTACAGGGAAAGGGGTATGCTTGTATTTCTCCAGATGGATCCAACGAACTCACATGGC
TTCCTCTTCGGAAGATTTGACCCAAGGGGGCCCCCAACATTCAAACTAGAGACGAAACAA
CAAAGACCCCAAGAGGAAGAAATTCCAATACAAGCCATGGCAGATTTAAAAATTTCCAAA
AAACACCGCACTCGACGTCATAGACCTTATGATCTCCCTACTTGGGGACAGGTAAAACCC
CTTACTAATCAAGCTGAAAATCTGATTTCTCAACAGGGAATGCCTTGGAATCCTGAAAAT
ATTTTTGTTGCTATGCTTGCTTTGTTTGCTTTTGCTTCCCCCGCTCAGGCTGACTTGATT
AATCACACTTATTGGGCTTATATATCTAACCCCTCCTTTCATTGTAGGTTATAGAATGGA
CAGATATAAGACCAATCTCATCCACTAATGACTCAACACATATGCCCCCTCCTTGGAATT
TGGAGGCACCCTCTCATCCTGAGGACGAAGGAAGACTAACATTTCTCTAGGCTATGAAAT
CCTTCCTTTATGCATGGGCCCAACAAAATTATGTATTAATGTTAGTCAACAAACATGGGC
TTTCATCCTGCCTCCAGAAAGGAACTTCCACACATTGCTTGGACTGTTTACTGCCCTGTC
CTTTTATAAAAACCATGTCAATACTACATGTCAATACATACAAAAGTTAGAATGTAAGGG
GTTTACTTATAAAGACTTTAAATATACTCCTGTTTATTGAGATAAATGTCAAGATAAATC
AGGGAAATTAATGTTTATGGCCAATTACACCATTGTTAATTGGGGACCCCATAGTATGTG
TGTATCTAACTGCTTAGATGATATTAACAGCACTATGTGTGACTATGCTACTCAAGTAGC
ATAGAAGGTTACTAACACTACAATATAGCATTACCATGACAAAGGACTTCTTGGGTGGCT
TGAAGGTGGAATGGCCCCCCCTTGTCCTCAAATCATCCTCAATAAACAGATTGGGCCTGA
ACAATGGGACATGTGGAAACTTGCTGTGAGCACCAAAGAACTTGGAACTTGGACTGGACA
TTTCACAGGGACCAATCATAGTCATCGGAGAAGGCAATGGCACCCCACTCCAGTACTCTT
GCCTGGGAAATCCCATGGATGGGGGAGCCTGGTGGGCTGCAGTCCATGGGGTCACTAAGA
GTTGGACACTACTCAGCGACTTCACTCTCACTTTTCACTTTCATGCACTGGAGAAGGAAT
GGCAACCCACTCCAGTATTCTTGCCTGGAGAATACCAGGGACAGAGGAGCCTGGTGGGTT
TCCATCTATGGGGTCGCACAGAGTCGGACACGACTGATGCGACTTAGCAGCTGCAGCAGC
AGCAGCAGCAGCAGCAGCAGCAGCAGCAGCAGCAGCAGTTGTAGCCATAGGAACTAT
TTCTTTTGTTATAATCAATCATATTTCATACAAGCTTGTATTCCCCTTCCTTTTGTTCTA
GCTATAGGAAGTTTACAATTAAATGAGACTTTATGTTCTGTAACTTGTATAAATTGCAAA
TTATATACTTGTCTTAACTCTTCTGCTTTCTTAAGAAATGAATCCCTTTAGATTCTCTGA
TCTTGACGTAGTCTGTGGTTACCAATAAATCTCCAGCGGCCCTGGGAAGAAGGTCCCATG
GCTGGACTTGCTTCCTGGTTACTTACTAAACTGCTCTGGTGATCCAAACGATTCATTGGA
TGGCTGATTCTTGGCATTTTGGGATTAATAGCTGTTTGCACCACTGCTGCTGTCACTGGT
GTTGCTTTACAAACCTCAATTCAAACACATAATTTTATCCAAAATTGGACTAAAGATGCT
CATACTATGTGGGCCACTCAGGCTCAGATAGGTGAGGATATTCGAGATGAAATACAGGAA
CTAAAAACAGCCATCAAATGGGTTGGAGACCAATTAATAGATGAAAAAACAGGTGATGCT
AAAATGTGATTGGAATTCTACTCACTTTTGTGTTACTCCTGTTCAATTCAATCATAGTGC
CTACAACTGGGAACAAATCAAATTTCATTTACAAAACATACATAATAATGCTTCTCTGAA

TGTACAATTATTACAAAAAGAAATCTTTGAAACCTTTTCTAAAAATCTGCCCTCTTCCAC
TAATTTGAAAACTTTAGCTGAACAACTAGCTGATCAATTATCTGGGCTAGACCCATGCAG
ATGGTTTCAAAGCGTTACTCGCACCATCGGGTCTGGAACTGTAATTTTGGTAATTGTCTT
GATAATTATATTTGTCATTTACCATTGCCTTCATGCAAAAATTGTTAAAACTAGACAAAC
TCAAATGGTCAGAACCCTTTTTACAAATATTATAAATAAATAAGGGGGAATTATCAGGGA
ATGTTTAACAGGAGGATTCCTACATGCTGTTTCTCGTAAATCCTCTGTTCCTTTTCAGTT
TCTGTTGCCAGATACCAGTGGTATGGCACACTGCTCCCAGGACTGAGGTCATAGGATGAG
ACAGGCTTGAATCTCCTTCAGTAAACATTCT
>CAP_ERV_24
TGCGGGGGACTACCCGTGAAGGGTTAAGTCTTGGGAGCTGCTCGGCGTTATGCAGAGCCC
TAGGACATGTGCCTAAGCTCCCTGTCCCGCCACCCTCAAGAATTTTTATAGCCCTTAAGG
CTCCAAGATGTTTGGTTTCGGCAACATGTCATAGAAGATAGATTAGCTTATTGTGATCTG
TATACAATGGTACGGGTCTGGTGATTGTATCTGGAGATGAAAAACAATCTTGTAAAGGTC
AGAAATCACGTATTTTATCCTATATATGCTGCAGCATAATAAAGCAAGGTATCAGCCATT
TTGGTCTGATCCTCTCAACCCCATCTTTTGTCTCTCTCTTATCTTCTTAGCGGGGACGCT
CCGTTCTCTCCCTGTGCAGGTGCGACTCTTGCTTGTGCTGGCCGCGGCAGGTGGCGCCCA
ACGTGGGGCGGTTCGACAGTTTTCCTCGCCACTACTCTCATTAATTAAAAAGAGTGAGTA
TATAAGTATACAAGTGCATTACAATTGAGGAGGAGTAGTAAGGTATATAGTTGAGAGTAT
AAATATGGGACAGACGCATAGTCGTCAATTGTTTGTACATATGCTATCTGTAATGTTGAA
ACATCGGGGAATTACTGTTTCCAAACCTAAACTAATCAATTTTCTTTCATTTATTGAGGA
GGTTTGCCCTTGGTTCCCCAGAGAAGGTACAGTAAATTTGGAGACATGGAAGAAGGTAGG
GGAACAAATCCGGACTCATTATACTTTACATGGCCCTGAAAAAATCCCTGTTGAAACTTT
ATCCTTTTGGACACTAATTCGTGATTGTCTGGATTTTGATAATGATGAATTAAAACGTTT
AGGAAATTTATTAAAACAGGAAGAAAATCCTCTCCATGTTCCTGATTCGGAACCCAGGTA
TGCTGTTCCCGAGGGAGTTGAAGGTGATCCTCCGCTTCCTAATTTATTGCGTCCTTCGGA
TAATGATGATTCACTTTCCTCCACAGATGAGGCAGAATTAGACGAAGAAGCTGCTAAATA
CCATCAAGAAGATTGGGGTTTTTTAGCACAAGAAAAGGGGGCGTCAACATCTAAAGATGA
ATTGGTTGAATGTTTAAAAAACCTCACTATTGCTTTACAGAACTCAGGAATCAAGTTTCC
TAGTAACAATGCCAAATCTCCTTCTGCTCCGCCTCTTCCCCCTGCTTATGCTCCTTCTGT
TGTGGCTGGTCTCGATCCCCCTCCAGGGCCCTCTCCACCGTCTGAAAACATGTCTCCGCT
GCAGAAGGCATTGAGACAAGCACAGCGACTTGGTGAGGTTGTCTCTGATTTTTCTCTTGC
TTTTCCTGTCTTTGAAAATAACAACCAGCGTTATTATGAATCACTGCCTTTTAAGCAACT
AAAAGAGTTAAAGATTGCTTGTTCACAATACGGTCCTACCGCTCCATTCACCATTGCTAT
GATAGAAAATTTGGGTACTCAAGCTTTACCCCCAAATGATTGGAAACAAACAGCTAGGGC
TTGTCTTTCAGGGGGAGATTATTTACTATGGAAATCTGAATTTTTTGAACAATGTGCTCG
TATAGCTGATGTTAACCGACAGCAAAATATACAAACCTCCTATGAAATGTTGATTGGTGA
AGGCCCTTACCAGGCTACTGATACTCAACTTAATTTCTTACCTGGTGCATATGCACAAAT
ATCAAATGCAGCTCGGCAGGCATGGAAAAAACTTCCTAGCTCCAGTACTAAGACAGAAGA
TCTTTCAAAAGTCCGACAGGGACCTGATGAGCCTTACCAAGACTTCGTGGCACGGCTTTT
AGATACTATAGGTAAGATAATGTCAGATGAACAGGCTGGGATGTTATTGACAAAACAATT
GGCTTTTGAAAACGCTAACTCTGCCTGTCAAGCTGCCTTAAGACCTTATCGTAAAAAGGG
AGATCTGTCTGATTTTATTCGCATTTGTGCTGATATTGGACCCTCCTACATGCAAGGCAT
TGCTATGGCAGCAGCATTACAAGGAAAAAGCATAAAGGAGGTACTTTTTCAGCAGCAAGC
CCGGAATAAGAAAGGACTTCAAAAGTCAGGTAATTCTGGTTGTTTTGTTTGTGGTCAACC
TGGCCATCGGGCGGCAGTGTGCCCTCAAAAACAACAAAGCCCTGTTAACATTCCTAATTT

GTGCCCACGATGTAAAAAAGGAAAACATTGGGCACGGGATTGCCGTTCTAAAACGGATAT
TCAAGGTAATCCTTTACCCCCGGTTTCGGGAAACTGGGTGAGGGGCCAGCCCCTGGCCCC
GAAACAATGTTATGGGGCAACGCTGCAGGTTCCAAAAGAACCATTGCAGACCTCTGTCGA
GCCACAAGAGGCAGCGCGGGATTGGACCTCTGTGCCACCTCCTACACAGTACTAACTCCC
GAGATGGGGGTTCAAACCCTTGCCACAGGAGTGTTTGGGCCTTTACCTCCAGGGACAGCT
GGATTGCTCTTGGGGCGCAGCAGTGCGTCTTTAAAAGGAATACTTATTCACCCTGGTGTG
ATTGACTCTGATTATACAGGAGAGATAAAAATATTAGCCTCCGCTCCTAATAAAATTATT
GTAATCAATGCAGGACAACGTATAGCTCAACTCCTTTTAGTTCCATTAGTTATACAAGGA
AAAACAATTAATAGAGACCGTCAAGATAAAGGTTTCGGGTCATCTGACGCCTATTGGGTG
CAAAATGTTACCGAGGCACGACCAGAACTTGAGCTACGCATTAATGGTAAGCTTTTCCGC
GGAGTGCTTGATACAGGGGCCGATATTAGTGTTATTTCTGATAAATACTGGCCTACTACA
TGGCCTAAACAGATGGCTATTTCCACTCTTCAAGGTATTGGCCAAACTACCAATCCAGAA
CAAAGCTCGTCCCTTCTTACTTGGACAGATAAAGACGGCCATACAGGTCAATTTAAGCCT
TATATTCTGCCCCATCTTCCAGTTAATCTATGGGGGCGTGATATATTAAGCAAAATGGGT
GTTTATTTATATAGTCCTTCACCCACCGTAACAGATTTGATGTTAGATCAGGGATTACTT
CCAAATCAAGGTTTGGGTAAACAACATCAAGGCATCGTTTTACCCCTTGATTTAAAACCT
AATCAAGAGCGAAAAGGCTTGGGGTGTTTTCCCTAGGGACCTCTGATTCTCCTGTGACAC
ATGCCGATCCTATTGATTGGAAATCTGAGGAACCGGTATGGGTCGATCAGTGGCCCCTGA
CACAGGAAAAACTTTCTGCCGCACAACAGCTGGTGCAGGAACAGCTGAGGCTTGGACATA
TTGAACCCTCTACCTCTGCTTGGAATTCCCCAATTTTTGTTATTAAAAAGAAGTCTGGGA
AATGGAGATTGCTACAAGACCTTCGTAAGGTAAATGAAACAATGATGCATATGGGAGCCC
TACAACCTGGGTTACCCACTCCTTCTGCTATACCTGATAAATCCTATATCATTGTTATAG
ATTTAAAAGATTGTTTTTACACCATTCCTCTTGCACCTCAAGATTGTAAAAGATTTGCCT
TCAGTTTACCCTCTGTTAATTTCAAAGAGCCTATGCAACGTTATCAATGGAGAGTCCTCC
CGCAAGGAATGACTAATAGTCCTACGTTGTGTCAAAAATTTGTTGCTACAGCATTAGCTC
CCGTTCGTCAACGCTTTCCTCAGCTATATTTAGTTCATTATATGGATGATATATTACTAG
CTCATGCTGACGAACAGCTATTGTACCAAGCTTTTTCTATTCTAAAGAAACACTTAAACC
TTAATGGTCTTGTTATTGCTGATGAAAAAATTCAAACTCATTTTCCTTATAATTATTTGG
GTTTCTCCTTATATCCTCGCGTTTATAATACTCAATTAGTAAGATTACAGACTGACCATT
TAAAAACTCTAAATGATTTTCAAAAACTTCTAGGAGACATTAACTGGATACGCCCTTATT
TAAAACTACCCACTTATACCTTGCAGCCTTTGTTTGATATCCTTAAAGGTGATTCTGACC
CTGCGTCACCCCGAACACTTTCTTCAGAAGGGCGATCAGCCTTACAATCAATAGAGGAAG
CTATTAGACAACAACAGATTACTTATTGTGATTACCAACGATCATGGGGTTTATATATAC
TTCCTACCCCCGAGCACCCACAGGGGTTCTCTATCAAGATAAACCTTTGCGATGGATATA
TCTGTCTGCTACTCCAACTAAACATCTGCTCCCTTACTATGAACTTGTTGCAAAAATTGT
AGCAAAGGGACGTCACGAGGCCATCCAGTATTTTGGAATGGAACCCCCCTTCATTTGTGT
TCCTTATGCTTTAGAACAACAAGATTGGCTTTTTCAATTTTCAGATAATTGGTCTATAGC
TTTTGCAAATTACCCGGGACGGATTACTCATCATTACCCTTCTGATAAATTGTTACAATT
TGCTAGCTCTCATGCCTTTATTTTTCCAAAAATAGTTCGCCGACAACCTATTCCCGAAGC
GACACTTATATTTACAGATGGATCTTCTAATGGTACTGCAGCTTTAATTATTAACCATCA
AACTTATTACGCACATACCAGCTTTTCTTCTGCTCAGGTTGTGGAATTATTTGCAGTCCA
CCAAGCATTACTAACTGTACCTACTTCCTTTAATTTATTTACAGACAGCTCCTATGTGGT
CGGTGCCTTACAGATGATTGAAACTGTTCCAATTATCGGCACCACCTCTCCTGAAGTTCT
TAACTTATTTACATTAATTCAACAGGTCCTCCACTGTCGCCAACACCCCTGTTTCTTTGG
GCATATTCGTGCACACTCCACCCTTCCTGGTGCCCTCGTACAAGGCAATCACACTGTGGA

CGTTCTTACTAAACAAGTGTTTTTTCAATCAGCTATTGATGCAGCTCGAAAATCCCATAA
CTTACATCACCAAAATAGTCATTCTCTACGGTTGCAATTTAAAATTTCCCGTGAAGCTGC
ACGGCAAATTGTTAAATCTTGCACTACTTGTCCTCAATTCTTTGTTCTCCCTCAATATGG
TGTCAACCCTCGAGGTTTACGCCCTAATCACCTCTGGCAAACAGATGTTACTCACATTCC
TCAATTTGGGCGTCTTAAATATGTTCATGTCTCTATTGACACTTTTTCCAATTTTCTCAT
GGCCTCCCTTCACACTGGAGAATCGACACGTCACTGTATTCAACATTTGCTGTTTTGCTT
TTCTATTTCAGGAATCCCACAAAACCTTAAAACAGATAATGGACCTGGTTATACTAGCCG
TTCTTTTCAACGTTTTTGTCTTTCTTTTCAAATTCATCATAAAACAGGAATTCCATATAA
CCCACAGGGCCAAGGTATTGTGGAACGAGCTCATCAGCGTCTCAAACATCAACTATTAAA
ACAGAAAAAGGGGAATGACTTGTATAGCCCCTCACCGCATAATGCCTTGAATCATGCTCT
TTATGTTTTAAATTTTTTAACTTTAGACGCAGAAGGCAATTCAGCAGCCCAGCGTTTTTG
GGGAGAACGATCCTCATGCAAAAAACCACTTGTACGATGGAAGGATCCACTTACCAATCT
GTGGTATGGGCCAGACCCTGTACTAATATGGGGACGAGGGCATGTTTGTGTCTTTCCACA
GGATGCCGAAGCACCGCGTTGGATACCGGAAAGGCTGGTACGCGCGGCAGAGGAACTCCC
TGACACATCAGATGCGTCGCATGACACTGAGCGAGCCCACGAGTGAGCTGCCTACCCAGA
GGCAGATTGAGGCGTTGATGCGACATGCTTGGAATGAGGCTCATGTACAACCTCCAGTGA
CACCTATTAATATACTGATCATGTTATTATTATTGTTACAGCGGATACAAAACGGGGAGG
CTGCGGCTTTTTGGGCATACATTCCTGATCCGCCCATGATCCAATCCTTAGGATGGGATA
AAGAAACAGTACCTGTATATGTCAATGATACAAGACTTTTAGGAGGAAAATCAGATATTC
ACATTTCTCCTCAGCAAGCCAATATTTCCTTTTATGGTCTTACTACACAATACCCTATGT
GCTTTTCTTATCAATCACAGCATCCTCACTGTATACAGGTGTCAGCTGATATATCCTATC
CTCGAGTGACTATTTCTGGCATTGATGAAAAAACCGGAAAAAGATCATACCGTAACGGAA
CTGGACCTCTCGACATTCCGTTTTGCGGCAGACGTCTAAGCATCGGCGTAGGAATAGACA
TTCCTTGGACTTTATGTCGAGCACGGGTCGCATCAGTGTATAACATCAACAATGCCAATG
CCATCCTTTTATGGGACTGGGCACCTGGGGGAAAACCTGATTTCCCCGAATATCGAGGAC
AGCATCCACCCATTCTCTCTGTAAACACTGCTCCTATATTTCAAACTGAACTGTGGAAAC
TTTTGGCTGCTTTTGGTCATGGCAATAGTCTATATTTACAACCCAATATTAGTGGGAGTA
AATATGGAAATGTAGGAGTTACGGGGTTTTTATATCCTCGAGCTTGTGTCCCTTACCCGT
TTATGTTGATACAAGGCCATATAGAAATAACACTGTCATTGAATATTTATCATTTAAATT
GTTCTAATTGCATACTTACTAATTGCATAAGAGGTGTTGCTAAAGGAGAACAAGTTATAA
TAGTAAAACAACCTGCTTTTGTCATGTTACCTGTTGAAATAACTGAAGAATGGTATGATG
AGACTGCTTTAGAATTACTACAACGCATTAACACGGCTCTTAGCCGCAAGGAAAGAAGTG
TGAGCCTGATTATTCTGGGTATAGTATCTTTAATCACCCTTATAGCAACTGCTGTTACTG
CTTCTGTATCCTTAGCACAATCCATTCAAGCTGCTCATACTGTAGATTCCTTGTCATATA
ATGTTACTAAAGTAATGGGAACTCAAGAAGATATAGATAAAAAGATAGAAGATAGATTAT
CAGCTTTATATGATGTAGTTAGAGTTCTAGGAGACCAAGTTCAGAGTATTAATTTTCGCA
TGAAAATTCAATGCCATGCTAATTATAAATGGATTTGTGTTACAAAAAAGCCTTACAATA
CATCTGATTTTCCGTGGGATAAGGTGAAAAAACATCTACAAGGAATCTGGTTTAATACTA
ATGTTTCTTTAGATCTTTTACAATTGCATAATGAAATTCTTGACATCGAAAATGCTCCAA
AAGCTACTTTGAATATAGCTGATACTGTTGATAATTTTTTACAAAATTTATTTTCTAACT
TTCCTAGCCTTCATTCACTGTGGCGAAGTATAATTGCTGTGGGCACGGTTCTGACTGTTG
TGCTTATCATAATTTGTCTAACTCCTTGTCTTATTCGTAGTATTGTTAAAGAATTTCTAC
AGATGAGAGTTTTAATACATAAAAACATGTTGCAACACCGACATCTTATGGAGCTTTTAA
AAAATAAAGAGAGGGGAGCTGCGGGGGACTACCCGTGAAGGGTTAAGTCTTGGGAGCTGC
TCGGCGTTATGCAGAGCCCTAGGACATGTGCCTAAGCTCCCTGTCCCGCCACCCTCAAGA

ATTTTTATAGCCCTTAAGGCTCCAAGATGTTTGGTTTCGGCAACATGTCATAGAAGATAG
ATTAGCTTATTGTGATCTGTATACAATGGTACGGGTCTGGTGATTGTATCTGGAGATGAA
AAACAATCTTGTAAAGGTCAGAAATCACGTATTTTATCCTATATATGCTGCAGCATAATA
AAGCAAGGTATCAGCCATTTTGGTCTGATCCTCTCAACCCCATCTTTTGTCTCTCTCTTA
TCTTCTTAGCGGGGACGCTCCGTTCTCTCCCTGTGCAGGTGCGACTCTTGCTTGTGCTGG
CCGCGGCAAG
>CAP_ERV_25
TGAAGGAGCTGGGAGGCTTTAAGCAAATACTGAGAATGTATTTGCTCCACTCGTGACGAA
GGTTGGAAGCTGGGACGAGCATAACAAAGGGTTATGAGCGTTCACCAAGTGCCCAAGGCT
GGGAACGGATGACGAAGGGTCATACGCCCGGCCTTGAAGCGTTCGGAGGCTTCCTTCTGA
CCACCTGTTTCTGGGAGCAAGGACTGTTGTTTCATGATAAGACTCCCTTTAGAGTTTCGC
CAAAGCTATGTTACGGCTTGGGTGGTGGGAACTGTATTTTATGCTTGAATGCTTTGACGT
TTTATCGAGAAAGGTTACGTGCAAGTCTGCTTTATGCTCTGCTCCCTGAGACCATATATC
TGCGAAAGCTGGATAATAAAATTTGTCAGTCCACTAGAGGCTGTCCCTGAGTGTTCCTTT
CAGAGTGTGGTTCTCCGAACCTTACAACTGGTGCCCAGTGTGGGGCTCAAGCAGCAGACT
GATTTTAAACAAGGACTGCATTCCTGCAGAAGCAAGGTAAGCAGGATGGGACATCAGACA
AGTAAAATTCCTTTTGTTCATCTCATGCATCATTTCTTGAAACAATACGGGGTTAATCTG
CCTGAAGAGCGGTTAACTAGCTGTTACCAGACAGTGCTTGAATATAATCCATGGTTTCCG
GAGGAAGGAACCTTGGATTTACAAACTTGGACCAGAGTAAAGAATAATGTTCTAAAAGCT
TATACACAGGGGGTAAAAATTCCCCCACAATGGTGGGTAACTTGGTCTCTTTTACAGGCC
ATTATGGAAAAATTAGATGGTTCAGGAGGAGATTTAGAGGTTGAAACTGTTCGATCCCTG
CATGAATGTGAATTAGAAGAGAAAGATCTATCAGAGGTTTTAAATCAGAAAAATGTCATG
CTAGGGCAGATTACGGACAAACAAGAGTCACAGGTGCTGAAAGCTGTTAAACAATCAACT
TTGCCAGAGGTCCCTGATCCGCCGCGAGCTCATATTAACAAGCCTCTAACACCGGCCTTT
CCGCCCACTGCCACGCTGTCTGCTGCTGCTTCTGCTCCCCTATTTCCTAGCATTCCTCCT
CCGGACATGCCTCTTTGGGCGTGGGCTTTTCCAGTCCAATTTAATAATCCTCAACCTGGA
CATAATCAGTGGCAATCACCTGATTTCGGTTTGCTCACACAATTCAAAAAGGCATGTACG
CTATATGGTCCTACCTCACCCTACTGTATGGAATTTCTTAGGGGCTAAGCAGATCAATGG
CTTCATGCAGATTTTTTTACAGTTGCTAAAATGGTTATGACTCCACAACAGCTACTACAA
TGGCAAATGTGGGTCACGGATGAAGCCAAATTAATCTTGCAAGAACAGCAAAGCAAGGGA
AACCCTACTGGACTAAATTTTGAAATTCTCACCGGCACCAAAGCTATGGCTAAAACTGCT
GCGCAATTACAATTTGTGCAGCTGCCCACGTTATACTGGATTAAGGAAGCAGCTATCAGA
GCATGGGCTAAAATTGACAGTTCCACCTCTGATGGATCTTTTGTAAAAATACTGCAGGGA
CCAACTGAAGAATATGCTCAATTTATTGGTAAATTGAAGGAGGCCATTGATCACAGTCTT
AAGGATGCATCTTTGTGAGAAATCATTTTGAAACAACTGGCTTTTGACAGTGCTAATGAA
GATTGTCAGGCTATTATCAGACCTATTAGGGAGCAAGGAGGAATTATGGAATACTTGAAA
GCCTGCAGGAATGTGGGGACGATTCAACATAAAGCTAAAATAGCCACTTTAGAAACCTTA
AATGTTTCCCAAAAGTCTAAAGTTATATGTTTTAGCTGCAGCAAGCCGGGACACATGTGG
AAGCAGTGTCGCTTGCCTCGGCAAACAGGTCTTTCTCCTGACAAAGGAGGGGCTATTAAA
ACTAAGCCCCCCGGGCTCTACCCAAGATGTAAAAAGGGGAATCATTGGCTGAGTGAATGC
TGCTCTAGATTTGATAAACAAGGTAATGCTTTACCCATTCAGACACCTCCTTCGGGAAAT
TAGAACAAGGGCTCTCCTCTAGCTCCGGTAAACAAGGAGGACAACCAAATATTACTGAAT
TAACAGTGGCTACCAGACATAGCACATGTGTAGACATTCCTGCTCCCCAAGATATGGAAT
TATTAATGGTAAATAATCTTATGAAAATCTTAACTGGATATTTTGGCCCTATACCAAAAA
ATACTGTAGGCCTCCTGTTGAGACGAAGCAGCAACACCATGTGCAGGATAATTGTACATA

CTGGGATCACTGATGAAGATTACACGGGTGAAACTGCAGTAATATTACATGTGACTTGCA
ACTTGTATTTACAAAAGGGTGACAGATTTGCTCAGCTATTGCTATTACCTTATGTCCCCC
CACTTAATAGAAAAGCAGACACCAAAACAGGTGGCTTTGGGAGTACCAACATTACTGCAG
CCCTTTCTAACTGTTATAAAAAAAAATCAATACGCCCATGTTAAAATTAAAAATCAGAGG
AAGAACTTTGGAAGGAATGTTAGACACTGGGGCAGATGTTTCCATCATAAGAACAAAGGA
ATGGCCTTCAGATTGGCCTGCAGTTTTAGCCTCACACCAGTTGGTGGGAATAAGAACTGC
AGATGCAGCTCAAACTTATGTTAGTTCATCTTATTTACAAGCCCTGGGCCCTGATCAATT
AGTCGCTTACATTAAATCGTATATTGCCCCATTACCATTAAATCTGTAGGGAAGAGACTA
CAACAAACTCAGGCGACTACACAATTGAATAAGAACCTTTTTCTTAGGGGTCACTAAGAT
AAAGCCACTGAAGTTAGAATGGAAGTCTGATAAACCTATCTGGACAGCTCAATGGCCCCT
ATCAGAAGAGAAACTGTCTGCTTTGCATACTTTGATGGCTGAACTACTACAACAAAATAG
AATAGAAACTACTCAATCACCATGGAACTCACCAGTTTTTGTCATTAAAAAGAAATCAGG
TAAATGGAAAATGCTAACAGATTTAAGGAATATTAACGCTATAATGACTCCTATGGGAGC
ATTACAACCAGGACTCCCAAGCCCTGCTATGGTCCCTAAGGACTGGGCTATTATGATTAT
TGATTTAAAAGATTGATTTTTCACTATACCTTTATATCCCGATGACAGACAACGCTTTGC
CTTCTCAATACCTTCCATTAATAATCAATCTCCTGTTCAACAGTATCAATGGAAGGTCCT
GCCTCAAGGTATGATGAACTCCCCTACGGTCTGTCAATTCGTTGTTGATAAAATTTTGCA
GCCCATCAGACAGCAATTCCCTGAGGCATATCTCATTCATTACATGGATGACATTTTATT
GGCTTCTCCCTCAGAATCTCAATTAAGTTTATTAGGTAATGAGGTCATAACTAATTTAAC
TAATCATGGGCTGCTAATAGCAGAAGATAAATTGCAACACCATTCCCCTTTTAAATATCC
TGGATATCTTATGGACTGCTGCACTGTAAAGCCACAGAAACTTTCTATCAGAAGGGATAA
TTTACAAACACTTAATGACTTCCAGAAACTTCTTGGGGATATTAATTGGCTACAACCTAC
CTCGGGAATGCCCACATACGCTTTACAAAACTTATTCAAATATTAGAAGGTTCCTCTGAT
TTGAATAGTCCCCGACAACTTACCCCTGAGGCTGAGAAAGAATTACAATTGGTAAAACAG
AGAATTCAACAAGCATTTGTTTACCGTATTAATTACAATTCCCCTTTTCAGATATATGTC
TTTGGTACTAAGATATCTCCTACTGCCATTATAGTGCAGGATAATTACCCTATTGAATGG
GTATATCTCCATTCCAAACAGACTAAACACATTGTTTCCTATATAGACTTAACAGGGAAA
ATCATTTTTCTTGCACACTCTCGCTTATGCACTGTAGCTGGATATAACCCTACTAAGATT
TACCTACCTTTAGCAAAAACAGAAATTCATAATGCTCTTCAGGTGTATACTACCATTCAG
ATAGCCCTTGCTGATTATTCAGGGGAATTATTGGCCAACCCACCTAAAAGAAAATTATGG
AATTTCTTACAAAACACTTCTTTTATCATCAACAATATTGTTTCTGAACACCCTCTCATG
AATGCACCTAACTATTTCATAGATGGAAATAAGGCAGGATAGACAGCCATAGTAGGTCCC
AACCTGCAAGAGAAAATTAAAAGTCCTTATCAATCCGTTCAAAAAACCGAATTATTCACA
TTATATTGTTTACCTACTCTAATAAAAACCCCATTGAATGTTTTAACTGATTCTCGCTAC
GTGGCACATCTTTTTCCATCCTTTGTAATGGCTCATTTTATATCCAATGAAAGTAATCTT
ATACATTTGTTCTTATTGATTCAGCAAGAAATAAGAGCTAGACTCCATCCCTTCTTCATT
ACTCACATTCGTGCTCATTCCCATTTGCCAGGACCCCTCAGTTTAGGCAATGATTTGGCT
GATCGCCTCATCGCCTCTATATTTTCTTCCCCCGAACAGGAACATCAGCTCTTTATACTA
ACGCAATAGACTACACATTCAATATAAAATACCATTACAAACGGCTAGAAAAATTGTTCG
GGACTATGCCACATGTGCCCCCTTTCATTTGACCACTAGTCCCCAAGGGACTAATCCTAG
AGGCTTACAAGCAAATGAATCATGGCAAGCTGATTTTACACATTACAAACTGCCCCCTTT
TAAATTGTTATTTGTAGTCATAGATACATTTTCCAGCTTCATTTGGGCAGTTCCTTCCAC
CGCCAAGACTACTAAAGCTGCCGTCACAGCTCTTCTGCAATGTTTTTCAATGATGGGGAT
CCCTGCCTCCATCAAAACGGACAATGGTCCTGCTTTTACAGCCAATGCTTTTCGTGATTT
CATGCATCAGTGGGGTATTTGCCATCTTACTGGCATCCCGTACAACCCTCAAGGCCAAGC

CATCATTGAACGGGCCCACCGTACACTCAAGCTCGTTCTTAATAAACAAAACAGGGGGAA
TAAGCTAAGGGACCCCTATGGACCTAAAGCCATCTTGCCTATAGCCCTTTTAACAATCAT
TTGTTTTAATTGGCCTCTACACAGTCAGGAAACACGAGCAGAGCGACATTTTTCTGATTC
ACCCTCACGTATGCCTGAACAAACTGTTGTTTGTTCTTGATCAATGGCGGCCAGGAACAC
TTAAGCTCCTAGGCAAGGGATGTTGTCTTGTCATTTTAGATGATGGAACCGAACAGTGGG
TCCCAATCAGAAGAGTCAGAAGACGGACAGACCTTGCTCCACACCCCTCGACCGGTAACA
GCACTAAAACGGAAATTGCAACAGATGACATTGAAAGACAGCAAGAAAATGAAACGTCCC
CAATGCAATGTGCCTTTTCCGACCTGGGCTCAAATGAAAAATCTGTCAAGACGAGCTGAG
GATACTCTGTTAATGACCAATAGCGAGGTAACACCGGAAAAACTGTTGCTGGCCATGATG
GCCGTTTTAACCTGTGCTTCTGGGGTAAGTGGTAATTACACCGATTGGGCTTATATCCCC
GACCCGCCTCTTTTACAAGTGGTGGATTGGACAGAATCATCTCCTGTGGTTTTCACAAGT
GATAGCTTACATTTTCCTGCCCCCGGCTGGATCTAGGACCACGAATAAAAGGAGGAGGAG
GAAAGACAGTTAACTTAACACTTACGTATCCAGTGCTCCCCATCTGTTTTGGTGCCCTCC
CTTTGTGTCTCCACCTATATCCACAATGGTGGGCTTACTCTTTCTCAAATGGCTCTTTCC
GTCTTGGAATGTTCGCTACCATGACCTTTATTCTTAACTGGACAGACTACCTACCATGGA
CAAATAGCCAACTTCACCCACTATCTATTTAGGCCTGCTGAGCCTCCTTGCGACAATGTA
TCTTGGAAGAAAAAATTGTCTGTGCTGGGAATGGACTGGTCCACCTGTAGAGAGTTTGGA
AAAGTCTCTGTTCATGCCATCATGACTTGTGTTGATTGGGGGCATCATGGACTCTTTGTG
AATTGCTCAGAATCACAAGAAAATAATTACACCTGCCCATGGTGTAATGTTTCAGCACCA
CATTTTAACAGAACACAAACAGGACTCTGGCATCAACGGGATGAACTTTTGAACTGGTAC
AACGGAGGTCTTTCACCTCCCAGACCCCAGCTAATTAGTCCAGTCCCGGGGCCTGAACAC
TGGCATCTTTGGAAAATTCCTGCTTGCTTGTCCCGGTTTAGCGTCTCATATGCTTATGCA
CATGTTTCCATCCCACAAAATTATACTATTGAGTATAATTATACCAGTTATGTTCGTTCT
TGTGTAAATGCGCCCTATCTTTTTGCTATTAGACAGTTTAGGAGTAATGGCTCAATTCCG
TCCTGTACTGAGTGTTGTTTACTTGCTTAAACCATAGTGTGCCAATTAATGTTAGTAAAG
ACAGTGGTTTTTTAGTCTGGCAAAGGACTGATTTGTGGGTTCCAGTTAAGATTTCTGAAC
CCTGGTCAGATTCCATGTTGTTGTTTTTCGTTCTGAGAGAATCCCTAAAAAGAAGCAAAT
GCTTTATTGGCTGGATTGTAGCTGCCATAGTGGGTATTATTTCAGTTGTAACTGTTGGTA
CAGTTTCTGGAATGGCATTGTATAATTCTATTCAAAATCATGATTTCATTACTGCTTGGA
AAAAGGATTCTCATGAGCTCTGGGCCCGGCAAGCTCAGATAGATCAACAAATACAAACAC
GCTTAGATGACCTACAAGCCGCCCTTATGTATGTGGGGGATGATCTGCATACTTTACAGG
TACAATTGAAGTTGTGGTGTCACTGGAATTTCACTACTTTCTGTTTGACCAATATGCCAT
ACAATGCCACTGAATATCCTTGGGAACAAATAAAGTTACATCTTTTAGGTTCGAAATCAA
ACACTAGTCTAGATATACAGAAACTGAAGCAACAAATCACGTCTACTACCTTCAGCAGCA
TTCCCCCTGTTATATAACACTGACTTCTATAATACACTATCCGCTAGTACTTCCTTTCTA
AATCCAAAAAATTGGGTACCCCATACAATGGCTTCTTACGCGCTGATCTGTTTATTGTTT
GTTACTGTTCTTATAGGATTCCGAACGTTATGTGCTCAAGCCACCGCTGCCCAAAAAGCA
GGAGTAACCATAGCTGCAGCAGTTCTTGCTCTTGAGGAAAAAGGGAGGAAATATGGAGGA
GCTGGGAGGCTTTAAGCAAATACTGAGAATGTATTTGCTCCACTCGTGACGAAGGTTGGA
AGCTGGGACGAGCATAACAAAGGGTTATGAGCGTTCACCAAGTGCCCAAGGCTGGGAACG
GATGACGAAGGGTCATACGCCCGGCCTTGAAGCATTCGGAGGCTTCCTTCTGACCACCTG
TTTCTGGGAGCAAGGACTGTTGTTTCATGATAAGACTCCCTTTAGAGTTTCGCCAAAGCT
ATGTTACGGCTTGGGTGGTGGGAACTGTATTTTATGCTTGAATGCTTTGATGTTTTATTG
AGAAAGGTTACGTGCAAGTCTGCTTTATGCTCTGCTCCCTGAGACCATGTATCTGCGAAA
ACTGGATAATAAAATTTGTCAGTCCACTAGAGGCTGTCCCTGAGTGTTCCTTTCAGAGTG

TGGTTCTCCGA
>CAP_ERV_26
TGTAGCGAATATGCTGTGCACAGGCCTGTGCTATAATTAACAGGGCTTCTTTGAAGAATA
AGAATGACCTTCTCATGACCCCCAGGCAAAGGGCTAGGAGCAGTAAAAAGTACCTTGTGG
AAAAACATCTTGAAAACAAGATGCTGAAATAGTGATGTGACTGATTGAAAACCATTAGTG
ACTATTCTCATGACCAGAGCCTTGAAGGAGTTGCTGAGAAAGGTCATCACTAGCTGAAGG
GCTAAACATGGTCATGGAAGGCCTGAACGTTTGACATTGGGGACTGTGCATTGTATATCG
TTATCCCCAATCTGAGATTGTAAAAACAGATGTCTTTAGAGCACGTACAAGGAAGTAGAT
GTTAGCAATTTAAAAAAAAAAAAAAAGCCTACAAGGATCAGGATCCTGGCTTTTGCCTGTG
AATGGCATCACCACCCCCGCACCACCCCCCCCCGACCCTCACCTTATTTCTGAGTCTTAT
TTTTCTTTATTCTTCTGTGGCACTGGTCCCTCAGGTTGGTTTGTTGTTGGGCTAGTCCGA
ACATTTGGCACCCAAACAGGGACTTGAGGGCATGAGTAGCTTTCGAGGTTTTTTTGACAG
TGCCAGAAAATATGGGGAATTCCAGCTTAGAGATAGAAGGACGGTGGAAGTACTTGGTGA
GTACACCCCCATGGATTTGTCAATCAGGGACACAATGGGGCAATCTCCTTCTAAGCATTC
TGACTACACTGTGCTTCTAAAAACCTTACTACATAGCTCTGGAGTAAAAGTGAAAGAAGA
GAATTTGCAGGAATTATTTCAGGCAATTCATAAGCACTGTTACTGGTTGGACCCAGAAAA
AAGGGAATTTGCTTCTTAATGGTGGAAAGAAGTGATGCGTTGTTTGTGTACAGCCTATCA
GTCTGGGGAACCCATTCCTATAAGTGTGTGGTCTCTTTGTAACCTTATTTACACTACTTT
GGCTCCTTTACAGTCTGAACAGTCTGACTCTTCTGATTCAGAATCTGATACTCCTGCTCC
TCCTGCATCTCAGGAGTCAACCTGTCTGGCCAGCTCATATTTATGAAAATCTGGATAAAG
AGAAATGTAATTCTTTGGAAGGGGAGAGAGACACTCAATTTTTGAGTCCTAAATCCCATG
TTAACTATGGTACTGCACAACATCCTAAAGCTTTCCCTTTATCACCAGTCTCACAGCCTT
TGGCTCCTTGACCAGAGCCCCCTATAGTTCAGAAACCCCCTCCACTAGATCCCGATACAC
CATGGGATGATGCGGTATTTAGAGCCACAGTGACAATACCAAAGACCTGCTCTGCTGGTG
CAGACCAATATCTGCACCTGCTCTCTCATCTTTTCAGCAAGCTGTATGAGCAGCAAGGTG
AGAAGGCGACCTTGATACTTTGGTTTTGCCTGTTACCGTGATCCAGGTGCCCCTTAATGC
TCAGCTTCCACAAGGCGGGGCACAGTTTGAACATGCTCTCTCTTTTAAAACTATTAAGGA
GAGTAAACAGGCATACACGCAATATGCAGCTACATCTCCTTACACACAGGTCTTACTCAG
GGGTTGGCTCAGTCTGAAGACTCATTCCATATGATTGGGAAATGATTGCTAGAACTTGCT
TATCCACTTCGGAATTTTTACAGTTTCAAACATGGTGGCAAGGTGAAGACTGTCAACAGG
CTCAAACAAATGCAGCTGCTAATCCTCCTCTTAATATTACTGTGGAACAATTAATGGGGT
CTGGGCATATCAAGAGGTTCAGAGACAACTACAATTCGATGATCAACTTATAGGGCATGC
GAAAAGGTCAGCCCCCAGGACAATCTCATCCTTCTTCTGTTAATGTAAAACAATCTAATG
GAGAACGTTATACTGATTTTATCACCCAATTAAGACGAAATCTGGTGAGAACTGTTGTTC
AAATTGAATTAAAGGATATGTTATTATAGATGCTTGCCTATGATAATACAAATTCAGAAT
GTCAAAGGTTTTACAGCCTCATAAAGCACAAGGAAAACCTCACAAGGATTATATTAAGGC
TTGTCATGATATTGGGTCAGAACCCTATAAGATGCGATTGTTGGCTCAAGCTATTATGAG
CTTGAAACAACAAGCAAATCAGCAGGTTAAATGTTTTAGTTGCGGTAAGAGACGACATGT
GCAGAAAAAATTGCAAGACTAATAAAAATACACCTAATAAGCCTACTCCCACAAATGAAAA
GAAAACTGGATTATGCCCTTGCTGAAATAAAAGCAATCATTAGGCTAATCAGTGTAGATC
TAAATTTCATAAAAATAGATCCCCTTTGTTAGGAAACTGAAAGAAAGGCCCCACTCAGGG
CCCATCAAACAACATGAGCTCTTAACAAACCTGGAACGCTGTTCCTTTTGTGTACCCAGT
CAGCGAACAGCAACCTCATCCAGCCCTTCCAAACCATACACCTGGGTTACCGACCTCCTT
CCTGCAACATCCAGGAGCACAACATTAGATGTCCCCGCCATCGATAATTTACTTCTTATG
CCAGCTATGGGAATTTATAAAATCCCTACTGGAATTAAAGGTCCAATACCTAAAGGAAGT

GTGGGGTTATTATTAGGTTATTATTAGTAGTTTAACAAGTAAGGGAGTTCAAGTCCTAAT
GGGGGTAATAGATGAAGATTATGAGGGCAAATTTTATATTATGATGAAAACAGAGTATCC
TTATCAAATAATAAAAGGGGATCGTAAAGCTCAATTGTTACTATTACCTTATATCACTAC
AAGTAAATCTCATATTAAAAGAACTGAAGGATTTGAAAGTGCAGGAAAACAGGTATACTG
GCAAACTTTTGTGTCTGATTCTAGACCCACATTATTTGTATATATCAATAATAAGCAATT
TTCAGGACTTGTACATACCGGAGCTGATATTTCTATAATATCTAAAAAGCATTGGCCAGT
TTCTTGGTCCTTAACAGATGTCCCTATTATGTTAACTGGAATTGGAACTATGCAAACTAT
TCAAAAAAGTACTAATATTTTGACCTGTTCAGGCCCCAAACAACAACCTGCAACTTTACA
ACCTTTAGTTGCAGATATCCCTATTAATTTATGGGGACAAGATCTATTGATGCAATGGAG
AGCTTATGTAACAATTCCTACTATATCTTCTCAAGCTAAACAAATTACGGTAAATATGAG
ATACAATCCTGTTCAAAATACATAAGCTTTTTAGTGGAGGCCACTGCCAAACAACAAAAA
CAGCACTTAAGTTGACATGGAAATCTGATGAGCCTATTTGGACAGAGCAGTGGCCCCTAT
CACGTGAAAAGTTACAGGCTCTTGAACAGCTAGTAGAGGAACAATTATCTCTTAATCATA
TTGCCTCAACTACTAGTCCTTGGAATACCCCGTCTTTGTTATTAAGAAGAAATCTCTGAA
GTGGAGAATGTTAACCAATTTAAGAAAAACTAATGCTATAATCGAACCTACGGGTGCTTT
TCAACCAGGCATTCCATCACCTTCTATGATACCATGAAATTGGAAAATAAAAATCATTGA
TCTTAAAGATTGTTTTTATACTATTCTTCTACAAGAATCTGATGCTCTCCATTTTGCTTT
TACTATATTGTCAATTAATAATAAAAAATCAGTGTCTCAATATTACTGGAAGGTATTGCC
ACAAGGAATGCATAATAGCCTTATATTATGTCAATTATACATCTCCCAACCACTTAAATT
TATTAGAAAACAATTCCTTCATGCTTCTATTATCCACTATATGAATGATATTCTTTTAGC
TTCTCCTTCTGCTGAGGAACTTCAATGTATTTTCTTAAATACAGACGTTAAATTAAAAGA
ATATGGGTTATATATTGCTGTTGATAAATCATAAAAGCAGGAACCCACATACCTATTCAG
GATATATATTGCAAAGAAATATTAAACCACAAAAAAATACGAATTTGAACTGACTCCTTG
AAATTTTTAAGTGATTTTCAGAAATTATTAGGAGATATAAATTGGTTACAACCATCTTTA
GGAATTACCAATCATTCCTTAACACACATATTTCAAGTCCTACAAGGAGAATCTGATTTA
AACAGACCCCGTGTACTAACAGAACAGGCTAAACAGAAATTGGATCTTGTAAATCAAGCT
ATACAACAAAGACAACTTAAACAAGTAGATCTGACTGTACCAATTTCTTTACTTATTCTT
CCTACTTTAGATTCCCCTACAGGAATATTATGACAACTGCAGAGTATCCTTGAATGGGGA
ATTTTATCCCACACTCCTAAGAAAATAAGGACTACTTACATAATGCTTATTTCCTTTCTT
ATTTCAAAAATGAGATCCAGATGTATACAACTTTCTGGTTATGATCCGTATGAAATTGTT
TTTCCTTTCACTAATGATCAAATCCATAGATTATTTGTTACCAGTATAGATTGTCAAATT
GCCATTGCTGATTTCATAGGTGAGGTTAATAACCATTTTCCAAAGTCACCTTTGATTACT
ATTGCTGCTAAACCAAATGGATAATTCCTATTATTACTAAAAAATTTACTCTTGCTAATA
CCCCCATGTATTTTACTGACACAAATAAAACTCTCAAAGCCGGTTATATTGGACCCACAA
CAAAGGTTTATCAATCTACCCTAGCCACTGTTCAACAAGATGAATTAGAGGCTATTGCTA
CCTTATTACAGGATGTTTCTAAATATTATATATTATATATAGCTTTAAATATTATATCAG
ATTATAAATATGCTGTACAGATAACACAAATTATTGAAACCATATCTTTACCAAAGGCTT
CTTCTAAATCGTCTATTATTACAATATTATCCCAATTACAAAATATAGTAAGGCTTCGTT
CTGCCCCTTTTTACATTACACATATCAGATCTCATTCCAAATTGCCTGGACCATTGGCAA
AGGGCCATGATTCCATCAACTCTTTGTTAATTGCTTTTCTAATTCCCTATGAATTTCACC
AACTCACACAAATAAATATACATGGCTTGACAAATAAATTTCAATTATCCAGACAAGCGG
CCCGAAATATTGTTTCTGCCTGCCCTACATATACTCTTACTCACCACATTCCTACAACTA
CAGGGGTTAATCCCAGAGGCCTTTATCCCAGTGATATCTGGCAGACTGGTGTTACTCATT
TCCTTCTTTTGGACATTTGGGAGCTGTTCATGTTTCTGTGGACTTTCTCCCTTCGCTTAC
CTAAATTACCTTATATTCCTTTACATTCAGAATTGTGGAAAATATCAGCTGCCTTCCAAC

CCATAACAGCATGGAAGGGGAAATTTAATTTGATAGTATCTGGTCATTCCTTACATTTTG
ATAAAAATTCAACCCATTGGATTAAAGCATGTGTTCATCTACCTTTTGTATTTTTGATAG
GACCTACTTTTTATAATGCCTCTTCTGGGTTACTTACTTGTCAAAATTGTTCCTTTTACA
CTTGTATCAATTCTCTCCAATTTCAAAATGACTATTCTCTATATATTATAAAAATCAGAT
CTGGAGTATGGCTTCCTGTAAAAATGAAAAAACCATGGCAGGATAGCCCCACAGTCTATA
TTGTAGAAGAAATCCTTAAGAAAGTGTTCAAACACACTGAACGGTTTATTGGACTGTTAA
GTGCTGCCATTTTAGGAATTATTGCTATAGCTACTACAGCAGCTGTAGCAGGACGGCATT
ACTTCAGAGTGTACAGACTGTGCACTTTGTGCAAGAATGGCATAAAGACTCTGATGTTCT
TTAGTCTACCCAGATGGAAAATTAGCTGCGTAAATGGCTGATTTACAACAGGTTGTTATA
CTACTAGGTGATCAAGTAAATAGTCCGCAAAAACAGATTTTTCTTAAGTGTGATTGGAAC
ATAACTTCTTTTCGTGTCACTCCTCACAAATATAATGAATCTTCATTTCATTGAGACAAA
GTTAAACAGCACCTCTTAAATCAAGGCAATATATCTCTGGATATTAATAATTTACAACAA
GAGATTATGGAAATTTTCTCTCAGAAACTACACCTTTTGCAAGGATCTAATCTGCTGGAA
GCAGCAGCAGATGGGATTTCTCAATTAAATCCCGTTCAACACTTGACAACTATCAGAGGA
TCAATGGCTGGTTTTATGTTAATATTTATATGTCTTTTTTTCTGTTTCTACATAGTCTGG
CAGTGAACCAAGAAAGCCAATGGACAGCAACAGCAATTAGTGACTATCGCTTTGGCTTTT
ATGATCAACTATAATCATCAAAAATAAGCTTCTAAAAATAAAAAGGGGGTGTGTAGGGAA
TATGCTATGCACAGGCCTGTACTATAATTAACAGGGCTTCTTTGAAGAATAAGAATGACT
TTCTCATGGCCCCCAGGCGAAGGGCTGGGAGCAGTAAAAAGTACATCGTGGAAAAGCATC
TTGAAAACAAGATGC
>CAP_ERV_27
TCAGGAAGCAACAGTTAGAACTGAACATGGAACAAAAGACTGGTTCCAAATAGCAAAAGC
AGTATTTCATGGCTGTGTATTGTCACCTGCTTATTTAACTTATATGCAGAATACATCATG
AGAAACACTGGGCTGGAAGAAGCACAAGCTGGAATCAAGATTGCCGGGAGAAATATCAGT
AACCTCAGATGTGCAGATGACACCACCCATATGGCAGAAACTGAAGAGGAACTAAAAAGC
CTCTTCATGAAAGTGAAAAAGGAGAGTGAAAAAATTGGCTAAAGCTCAACATTCAGAAAA
CGAAGATCATGGCATCTGGTCCCATCACTTCATGGGAAGTAGATGGGGAAACAGTGGAAA
CAGTGTCAGACTTTATTTTTTGGGGCTCCAAAATCACTGCAGATGGTGATTGCAGCCATG
AAATTAAAAGACGCTTACTCCTTGAAAGGAATGTTATGACCAACCTAAATAGCATATTGA
AAAGCAGAGACATTCCTTAGCCAGCAAACGTATGTCTAATCAAGGCTATGGTTTTTCCAG
TGGTCATGTATGGATGTGAGATTTGGACTGTGAAGAAAGCTGAGTGTGGAAGAATTGATG
CTTTTGAACTGTGGTGTTGGAGAAGACTCTTGCAAGTCTCTTGGGCTGCAAGTAGATCCA
ACCAGTCCATTCTAAAAGAGATCAGTCCTGGGGGTTCTTTGGAAGGAGTGGTGCTAAAGC
TGAAACTCTCATACTTTGGCCACCTCATGCAAAGTTGCCATATTGGAAAAGACTCTGATT
CTGGGAGTGACTGGGGCAAGAGGAAAAGGGAGCAACAGAGGATGAAATAGCTGGATGCAT
CACTGACTCGATGGTCGTGAGTTTGAGTGAACTCCGAGAGTTCGTGATGGATAGGGAGTC
CTGGCATGCTTCAATTCATGGGGTCACAAAGAGTCAGACACGACTGAGCAACTGAACTGA
ACTGAACAGAAGCAGAAGACATTAGGAAGAGGTGGAACGTATACACAGAAAATCTGTACA
AAAAAGAGCTTCAAGACTCAGATAATCACGATAGTGTCTTCACTTGTGGGGAGCCTCATT
AGGCATTACTGACAAAATGGAGGCATGACCTCAACTTCTTCTCTTTGTCCCGCAGGCACA
GACCCAGGATGAAGGATTTTGGCCTTGTGATTCTGACTTGTTTTTTCCTTTCCTCAGCTT
AGTTGACTGAAAAGAATATTAATGTGCTTATTGTTCTTGAGAGGAGCATGAGAAGGCACA
TCCTTCTGCTGTTGTGCTCAGAGAATTATTCATAATCATTGACATTTGTTCAAGGATCTT
TACAAAAAAATTGTCCCAGGATGAGCACGTAGGCCTCAGCTTGAGGCCATGGGAAGGATT
GTTATCTGAAACTTATTTGTAAGGGAAATGTTTACGGCAAAAGAGTTTACTGAGTTTAAG

GCTTAGGAATAATTAAAAATAGTTAGAAGCTAAAGATTTAAGTAACATTGTAATGTTAGC
ATATTTTGCTATAGCTTATAGAGATTAGGAATTTTAGAGATATTAATAGCTAGAAGCCTT
TTTAAGAGGTAGTGAGATCAGGATACTAGGGGCAAACAGATTTAGAAAGATAAGAAATAC
ATTGAGGTTTGTGTTATGCAGCCCAGATATGAGCATGAGTTACAATGTAATCACAAGTTA
AAGTAAACACAATTCTGAGAGTACTGCTGAAGCAGAAACTCTGCTTGAAGGACAACAGTG
ATTTATGGAGACAATAAATCTGGGTGAGGGGAAATTGAAAATGTCAAACCTCTGACCTAA
TGCTTTTGTAAAAGTATAAAAGAGAATCTAAAGCTTGAAATAAGCATACAGTCCAAGAAA
ACTGAGAGGCTGCATCATTACTCACCGACACCTCCCATCCTTTCAGGCTGATTCCCTGGC
TTCTGGACTCTGGCAGGTGGTGCCTGAATAGGAATATCAAACATTTGAAGGGTAAGTAAC
TTCGTGATTGTAGTGCAGGGTGGTTAGGATTGTACCTGTGGGTGGCCGGCACCCCTCTGC
AGTTTAGGCAGGGTGAGGAGGGTACAGAAGGAATAAAGAATATTCTCTAGGAAAGTAGGT
CTCTCTGCATCGAAGAATAGACAAATGTTTATTGAAATATTACTTCACATGTTAGCCCAT
AGAGGTGTTAAAGTAATCAAGGGAAGACTAAGTTTCTAATCTTTGTCCAAGAGCAATGCC
CATGGTTTCCTGAGGAAGGTACCGTAAATCTAGAAATGGACTAAGGTTGGAGATCAGTTA
CATTTGTTTCATACTGTATACGGCCCAGAAAATGTTCCTGTAGATGCTTTTGCTCTGTGG
ATTATGATCAGGGATGTCCTGGATCCTCGGCATGAGGCTGTTAAACAGCCTATGGGAGAG
GCTGGAGCAGTGAATGGTGGGTCTCCACCTTCTGTGCAGATCATGTTTTCTGCCACTGAC
GAAGAAAAGGAGGGAAACTGTTCTCAACCTCCTGAGGAAGGAGAGGGCTTGCAGGACACT
GCGTCCAGGGGCTCTAGTGAGCCTCCTCCCCCTCTATGCAAACCTTTGAAAGTTTATCCA
CCACTTTCTGATTTAAGGCGATTGCCACCACCTCCGTTTGTGCCTCCCAGGGCCCAGGAG
TTCCCCACTTTGCCCCCAAAGCCTCCCAGAGCATTGCCTGAGGCTGACGAGGATGAAGAC
TTTTCTGAAACAAAAGAGCTTTTGAGGCAGTCTCCTGTACAATATGCAGAGTGTGATCCT
TGGAGTAAACCGCCTAAGGGGGGGGGGGGGCTCACCCAGCCTAGAAGGGAGGCAGAACAA
CAGGGGGACTTGGAATTTTCTATGCCCTTTCCAGTAGCTTTTACCAAAACCGAAGGGGAT
GATAAGGAAAAAGGAAAACGGGAATCAATCCCATATAAGCTACTGAAAGACCTTAAACAA
GCCTGTCATGATTATGGGTCTACCTCCCCTTACACACTTACTCTGCTAGATGCCTTGGCT
GGGAGATGGATGGCACCATGTAATTGGAGAATGGTTTCAAAGGCTTGCCTTCCTGGAGGA
GAGCACCTCCTGTGGTTGACAGAGTATGATCAGTTGGCTAGACTACAGTCACCAGAAAAC
AAAACCTCTAATGATGCACAGCTCTGGGCAGTGGGATCTGCAGCATTAAAAGGAGATGGA
GAATACCAATCTAACATAACCCAAGCAAGGTTATCTAAAGAAGCACTTAATCAGATTATG
GCCATAGATGTCTTGACATGGAAATCTCTGCCCCCTTCAGATGGAAAATTATCCACTCTG
AATAATATCAAACAGGGACCCGATGAGAAATATGAAGAATTTGTGGCCAGATTGAAAACT
GCTGTAGAAAGAACTATTAAGAGTACTGACCCAGCAGAGATAGTTCTTAAACAATTGGCT
TATGAGAATGCTAATTCTACCTGTCAGGCTTTGCTTAGACCTTTCAGAAGTAAAGAAAGT
CTTTGGACTTACATACAGACCTGCCAGGAAGTAGGAACGTCCTTTATGCAGGGGGTTGCC
TTAGCCTCAGCTTTAAGGGAAGAAACAGCTGCCCGAGTTATTCAGGGAATGAGAAAAAAG
ATTAATCCTAATGGAAATGATTTTACAAATAAAAGGTTCTTCTCTTGTGGCCAAATGGGG
CATTTTTGCCTGTAATATCCTGCCAAGCAGGGACAACAAACTGTGCCAACACAGACTAAG
ACCAATCCCCCAAAGGCACATTGCCCCCCATGCCACAAAAGGGTATCATTGGGCAAAAGA
CTGTAGATCTAAATTCCATAAGGATGGGACTACGCTCACCCTCCAAGTACAGAGAGTAAT
TTTTCCCAGTTTCAAGGAAACGGGCAGCAGGGGCAGCCCTGACCCTGGACAACAATAGGG
GCAGCCACGTTGAATTCCTTTATTCCCTTTGTCCTGTCTCAGAAATCCCCAGAGCAACCC
CAGGTAACGCAGGACTGGACCTCAGTTCCACCACCACAACAATATTAATCCCTGATGTAC
TGGTTACTCCGATTCCCACAGGAGTGGCTGGCCCCTTACCTGAGGGCGTTGTAGGACTTG
CTCTGGGGTGTAGCTTGCTCTCCCTTCAAGGAATTTTGGTGGTGCCTGCTGTAGCAGATT

CTGATTATACTGGGGAAATTAAAGTTTTGATCTCTCCACCTATCAAAACTGTGCAAATTA
ATAAAGGTCAAATAATAGCACAGCTTTTGCTTTTACCTTATCATCAAACAGGAAGAACCT
TGGCTTCTTAAGCTAGGGGCCCCAGAGTATTTGGATCTAGTGATCTAGCCTTTTGGGTGC
AGGAAATTACAGCTTCAAGGCCTTTGAAAGATCTTTTAATTCAAGGAAATAAAATGTTAG
GGCTGTTAGATACTGGAACAGATATCTCTTGCATTGCTGGAAAAGATTGGCCCTCATCCT
GGCCAACATGCTTGACCAGTGCCGACTTGGTAGGAATAGGGTCAGTGCCCTAGGTTGCTA
AGAGCTCACAAATTTTGACATGGTCAGATGAGAAGGGCACAAGGCACCTTTTGTCCATAT
GTGATTACTTCACAACCTTTTTCTTTATGAGGGAGGGATATATTATCTCAGATGGGAATG
CTTTTATATAGCCCAGATGAAAAGGTTACTAATCAAATGCTGCAAATGAGGTATAATTCT
GATAAAGGACTTGGTAAAGATCAGCAGGGAATTGTTTCTCCATTAGAAGCAGTTCCTAAC
AAGAATAGAGAAGGTCTGGGATACTCAAATTTATCCTAAAGGCTGTTGCTCTTGCTGCCA
ACCCTATTACCTGGAAATCTGATGATTCGGTATGCGTGGAGCAATGGCCATTAACAGCTG
AAAAGCTGCAGGCAGCTGAGGATTTAGTTATGGAACAACTGGCAGGCAGGCATATAGAGC
CTTCTAATAGCCCCTGGAATATCTGTATTTTTGTTATTACGAAGAAATCTGGAAAATGGA
GATTGTTACAAGATTTGAGAGCTATGAATGCAACTATGGAAGATATGGGGGCCCTCCAGC
CAGGCCTCCCTTCCCCAGTGGCTGTGCCCTTTCAATATAATGTGATAGTTATAGATCTAC
AGGATTGTTTCGTTACCATCCTCCTGGCTGATCAGGATTGTAAACGGTGCTTTCAGTCTC
CCTTCAGCTAATTTTAAACAGCCCTATAGAAGGTTTCAATGGAAGGTTTTGCCTCAGGGA
ATGAAAAAATAGCCATACCTTGTGTCAGAATTTGTCAACCAAACTGTGCAAAATGTTAGAG
GAAATTATAAAGATCTGTATTTGACACATTATATGGATGATATTTTGGCTGCTCATAGGA
CAGAGCCTTGTTGCAACAAATATTATCTGAATTGATTGAGGCCTTGGAAAACTGGGGTTT
AAAGATAGTTCCAGATAAGATACGAGCAAATCCTCCTTTTTCTTATGTAGGGAGGGTATT
AAATACCCATACTGTGAGTCATGCTCCTTTGCAGCTGCAGAGAAGTCCTTTACTAACTTT
AAATGATTTCCAGAAACTATTAGGAGATATTGATTAGATATGCCCACATTTAAGACTGAC
TACTGCAGATTTAAAGCCTCTGTTTGATTGCTTAAAAGGCGATCCTAATCCCAGTTCTAA
GAGAGAATTGACTAGTGAGGCAGAGTCAGCTCTTGTTAGAGTGGATGAGGCTTTAAGTGA
TCAGTTAATTAGGATTAATATTATCAGAGGATGGGATTTAATTATTCTCACCACAGAGCA
TACACCTACAGGATGCTTGTGTCAAGAAGACCCATTGGAATGGCTCCATGTACAAGTGGC
CCTAAGAAAATAGTTTTGTATGATCCCAGCTTGGTGGCACAATTAATTATAAAACGTAGC
AAAAGAGGTGTGGAACTTTTGGGGAAAAAAATAGCAAATATTGTGATTCCATTCAATAAG
GATCAACTGCAATTTCTTTTACAAAATAGTGATGATTGGCAAGTTGCCCTGATAGATTCC
AGAGATCAAATTTTATTTCATTTGCCCTCAAACCCCCTTTTGCATTTTTTGAAAACACAT
CCAGTGATTTTTCTGAAGAAATTTTCTATTCAACCTTTGGAAAGGACAATTTTACTGTTC
ACAGATGGTTCCTCTAATGGTAAAGCAGTCACAATTATTATTGGAAAATCCCATGTTCAA
GTAACTGAAGAGACATCTGCCCAGAGGGCTGAGTCAAGAGCAGTTATTTGGGGTTTTCAA
CATGTAAGAGACTGTACCTTCAATCTTTTGACTGCCTCCCGATATATTGTAGAGTTATTT
CCTCATATTGAGACTGCTAATATTTTGGAAAATAAAGCTATAATCTTCTCCTTGTTATCT
GATTTACAGAAGGAGATTAAACATAAAGATAAGAAATATTTTGTGGGACATAGTGGAGCC
CATTCCAGCTTGCCCGGCCCTTTGCATGAAGGAAATGCTTTGGCAGACGCCTTAACTAAA
GCAATTGCTTTAAACTTACATAAAAAGATTGACAAGGCCAAAAGTTCTCTCAAAATTCAC
CATCAAAATGCAGCTGGTTTAAGGTATGAATTTCATATCTCCAAGGAAGCAGCTAGACAA
ATAGTTAAATTGGGTTCAAATTGCCCAATATTTAATACATCTCTACCATTAAGAGTAAAT
CCCCGAGGCCTTAGGCCTAATGCTCTCTGGCAAATGGGCATAACTCATATCCCAGCCTTT
GGAAAACTGTCCTTTCTGCATGTTACCATGGATACCTTTTCTCATGTTATTATAGCCTCT
GCTAGATCAGGTGAAGGAGCAAGAGATGTAATTCAGCACTTGTTTCAACAGCTTTTCCCA

AATAGGAGTCCCCAAACAGATAAAAACAGATAATGCCCCAGCTTATACTTCTGCTGTTTT
TAAGAGATTTTTGTCAACAATTTTCCATAGTACATTCAGCAGGAATACCTTATAATCCCC
GAGGCCAAGCCATAGTGGAAAGGGCACACCAGACTTTAAAAAATCAGATCGATAAATTAA
CACAGGGAGAATTTAAGTATTCCTCTCCACGTCATGTTCTACACCATGGTTTATTTGTAA
TTAATCATTCAAATATGAACACACAGGGGCAGACTGCAATGATGAGACACTGGATTCCTG
AAGGGGCTATGACTCAGGTTCTGGTTAAGTGGAAAGACATCCTTATCGGAGAGAGGAAAG
GGTCAGATATGCTGTTAAACTGCAGGAGAGGGTATGCTTGTGTGTTTCCACAGGATTCAA
CTTCTCCTGTTTGGATTTCTGACAGACTGATTCGACATGTCCAGTCCCATGAGACCTCCA
GGATCACAAAGACCCACAAGGTCACTGAAATCTCTGGAGTCCTGGAGTCAGGGACTGAGG
AAAGATGGGGAGACCAGAAGTTCGTCGAGTCCAGACCTGAGGGAGCCAAGGAAAAGTGAA
AGGGCGAGTGAATCTTCACCTGAGGCTTCACTGCTAGTACATCACTTCAGGCACCTGGAG
CTTCAAGAAGAGACATGCTGATCAAGTCCAACCGTATTGTGCTCCATCTCGTCACTGTCA
TTGTACTCAAACAACTACCTCCAGCACGGTACCAACTTGGGGCCTAAAACATCTCACTCA
GCAGGCTGAAGAATTAACAAAAAGGGGGAGACATGAGGCCACTCCCATGGTAATGTTTGT
GCTATGGAGGTGATGGAATACCAGTTGACCTGTTTCAAATCCTGAAAGATGATGCTGTGA
AAGTGATGCACTCAAAATGCCAGGAAATTTGGAAAACTCAGCAGTGGCCACAGGACTGGA
AAAGGTCAGTTTTCATTCCAATTCCAAAGAAAGACAATGCAAATGAATGCTCAAACTACC
GCACAATTGCACTCATCTCACATGCTAGTAAAGTAATGCTCAAAATTCTCCAAGTCAGGC
TTCAGCAATAGGTGACCATGAACCCCCTGATGTTCAAGCTGGTTTTAGAAAAGGCAGAGG
AACCAGAGGTCAAATTGCCAACATCCGCTAGATCATCAAAAAAGCAAGAGTGTTTCAGAA
AAACATCTATTTCTGCTTCATTAACTACGCCAAAGCCTTTGACTGTGTGGATCACAAGAA
ACTGTGGATAATTCTGAAAGAGATGGGAATACCAGACCACCTGACCTGCCTCTTGAGAAA
CCTCTATGCAGGTCAGGAAGCAACAGTTAGAACTGGACATAGAACAACAGACTGCTTCCA
AATAGGAAAAGGAATACGTCAAAGCTGTATATTGTCACCCTGCTTATGTAACTTATATGC
AGAGTACATCATGAGAAACATTGGGCAGGAAGAAGCACAAGCTGGAATCAAGATTGCCGG
GAGAAATGCCAATCACCTCAGATATGCAGATGACACCACCCTTATGGCAGAAAGTGAAGA
GGAGCTAAAAAGCCTCTTGATGAAAGTGAAAGAGGAGAG
>CAP_ERV_28
TGGACTGCAAGGAGATCCAACCAGTCCATTCTGAAGGAGATCAACCCTGGGATTTCTTTG
GAAGGAATGATGCTAAAGCTTAAACTCCAGTAATTTGGCCACCTGATGCGAAGAGTTGAC
TCATTGGAAAAGACTCTGATGCTGGGAGGGATTGGGGGCAGGAGGAGAAGGGGACAACAG
AGGATGAGATGGCTGGATTGCAACACTGACTCGGTGGACATGAGTCTGAGTGAACTCCGG
GAGTTGGTGACAGACAGGGAGGCCTGGCATACTGCGATTTGTGGGGTCGCAAAGAGTCAG
ACATGACTGAGCAACTGAACTAAACTGAACTGAACGCCCAATGGCAAATAATACCTCAGT
AGGAAGGCATTAATTTTTCTCACATGCAACAAATGAAAAAAGCTGTAACTATGTATGGCT
GTCATTCACCTTTTACTAAGAAACTTTTAAACACTATGGCATCTTCTCCTGGAAATTTTG
CAACTTATGATTGGCGACTTTTGATAAGCGCTCATCTTAAACCGGGAGAATATCTTCAGT
GGACCATGTGGCTTCAGGGTATGGCCCAAGATCGTGCCAATTCTAATGCCCGAGCTGGTA
CTGCCCAAAAGCAAATTACTTTTGAAATGTTAACTGGTACAGGGCAATGTAATGCTATAG
AAACTCAAATACAAAGCCCTCCTTGGTGGAAAACTATCTTGACTGTAATAAATGTAGTCA
TCTGGTTCTTGCTGTTTGCATTTGTATCTGTAACTATATAATGTACTTTGTATCTAAGTA
ACTTAGGCATTTACATTACAAGTGATTGTTCATGCTCCTGCAGTTTCCGCTACCTCCTCT
AGCTATTACTCAGGGCCACTGGATCAGAGACCCTCAATAGTAGGATGAGGAGAATATGTT
GCCTCAACAATTTAGGGTCTATGCCCCTTCCCGGCAGGAAGAAGTTACAGAACGATTTCT
CCACCCCTTTCCCTGCCAACACTATTCTCCTAAAAGCAGGAGGAAATGAAAGAGTTAATG

CTTAGGCAGGCAGTCCAAGGCCCTGTAAGGAGGAGACAAACATTCTCACTCATGCTTTCC
TTAAGCCTTGTGCAGCAACATAGCTTGCCAGAGAACTGACCTTTCTTTAGGTCCAGAACT
AATGATTAGACAGCCGAATGTCTTACTCATGGAAATGCTTTTCTTAGGCTCTATGGTAAT
GATTATAATTGTAACAAATCTTGCCTGGGAACCTATATCTCAAGACTTGTACCCCTGCTT
ACACAGCAACAGTATATCTTGCATAGAGACATTGGCCGGAACCTGTTCTCCCTGGCTAAT
CTTGTACCAAAGTTATCTCAGGATGTATGTCTTGGGAAAGGGCCTAATAGACCTCTTACA
ACGTTGAGGTATTCTTTTTATCTGTTCTCAGCAGCTAGTTGAGAAGTATAGAAGGTCCCA
CTTAAACTAGTGAAGTGGATACTCTTTCTGCCCCCACAAACTTTCTGCCTTTTACTTGGC
CGATCTAGTTTGACTTCTAAAGGAATTAGTGTTCACCCTGGAATAATTGATTCAGATGAT
GAGGGAGAAATTCAAATTATGATGTCATCTCAGATACTATGGCGTTCAAAAAGGGGGCAA
GATGGCCCAACTACTCCCTTCACCTTACATGCCTATTAGCTCCTCTAATGGCGTCTGCAC
AGGTGGATTTGGCGGTTCAGATCAAAAACAGTCCTTATGGACGTCCATAGTATCTGAATG
TGCACGACCGAATATAAATATCAAAATTAATGGCAAAAGATTTTCTGGTCTTCTTGATAC
TGGATCTGATATTACCATTATTTCCAAATATTTATGGCCCAAATCTTGGCCTATACAGAG
AATTTCTTGCCAAACTGCAGCAGTTTCTCAAACCAAAGCACAAGAGGTTTATCAAAGTGT
CCAAATATATCCATGTGAGGGACCAAAAGGCCAGCCTGCAACATTACAACGTTATGTGAT
AGGTACACCCCTCAATTTAATATGAAGAGATTTACTTATGCAACGGCAAACTCAAATATA
TATTCCACATTTTTCCTAGGGGCCACTGCTCACCTAACAATCAATGCAATTATTAAAATT
ACTTGGAAAAATGACGAGCCTATTTGGACAGAGCAATGGCCCCTCACAAAAGATAAATTG
GAGGCTACTAAAGAACTTATCAACACAATGGAAATCACAGCATATTGAGGAATCTTATTC
CCCTTGGAATTTTCCCATTTTTGTAATAAATCTGGCAAATGGCATCTCTTAACAGACCTT
AGAAAAATTAAAACATCTATGAAAGTTATGGGTGCATTGCAACCAGGAATTCCATCACCT
GCCACTATCCCTCAAAACTGGCATATTATTATTATAGGTTTACAAGGTTGCTTTTTTACT
ATAGTTTTACACCCTCTAGACCCAGACAGATTTGCTTTCTTTCTTCCTTATTCTAATCAT
GCTGCTGCTACTGCTGCTAAGTCACTTCAGTTGTGTCCGACTCTGTGCGACCCCATAGAA
GGCAGCCCACCAGGCTCCTCTGTCCCTGGGGTTCTCCAGGCAAGAACACTGGAGTGTGTT
GCCATTTCGTTCTCCAATGCATGAAAGTGAAAAGTGACAGGGAAGTCGCTCAGTCGTGTC
TGACCCTTAGCATGGACTGCAGCCTACCAGGCTCCTCCGTCCATGGGATTTTCCAGGAAA
GAGTCCTGGAATGGGGTGCCACTGCCTTCTCCGATCCTAATCATATCGGGCCTAATAAAT
GATATCAGTGGACTGTATTGCCTCAAGGAATGATGAATTCTCCCACCGTGTGTCAATATT
ATGTAGCCAAAGCCCTTGAACCTGTGAGAAAACAATTTCCTAACTTTCTTGTTATTCATT
ATGTGGATGATACATTGTCTTCAGCTCCCTGTGTTTTAGAAACTGAACAAATGTTTGACA
TAGCTCAACAGTGCTTGGAAGACTCTGGATTAAACATTGCTCCTGAAAATATTCAAACCT
CTACTCCTTACCATTACTTAGGCTCTGTTGTTAATAGACAACGTATTACTCCCCAACTAA
CAGATTCATGTTAATAAATTATCAACCTTGAATGATTTTCAGAAACTTTTAGGTTATATA
AACTGGATTAGACCCACTTTAGGCATTGCAAATGATCAACTGACTAATTTATTGAATACT
TTAAAAGGAGATCCCTATTTAAATAGCCCTTGTTTGGTACAAAATAAATTCCAAAAACAG
TTTCTTACTCGTATTAGACTTGATTTACCTCTCGAGTTGTTTATACTCCCTTCTCTCTAT
TCCCCGACGGGCCTTCTTGCCCAACAAGAACACCTGATAGAATGGATCTCTACCCATTTT
AGAGGGACGAGGTCACTTACGCCCTATATTGATTTGATCACTCTAATAATTATCAATGGG
AGAAATAGAACTAAGACACTAATTAGTTCGATCCTCATAGCATTGTAATTAATAAACCTC
AATTTGAGAATGCACCACAAACGTCTACCAATTTCCAAATAGCCTTTCTGGAATATTTTA
AAGATATTTCATTTCATAATCCTTCTAACAAACTCTGAAATTTCTTGAAAAATACTGAAT
TTATTATCTCTTGCATTATCACATTACAACCTATACCTCGAGCTGATGTTTTCTATATAG
ATGGGACTAAAAACACCAAAGCCTCTTTCTGGTCTGTCGAAGAATACAAAGTCTTTTATA

CTAAATTTCATTCTGCTCAACAAAACGAATTATATACTCATTCAAGTTATTCACCTACAC
CCTTACCTTATTAATATAGTCTCTGACTCTTTATATTCAGTTTTTATATTAGGAAATATA
GAAACCTCTATTATAAATTCCAACCAACCTATTATTCAACAAGTTTTTCTCGAACTACAG
TCTCTTATTAGGGACTGCACTTCCCCCATTTACATTACCCATATTCGAACACATTCCTGC
CTTCCTGGCCCTATGACTCATGGTAATGAACAAGCTGATAAACTTGTTTCTTTTGCTACT
CCCGAGGAACAACATGCTCTATTGTACAATAATGCTGGCTCCTTAAACCAAATTTAGAAA
ATTCCATACTGGTGGACTGCAAGGAGATCCAATCAGTCCATTCTGAAGGGGATCAGCCCT
GGGATTTCTTTGGAAGGAATGATGCTAAAGCTGAAACTCCAGTACTTTGGCCACCTCATG
TGAAGAGTTGACTCATTGGAAAAGACTGATGCTGGGAGGGATTGGGGGCAGGAGGAGAAG
GGGACGACAGAGGATGAGATGGCTGGATGGCATCACTGACTCGAAGGGCGTGAGTCTGAG
TGAACTCCGGGAGCTGGTGATGGACATGGAGGCCTGGCGTGCTGCGACTCATGGGGTCGC
AAAGAGTCGGACACGACTGAGCTACTGAACTGAACTGAACT
