# Supplementary material for: Evolution of Endogenous Retroviruses in the Subfamily of Caprinae
Source: Viruses. 2024 Mar 4;16(3):398. doi: 10.3390/v16030398 (PMC10975924; doi:10.3390/v16030398)
Supplement: Supplementary file 1 [file viruses-16-00398-s001.zip › 3-Supplementary File (3)-Figures.pdf]

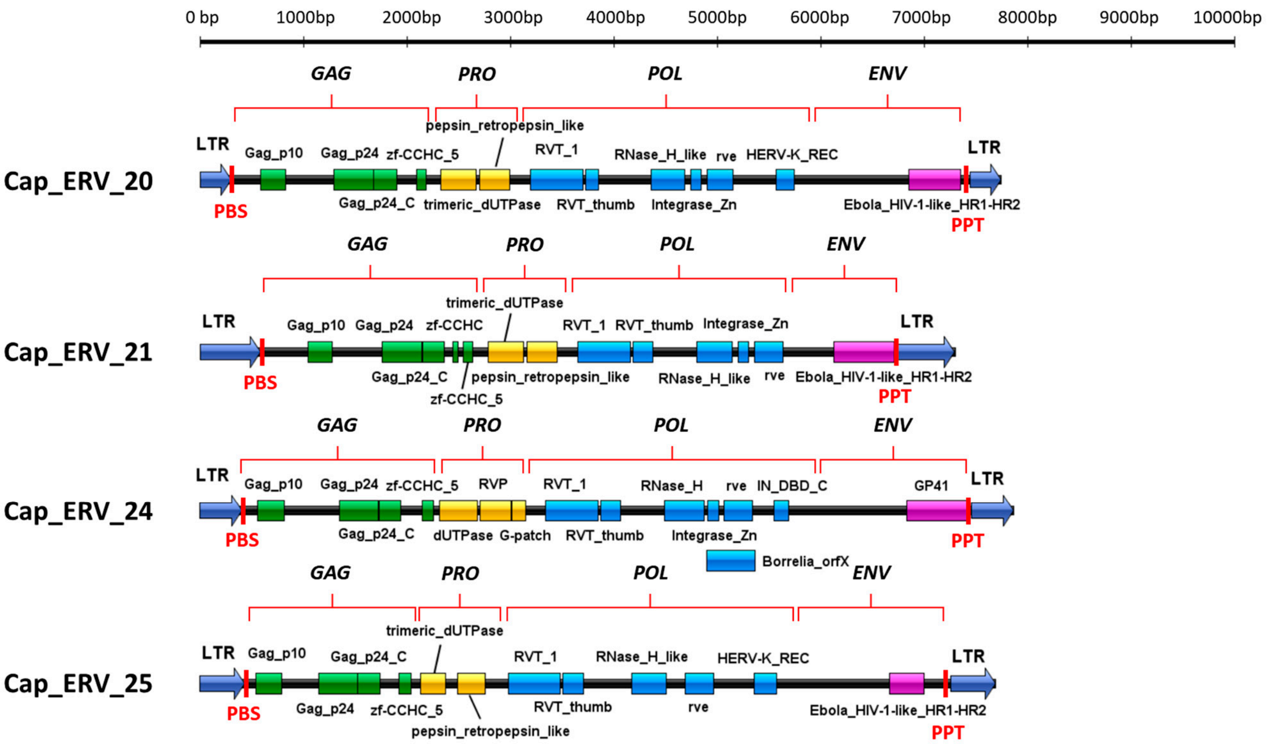

Figure S1. Structure organization of "active" ERVs. **Gag_p10**: Retroviral GAG p10 protein; **Gag_p24**: gag gene protein p24 (core nucleocapsid protein); **Gag_p24_C**: Gag protein p24 C-terminal domain; **zf-CCHC**: Zinc knuckle; **zf-CCHC_5**: GAG polyprotein viral zinc-finger; **trimeric_dUTPase**: Trimeric dUTP diphosphatase; **pepsin_retropepsin_like**: Cellular and retroviral pepsin-like aspartate proteases; **RVP**: Retroviral aspartyl protease; **G-patch**: G-patch domain; **RVT_1**: Reverse transcriptase (RNA-dependent DNA polymerase); **RVT_thumb**: Reverse transcriptase thumb domain; **RNase_H_like**: Ribonuclease H-like superfamily; **Integrase_Zn**: Integrase Zinc binding domain; **rve**: Integrase core domain; **HERV-K_REC**: Rec (regulator of expression encoded by corf) of HERV-K-113; **Ebola_HIV-1-like_HR1-HR2**: heptad repeat 1-heptad repeat 2 region (ectodomain) of the transmembrane subunit of various endogenous retroviruses (ERVs) and infectious retroviruses, including Ebola virus and human immunodeficiency virus type 1 (HIV-1): **GP41**: Retroviral envelope protein.

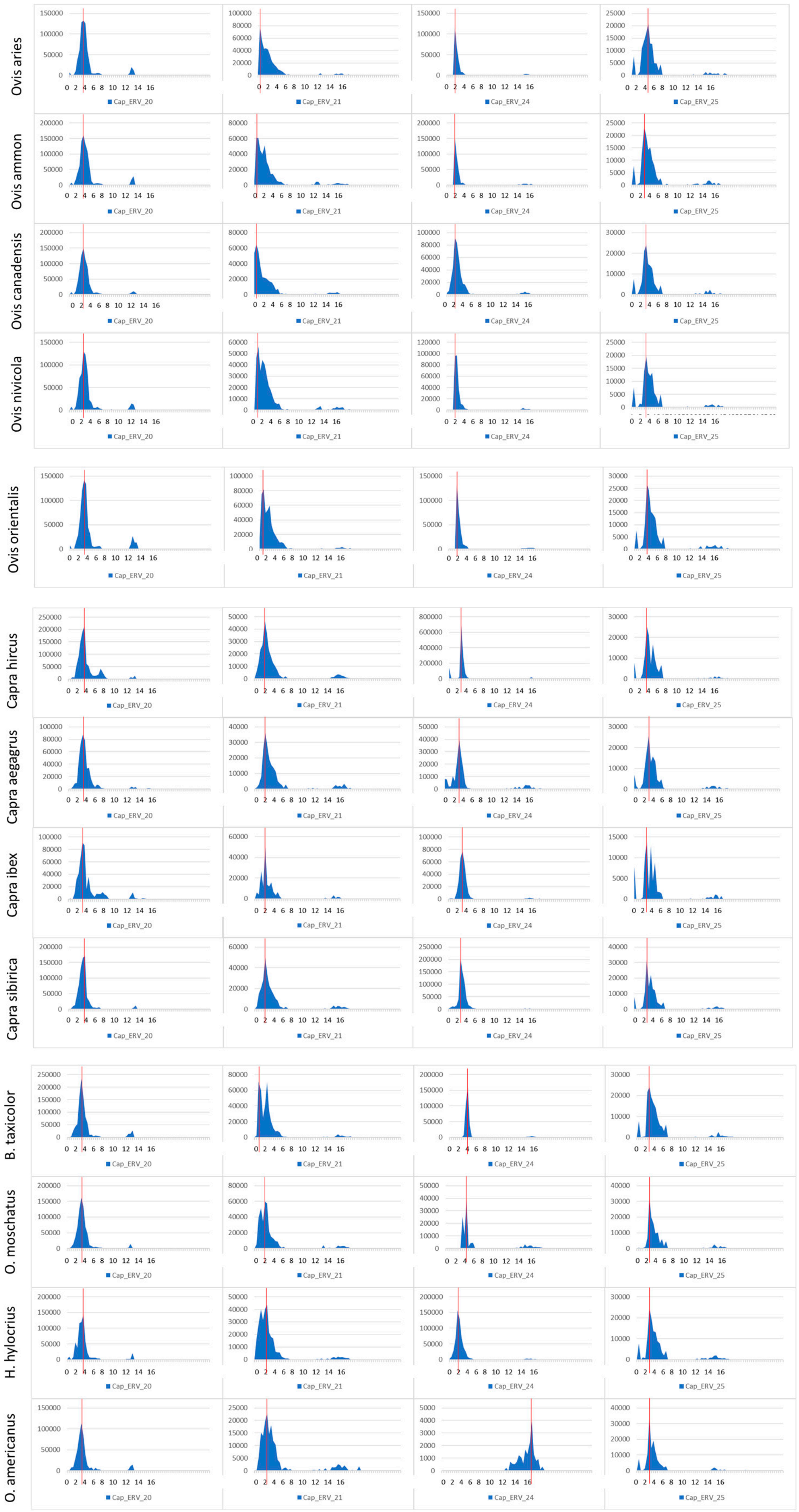

Figure S2. Divergence analysis of four full-length ERVs among the 13 species of the Caprinae subfamily.
